# Direct formation of amide/peptide bonds from carboxylic acids: no traditional coupling reagents, 1-pot, and green†

**DOI:** 10.1039/d3sc00198a

**Published:** 2023-02-28

**Authors:** Kaitlyn M. Freiberg, Rahul D. Kavthe, Rohan M. Thomas, David M. Fialho, Paris Dee, Matthew Scurria, Bruce H. Lipshutz

**Affiliations:** a Department of Chemistry and Biochemistry, University of California Santa Barbara CA 93106 USA lipshutz@chem.ucsb.edu

## Abstract

Technology for generating especially important amide and peptide bonds from carboxylic acids and amines that avoids traditional coupling reagents is described. The 1-pot processes developed rely on thioester formation, neat, using a simple dithiocarbamate, and are safe and green, and rely on Nature-inspired thioesters that are then converted to the targeted functionality.

## Abstract 2

Technology for generating especially important amide and peptide bonds from carboxylic acids and amines that avoids traditional coupling reagents is described.

## Graphical abstract

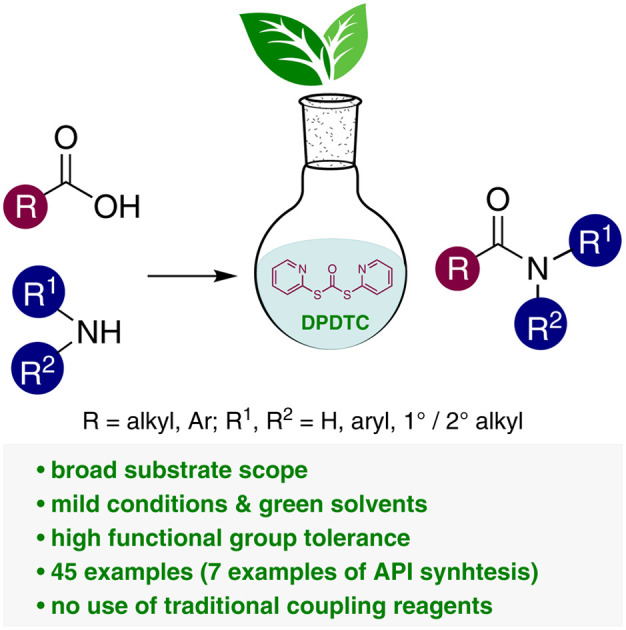

## Introduction

**Fig. 1:**
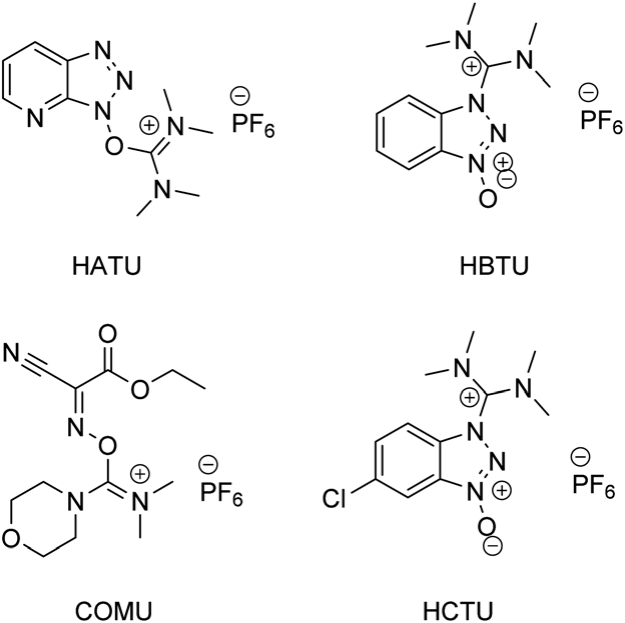
Commonly used amide/peptide coupling reagents.

According to a paper back in 2016 by Brown and Boström, amide/peptide bonds are the number one type of construction practiced in medicinal chemistry.^1^ A virtual toolbox full of coupling reagents has been accumulated to meet the many challenges posed by reactions between carboxylic acids and amines which formally release an equivalent of water. Some of these reagents are quite aged while others are of relatively recent vintage, including those that are “green” in nature (*e.g.*, see Fig. 1, and the very recent review in ref. 2). Although most of these reagents in use are now readily available, and in many cases considered even inexpensive, there can be no argument about the by-products formed, which may present separation issues. Most, with perhaps the exception of COMU leading to polypeptides formed in an aqueous medium,^3^ have also been utilized in strictly waste-generating organic solvents. Particularly noteworthy is the well-known early warning on the explosiveness of HOBt, and the more recent report by Nowick and co-workers^4^ highlighting the non-trivial safety issues (especially anaphylaxis) that surround use of common uronium couplings agents HATU, HBTU, and HCTU. Thus, while applications of these dehydrating agents continue to appear unabated due to the importance of the amide/peptide unit, there is more than ample justification for devising alternatives that avoid these issues in their entirety;^5^ indeed, a truly green chemical solution applicable to highly functionalized molecules and that can be used in a sequential fashion in the same reaction vessel seems long overdue.^6^

In this report, therefore, is presented a 1-pot solution that appears to be general, *safe*, and very responsible in terms of its impact on the environment, and which allows for applications to several challenging yet representative targets within the pharmaceutical industry. At the heart of this approach lies recognition of the simple question: How does Nature make these bonds? And while one can quickly gain an appreciation for the complexities of such constructions, the overarching conclusion is that thioesters are usually key intermediates along the biosynthetic pathway to the desired functionality. The choice of the “right” thioester loomed large in developing this technology, since the required bonds must be formed using water as the reaction medium, which is Nature's chosen “solvent.” Prior art, albeit *not related* to amide/peptide bond formation involving thioesters certainly exists,^7^ and here, the work of Fukuyama stands out,^8^ although such *alkyl* thioester intermediates, made in, and isolated from, organic solvents are inappropriate for the goals at hand.

They typically involve initial acid chloride formation, and the derived thioesters are considered mainly as educts for reductions to aldehydes, as well as intermediates en route to ketones.^9^ It was also important to acknowledge and deal with the common criticism associated with much of sulfur chemistry; that is, the potential odor. Moreover, the reagent to make the thioester of choice had to be readily available and inexpensive, while the efficiency of its use in thioester formation had to be such that the overall conversion to the amide/peptide would be high-yielding.

**Scheme 1:**
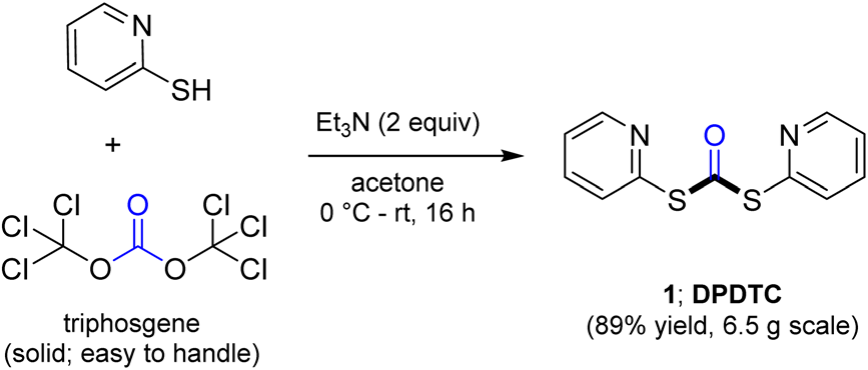
Formation of DPDTC for use in thioester formation.

Ultimately, the ideal thioester was that containing the 2-thiopyridyl residue, where DiPyridylDiThioCarbonate, or DPDTC (1) is readily derived in multi-gram quantities from treatment of commercially available 2-mercaptopyridine with triphosgene (Scheme 1), following an old recipe used to arrive at several common reagents (*e.g.*, CDI) at scale.^9^ This odorless dithiocarbonate (DPDTC) had been made decades ago,^10^ and more recently, related albeit limited applications have also been reported by Lee.^11^ Preparation of thioester intermediates is typically performed in organic solvents (*e.g.*, CH_3_CN), and usually isolated prior to conversion to various functionalities. The corresponding 1-pot process is also known in a single case.^11^ Hence, reagents such as DPDTC are rarely used, and could rightly be considered today, as far from sustainable technologies.

## Results and discussion

**Scheme 2:**
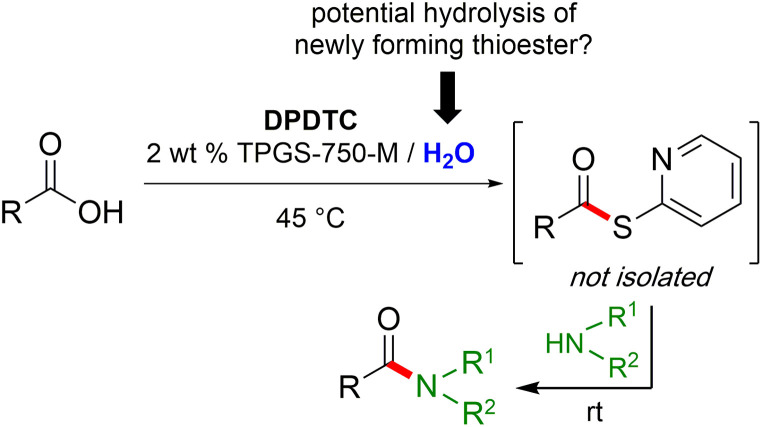
Thioester formation in water followed by conversion to amides/peptides.

**Scheme 3:**
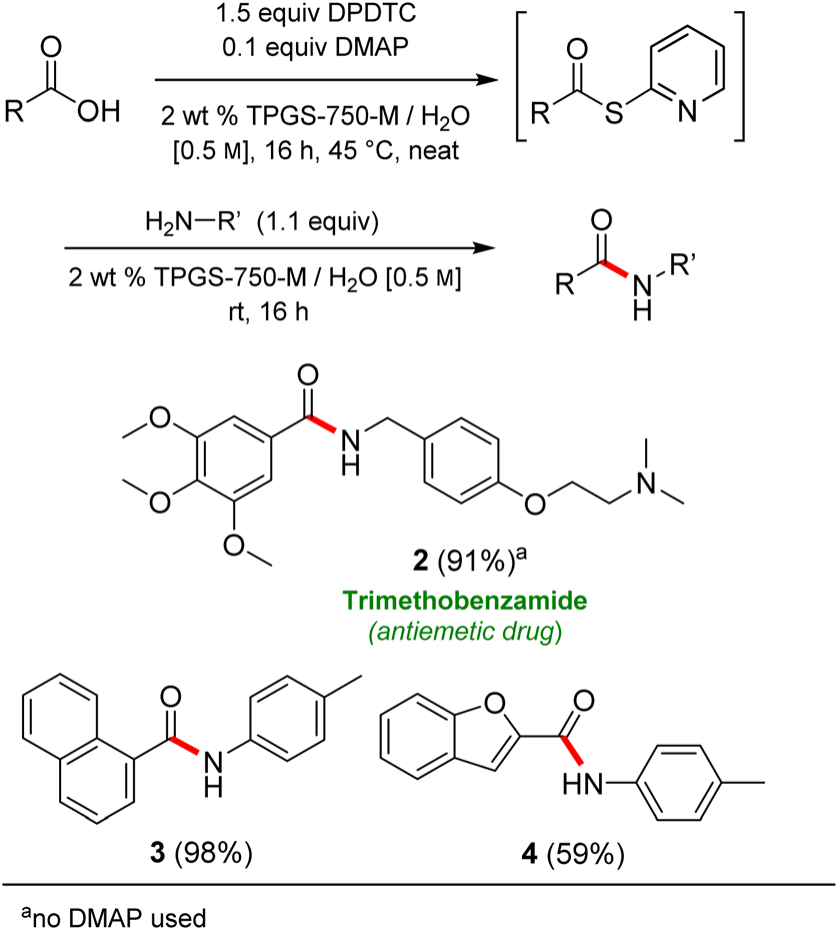
Representative examples of amide bond formation in 1-pot, all in water.

Formation of a 2-pyridylthioester using DPDTC could be accomplished in an aqueous micellar medium, as shown in Scheme 2. While such intermediates are isolable and have considerable shelf life, it was very straightforward to treat these *in situ*-formed thioesters directly with an amine to form the corresponding amide. And while this approach offers the desired simplicity and fulfils many of the criteria for “greenness”, the potential for competitive hydrolysis of the thioester back to its precursor acid imparted an element of unpredictability given the aqueous medium involved. Hence, as shown in Scheme 3, isolated yields of targeted amide could vary substantially, even when catalytic DMAP was present to enhance the rate of initial thioester formation.

**Scheme 4:**
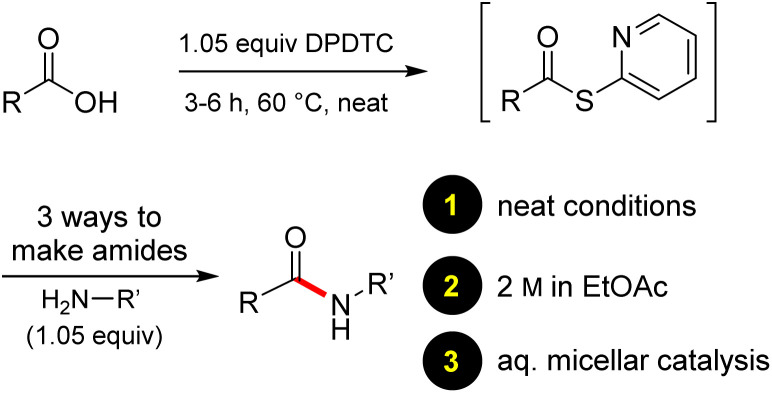
Approaches to amide formation from the intermediate thioester.

**Scheme 5:**
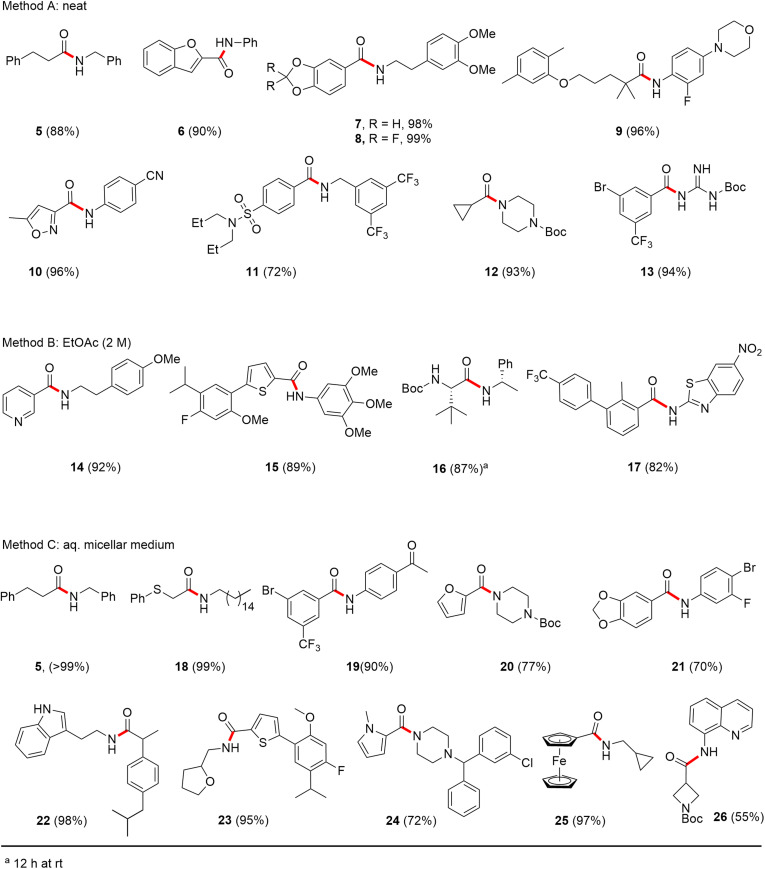
(A) Examples of amide bond formation using neat conditions (method A). (B) Examples of amide bond formation using highly concentrated mixtures in EtOAc (2 M; method B). (C) Examples of amide bond formation using thioesters to which is added an aqueous micellar medium (method C).

**Scheme 6:**
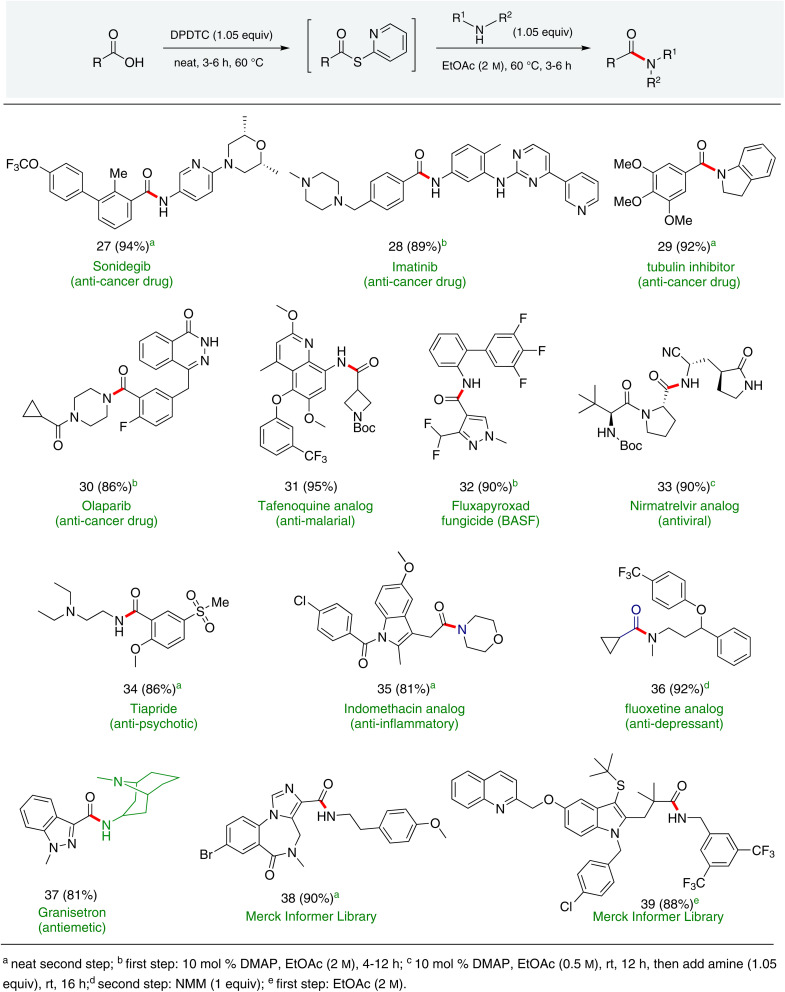
Amide/peptide bond formations featuring highly functionalized reaction partners.

In full appreciation of the guidance offered years ago by Sheldon and co-workers, in whose monograph *Green Chemistry and Catalysis* it states: “The best solvent is no solvent…”,^12^ we found that by adding DPDTC to the acid (solid or otherwise) followed by gentle heating (*i.e.*, neat) to *ca.* 60 °C, DPDTC melts leading to the desired thioester, which is formed cleanly (see ESI, Table S1†). At this stage, and without thioester isolation, three different approaches were developed for subsequent conversion to the corresponding amides/peptides (Scheme 4): (1) direct addition of an amine (neat; see ESI, Table S4†); (2) use of concentrated solutions of the amine in EtOAc (2 M; see ESI, Table S4†); and (3) dilution with aqueous 2 wt% TPGS-750-M^13^ (see ESI, Table S9†); followed by introduction of the amine in the presence of small amounts of co-solvent (*e.g.*, EtOAc), which can aid in maintaining an emulsified reaction medium. Surfactant MC-1 is also viable for more polar cases.^14^ Examples using each of these 1-pot procedures are shown in Scheme 5A (neat conditions), 5B (in 2 M EtOAc), and 5C (in aqueous TPGS-750-M). These were all chosen to document the overall efficiencies to be expected regardless of the approach selected. It should also be appreciated that both amides and peptides (*e.g.*, 16, along with the application of this technology to nirmatrelvir)^15^ are amenable, and are formed without loss of stereointegrity. Tolerance to a very wide array of functionality within these cases is also noteworthy. Method B, that relies on concentrated solutions of EtOAc, was selected since recovery of this single greener organic solvent is readily achieved. Likewise, when using an aqueous micellar medium (method C), its recycling following amide formation and after an extractive work up with recoverable ethyl acetate is routine. Moreover, the process associated with method C could be smoothly scaled to the gram level to give 5 in 94% isolated yield (see ESI, section 8†). Several additional applications of this 1-pot sequence have been made with a focus on targets in the pharmaceutical area. Thus, as illustrated in Scheme 6, compounds of known bioactivity, such as sonidegib (27), imatinib (28), olaparib (30), antimalarial tafenoquine analog 31, and fluxapyroxad (32) are all prepared in very good isolated yields. Highly functionalized drug analog 33, related to polypeptide nirmatrelvir, and as with nirmatrelvir itself,^15^ no indication as to the loss of stereointegrity (*i.e.*, epimerization) was observed. Also, included within this same study are two amides (38) and (39) derived from heavily functionalized educts within the Merck Informer Library,^16^ suggestive of opportunities for late-stage functionalization. Taken together, these examples are indicative not only of the quality of the bond constructions and functional group tolerance, but importantly, the environmentally responsible conditions under which such couplings are now possible. In comparison to literature methods, this 1-pot conversion not only allows for an isolable and stable intermediate, but also affords equal or better yields, as demonstrated in Table 1 with representative compounds 6, 21, and 42.

**Table 1:** Comparison cases with existing literature examples

Substrate	This work	Literature
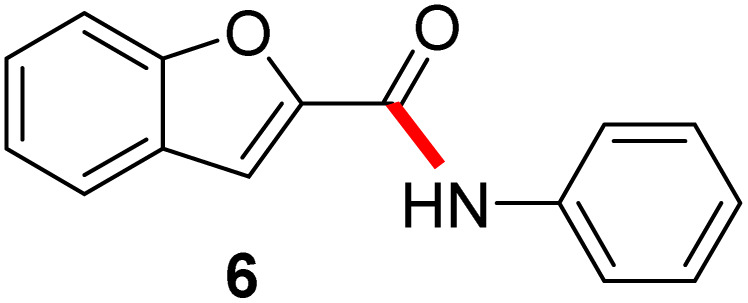	90%	87% (ref. 17*a*)
		77% (ref. 17*b*)
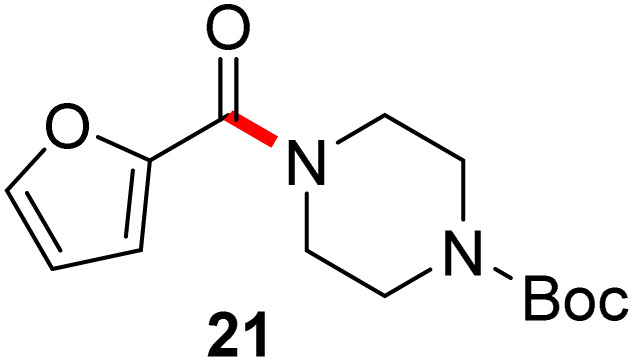	77%	71% (ref. 18)
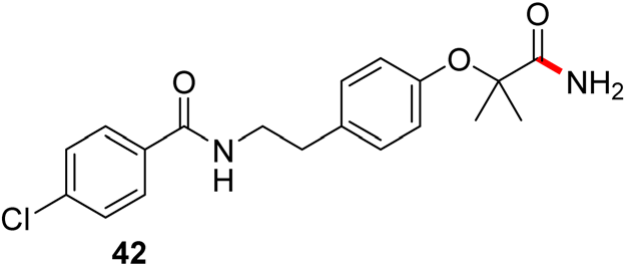	80%	40% (ref. 19)

**Scheme 7:**
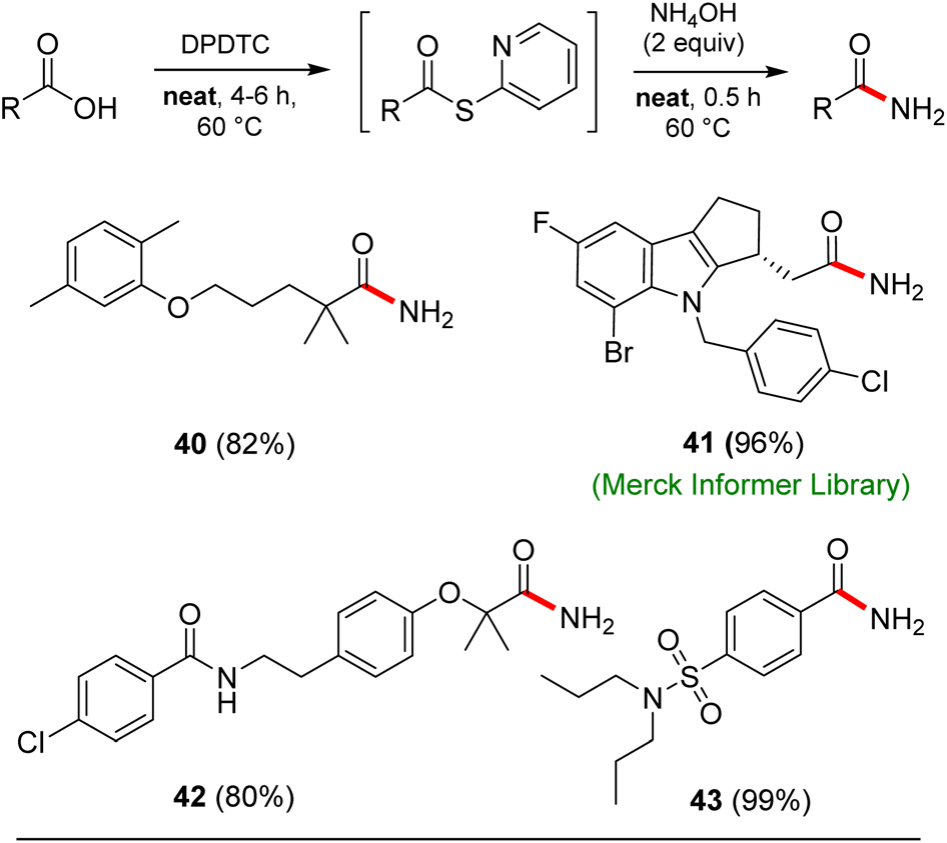
Examples of neat conditions applied to formation of primary amides.

Opportunities also exist to form primary amides using this same approach. Examples of four such isolable products are shown in Scheme 7. In these cases, after formation of the intermediate thioesters, addition of aqueous ammonium hydroxide (2 equiv.) afforded the targeted amides in good yields.

**Scheme 8:**
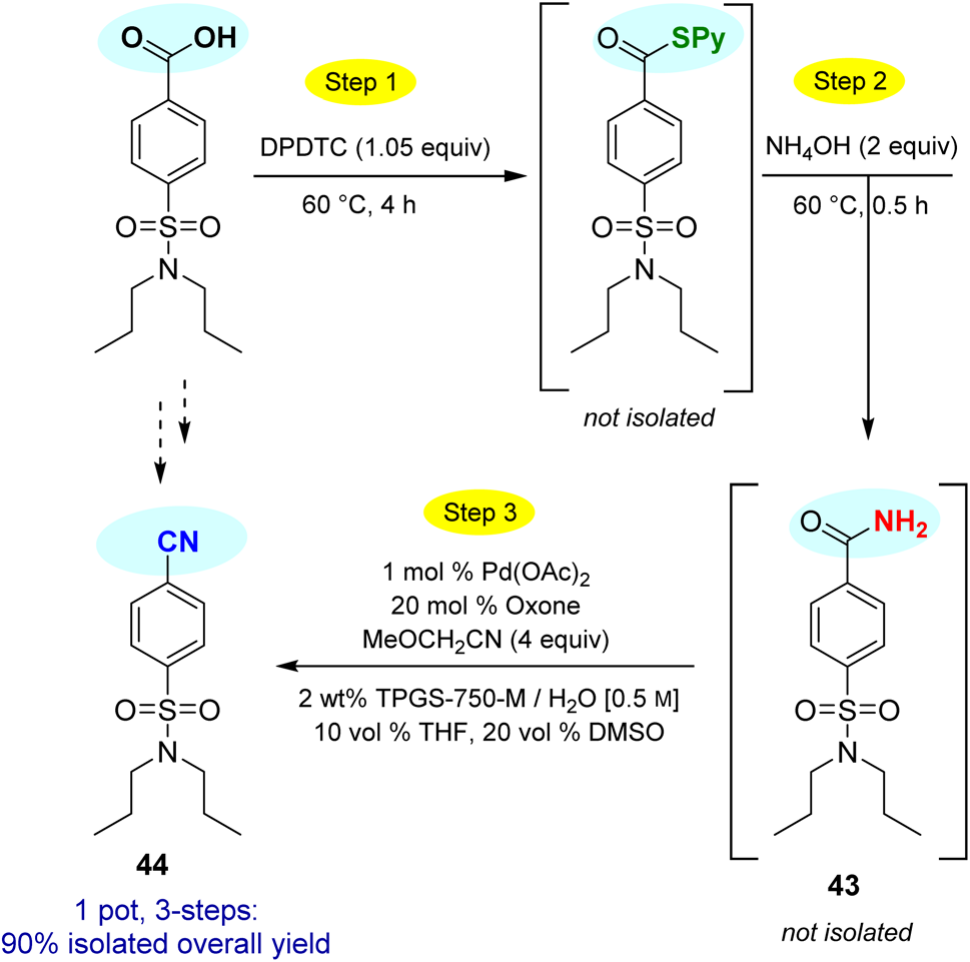
1-Pot conversion of a carboxylic acid to its derived nitrile *via* Pd-catalyzed dehydration.

**Scheme 9:**
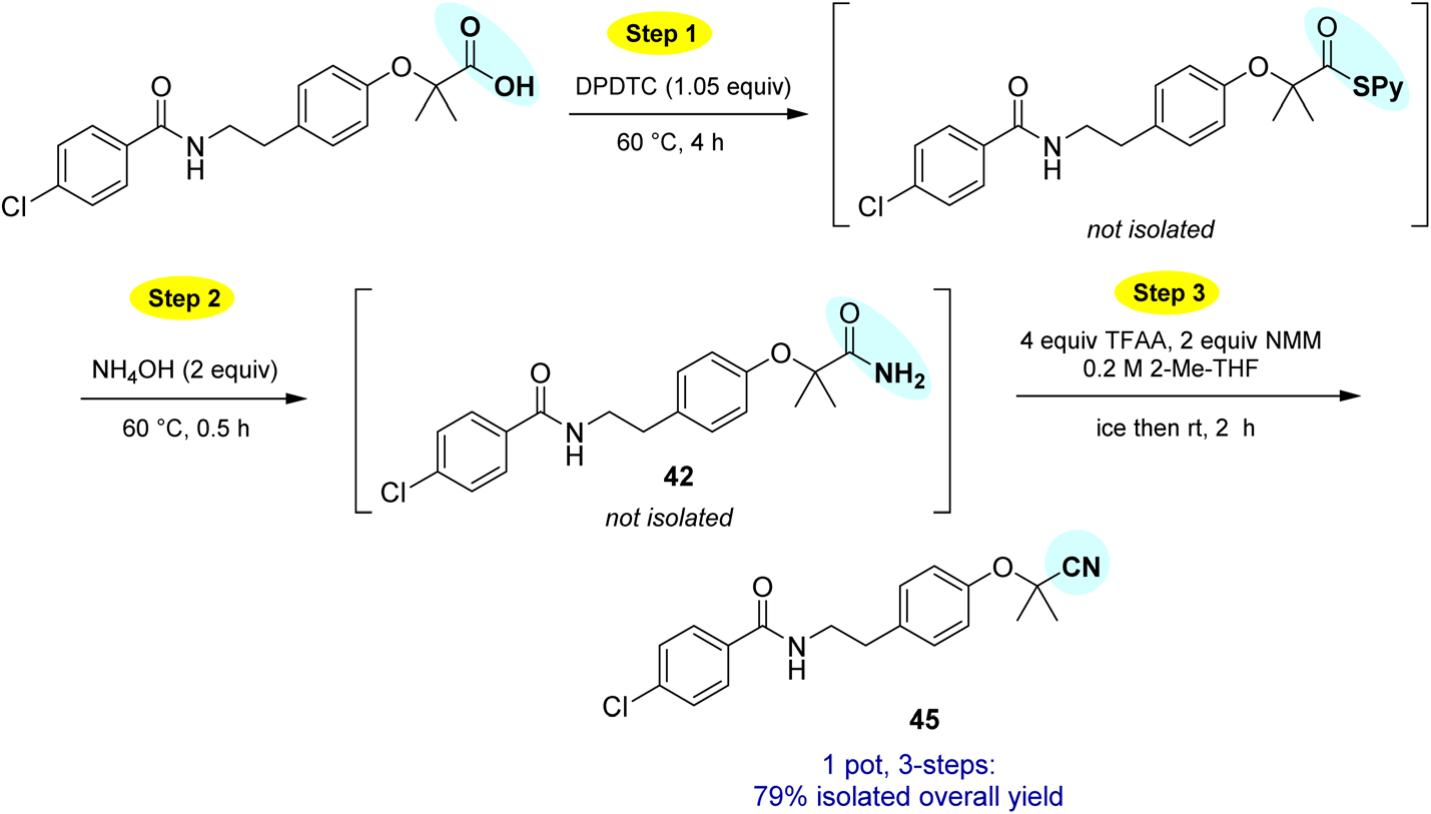
1-Pot conversion of a carboxylic acid to its derived nitrile.

This particular conversion takes on special significance in that the newly formed primary amides are immediate precursors to the corresponding nitrile derivatives *via* dehydration. For example, starting with probenecid (Scheme 8), arylnitrile-containing 4-cyano-*N*,*N*-dipropylbenzenesulfonamide 44 was ultimately formed *via*: (a) thioester generation; (b) treatment with aqueous ammonia, and (c) Pd-catalyzed dehydration using methoxyacetonitrile as the sacrificial, water-absorbing nitrile.^20^ The overall isolated yield for this 1-pot process was 90%. Likewise, the derived *alkyl*nitrile of bezafibrate 45 was formed *via* dehydration of the primary amide 42 with TFAA^21^ in 1-pot (Scheme 9).

**Scheme 10:**
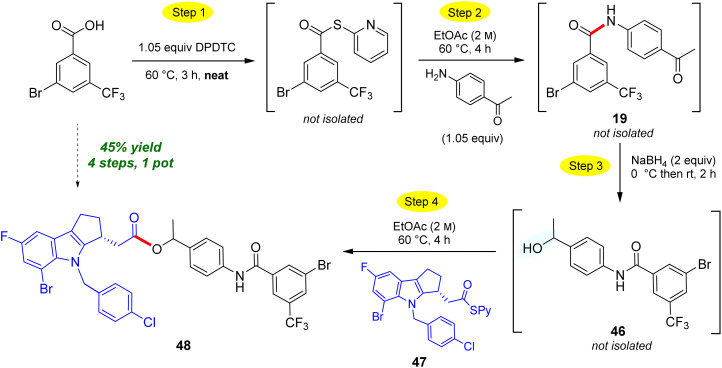
A representative 4-step, 1-pot sequence.

The use of neat conditions to form intermediate thioesters followed by addition of ethyl acetate (2 M), allows for more than initial amide formation, these additional bonds also being made in the same 1-pot fashion (Scheme 10). Thus, from the extensive toolbox of reactions that can now be run under green conditions,^22^ after generating the initial amide bond between 4-aminoacetophenone and 3-bromo-5-(trifluoromethyl)-benzoic acid to afforded *N*-(4-acetylphenyl)-3-bromo-5-(trifluoro-methyl)benzamide 19. Addition of sodium borohydride affords secondary benzylic alcohol 46. Without isolation, introduction of a Merck Informer Library-derived thioester 47 ultimately arrives at highly functionalized ester 48 (45%), a 4-step, 1-pot sequence. The ester bond made in this fashion *via* the 2-pyridyl thioester is representative of the additional technology envisioned (*e.g.*, to make esters) and which is currently under further development. The potential to benefit from both time^23^ and pot economies,^24^ not to mention the savings to be gained in terms of waste creation, should be quite apparent.

In forming intermediate thioesters, DPDTC leads to loss of only CO_2_ and an equivalent of (odorless) 2-pyridinethiol. The mercaptan can be recovered *via* manipulation of the pH of the aqueous medium. Hence, both “by-products” are easily removed relative to handling of most other traditional coupling reagents. Amide formation using this 1-pot sequence also eliminates the need for base in the coupling step, which could be advantageous when epimerizable stereocenters are present.

The calculated Process Mass Intensity (PMI)^25^ associated with formation of 43 was determined on the basis of a 0.25 mmol scale reaction, while the data provided in Table 2 are an average of multigram reactions run by GSK.^26^ With increasing scale it is likely that the PMI derived from the workup for this methodology would decrease. For reactions where the product readily precipitates out, *e.g.*, for some primary amides, the PMI decreases by *ca.* 50% since the product is simply filtered and hence, there is no need for a workup (see last entry in Table 2).

**Table 2:** Comparisons of PMI values en route to 43 with literature values^26^

Reagent	PMI from reaction	PMI from work-up	Overall PMI
Acid chloride	24	11	35
Mixed anhydride	24	19	43
T3P	12	31	43
CDI	16	36	52
HATU	11	23	34
EDC	15	31	46
Oxalyl chloride	12	48	60
**DPDTC** (neat; compound 43)	**0.94**	**14.4**	**15.3**

**Fig. 2:**
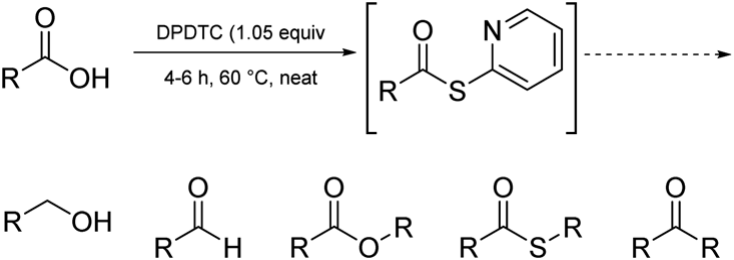
Functional groups realizable *via* the same *in situ*-formed thioesters.

Finally, it is worth noting that these same thioesters are also amenable to conversion to several additional functional groups other than amides/peptides, including, aldehydes, esters, thioesters, and ketones, in addition to esters (see formation of 48 in Scheme 10, and Fig. 2). For example, net conversion of a carboxylic acid to the corresponding aldehyde without resorting to DIBAL reductions of initially prepared esters or Weinreb amides, or alternative reductions that require initial acid halide formation, are processes now in hand. Likewise, reductions of acids directly to alcohols, commonly described within textbooks as under the domain of LAH, can be done safely in 1-pot, in water. Details for these new processes will be reported shortly.

## Conclusions

A safe, effective, and general 1-pot procedure to arrive at highly desired amides has been developed that follows Nature's lead: using a readily formed thioester intermediate, followed by its conversion in 1-pot to the desired product. Applications to several important and highly functionalized amide/peptide-containing targets have been documented herein. The key aspects to this contribution can be summarized as follows:

• Traditional peptide coupling reagents are no longer needed.

• Relies on initial thioester formation involving an easily made, odorless reagent DPDTC (dipyridyl dithiocarbonate).

• Three approaches have been developed, each leading to the same end, are less waste-generating and hence, are far “greener” than are existing technologies.

• Where present, stereocenters are maintained.

• Several sequences are illustrated highlighting options for multi-step processes in a single pot, illustrative of both time and pot economies.

• A novel process that converts, in 1-pot, an acid to a nitrile *via* the *in situ*-formed primary amides has been disclosed, applicable to both aryl- and alkyl-substituted carboxylic acids.

## Data availability

The synthetic procedures, characterization, and spectral data supporting this article have been uploaded as part of the ESI.†

## Author contributions

All authors have given approval to the final version of the manuscript. K. M. F. and R. D. K. contributed equally. R. M. T. contributed to the 1-pot, four step sequence. D. M. R. contributed to initial optimization. P. D. contributed to optimization of the formation of the thioester. M. S. contributed to purification of substrates, and 1-pot sequences. B. H. L. oversaw work and aided in drafting the manuscript.

## Conflicts of interest

There are no conflicts to declare.

## Supplementary Material

SC-014-D3SC00198A-s001
