# Supplementary material for: Direct formation of amide/peptide bonds from carboxylic acids: no traditional coupling reagents, 1-pot, and green
Source: Chem Sci. 2023 Feb 28;14(13):3462–9. doi: 10.1039/d3sc00198a (PMC10055766; doi:10.1039/d3sc00198a)
Supplement: SC-014-D3SC00198A-s001 [file SC-014-D3SC00198A-s001.pdf]

Electronic Supplementary Material (ESI) for Chemical Science.
This journal is © The Royal Society of Chemistry 2023

Supporting Information

Direct formation of amide/peptide bonds from carboxylic acids: no traditional coupling reagents, 1-pot, and green

Kaitlyn M. Freiberg, Rahul D. Kavthe, Rohan M. Thomas, David M. Fialho, Paris Dee, Matthew Scurria, Bruce H. Lipshutz*

Department of Chemistry and Biochemistry, University of California, Santa Barbara, CA 93106 USA

Phone: 805-893-2521
Fax: 805-893-8265
Email: lipshutz@chem.ucsb.edu
Website: https://lipshutz.chem.ucsb.edu/

Contents

1. General information S2
2. Synthesis of di-2-pyridyldithiocarbonate (DPDTC) S2
3. Optimization of the reaction conditions S3
   3.1. Optimization of S-(2-pyridyl) thioester formation S3
   3.2. Optimization of amide bond formation from S-(2-pyridyl) thioesters S5
   3.3. NMR Yield Calculations S10
4. One pot amide bond formation from carboxylic acid S14
5. General Purification Procedures for amides S15
   5.1. General Purification Method for General Procedure A S15
   5.2. General Purification Method for General Procedure B S15
   5.3. General Purification Method for General Procedure C S15
6. Synthesis of starting materials S16
7. PMI and E Factor Calculations S17
8. Large Scale Reaction S19
9. Recycle Study S20
10. Multi-step, 1-pot: chemocatalysis sequence procedures S21
11. Analytical data S24
12. References S50
13. NMR Spectra S52

1. General information

A solution of 2 wt % surfactant/H_2O was prepared by dissolving the surfactant in degassed HPLC grade water and was stored under argon. TPGS-750-M was obtained from a contract manufacturer, but is also available from Sigma-Aldrich (catalog #733857). All commercially available reagents were purchased from Sigma-Aldrich, Combi-Blocks, Ambeed Inc., Acros Organics, BLD Pharma, Fischer Scientific, or ChemScene and used without further purification. Thin layer chromatography (TLC) was done using Silica Gel 60 F254 plates (Merck, 0.25 mm thick). The developed TLC plate was analyzed by a UV lamp (254 nm). The plates were further analyzed with the use of an aqueous ceric ammonium molybdate stain or ethanolic vanillin and developed with a heat gun. All commercially available reagents were used without further purification. Flash chromatography was performed using Silicycle Silicaflash® P60 unbonded grade silica.

^{1}H, ^{13}C, and ^{19}F NMR were recorded at 25 ºC on either an Agilent Technologies 400 MHz, a Bruker Avance III HD 400 MHz, or a Agilent Technologies 500 MHz, a Bruker Avance III HD 400 MHz spectrometer in $CDCl_3$, DMSO-*d*$_6$, or benzene-*d*$_6$ with residual $CHCl_3$ (^{1}H = 7.26 ppm, ^{13}C = 77.16 ppm), DMSO (^{1}H = 2.54 ppm, ^{13}C = 40.45 ppm), and benzene (^{1}H = 7.16 ppm, ^{13}C = 128.06 ppm) as the internal standard. Chemical shifts are reported in parts per million (ppm, or Hz). The data presented will be reported as follows; chemical shift, multiplicity (s = singlet, bs = broad singlet, d = doublet, dd = doublet of doublet, t = triplet, q = quartet, quin = quintet, m = multiplet), coupling constant (if applicable), and integration. High-resolution mass analyses (HRMS) were recorded on Waters GCT Premier GC TOF or Agilent 6230 TOF LC/MS System.

2. Synthesis of di-2-pyridyldithiocarbonate (DPDTC)

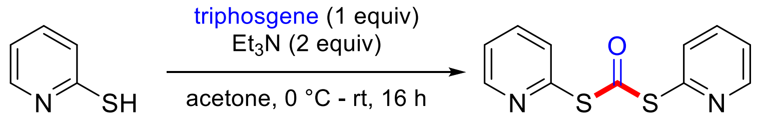

2-mercaptopyridine

1
DPDTC
89% yield, 6.5 g scale

All glassware was flame dried. To a 500 ml round-bottom flask equipped with a PTFE-coated magnetic stir bar was added 2- mercaptopyridine (6 equiv, 60 mmol, 6.67 g), then the flask was sealed with a rubber septum and anhydrous acetone (100 ml) was added via syringe under a positive flow of argon, followed by anhydrous Et_3N (6 equiv, 60 mmol, 8.36 mL) and the solution was stirred until all components were fully dissolved. An ice bath was used to cool the resulting solution, an argon balloon was affixed to the septum via a needle, then a solution of triphosgene (1 equiv, 10 mmol, 2.967 g) in acetone (12.5 mL) was slowly added over the course of 15 min. The ice was replaced as needed to keep the solution cool during addition of triphosgene to prevent excessive generation of phosgene gas. Upon full addition, triethylammonium chloride was observed to precipitate. The reaction was allowed to warm to rt and stir overnight. Upon completion, the septum was removed inside a fume hood and allowed to expel any excess phosgene

gas, then the reaction mixture was filtered to remove triethylammonium chloride, and the filtrate was concentrated in vacuo to afford a crude oil containing crystals of remaining triethylammonium chloride. The crude residue was redissolved in EtOAc in 10 ml portions and filtered into a separatory funnel. The combined organic extracts were washed with saturated aqueous $NaHCO_3$ (100 mL), followed by DI water (100 mL), followed by saturated brine (100 mL). The organic layer was separated and dried over anhydrous Na_2SO_4, filtered, and concentrated *in vacuo* and residual solvent was removed under high vacuum overnight to afford DPDTC as a light-yellow solid (6.596 g, 89% yield).

Caution: Triphosgene is acutely toxic and releases toxic phosgene gas on contact with moisture. It should be handled on small scale in a fume hood or glove box and weighed out using a pre-weighed, tightly sealed container.

Note: Commercial sources of inexpensive 2-mercaptopyridine may require purification prior to use due to the presence of the derived disulfide. This can be accomplished via recrystallization from EtOAc.

3. Optimization of the reaction conditions

3.1. Optimization of S-(2-pyridyl) thioester formation

General Procedure for thioester formation under neat conditions: To a 1-dram vial, a PTFE-coated stir bar, carboxylic acid (1 equiv, 0.125 mmol, 36.8 mg) and DPDTC (1.05 equiv, 0.1275 mmol, 31.7 mg) were added. Upon completion, 1,3,5- trimethoxzybenzene and $CDCl_3$ was added to the reaction vial and stirred for 15 minutes until fully homogeneous. The NMR was then directly taken. The contents were stirred at various temperatures and times. For all of the following optimization reactions, ^{1}H NMR yields were calculated using 1,3,5-trimethoxybenzene as the internal standard, integrating for the product at 7.88 ppm as described in section 3.3.

Table S1. Screening of equivalents of DPDTC

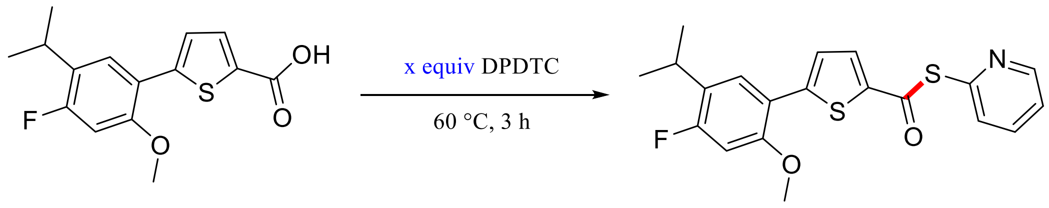

entry	equivalents DPDTC	NMR yield (%)[a]
**1**	1	83
**2**	1.05	88
**3**	1.10	93
**4**	**1.25**	**98**
**5**	1.50	94

[a] Yields were determined by ^{1}H NMR using 1,3,5-trimethylbenzene as an internal standard.

Table S2. Screening of temperature and time

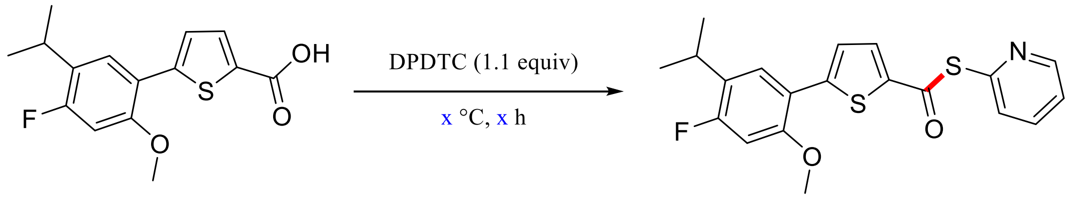

entry	temperature (°C)	time (h)	NMR yield (%)[a]
**1**	40	1	9
**2**	40	2	37
**3**	40	3	55
**4**	40	4	72
**5**	50	1	42
**6**	50	2	71
**7**	50	3	70
**8**	50	4	84
**9**	60	1	71
**10**	60	2	88
**11**	60	3	93
**12**	**60**	**4**	**98**

[a] Yields were determined by ^{1}H NMR using 1,3,5-trimethylbenzene as an internal standard.

Figure S1. Effect of time and temperature on thioester formation

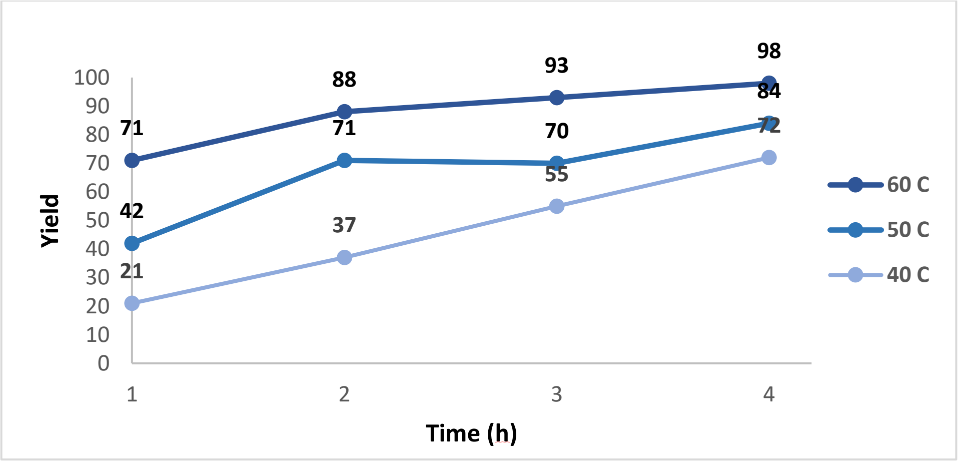

Table S3. Screening of additives

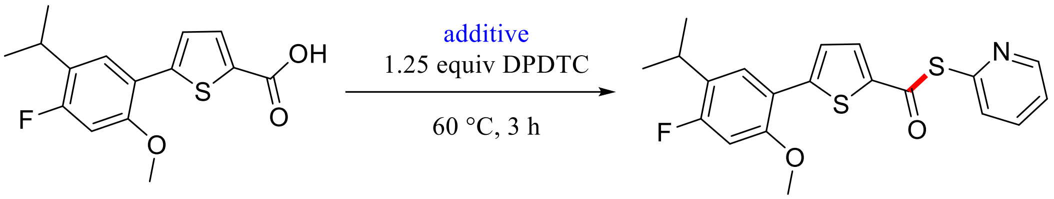

entry	additive	NMR yield (%)[a]
**1**	**DMAP**	**99**
2	$NaHCO_3$	92
3	DIPEA	70
4	TEA	65

[a] Yields were determined by ^{1}H NMR using 1,3,5-trimethylbenzene as an internal standard.

3.2. Optimization of amide bond formation from S-(2-pyridyl) thioesters

General Procedure for amide bond formation from S-(2-pyridyl) thioesters: To a 1-dram vial, a PTFE-coated stir bar, pyridyl thioester (1 equiv, 0.10 mmol, 38.7 mg) and 3,4,5-trimethoxyaniline (1 equiv, 0.11 mmol, 20.2 mg) was added. Unless otherwise state 1.1 equiv of trimethoxyaniline was used. The contents were stirred at various temperatures, times, and in various solvents, or under aqueous conditions. Solvent or 2 wt % TPGS-750-M / H_2O was subsequently added when noted. Upon completion 1,3,5-trimthoxybenzene and $CDCl_3$ was added to the reaction vial and stirred for 15 minutes until fully homogeneous. For aqueous systems, the reaction was extracted with EtOAc until no product remained in the aqueous layer. The combined organics were dried with anhydrous $MgSO_4$ and concentrated via rotary evaporator. For screening reactions with organic solvents, the reaction was concentrated via rotary evaporator. 1,3,5-Trimethoxybenzene and $CDCl_3$ were then added and stirred for 15 minutes until fully homogeneous. The NMR was then directly taken.

For all reactions, ^{1}H NMR yields were calculated using 1,3,5-trimethoxybenzene as the internal standard, integrating for the product at 7.65 ppm as described in section 3.3. To each of the resulting reaction mixtures 1,3,5-trimethoxybenzene was directly added. The yield was determined by ^{1}H NMR relative to the amount of internal standard added.

Table 4. Screening of solvent for amide bond formation under highly concentrated mixtures

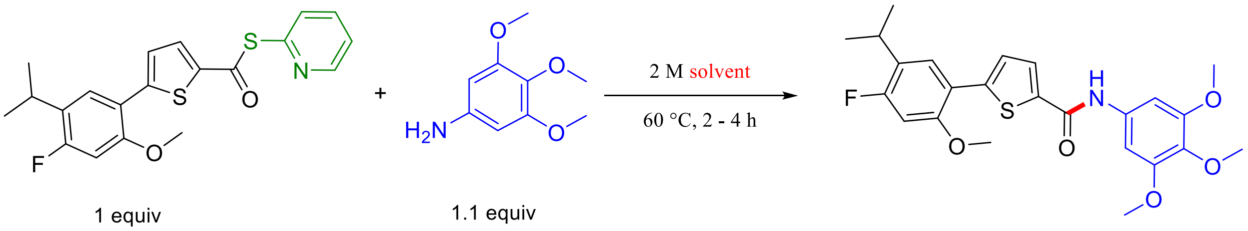

entry	solvent (2 M, 50 μL)	NMR yield (%)[b] (2 h)	NMR yield (%)[b] (4 h)
1	mineral oil	44	74
2	mineral oil	-	66
**3**	**EtOAc**	**84**	**97**
4	2-Me-THF	75	98
5	acetone	80	98
6	isopropyl acetate	83	99
7	EtOAc	84	-
8	TEA	86	-
9	TEA (5 equiv)	92	-

[a]*100 μL of mineral oil used.* [b]Yields were determined by ^{1}H NMR using 1,3,5-trimethylbenzene as an internal standard.

Table S5. Screening of equivalents of amine

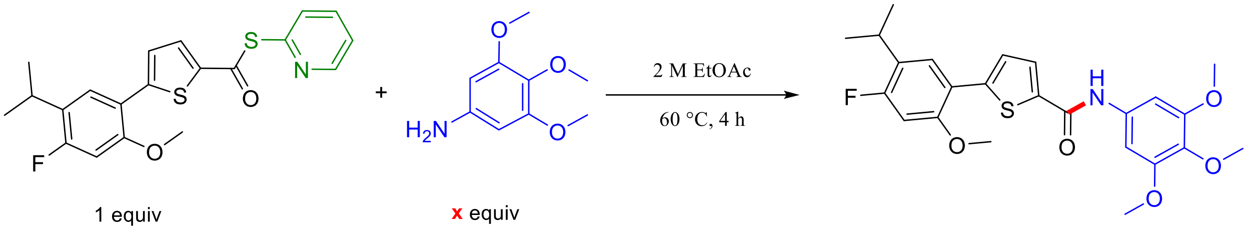

entry	amine (equiv)	NMR yield (%)[a]
1	1	92
**2**	**1.05**	**94**
3	1.1	90
4	1.25	89

[a] Yields were determined by ^{1}H NMR using 1,3,5-trimethylbenzene as an internal standard.

Table S6. Screening of temperature for amide bond formation under highly concentrated mixtures

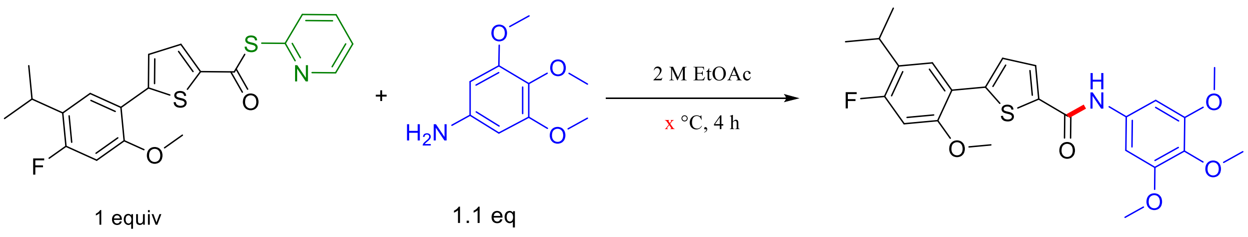

entry	temperature (°C)	NMR yield (%)[a]
1	40	84
2	50	95
**3**	**60**	**97**

[a] Yields were determined by ^{1}H NMR using 1,3,5-trimethylbenzene as an internal standard.

Table S7. Screening of additive under neat conditions

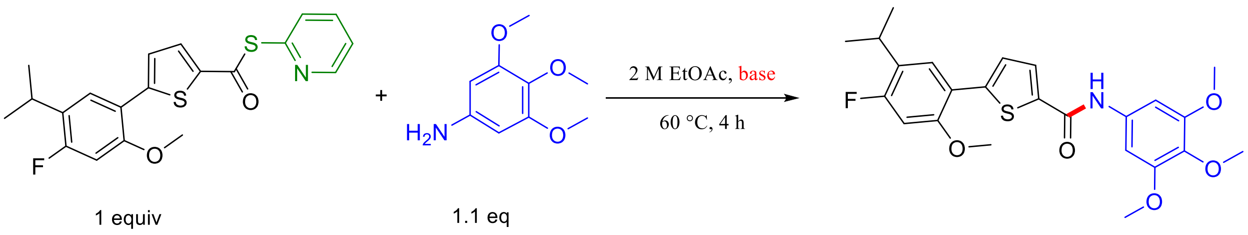

entry	base (5 equiv)	NMR yield (%)[a]
1	NMM	87
2	DIPEA	94
**3**	**TEA**	**99**
4	*s*-collidine	90
5	2,6-lutidine	80

[a] Yields were determined by ^{1}H NMR using 1,3,5-trimethylbenzene as an internal standard.

Table S8. Screening of temperature and time for reactions run in 2 wt % TPGS-750-M/H_2O

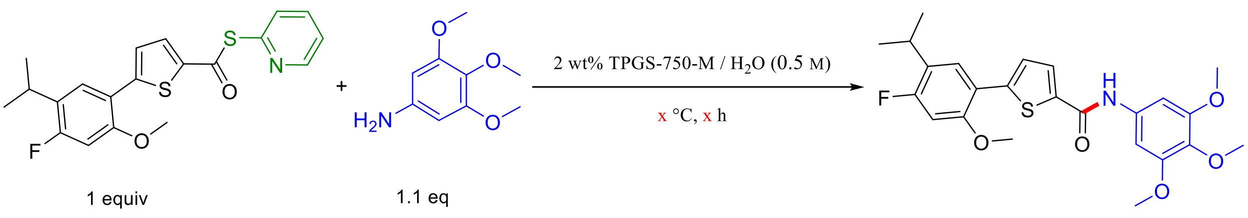

entry	temperature	time (h)	NMR yield (%)[a]
1	rt	2	7
2	rt	16	3

3	40 °C	2	7
4	40 °C	2	5
5	40 °C	6	12
6	40 °C	8	10
7	40 °C	16	95
8	50 °C	2	31
9	50 °C	16	90
10	60 °C	2	89
11	60 °C	6	95
12	60 °C	8	89
**13**	**60 °C**	**16**	**>95**

[a] Yields were determined by ^1H NMR using 1,3,5-trimethylbenzene as an internal standard.

Figure S2. Effect of time and temperature of amide formation under aqueous micellar conditions

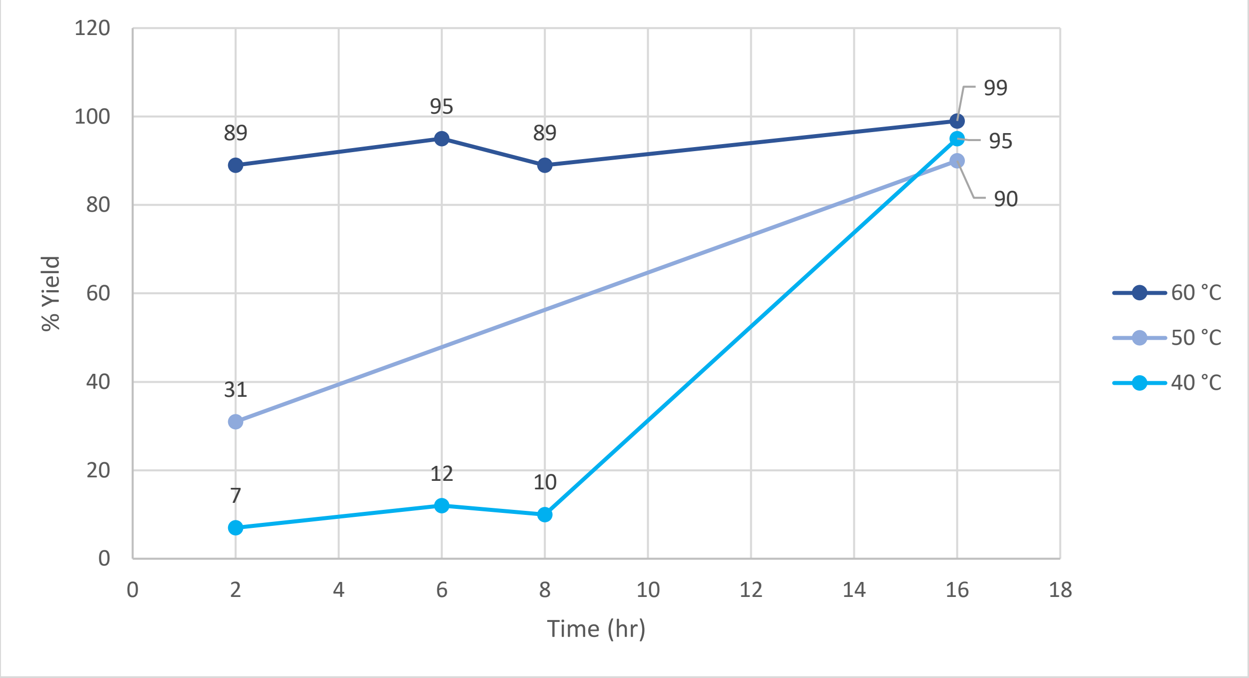

Table S9. Screening of surfactant

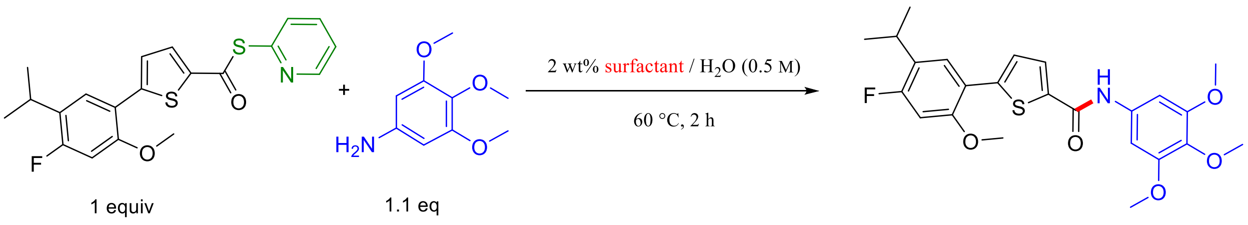

entry	co-solvent (10 vol %)	NMR yield (%)[a]
**1**	**TPGS-750-M**	**89**
2	MC-1	52
3	Triton-X 100	70

4	SDS	54
5	on water[a]	61

[a] Reaction time was 4 h. [b] Yields were determined by ^{1}H NMR using 1,3,5-trimethylbenzene as an internal standard.

Table S10. Screening of co-solvent in 2 wt % TPGS-750-M/H_2O

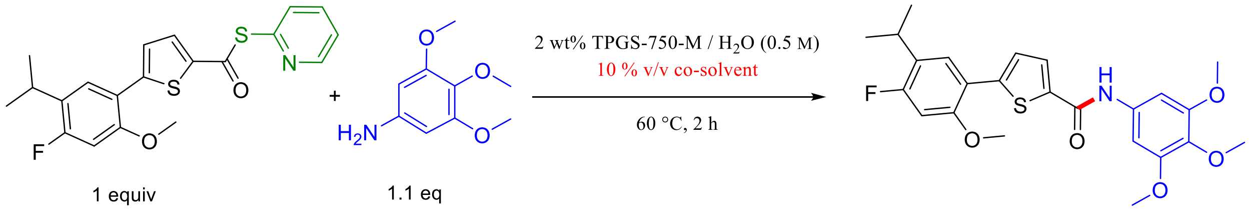

entry	co-solvent (10 vol %)	NMR yield (%)[a]
1	EtOAc	98
2	acetone	70
3	2-Me-THF	88
4	isopropyl acetate	91

[a] Yields were determined by ^{1}H NMR using 1,3,5-trimethylbenzene as an internal standard.

3.3. NMR Yield Calculations

All NMR yields were calculated as follows using 1,3,5-trimethoxybenzene as the internal standard.

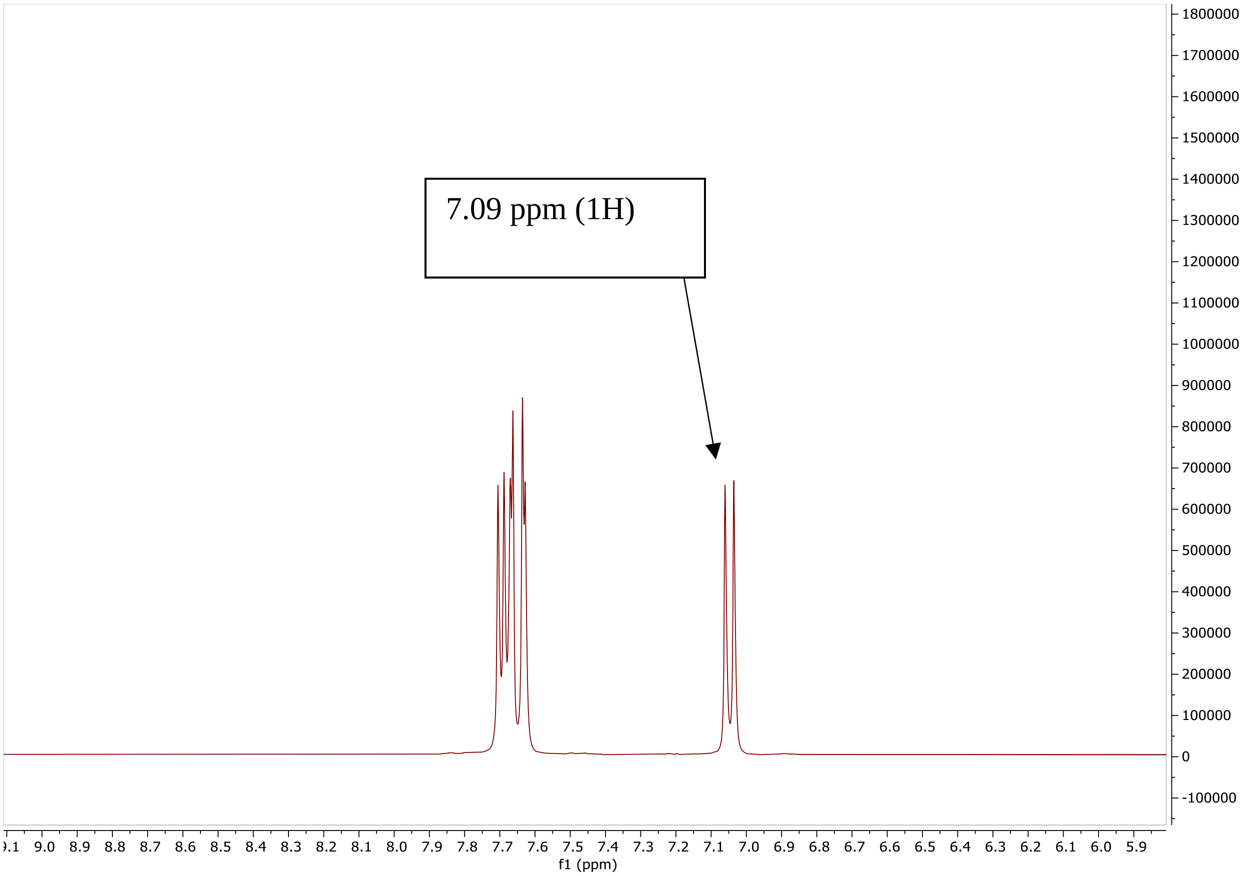

Figure S1: ^{1}H NMR spectrum of 5-(4-fluoro-5-isopropyl-2-methoxyphenyl)thiophene-2-carboxylic acid in DMSO-d_6.

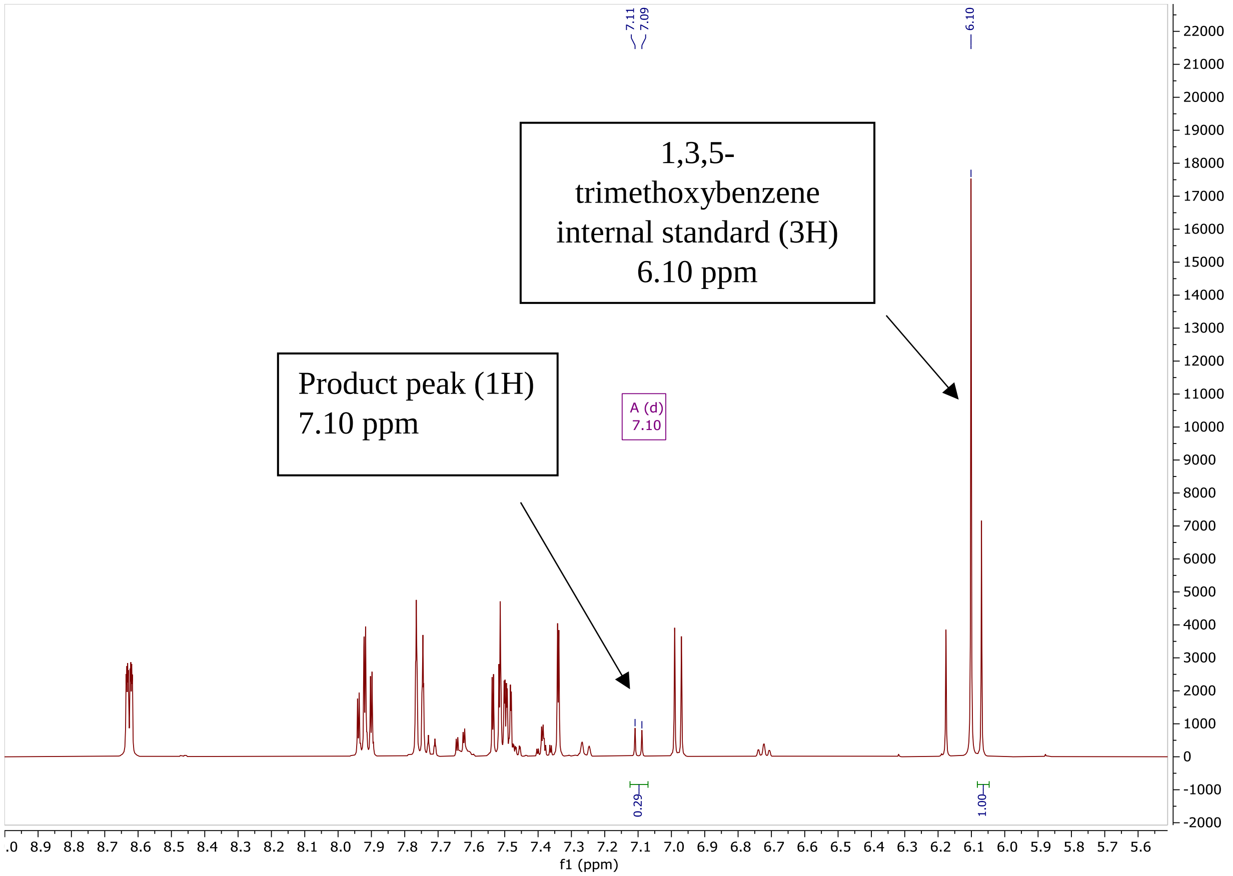

Figure S3: Example of ^{1}H NMR yield of pyridyl thioester from carboxylic acid in DMSO-d_6.

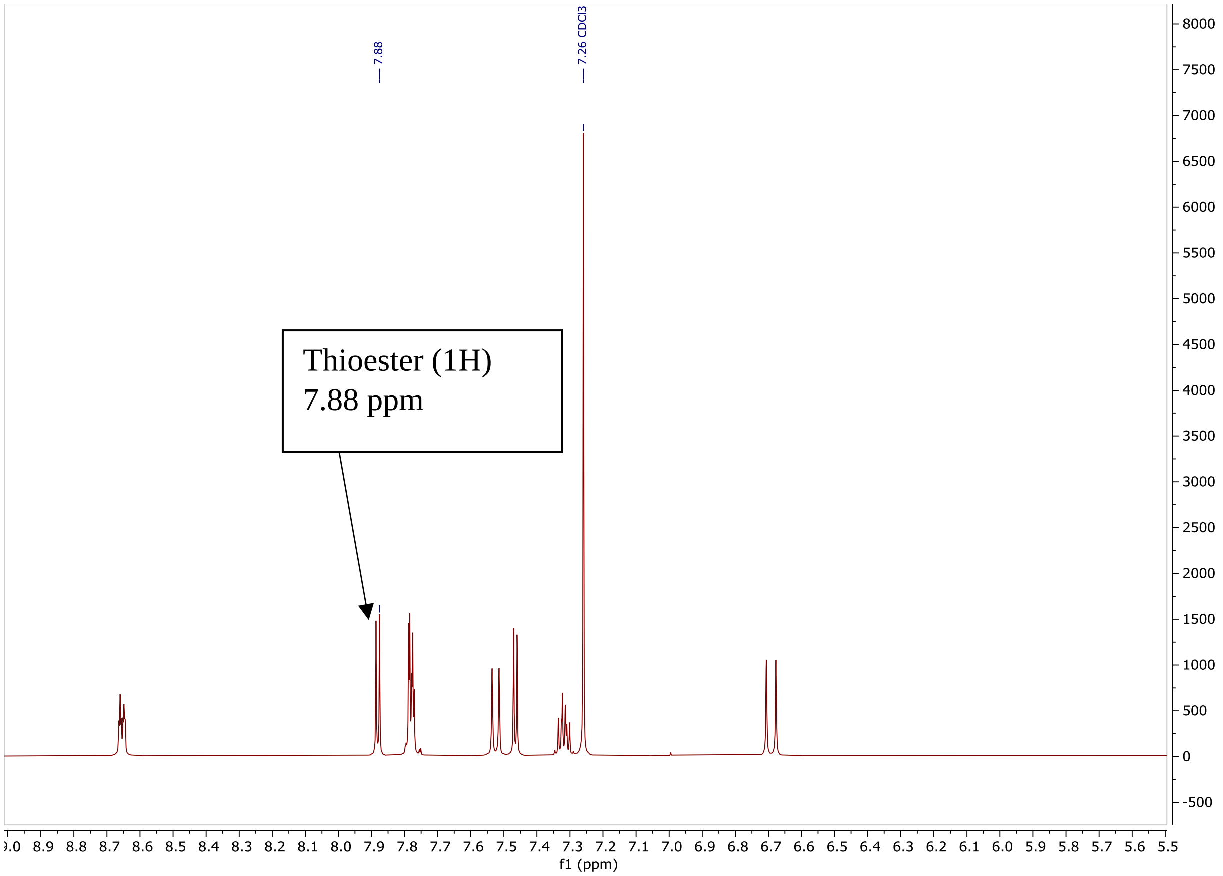

Figure S4: ^{1}H NMR spectrum of S-(pyridin-2-yl) 5-(4-fluoro-5-isopropyl-2-methoxyphenyl)-thiophene-2-carbothioate in $CDCl_3$

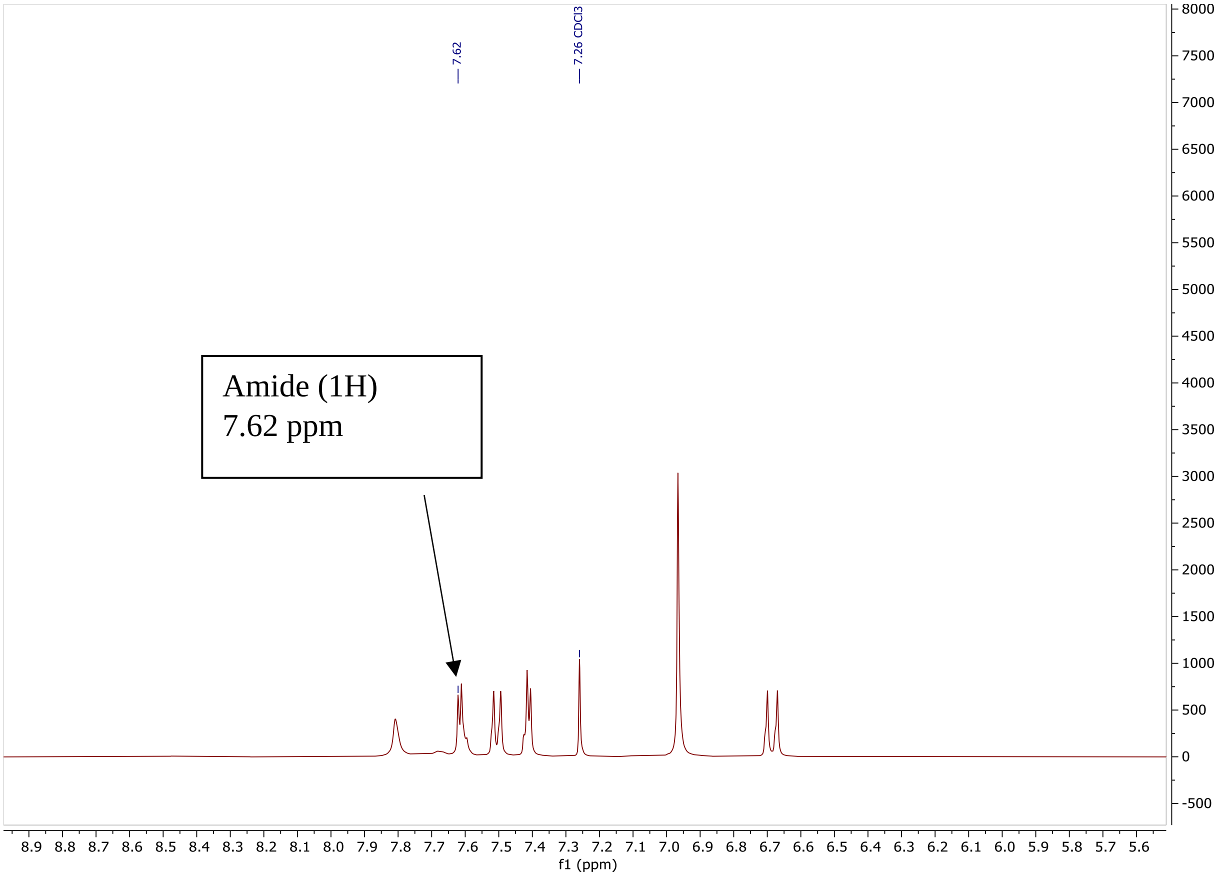

Figure S5: ^{1}HNMR spectrum of 5-(4-fluoro-5-isopropyl-2-methoxyphenyl)-*N*-(3,4,5-trimethoxyphenyl)thiophene-2-carboxamide in $CDCl_3$

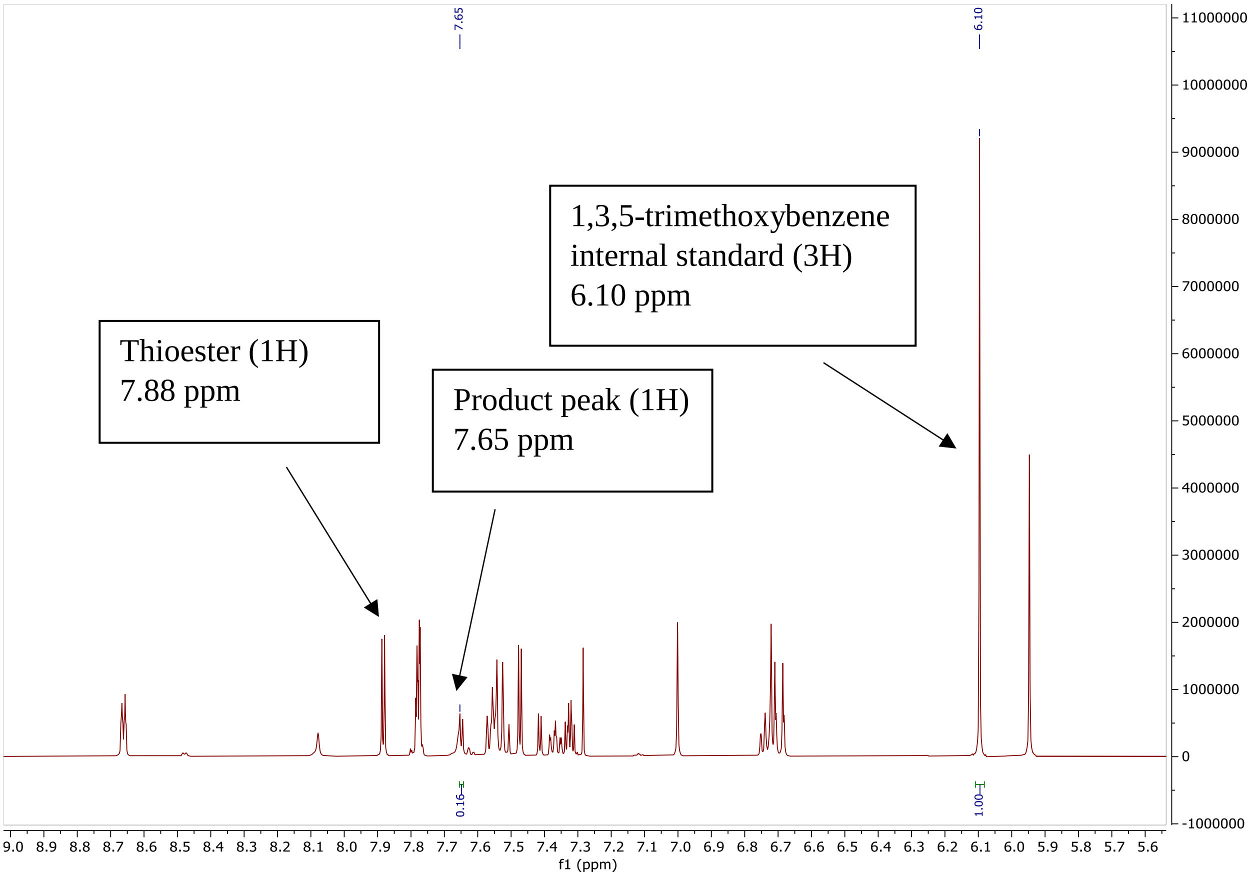

Figure S6: Example of ^{1}H NMR yield of amide from 2-pyridylthioester in $CDCl_3$

4. One pot amide bond formation from carboxylic acid

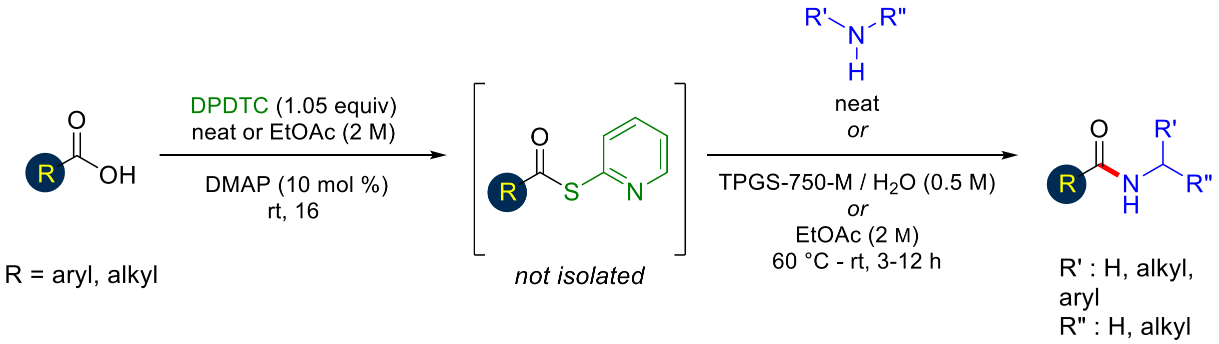

General procedure of amide bond formation under neat conditions (Method A): To a 1-dram vial, a PTFE-coated stir bar, carboxylic acid (1 equiv, 0.250 mmol) and DPDTC (1.05 equiv, 0.263 mmol, 65.2 mg) was added. The contented were stirred at 60 °C until full consumption of the acid, as determined by TLC (~4-6 h). Because the reaction is neat, it is important for the completion of the reaction for all contents to not be on the sides of the reaction vessel. In addition, the stirring

should not be too vigorous to cause splashing up the sides of the vial. Upon complete consumption of the thioester the amine was directly added, in 1-pot to the vial and stirred until complete consumption of the thioester. The crude reaction mixture was dissolved in EtOAc and washed with 1 M NaOH (1 mL x 3) and concentrated via rotary evaporation.

General procedure of amide bond formation by using EtOAc (2 M) (Method B):

Upon complete consumption of the thioester, amine (1.05 equiv, 0.263 mmol), followed by EtOAc (2 M, 125 µL) was directly added, and stirred at 60 °C until complete consumption of the thioester. The crude reaction mixture was dissolved in EtOAc and washed with 1 M NaOH (1 mL x 3), then 1 M HCl (1 mL x 3), the EtOAc was then concentrated via rotary evaporation.

General procedure for amide bond formation by using aqueous conditions (Method C):

Upon complete consumption of the thioester, amine (1.05 equiv, 0.263 mmol), EtOAc (10 vol %, 50 µL), and 2 wt % TPGS-750-M/H_2O (0.5 M, 0.5 mL), respectively, was directly added, and stirred at 60 °C until complete consumption of the thioester. The crude reaction mixture was washed with 1 M NaOH (1 mL x 3) and dried over anhydrous $MgSO_4$.

5. General Purification Procedures for amides

5.1. General Purification Method for General Procedure A

Method A-1
The crude reaction mixture was dissolved in EtOAc and washed with 1 M NaOH (1 mL x 3) and concentrated via rotary evaporation.

Method A-2
The crude reaction mixture was dissolved in EtOAc and washed with 1 M NaOH (1 mL x 3), then 1 M HCl (1 mL x 3), and dried over anhydrous $MgSO_4$. The combined EtOAc layers were then concentrated via rotary evaporation.

Method A-3
The crude reaction mixture was washed with 1 M NaOH (1 mL x 3) and dried over anhydrous $MgSO_4$ and concentrated via rotary evaporation to afford the crude product.

5.2. General Purification Method for General Procedure B

The crude reaction mixture was extracted with EtOAc until no product could be observed in the EtOAc, as determined by TLC. The extracts were then concentrated and purified via silica gel chromatography. The eluent varied per substrate.

5.3. General Purification Method for General Procedure C

The crude reaction mixture was extracted with EtOAc until no product remained as determined by TLC. The extracted layers were combined and then washed with 1 M NaOH (3 times) followed by

1 M HCl (3 times). The crude material was then obtained via rotary evaporation followed by purification via silica gel chromatography. The eluent varied per substrate.

6. Synthesis of starting materials

Synthesis of 5-(4-fluoro-5-isopropyl-2-methoxyphenyl)thiophene-2-carboxylic acid

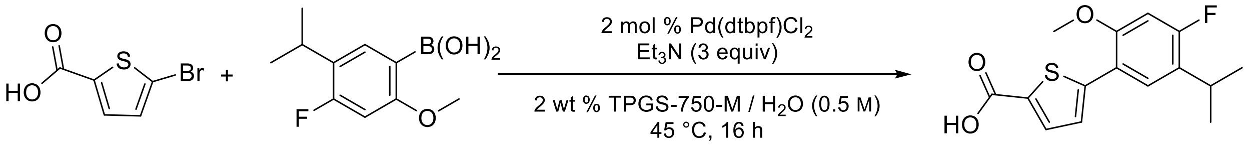

To an oven dried round bottom flask with a PTFE coated stir bar were added 5-bromo-2-furoic acid (1 equiv, 7.4 mmol, 1.55 g, (4-fluoro-5-isopropyl-2-methoxyphenyl)boronic acid (1.2 equiv, 8.88 mmol, 1.905 g), and Pd(dtbpf)Cl_2 (1 mole %, 97.6 mg). The round bottom flask was then capped with a rubber septum and purged with argon. Triethylamine (3 equiv, 22.2 mmol, 2.27 mL) and 2 wt % TPGS-750-M/H_2O (0.5 M, 12.7 mL) was then added via syringe and stirred vigorously overnight (16 h) at 45 °C (internal temperature). The reaction was then quenched by the addition of concentrated HCl. The precipitate was filtered and redissolved in EtOAc and then washed with 50% sodium bicarbonate/water solution. The EtOAc layer was either reconcentrated *in vacuo* or precipitated with concentrated HCl. The resulting compound was an off-white/light brown solid (1.85 g, 85% yield). R_f = 0.50 (1:1 EtOAc/hexanes).

Synthesis of 8-bromo-5-methyl-6-oxo-5,6-dihydro-4H-benzo[f]imidazo[1,5-a][1,4]diazepine-3-carboxylic acid

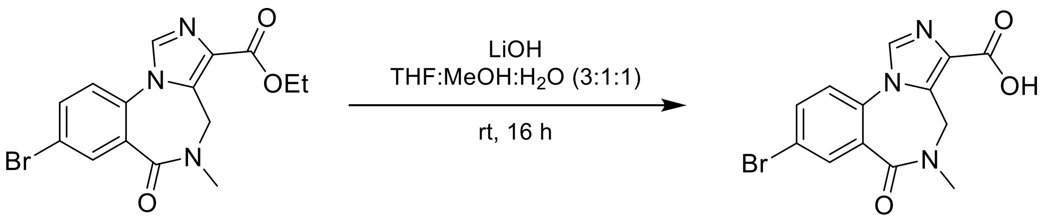

To an oven dried round bottom flask with a PTFE coated stir bar were added ethyl 8-bromo-5-methyl-6-oxo-5,6-dihydro-4H-benzo[f]imidazo[1,5-a][1,4]diazepine-3-carboxylate (300 mg, 0.83 mmol), THF (6 mL), MeOH (2 mL) and a solution of lithium hydroxide (59.3 mg, 0.24 mmol) in water (2 mL) was added. The mixture was stirred at rt for 16 h. The volatiles were removed *in vacuo* and the residue treated with 10% aqueous citric acid solution. The resultant precipitate was filtered and dried under suction to give the title compound 8-bromo-5-methyl-6-oxo-5,6-dihydro-4H-benzo[f]imidazo[1,5-a][1,4]diazepine-3-carboxylic acid as a white solid (251 mg, 90% yield).

Synthesis of 3-(3-(tert-butylthio)-1-(4-chlorobenzyl)-5-(quinolin-2-ylmethoxy)-1H-indol-2-yl)-2,2-dimethylpropanoic acid

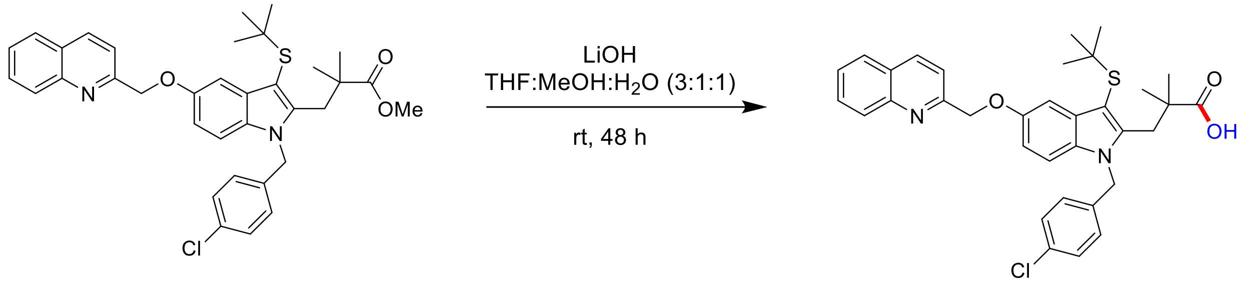

To an oven dried round bottom flask with a PTFE coated stir bar were added methyl 3-(3-(*t*-butylthio)-1-(4-chlorobenzyl)-5-(quinolin-2-ylmethoxy)-1H-indol-2-yl)-2,2-dimethylpropanoate (200 mg, 0.33 mmol), THF (6 mL), MeOH (2 mL), and a solution of lithium hydroxide (23.76 mg, 0.1 mmol) in water (2 mL) added. The mixture was stirred at rt for 48 h. The volatiles were removed *in vacuo* and the residue treated with 10% aqueous citric acid solution. The resultant precipitate was filtered and dried under suction to give the title compound 8-bromo-5-methyl-6-oxo-5,6-dihydro-4H-benzo[f]imidazo[1,5-a][1,4]diazepine-3-carboxylic acid as a yellow solid (140 mg, 72% yield).

7. PMI and E Factor Calculations

1. PMI for neat reaction (primary amide formation) on 0.25 mmol scale

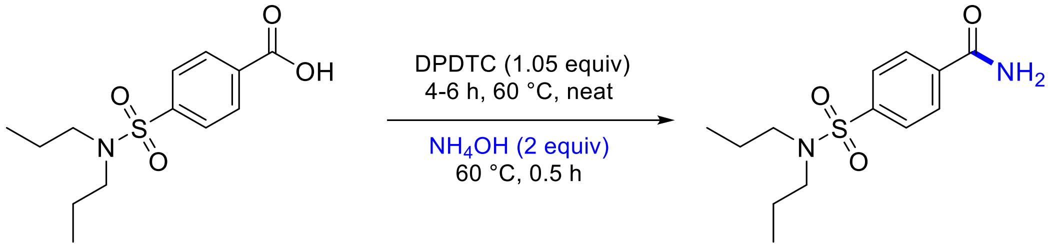

$$\textbf{E Factor} = \frac{mass\ waste\ organics}{mass\ product} = \frac{(mass_{\ DPDTC} + mass_{excess\ amine})}{(massproduct)}\ (massproduct) = \frac{0.0652\ g + 0.00426g}{0.0710} = 0.94$$

$$\textbf{PMI} = \frac{mass\ waste\ organics}{mass\ product} = \frac{(mass_{\ DPDTC} + mass_{excess\ amine} + mass_{aq})}{(massproduct)}\ (massproduct) = \frac{0.0652\ g\ +\ 0.00426g + 1.02g}{0.0710} = 15.3$$

2. PMI for neat reaction forming a secondary amide on a 0.25 mmol scale

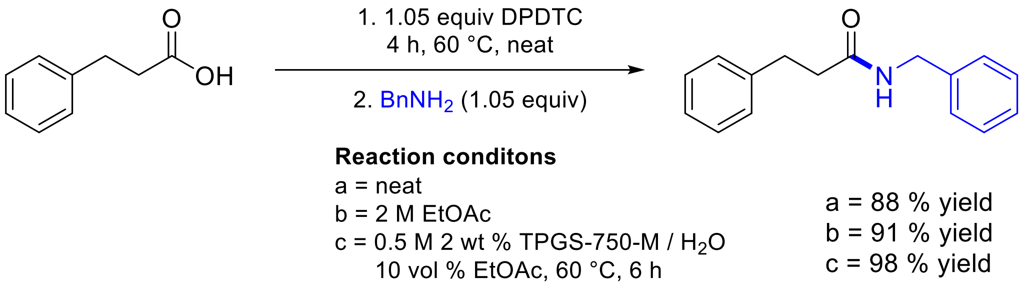

PMI and E Factor for a neat reaction (conditions a)

$$E\ Factor\ = \frac{mass\ waste\ organics}{mass\ product} = \frac{(mass_{\ DPDTC} + mass_{excess\ amine} + mass_{EtOAc})}{(massproduct)}\ (massproduct) = \frac{0.0652\ g + 0.00138g\ + 0.451g}{0.0527} = 9.77$$

$$PMI\ = \frac{mass\ waste\ organics}{mass\ product} = \frac{(mass_{\ DPDTC} + mass_{excess\ amine} + mass_{aq})}{(massproduct)}\ (massproduct) = \frac{0.0652\ g + 0.00138g\ + 0.451g + 1.02g}{0.0527} = 29.1$$

PMI and E Factor for a reaction using 2 M EtOAc (conditions b)

$$E\ Factor\ = \frac{mass\ waste\ organics}{mass\ product} = \frac{(mass_{\ DPDTC} + mass_{excess\ amine} + mass_{EtOAc})}{(massproduct)}\ (massproduct) = \frac{0.0652\ g + 0.00138g\ + 0.634g}{0.0542} = 12.9$$

$$PMI\ = \frac{mass\ waste\ organics}{mass\ product} = \frac{(mass_{\ DPDTC}\ + \ mass_{excess\ amine}\ + \ mass_{aq})}{(massproduct)}\ (massproduct) = \frac{0.0652\ g\ + \ 0.00138g\ \ + 0.634g + 1.02g}{0.0542} = 31.7$$

PMI and E Factor for 0.5 M 2 wt % TPGS-750-M/H2O, 10 vol % EtOAc reaction on 1 mmol scale (conditions c)

$$E\ Factor\ = \frac{mass\ waste\ organics}{mass\ product} = \frac{(mass_{\ DPDTC} + mass_{excess\ amine} + mass_{EtOAc})}{(massproduct)}\ (massproduct) = \frac{0.012416g + 0.0053575g\ + 2.233g}{0.234} = 23.2$$

$$PMI\ = \frac{mass\ waste\ organics}{mass\ product} = \frac{(mass_{\ DPDTC}\ + \ mass_{excess\ amine}\ + \ mass_{aq})}{(massproduct)}\ (massproduct) = \frac{0.0652\ g\ + \ 0.00138g\ \ + 0.634g + 5.06g}{0.234} = 31.3$$

Table 10: Comparison PMI

entry	reagent	reaction	work-up	overall PMI
**1**	T3P	12	31	43
**2**	CDI	16	36	52
**3**	HATU	11	23	34
**4**	EDC	15	31	46
**5**	oxalyl chloride	12	48	60
**6**	**DPDTC**	**1.2**	**27.9**	**29.1**
**7**	**DPDTC (1° amide)**	**0.94**	**14.4**	**15.3**

8. Large Scale Reaction

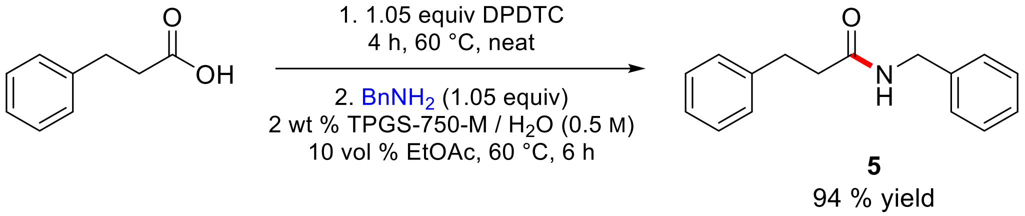

5
94 % yield

A 100 mL round-bottom flask containing a PTFE-coated magnetic stir bar was charged with phenylpropionic acid (1.501 g, 10 mmol) and DPDTC (2.607 g, 10.5 mmol) and stirred at 60 °C for 4 h. Benzylamine (1.147 mL, 10.5 mmol) was then added, followed by EtOAc (10 vol %, 2 mL) and 2 wt % TPGS-750-M (0.5 M, 18 mL) and stirred for 6 h at 60 °C (internal temperature, block set to 65 °C). The resulting contents of the reaction were extracted with EtOAc (3 x 3.0 mL). The crude product was purified via General Purification Method A-2 to provide the desired compound **5** as a white solid (2.25 g, 94 %). R_f = 0.41 (1:1 EtOAc/hexanes).

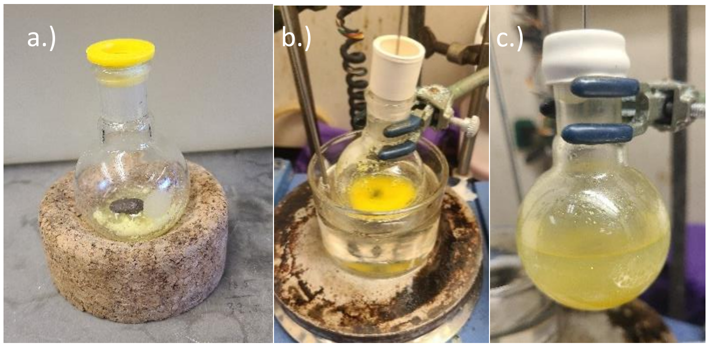

a.) Initial reaction, b.) after thioester formation, c.) after addition of benzyl amine and TPGS-750-M/H_2O

9. Recycle Study

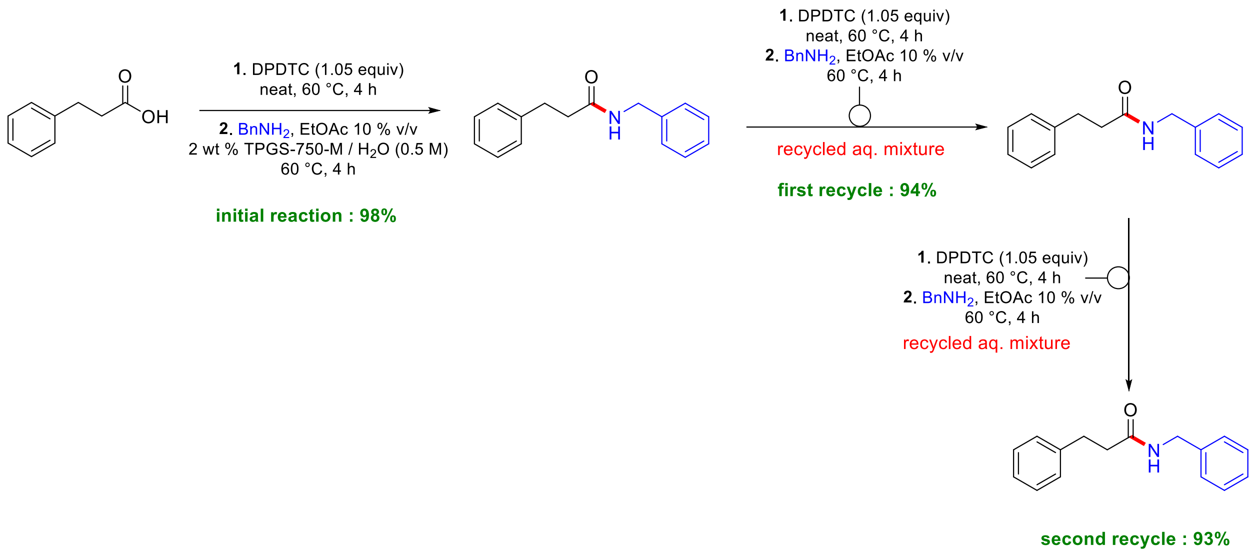

To a 2-dram vial equipped with a Teflon coated stir bar was added phenylpropionic acid (150.2 mg, 1 mmol) and DPDTC (260.7 mg, 1.05 mmol) and stirred at 60 °C for 3 h. Benzylamine (115 µL, 1.05 mmol) was then added, followed by EtOAc (10 vol %, 200 µL) and 2 wt % TPGS-750-M (0.5 M, 1.8 mL) and stirred for 4 h at 60 °C (internal temperature, block set to 65 °C). The resulting contents of the reaction were extracted with EtOAc (3 x 3.0 mL). The combined organic layer was washed with 1 M NaOH (3 x 2 mL) and 1 M HCl (3 x 2 mL). The resulting organic layer was washed with brine (1 x 2 mL) and dried over anhydrous $MgSO_4$ and concentrated *in vacuo* to afford a white solid (234 mg, 98% yield). The recovered 2 wt % TPGS-750-M was used in the followed reaction.

1st Recycle

To a 2-dram vial equipped with a Teflon coated stir bar was added phenylpropionic acid (150.2 mg, 1 mmol) and DPDTC (260.7 mg, 1.05 mmol) and stirred at 60 °C for 3 h. Benzylamine (115 µL, 1.05 mmol) was then added, followed by EtOAc (10 vol %, 200 µL) and the recycled 2 wt % TPGS-750-M (0.5 M, 1.8 mL) from the previous reaction and the resulting mixture was stirred for 4 h at 60 °C (internal temperature, block set to 65 °C). The resulting mixture was purified using the same conditions as stated above, resulting in a white solid (225 mg, 94% yield). The recovered 2 wt % TPGS-750-M/H_2O was used in the followed reaction.

2nd Recycle

To a 2-dram vial equipped with a Teflon coated stir bar was added phenylpropionic acid (150.2 mg, 1 mmol) and DPDTC (260.7 mg, 1.05 mmol) and stirred at 60 °C for 3 h. Benzylamine (115 µL, 1.05 mmol) was then added, followed by EtOAc (10 vol %, 200 µL) and the recycled 2 wt % TPGS-750-M/H_2O (0.5 M, 1.8 mL) from the previous reaction and the mixture was then stirred for 4 h at 60 °C (internal temperature, block set to 65 °C). The resulting mixture was purified using the same conditions as stated above, resulting in a white solid (222 mg, 93% yield).

At this point 0.3 mL of the 2 wt % TPGS-750-M/H_2O solution remained and was lost due to small scale extraction and no further recycling of the aqueous medium was pursued.

10. Multi-step, 1-pot: chemocatalysis sequence procedures

Scheme 1: 3-Step, 1-pot sequence to afford a nitrile as a drug derivative

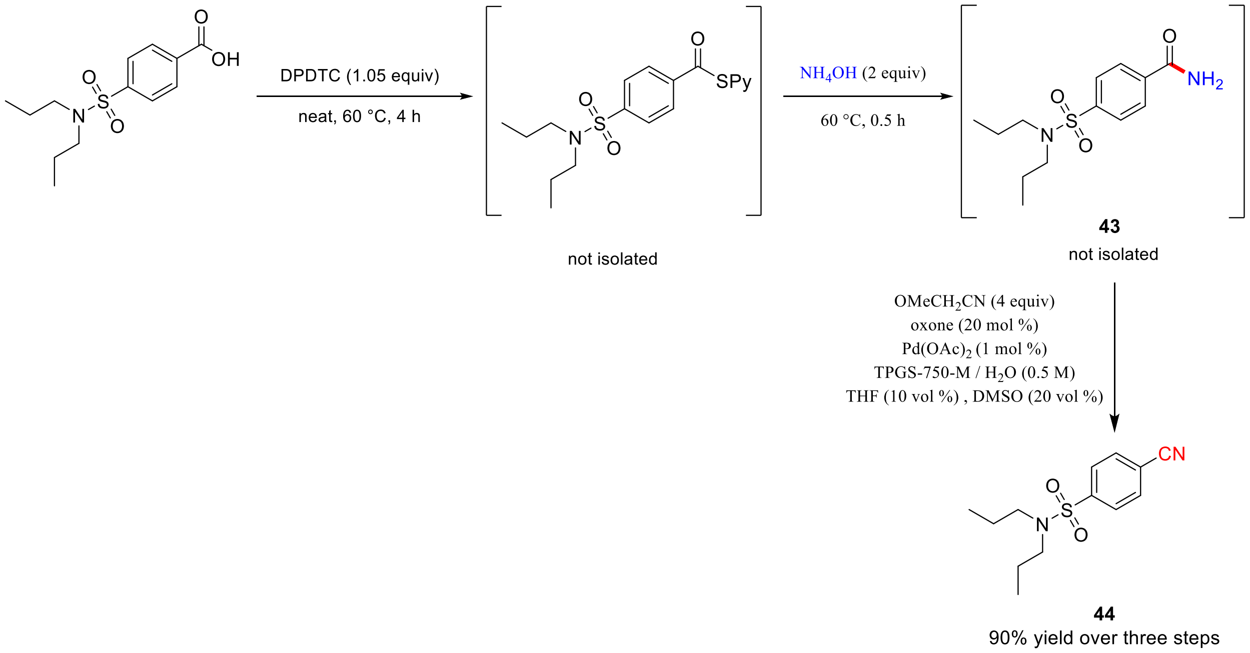

To a 1-dram vial equipped with a PTFE coated stir bar was added was added probenecid (1 equiv, 0.25 mmol, 71.4 mg), DPDTC (1.1 equiv, 0.26 mmol, 65.2 mg) and stirred for 4.5 h at 60 °C internal temperature. The reaction was carried forward without isolation or workup. To the vial, NH_4OH (2 equiv, 0.50 mmol, 63 µL) was then added and stirred for 0.5 h at 60 °C internal temperature. To the reaction mixture was added 1 M NaOH (1 mL x 3) after which the NaOH layer was removed, which removed the by-product 2-mercaptopyridine. The reaction mixture was then washed with water (1 mL x 2). To the vial containing the primary amide **43**, Oxone (20 mole %, 15.4 mg) was added followed by the addition of DMSO (20 vol %, 100 µL). The mixture was then heated with a heat gun until the primary amide dissolved. A 2 wt % TPGS-750-M/H_2O solution (0.5 M, 0.45 mL) was added, followed by methoxyacetonitrile (4 equiv, 1 mmol, 74.4 µL), and from a stock solution of $Pd(OAc)_2$ in THF, 50 µL was then introduced. The stock solution contained 5.6 mg of $Pd(OAc)_2$ in 500 µL of THF. This procedure was followed according to the previously described by Wood, *et al.*[1] The reaction mixture was stirred overnight until complete as determined by TLC. The entire reaction mixture was poured onto a silica plug and eluted with 20 % EtOAc/80% hexanes resulting in a compound **44** white solid (59.9 mg, 90% yield). $\mathbf{R_f}$ = 0.38 (20% EtOAc/80% hexanes).

Scheme 2: 3-Step, 1-pot sequence to afford the nitrile of a drug derivative

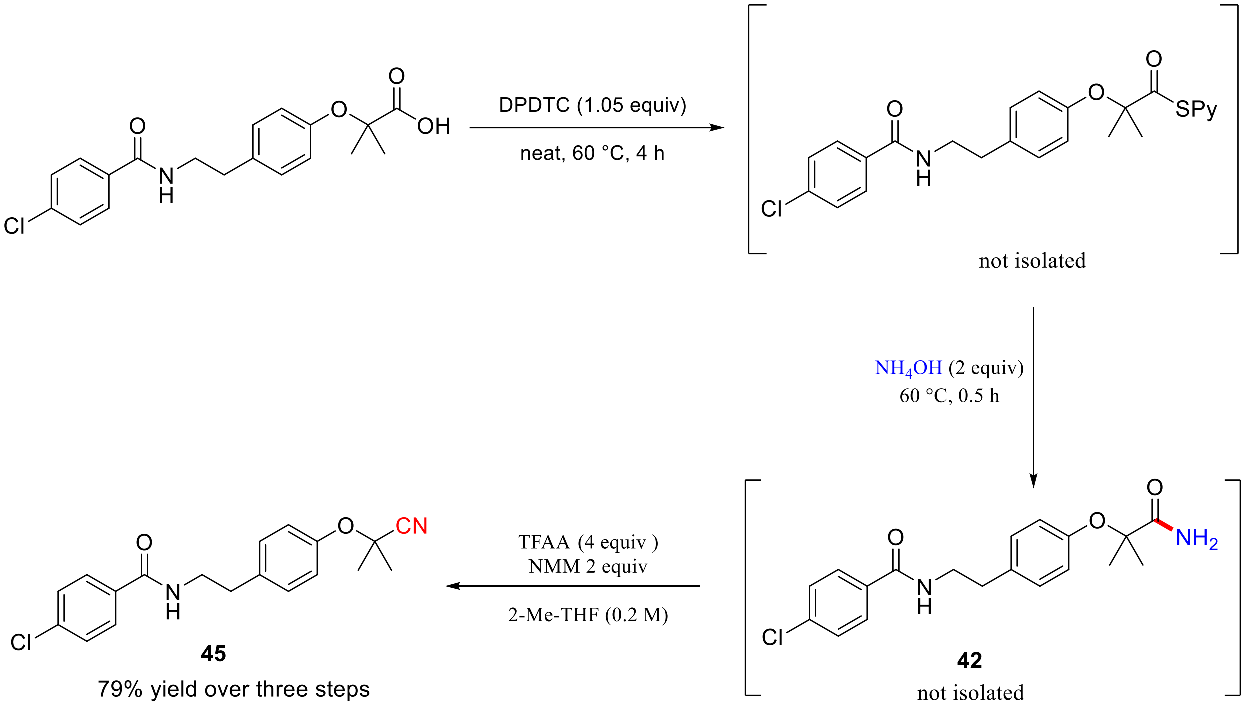

Procedure: To a 1-dram vial equipped with a PTFE coated stir bar was added was added bezafibrate (1 equiv, 0.250 mmol, 90.5 mg) and DPDTC (1 equiv, 0.25 mmol, 65.2 mg) and the mixture was stirred for 3 h at 60 °C internal temperature. The reaction mixture was carried forward without isolation or workup. To the vial, NH_4OH (2 equiv, 0.50 mmol, 63 µL) was added and stirred for 1 h at 60 °C internal temperature. To the reaction mixture was added 1 M NaOH (1 mL x 2) after which the NaOH layer was removed after centrifugation. Then, 1 M HCl (1 mL x 2) was added, followed by deionized water (1 mL x 2). Toluene (1 mL x 2) was added and sequentially evaporated under vacuum to remove any remaining water, as the following amide dehydration is sensitive to the presence of water. To the same vial with the primary amide **42**, NMM (2 equiv, 0.5 mmol, 100 µL) was directly added followed by the addition of 2-MeTHF (0.2 M, 1.25 mL) and trifluoroacetic anhydride (4 equiv, 1 mmol, 140 µL). The reaction was stirred for 10 min on ice followed by stirring for 2 h at rt. The reaction mixture was then quenched with brine and extracted with EtOAc. The combined organics were washed with 1:1 1 M HCl/brine and 1:1 sat $NaHCO_3$/brine, followed by a brine wash. The organics were then dried over anhydrous $MgSO_4$ and concentrated *in vacuo*, resulting in a product **45** white solid (71 mg, 79 % yield). **R_f** = 0.44 (20% EtOAc/80% hexanes).

Scheme 3: A 4-step, 1-pot sequence utilizing a Merck Informer Library acid to form ester **7**

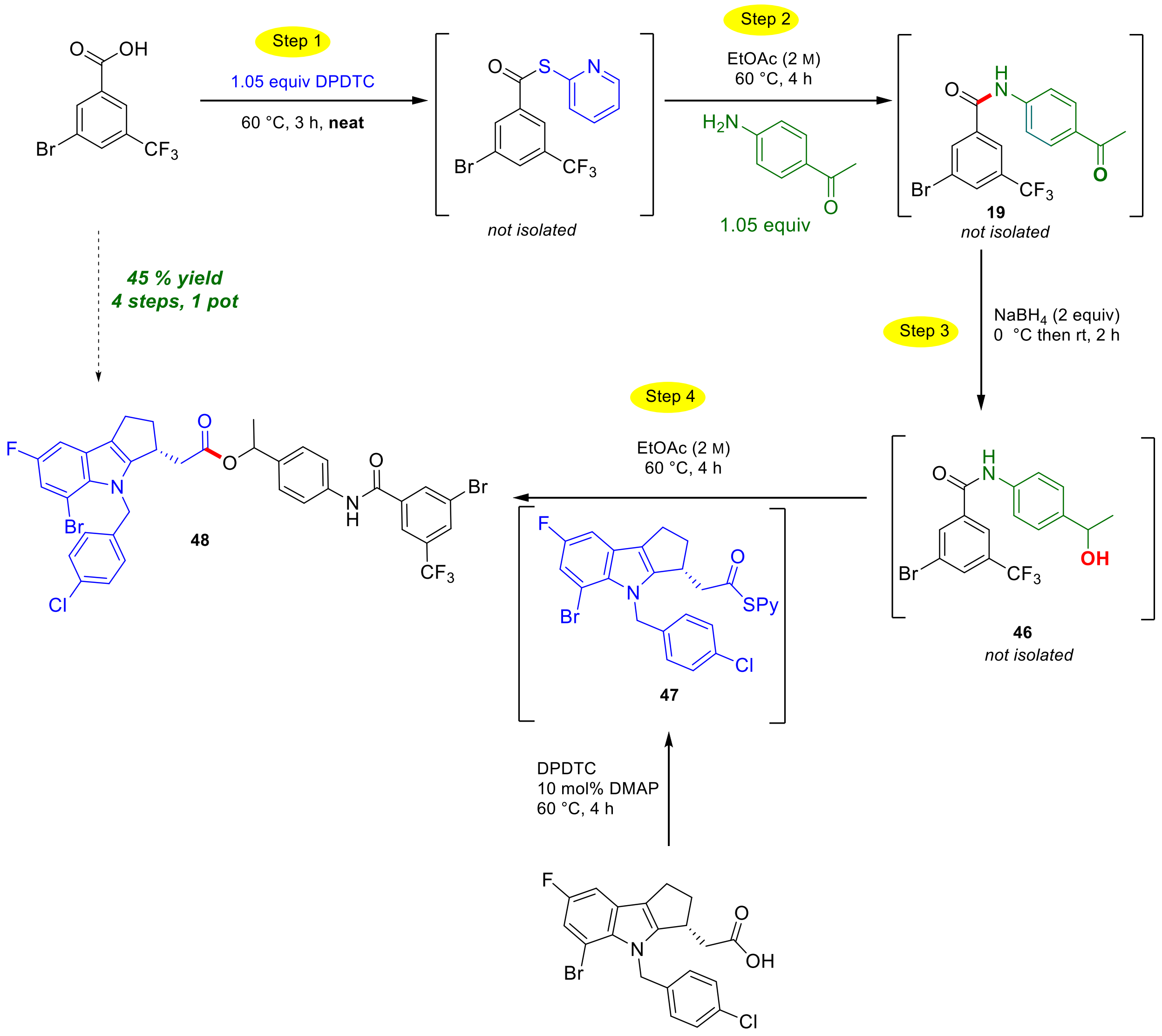

To a 2-dram vial equipped with a PTFE coated stir bar was added was added 3-bromo-5-(trifluoromethyl) benzoic acid (1 equiv, 0.250 mmol, 67.3 mg) and DPDTC (1.05 equiv, 0.263 mmol, 65.3 mg) and the mixture was then stirred for 4 h at 60 °C internal temperature. The reaction was carried forward without isolation or workup. To the vial, 4-aminoacetophenone (1.05 equiv, 0.263 mmol, 35.5 mg) and EtOAc (2 M, 125 µL) was added and stirred for 3 h at 60 °C internal temperature. The reaction mixture was washed with 1 M NaOH (3 x 1 mL), and then 1 M HCl (3 x 1 mL). To the same vial, $NaBH_4$ (2 equiv, 0.5 mmol, 18.9 mg) was added and the mixture was stirred on ice for 10 min and then at rt for 2 h. This mixture was then azeotropically dried with toluene. To the same vial, compound **47** (1 equiv, 0.25 mmol, 132 mg), DMAP (0.10 equiv, 0.025 mmol, 3.1 mg) and dry EtOAc (0.5 M, 125 µL), were added and stirred for 12 h at 60 °C. The EtOAc was dried over anhydrous K_2CO_3 and anhydrous $MgSO_4$ and stored on sieves before use. The reaction was applied directly onto SiO_2 and then purified via column chromatography using a gradient of 10% EtOAc/Hexane) resulting in a product **48** white solid (90 mg, 45% yield). $\mathbf{R_f}$ = 0.40 (20% EtOAc/80% hexanes).

11. Analytical data

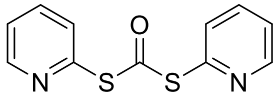

1

S,S-Di(pyridin-2-yl) carbonodithioate (1):

^{1}H NMR (400 MHz, CDCl$_3$): δ 8.58 (ddd, *J* = 4.8, 2.0, 1.0 Hz, 2H), 7.73 – 7.60 (m, 4H), 7.26 (ddd, *J* = 7.3, 4.8, 1.5 Hz, 2H).

^{13}C NMR (101 MHz, CDCl$_3$): δ 185.60, 150.60, 150.58, 137.46, 130.43, 124.12, 77.48, 77.16, 76.85.

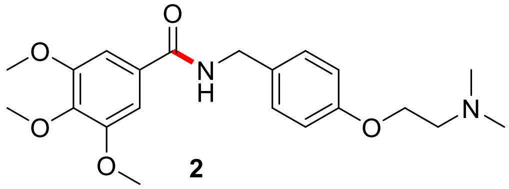

2

***N*-[*p*-[(2-Dimethylamino)ethoxy]benzyl]-3,4,5-trimethoxybenzamide (2):** Compound (**2**) was prepared according to Method C with the following modifications. In step 1: 1.5 equiv of DPDTC and 0.5 M TPGS-750-M/H_2O for 16 h at 45 °C. In step 2: 1.1 equiv of amine was added at rt for 16 h. The crude product was purified by flash column chromatography (10% EtOAc/hexanes) afforded **1** (88.4 mg, 91% yield) as a white solid; **R_f:** 0.57 (30% EtOAc/hexanes). R_f: 0.36 (30% MeOH/70% DCM).

^{1}H NMR (400 MHz, CDCl$_3$): δ 7.30 – 7.26 (m, 2H), 7.00 (s, 2H), 6.94 – 6.86 (m, 2H), 6.28 (s, 1H), 4.57 (d, *J* = 5.6 Hz, 2H), 4.10 (t, *J* = 5.6 Hz, 2H), 3.89 (s, 6H), 3.87 (s, 3H), 2.81 (t, *J* = 5.6 Hz, 2H), 2.39 (s, 6H).

^{13}C NMR (126 MHz, CDCl$_3$): δ 167.08, 158.41, 153.37, 141.13, 130.62, 130.02, 129.50, 114.99, 104.52, 77.41, 77.16, 76.91, 65.88, 61.07, 58.18, 56.52, 45.76, 43.89. Spectral data matched those previously reported.[2]

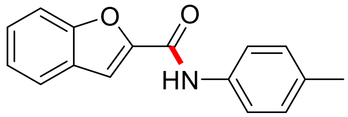

3

Benzofuran-2-carboxylic acid *N*-(4-methylphenyl)amide (3): Compound **3** was prepared according to Method C with the following modifications. In step 1: 1.5 equiv of DPDTC, 10 mol % DMAP, and 0.5 M TPGS-750-M/H_2O for 16 h at 45 °C. In step 2: 1.1 equiv of amine was added at rt for 16 h. The crude product was purified by flash column chromatography (30 % EtOAc in hexanes) afforded **1** (37.1 mg, 59% yield) as white solid. **R_f:** 0.40 (30% EtOAc/70% hexanes).

^{1}H NMR (500 MHz, $CDCl_3$): δ 8.31 (s, 1H), 7.73 (dt, *J* = 7.8, 1.0 Hz, 1H), 7.65 – 7.57 (m, 4H), 7.48 (ddd, *J* = 8.4, 7.2, 1.3 Hz, 1H), 7.35 (ddd, *J* = 8.0, 7.3, 1.0 Hz, 1H), 7.23 (d, *J* = 8.2 Hz, 2H), 2.38 (s, 3H).

^{13}C NMR (126 MHz, $CDCl_3$): δ 156.48, 154.80, 148.65, 134.68, 134.53, 129.69, 127.77, 127.18, 123.91, 122.86, 120.04, 111.80, 111.27, 77.28, 77.03, 76.77, 20.96. Spectral data matched those previously reported.[3]

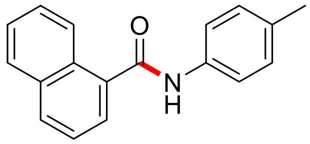

4

***N*-(*p*-Tolyl)-1-naphthamide (4):** Compound **4** was prepared according to Method C with the following modifications. In step 1: 1.5 equiv of DPDTC, 10 mol % DMAP, and 0.5 M TPGS-750-M/H_2O for 16 h at 45 °C. In step 2: 1.1 equiv of amine was added at rt for 16 h. The crude product was purified by flash column chromatography (30 % EtOAc in hexanes) afforded **3** (60.6 mg, 98% yield) as white solid; **R_f:** 0.20 (30% EtOAc/70% hexanes).

^{1}H NMR (500 MHz, $CDCl_3$): δ 8.40 (d, *J* = 8.1 Hz, 1H), 7.99 (d, *J* = 8.3 Hz, 1H), 7.95 – 7.91 (m, 1H), 7.77 (d, *J* = 7.0 Hz, 1H), 7.64 (s, 1H), 7.62 – 7.51 (m, 5H), 7.24 (d, *J* = 7.9 Hz, 2H), 2.39 (s, 3H).

^{13}C NMR (101 MHz, $CDCl_3$): δ 167.47, 135.51, 134.63, 134.36, 133.77, 130.95, 130.11, 129.64, 128.42, 127.32, 126.57, 125.33, 125.06, 124.75, 120.07, 77.37, 77.05, 76.74, 20.96. Spectral data matched those previously reported.[4]

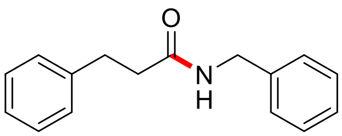

5

***N*-Benzyl-3-phenylpropionamide (5):** Compound (**5**) was obtained using General Procedure A on a 0.25 mmol scale. The crude product was purified via General Purification Method 1-B, as described in Section 5.2, to provide the desired compound as a white solid (53 mg, 88%). **R_f =** 0.41 (50% EtOAc/50% hexanes).

^{1}H NMR (400 MHz, $CDCl_3$): δ 7.36 – 7.26 (m, 5H), 7.24 – 7.11 (m, 5H), 5.58 (s, 1H), 4.40 (d, *J* = 5.5 Hz, 2H), 3.00 (t, *J* = 7.6 Hz, 2H), 2.52 (t, *J* = 7.6 Hz, 2H).

^{13}C NMR (126 MHz, $CDCl_3$): δ 172.03, 140.80, 138.19, 128.66, 128.57, 128.42, 127.72, 127.44, 126.27, 43.55, 38.44, 31.74. Spectral data matched those previously reported.[5]

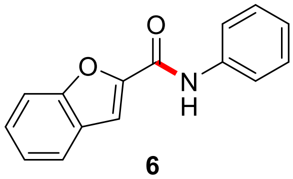

6

***N*-Phenylbenzofuran-2-carboxamide (6):** Compound (**6**) was obtained using General Procedure A on a 0.25 mmol scale. The crude product was purified via General Purification Method 1-A, as described in Section 5.1, to provide the desired compound as a white solid (53 mg, 90%). **R_f** = 0.71 (50% EtOAc/50% hexanes).

^{1}H NMR (400 MHz, $CDCl_3$): δ 8.34 (s, 1H), 7.72 (ddt, *J* = 7.6, 5.4, 1.2 Hz, 3H), 7.60 (d, *J* = 1.0 Hz, 1H), 7.57 (dq, *J* = 8.2, 0.9 Hz, 1H), 7.46 (ddd, *J* = 8.4, 7.2, 1.3 Hz, 1H), 7.43 – 7.36 (m, 2H), 7.33 (ddd, *J* = 8.1, 7.2, 1.0 Hz, 1H), 7.22 – 7.15 (m, 1H).**^{13}C NMR (126 MHz, $CDCl_3$):** δ 156.64, 154.83, 148.48, 137.23, 129.20, 127.73, 127.29, 124.86, 123.96, 122.91, 120.07, 111.84, 111.50. Spectral data matched those previously reported.[6]

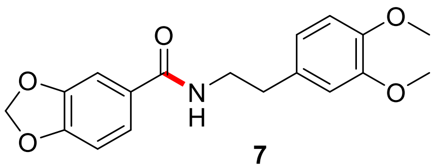

7

***N*-(3,4-Dimethoxyphenethyl)-3,4-methylenedioxybenzamide (7):** Compound **7** was obtained using General Procedure A on a 0.25 mmol scale. The crude product was purified via General Purification Method 1-A, as described in Section 5.1, to provide the desired compound as a white solid (81.6 mg, 99%). **R_f** = 0.24 (50% EtOAc/50% hexanes).

^{1}H NMR (400 MHz, $CDCl_3$): δ 7.19 (d, J = 7.6 Hz, 2H), 6.84 – 6.71 (m, 4H), 6.08 (s, 1H), 6.00 (s, 2H), 3.85 (d, J = 9.0 Hz, 6H), 3.65 (q, J = 6.4 Hz, 2H), 2.85 (t, J = 6.7 Hz, 2H).

13C NMR (101 MHz, $CDCl_3$:): δ 166.75, 150.28, 149.09, 147.97, 147.74, 131.45, 128.87, 121.34, 120.69, 111.99, 111.43, 107.99, 107.53, 101.69, 55.87, 41.32, 35.26. Spectral data matched those previously reported.[7]

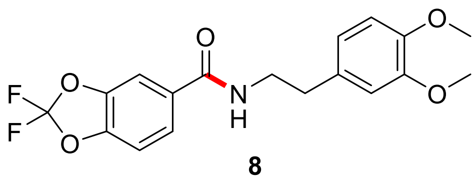

8

***N*-(3,4-Dimethoxyphenethyl)benzo[d][1,3]dioxole-5-carboxamide (8):** Compound **8** was obtained using General Procedure A on a 0.25 mmol scale. The crude product was purified via General Purification Method 1-A, as described in Section 5.1, to provide the desired compound as a white solid (89.7 mg, 98%). $\mathbf{R_f}$ = 0.50 (50% EtOAc/50% hexanes).

^{1}H NMR (400 MHz, $CDCl_3$): δ 7.47 (d, *J* = 1.7 Hz, 1H), 7.41 (dd, *J* = 8.3, 1.8 Hz, 1H), 7.07 (d, *J* = 8.2 Hz, 1H), 6.83 (d, *J* = 7.9 Hz, 1H), 6.79 – 6.72 (m, 2H), 6.02 (d, *J* = 7.2 Hz, 1H), 3.86 (d, *J* = 7.4 Hz, 6H), 3.69 (td, *J* = 6.8, 5.8 Hz, 2H), 2.88 (t, *J* = 6.9 Hz, 2H).

^{13}C NMR (126 MHz, $CDCl_3$): δ 165.78, 149.17, 147.85, 145.87, 143.95, 131.67, 131.14, 131.06, 122.74, 120.68, 111.90, 111.43, 109.21, 108.79, 77.30, 77.05, 76.79, 55.94, 55.88, 41.41, 35.14.

^{19}F NMR (376 MHz, $CDCl_3$): δ -49.78.

HRMS: *m/z* calcd for $C_{18}H_{19}F_2NO_5$; 366.115305 [M+1]: found 366.1127.

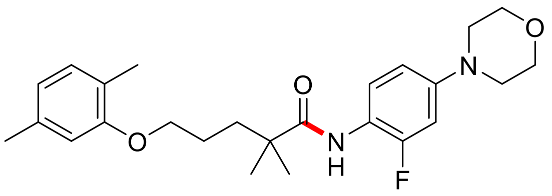

9

5-(2,5-Dimethylphenoxy)-*N*-(2-fluoro-4-morpholinophenyl)-2,2-dimethylpentanamide (9): Compound **9** was obtained using General Procedure A on a 0.25 mmol scale. The crude product was purified via General Purification Method 2, followed by flash chromatography (eluent: gradient 20% to 35% EtOAc/hexanes) to provide the desired compound as a white solid (104 mg, 97%). $\mathbf{R_f}$ = 0.60 (50% EtOAc/50% hexanes).

^{1}H NMR (500 MHz, CDCl$_3$): δ 7.48 (dd, *J* = 14.1, 2.5 Hz, 1H), 7.32 (s, 1H), 7.09 (dd, *J* = 9.0, 2.4 Hz, 1H), 7.02 (d, *J* = 7.4 Hz, 1H), 6.90 (t, *J* = 9.0 Hz, 1H), 6.68 (d, *J* = 7.5 Hz, 1H), 6.63 (s, 1H), 3.96 (d, *J* = 5.2 Hz, 2H), 3.92 – 3.86 (m, 4H), 3.11 – 3.02 (m, 4H), 2.31 (s, 3H), 2.19 (s, 3H), 1.88 – 1.80 (m, 4H), 1.35 (s, 6H).

^{13}C NMR (126 MHz, CDCl$_3$): δ 175.64, 156.82, 156.41, 154.45, 136.58, 133.15 (d, *J* = 10.6 Hz), 130.36, 123.51, 120.91, 118.75, 115.84 (d, *J* = 3.2 Hz), 112.20, 109.31 (d, *J* = 25.3 Hz), 77.29, 77.04, 76.78, 67.84, 66.99, 51.15, 42.79, 37.66, 25.62, 25.15, 21.40, 15.85.

^{19}F NMR (376 MHz, CDCl$_3$): δ -121.05.

HRMS: *m/z* calcd for $C_{22}H_{25}F_6N_2O_3S$; 429.5223 [M+1]: found 429.5222.

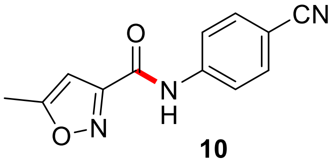

10

***N*-(4-Cyanophenyl)-5-methylisoxazole-3-carboxamide (10):** Compound **10** was obtained using General Procedure A on a 0.25 mmol scale. The crude product was purified via General Purification Method 2, followed by flash chromatography (eluent: gradient 30% EtOAc/70% hexanes to 50% EtOAc/50% hexanes) to provide the desired compound as a pale-yellow solid (54.5 mg, 96%). R_f = 0.29 (30% EtOAc/70% hexanes).

^{1}H NMR (400 MHz, CDCl$_3$): δ 8.69 (s, 1H), 7.83 – 7.76 (m, 2H), 7.72 – 7.64 (m, 2H), 6.54 (d, *J* = 1.0 Hz, 1H), 2.53 (s, 3H).

^{13}C NMR (101 MHz, CDCl$_3$): δ 172.27, 158.46, 157.11, 140.99, 133.45, 119.83, 118.64, 107.96, 101.52, 12.46.

HRMS: *m/z* calcd for $C_{12}H_{10}N_3O_2$; 228.077302 [M+1]: found 228.0768.

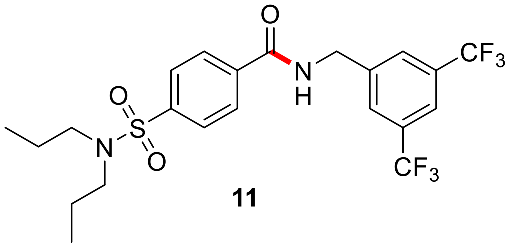

11

***N*-3,5-*bis*(Trifluoromethyl)benzyl)-4-(*N,N*-dipropylsulfamoyl)benzamide (11):** Compound **11**was obtained using General Procedure A on a 0.25 mmol scale. The crude product was purified

via General Purification Method 2, followed by flash chromatography (eluent: gradient 20% EtOAc/80% hexanes to 60% EtOAc/40% hexanes) to provide the desired compound as a white solid (101.2 mg, 79%). **R_f** = 0.37 (30% EtOAc/70% hexanes).

^{1}H NMR (400 MHz, $CDCl_3$): δ 7.88 – 7.78 (m, 5H), 7.73 (dd, *J* = 8.4, 2.3 Hz, 2H), 7.19 (p, *J* = 5.9 Hz, 1H), 4.75 (d, *J* = 6.0 Hz, 2H), 3.10 – 2.97 (m, 4H), 1.50 (h, *J* = 7.4 Hz, 4H), 0.83 (t, *J* = 7.4 Hz, 6H).

^{13}C NMR (126 MHz, $CDCl_3$): δ 166.57, 142.89, 140.78, 137.52, 132.01 (q, *J* = 33.3 Hz), 128.05 (q, *J* = 3.8 Hz), 127.93, 127.22, 124.29, 121.60 (h, *J* = 4.0 Hz), 77.29, 77.03, 76.78, 49.93, 43.31, 21.87, 11.09.

^{19}F NMR: (471 MHz, $CDCl_3$): δ -62.82.

HRMS: *m/z* calcd for $C_{22}H_{25}F_6N_2OS$; 511. 149008 [M+1]: found 511.1490.

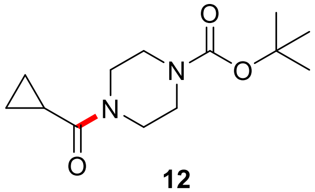

12

1-*t*-Butoxycarbonyl-4-(cyclopropanecarbonyl) piperazine (12): Compound **12** was prepared according to Method A as described above with modification of reaction time: thioester formation (4 h) and amide formation (4 h). The crude product was purified by flash column chromatography (15% EtOAc/80% hexanes) to afford **12** (55.2 mg, 87% yield) as white solid; **R_f** = 0.25 (30% EtOAc/70% hexanes).

^{1}H NMR (400 MHz, $CDCl_3$): δ 3.62 (s, 4H), 3.45 (d, *J* = 23.2 Hz, 4H), 1.71 (ddd, *J* = 12.7, 8.0, 4.7 Hz, 1H), 1.47 (s, 9H), 1.04 - 0.96 (m, 2H), 0.78 (dt, *J* = 8.0, 3.3 Hz, 2H).

^{13}C NMR (101 MHz, $CDCl_3$): δ 172.24, 154.65, 80.22, 45.35, 44.04, 43.48, 41.91, 28.41, 11.06, 7.56. Spectral data matched those previously reported.[8]

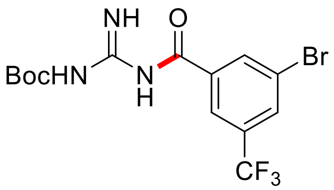

13

***t*-Butyl *N-[N*-(3-(trifluoromethyl)-5-bromo-benzoyl) carbamimidoyl]carbamate (13):** Compound **13** was prepared according to Method A as described above with modification of reaction time: thioester formation (4 h) and amide formation (4 h). The NMM (1 equiv) was added during amide bond formation. The crude product was purified by flash column chromatography

(10% EtOAc/hexanes) to afford **13** (96.4 mg, 94% yield) as a white solid; **R$_f$:** 0.55 (15% EtOAc/85% hexanes).

^{1}H NMR (400 MHz, CDCl$_3$): δ 9.06 (s, 1H), 8.63 (s, 2H), 8.49 (t, *J* = 1.7 Hz, 1H), 8.37 (s, 1H), 7.85 (d, *J* = 2.0 Hz, 1H), 1.52 (s, 9H).

^{13}C NMR (126 MHz, CDCl$_3$): δ 175.67, 159.62, 152.92, 140.22, 135.50, 132.14 (q, *J* = 33.2 Hz), 130.97 (q, *J* = 3.78 Hz), 124.76, (q, *J* = 3.78 Hz), 122.93, (q, *J* = 273.4 Hz), 83.93, 27.77.

^{19}F NMR (376 MHz, CDCl$_3$): δ -62.80.

HRMS: *m/z* calcd for $C_{14}H_{15}BrF_3N_3O_3$; [M+1]: 410.0327 found 410.0318.

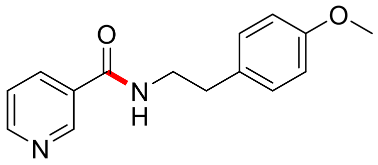

14

***N*-[2-(4-Methoxyphenyl)ethyl]-3-pyridinecarboxamide (14):** Compound **14** was obtained using General Procedure B on a 0.25 mmol scale. The crude product was purified via General Purification Method A to provide the desired compound as a pale-yellow oil (59 mg, 92%). **R$_f$** = 0.44 (50% EtOAc/50% hexanes).

^{1}H NMR (500 MHz, CDCl$_3$): δ 8.72 – 8.67 (m, 1H), 7.86 (dd, *J* = 6.3, 2.8 Hz, 1H), 7.81 (td, *J* = 7.6, 1.9 Hz, 1H), 7.76 (dt, *J* = 7.9, 1.2 Hz, 1H), 7.37 (q, *J* = 4.3 Hz, 3H), 7.31 (t, *J* = 8.8 Hz, 4H), 2.32 (s, 3H).

^{13}C NMR (126 MHz, CDCl$_3$): δ 164.27, 158.26, 149.96, 148.05, 137.35, 131.00, 129.75, 126.09, 122.20, 114.04, 55.28, 40.97, 35.06.

HRMS: *m/z* calcd for $C_{15}H_{17}N_2O_2$; 257.1290 [M+1]: found 257.1294.

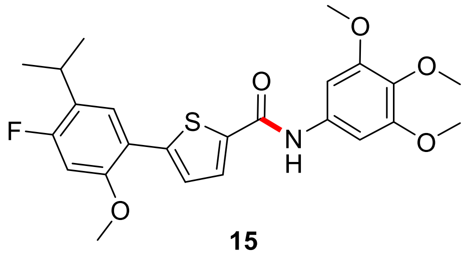

15

5-(4-Fluoro-5-isopropyl-2-methoxyphenyl)-*N*-(3,4,5-trimethoxyphenyl) thiophene-2-carboxamide (15): Compound **15** was obtained using the General Procedure, Method B, on a 0.25

mmol scale. The crude product was purified via General Purification Method 2, followed by silica gel column chromatography (eluent: gradient 30% EtOAc/70% hexanes to 50% EtOAc/50% hexanes) to provide the desired compound as a pale-yellow solid (101 mg, 89% yield). R_f = 0.46 (50% EtOAc/50% hexanes).

^{1}H NMR (400 MHz, $CDCl_3$): δ 7.81 (s, 1H), 7.61 (t, *J* = 5.2 Hz, 1H), 7.51 (dd, *J* = 8.4, 2.6 Hz, 1H), 7.41 (d, *J* = 4.0 Hz, 1H), 6.97 (s, 2H), 6.69 (dd, *J* = 11.8, 2.8 Hz, 1H), 3.92 (d, *J* = 4.5 Hz, 3H), 3.89 – 3.80 (m, 9H), 3.19 (p, *J* = 6.9 Hz, 1H), 1.28 (dd, *J* = 7.0, 2.2 Hz, 6H).

^{13}C NMR (126 MHz, $CDCl_3$): δ 162.06, 160.42, 160.08, 154.97 (d, *J* = 10.1 Hz), 153.36, 144.74, 137.16, 134.78, 133.95, 128.23, 127.62 (d, *J* = 15.6 Hz), 126.96 (d, *J* = 7.4 Hz), 125.12, 118.12, 99.95 (d, *J* = 27.6 Hz), 97.85, 77.30, 77.05, 76.79, 61.01, 56.11, 55.96, 26.94, 22.75.

^{19}F NMR (376 MHz, $CDCl_3$): δ -114.95 (dd, *J* = 11.9, 8.5 Hz).

HRMS: *m/z* calcd for $C_{24}H_{27}FNO_5S$; 460.159399 [M+1]: found 460.1574.

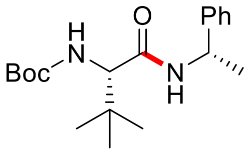

16

***N*-(*t*-Butoxycarbonyl)-(*S*)-alanine (*R*)-1-phenylethylamide (16):** Compound **16** was prepared according to Method B as described above with modification of 10 mol % DMAP, EtOAc (2 M), rt, then rt, and reaction time. The thioester formation (12 h) and amide formation (12 h.) The crude product was purified by flash column chromatography (20% EtOAc in hexanes) afforded **16** (77.5 mg, 93% yield) as white solid; **R_f** = 0.57 (30% EtOAc/70% hexanes).

^{1}H NMR (400 MHz, DMSO-*d_6*): δ 8.30 (d, *J* = 8.1 Hz, 1H), 7.29 (d, *J* = 1.5 Hz, 2H), 7.21 (tdd, *J* = 5.1, 4.0, 2.8 Hz, 1H), 6.37(d, *J* = 9.7 Hz, 1H), 4.94 (p, *J* = 7.2 Hz, 1H), 3.87 (d, *J* = 9.7 Hz, 1H), 1.39 (s, 9H), 1.35 (d, *J* = 7.0 Hz, 3H), 0.82 (s, 9H).

^{13}C NMR (101 MHz, DMSO-*d_6*): δ 169.31, 155.22, 144.39, 128.13, 126.64, 126.11, 78.08, 61.85, 47.77, 34.21, 28.14, 26.62, 22.35. Spectral data matched those previously reported.[9]

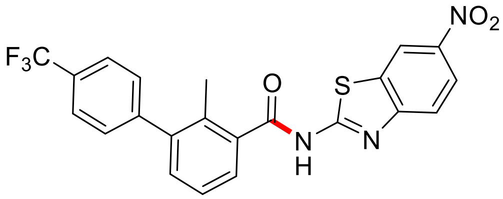

17

2-Methyl-*N*-(6-nitrobenzo[d]thiazol-2-yl)-4'-(trifluoromethyl)-[1,1'-biphenyl]-3-carboxamide (17): Compound **17** was obtained using General Procedure B on a 0.25 mmol scale. The crude product was purified via General Purification Method 2, followed by flash chromatography (eluent: 30% EtOAc/70% hexanes) to provide the desired compound as a yellow solid (93.4 mg, 82%). $\mathbf{R_f}$ = 0.66 (50% EtOAc/50% hexanes).

^{1}H NMR (400 MHz, CDCl$_3$): δ 8.72 – 8.67 (m, 1H), 7.86 (dd, *J* = 6.3, 2.8 Hz, 1H), 7.81 (td, *J* = 7.6, 1.9 Hz, 1H), 7.76 (dt, *J* = 7.9, 1.2 Hz, 1H), 7.41 – 7.34 (m, 3H), 7.31 (t, *J* = 8.8 Hz, 4H), 2.32 (s, 3H).

^{13}C NMR (126 MHz, CDCl$_3$): δ 192.33, 151.84, 150.60, δ 148.54 (d, *J* = 2.3 Hz), 142.55, 139.70, 138.51, 137.29, 134.03, 133.39, 130.72, 130.49, 127.72, 125.74, 123.73, 121.54, 121.13, 120.76, 119.70, 119.50, 17.77.

^{19}F NMR (376 MHz, CDCl$_3$): δ -57.78.

HRMS: *m/z* calcd for $C_{21}H_{16}N_3O_3S$; 390.091239 [M+2]: found 390.0912.

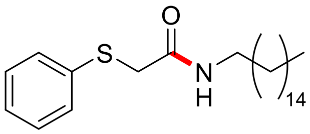

18

***N*-Hexadecyl-2-(phenylthio)acetamide (18):** Compound **18** was obtained using General Procedure C on a 0.25 mmol scale. The crude product was purified via extraction with EtOAc (2 mL x 3). The EtOAc was washed with 1 M NaOH (2 mL x 3), followed by a brine wash (2 mL x 1). The EtOAc was evaporated via rotary evaporation and the resulting solid was washed with acetone, removing the yellow color and providing the desired compound as a white solid (97 mg, 99%). $\mathbf{R_f}$ = 0.70 (50% EtOAc/50% hexanes).

^{1}H NMR (500 MHz, CDCl$_3$): δ 7.28 (s, 2H), 7.06 (t, *J* = 7.6 Hz, 2H), 6.97 (t, *J* = 7.4 Hz, 1H), 6.32 (s, 1H), 3.42 (s, 2H), 3.14 (q, *J* = 6.7 Hz, 2H), 1.54 – 1.36 (m, 20H), 1.31 – 1.18 (m, 6H), 1.10 (q, *J* = 8.1 Hz, 2H), 1.02 (t, *J* = 6.6 Hz, 3H).

^{13}C NMR (126 MHz, $CDCl_3$): 166.36, 135.61, 129.06, 127.99, 127.89, 127.69, 127.50, 127.32, 125.91, 70.64, 39.42, 36.56, 31.99, 29.86, 29.84, 29.81, 29.79, 29.71, 29.62, 29.50, 29.48, 29.30, 26.69, 22.77, 14.02.

HRMS: *m/z* calcd for $C_{24}H_{42}NOS$; 392.2987 [M+1]: found 392.2978.

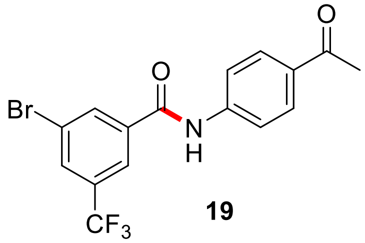

19

***N*-(4-Acetylphenyl)-3-bromo-5-(trifluoromethyl)benzamide (19):** Compound **19** was obtained using General Procedure C on a 0.25 mmol scale. The crude product was purified via General Purification Method 3, followed by flash chromatography (eluent: 30% EtOAc/70% hexanes) to provide the desired compound as a white solid (87 mg, 90%). $\mathbf{R_f}$ = 0.66 (50% EtOAc/ 50% hexanes)

^{1}H NMR (400 MHz, $CDCl_3$): δ 8.64 (s, 1H), 8.21 (d, *J* = 1.7 Hz, 1H), 8.07 (d, *J* = 1.9 Hz, 1H), 7.99 – 7.93 (m, 2H), 7.91 (d, *J* = 1.8 Hz, 1H), 7.81 – 7.74 (m, 2H), 2.59 (s, 3H).

^{13}C NMR (126 MHz, $CDCl_3$): δ 197.45, 163.27, 141.85, 137.14, 133.83, 133.51, 132.93 (q, *J* = 33.7 Hz), 131.77 (q, *J* = 3.7 Hz), 129.82, 123.73, 123.43, 122.99, 122.96, 122.94, 122.91, 119.80, 77.30, 77.04, 76.79, 26.54.

^{19}F NMR (376 MHz, $CDCl_3$): δ -62.83.

HRMS: *m/z* calcd for $C_{16}H_{12}BrF_3NO_2$; [M+1] 386.000349: found 386.0044.

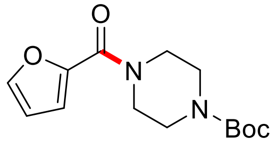

20

***t*-Butyl 4-(furan-2-carbonyl)piperazine-1-carboxylate (20):** Compound **20** was obtained using General Procedure C on a 0.5 mmol scale. The crude product was purified via General Purification Method 2, followed by flash chromatography (eluent: gradient 10% EtOAc/90% hexanes to 30% EtOAc/70% hexanes) to provide the desired compound as a white solid (108 mg, 77%). $\mathbf{R_f}$ = 0.55 (50% EtOAc/50% hexanes).

^{1}H NMR (400 MHz, $CDCl_3$): δ 7.48 (dd, *J* = 1.8, 0.8 Hz, 1H), 7.03 (dd, *J* = 3.4, 0.8 Hz, 1H), 6.49 (dd, *J* = 3.5, 1.8 Hz, 1H) , 3.77 (s, 4H), 3.54 – 3.47 (m, 4H), 1.48 (s, 9H).

^{13}C NMR (126 MHz, CDCl$_3$): δ 159.25, 154.59, 147.77, 143.85, 116.85, 111.42, 80.32, 43.41, 28.39. Spectral data matched those previously reported.[10]

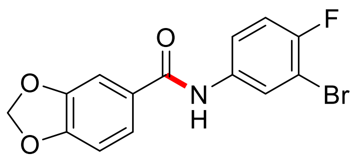

21

***N*-(3-Bromo-4-fluorophenyl)benzo[d][1,3]dioxole-5-carboxamide (21):** Compound **21** was obtained using General Procedure C on a 0.25 mmol scale. The crude product was purified via General Purification Method 2, followed by flash chromatography (eluent: gradient 20% EtOAc/80% hexanes to 50% EtOAc/50% hexanes, and 100% EtOAc until all product eluted) to provide the desired compound as a white solid (59 mg, 70%). **R_f** = 0.39 (30% EtOAc/70% hexanes)

^{1}H NMR (400 MHz, DMSO-*d$_6$*): δ 10.34 (s, 1H), 7.94 – 7.86 (m, 1H), 7.62 (td, *J* = 8.4, 2.5 Hz, 1H), 7.60 – 7.46 (m, 3H), 7.04 (dd, *J* = 8.2, 2.6 Hz, 1H), 6.12 (d, *J* = 2.8 Hz, 2H).

^{13}C NMR (101 MHz, DMSO-*d$_6$*): δ 165.29, 159.60, 157.20, 150.84, 147.91, 141.01 (d, J = 10.3 Hz), 133.56, 128.53, 123.57, 117.94 (d, J = 3.1 Hz), 108.53 (d, J = 27.27 Hz), 108.36 (d, J = 23.23 Hz), 102.39, 101.42 (d, J = 21.1 Hz), 40.61, 40.40, 40.19, 39.98, 39.78, 39.57, 39.36.

^{19}F NMR (376 MHz, DMSO-*d$_6$*): δ -107.17 (t, *J* = 9.8 Hz).

HRMS: *m/z* calcd for $C_{14}H_9BrFNO_3Na$; 359.9647 [M+23]: found 359.9639

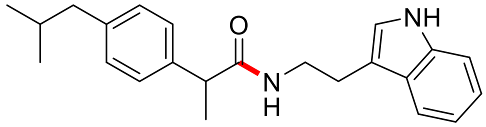

22

***N*-(2-(1H-Indol-3-yl)ethyl)-2-(4-isobutylphenyl)propanamide (22):** Compound **22** was obtained using General Procedure C on a 0.25 mmol scale. The crude product was purified via General Purification Method 3, followed by flash chromatography (eluent: 30% EtOAc/70% hexanes) to provide the desired compound as a white solid (85.4 mg, 98%). **R_f =** 0.66 (50% EtOAc/ 50% hexanes).

^{1}H NMR (500 MHz, CDCl$_3$): δ 8.38 (s, 1H), 7.57 (d, *J* = 7.9 Hz, 1H), 7.46 – 7.33 (m, 1H), 7.25 – 7.01 (m, 6H), 6.77 (s, 1H), 5.57 (s, 1H), 3.66 – 3.52 (m, 2H), 3.49 (q, *J* = 8.4 Hz, 1H), 2.92 (hept, *J* = 7.7 Hz, 2H), 2.59 – 2.43 (m, 2H), 1.90 (p, *J* = 7.0 Hz, 1H), 1.54 (s, 3H), 1.04 – 0.87 (m, 6H).

^{13}C NMR (126 MHz, CDCl$_3$): δ 174.57, 140.63, 138.61, 136.42, 129.57, 127.40, 127.30, 122.18, 122.04, 119.36, 118.67, 112.69, 111.32, 77.38, 77.13, 76.87, 46.83, 45.06, 39.91, 30.25, 25.19, 22.46, 18.52.

HRMS: *m/z* calcd for $C_{23}H_{28}N_2ONa$; 371.2099 [M+23]: found 371.2088.

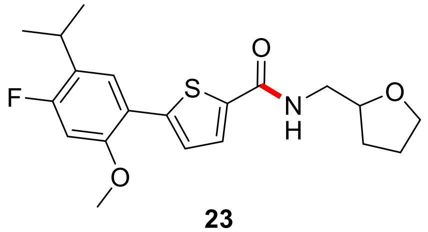

23

5-(4-Fluoro-5-isopropyl-2-methoxyphenyl)-*N*-((tetrahydrofuran-2-yl)methyl)thiophene-2-carboxamide (23): Compound **23** was obtained using General Procedure C on a 0.25 mmol scale. The crude product was purified via General Purification Method 3, followed by flash chromatography (eluent: 30 % EtOAc/70 % hexanes) to provide the desired compound as a white solid (89.6 mg, 95%). **R_f =** 0.33 (50% EtOAc/50% hexanes).

^{1}H NMR (500 MHz, CDCl$_3$): δ 7.54 – 7.45 (m, 2H), 7.38 (d, *J* = 3.8 Hz, 1H), 6.69 (dd, *J* = 11.9, 2.6 Hz, 1H), 6.50 – 6.35 (m, 1H), 4.10 (qd, *J* = 7.2, 3.3 Hz, 1H), 3.92 (dd, *J* = 5.7, 3.0 Hz, 3H), 3.85 – 3.75 (m, 2H), 3.38 (ddd, *J* = 13.3, 7.2, 5.0 Hz, 1H), 3.20 (hept, *J* = 7.0 Hz, 1H), 2.05 (dtd, *J* = 12.3, 8.3, 4.3 Hz, 1H), 1.94 (p, *J* = 6.5 Hz, 2H), 1.65 (dq, *J* = 11.7, 7.7 Hz, 1H), 1.29 (dd, *J* = 7.2, 2.4 Hz, 6H).

^{13}C NMR (126 MHz, CDCl$_3$): δ 162.37, 161.86, 159.89, δ 154.92 (d, *J* = 10.0 Hz), 143.67, 137.03, 127.94, 127.46 (d, *J* = 15.7 Hz), 127.09 (d, *J* = 7.3 Hz), 125.09, 118.36 (d, *J* = 3.6 Hz),, 99.97, 99.76, 77.91, 77.31, 77.06, 76.80, 68.22, 55.93, 43.49, 28.67, 26.90 (d, *J* = 1.4 Hz), 25.95, 22.75.

^{19}F NMR (376 MHz, CDCl$_3$): δ -115.59 (dd, *J* = 11.8, 8.4 Hz).

HRMS: *m/z* calcd for $C_{20}H_{24}FNO_3SNa$; 400.1358 [M+23]: found 400.1348.

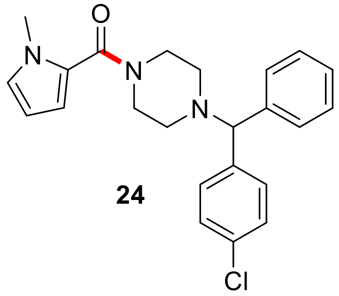

24

(4-((4-Chlorophenyl) (phenyl) methyl) piperazin-1-yl) (1-methyl-1H-pyrrol-2-yl) methanone (24): Compound **24** was obtained using General Procedure C on a 0.25 mmol scale. The crude

product was purified via General Purification Method 2, followed by flash chromatography (eluent: gradient 0% EtOAc/100% hexanes to 30% EtOAc/70% hexanes) to provide the desired compound as a white solid (71 mg, 72%). **R_f** = 0.57 (50% EtOAc/50% hexanes).

^{1}H NMR (500 MHz, $CDCl_3$): δ 7.40 (dt, *J* = 8.6, 4.6 Hz, 4H), 7.35 – 7.23 (m, 5H), 6.69 (s, 1H), 6.30 (qd, *J* = 3.5, 1.6 Hz, 1H), 6.07 (dq, *J* = 6.4, 2.7 Hz, 1H), 4.27 (d, *J* = 4.5 Hz, 1H), 3.79 (t, *J* = 4.0 Hz, 7H), 2.44 (q, *J* = 4.4 Hz, 4H).

^{13}C NMR (126 MHz, $CDCl_3$): δ 162.81, 141.65, 140.85, 132.82, 129.17, 128.83, 128.76, 127.83, 127.42, 126.21, 125.01, 112.87, 106.81, 75.33, 52.10, 45.37, 35.71.

HRMS: *m/z* calcd for $C_{23}H_{25}ClN_3O$; 394.168615 [M+1]: found 394.1685.

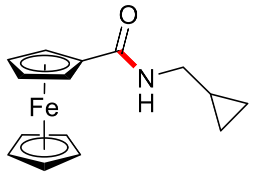

25

***N*-(Cyclopropylmethyl)feroocenamide (25):** Compound **25** was obtained using General Procedure C on a 0.25 mmol scale. The crude product was purified via General Purification Method 2, followed by flash chromatography (eluent: gradient 10% EtOAc/90% hexanes to 30% EtOAc/70% hexanes) to provide the desired compound as an orange solid (65 mg, 97%). **R_f** =0.45 (50% EtOAc/50% hexanes).

^{1}H NMR (500 MHz, $CDCl_3$): δ 5.83 (s, 1H), 4.70 (s, 2H), 4.41 – 4.17 (m, 5H), 3.26 (s, 2H), 1.39 – 0.88 (m, 2H), 0.73 – 0.08 (m, 4H).

^{13}C NMR (126 MHz, $CDCl_3$): δ 170.11, 77.29, 77.04, 76.79, 76.37, 70.34, 69.76, 68.10, 44.33, 11.14, 3.46.

HRMS: *m/z* calcd for $C_{15}H_{17}FeNOH^+$: 284.0738 [M+1]; found 284.0748.

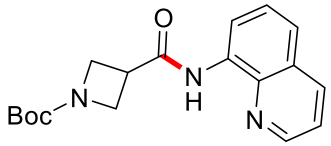

26

***t*-Butyl 3-(quinolin-8-ylcarbamoyl)azetidine-1-carboxylate (26):** Compound **26** was obtained using General Procedure C on a 0.25 mmol scale. The crude product was purified via General Purification Method 2, followed by flash chromatography (30% EtOAc/70% hexanes) to provide the desired compound as a yellow oil (44.8 mg, 55%). **R_f** = 0.54 (50% EtOAc/50% hexanes).

^{1}H NMR (500 MHz, $CDCl_3$): δ 9.87 (s, 1H), 8.86 – 8.76 (m, 2H), 8.18 (d, *J* = 8.3 Hz, 1H), 7.55 (d, *J* = 7.2 Hz, 2H), 7.47 (dd, *J* = 8.3, 4.2 Hz, 1H), 4.36 – 4.29 (m, 2H), 4.21 (t, *J* = 8.6 Hz, 2H), 3.60 (td, *J* = 8.8, 4.4 Hz, 1H), 1.47 (s, 9H).

^{13}C NMR (126 MHz, $CDCl_3$): δ 170.15, 156.23, 148.29, 138.30, 136.47, 134.02, 127.93, 127.37, 122.00, 121.76, 116.75, 79.79, 77.33, 77.07, 76.82, 34.85, 28.40.

HRMS: *m/z* calcd for $C_{18}H_{21}N_3O_3$; 328.1661 [M+1]: found 328.1673.

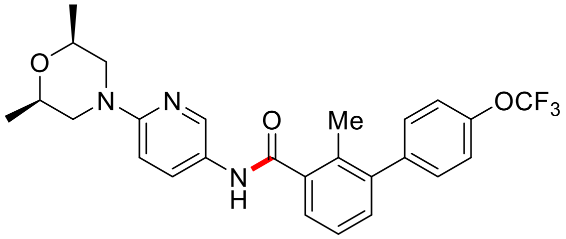

27

***N*-(6-((2S,6R)-2,6-Dimethylmorpholino)pyridin-3-yl)-2-methyl-4'-(trifluoromethoxy)-[1,1'-biphenyl]-3-carboxamide (27):** Compound **27** was prepared according to Method A as described above with modification of reaction time: thioester formation (4 h) and amide formation (4 h). The crude product was purified by flash column chromatography (10-30% EtOAc/hexanes) afforded **27** (114.0 mg, 94% yield) as light pink solid; **R_f** = 0.34 (30% EtOAc/70% hexanes).

^{1}H NMR (400 MHz, DMSO-*d*$_6$): δ 10.23 (s, 1H), 8.42 (d, *J* = 2.6 Hz, 1H), 7.92 (dd, *J* = 9.1, 2.7 Hz, 1H), 7.49 – 7.42 (m, 5H), 7.42 – 7.20 (m, 2H), 6.85 (d, *J* = 9.1 Hz, 1H), 4.12 – 3.95 (m, 2H), 3.60 (dqd, *J* = 12.4, 6.2, 2.3 Hz, 2H), 2.32 (dd, *J* = 12.7, 10.5 Hz, 2H), 2.20 (s, 3H), 1.14 (d, *J* = 6.2 Hz, 6H).

^{13}C NMR (101 MHz, $CDCl_3$): δ 168.68, 156.80, 148.47, 148.45, 148.43, 142.15, 140.25, 139.88, 137.64, 133.38, 131.55, 131.26, 130.90, 130.62, 126.08, 125.80, 125.77, 121.79, 120.71, 119.24, 116.68, 106.87, 71.55, 51.17, 18.98, 17.62.

^{19}F NMR (376 MHz, $CDCl_3$): δ -57.77. Spectral data matched those previously reported.[11]

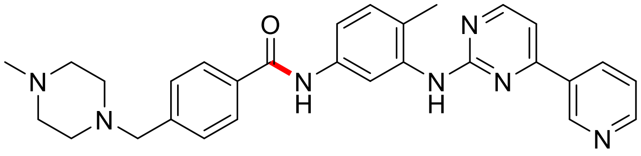

28

***N*-(4-Methyl-3-((4- (pyridin-3-yl) pyrimidin-2-yl) amino)phenyl)-4-((4-methylpiperazin-1-yl) methyl) benzamide (28):** Compound **28** was prepared according to Method A as described

above with modification of 2 M EtOAc and 10 mol % DMAP used for step 1. The thioester formation (4 h) and amide formation (3 h). The crude product was purified by washing with 1 M NaOH and centrifugation to remove supernatant to afford **27** (109.8 mg, 89% yield) as an off white solid; **R$_f$** = 0.36 (10% MeOH/90% DCM).

^{1}H NMR (400 MHz, DMSO-*d$_6$*): δ 10.14 (s, 1H), 9.26 (d, *J* = 2.3 Hz, 1H), 8.95 (s, 1H), 8.67 (d, *J* = 4.8 Hz, 1H), 8.57 – 8.39 (m, 2H), 8.06 (d, *J* = 2.2 Hz, 1H), 7.88 (d, *J* = 7.8 Hz, 2H), 7.57 – 7.33 (m, 5H), 7.18 (d, *J* = 8.3 Hz, 1H), 3.50 (s, 2H), 2.33 (br. S, 8H), 2.20 (s, 3H), 2.13 (s, 3H).

^{13}C NMR (101 MHz, DMSO-*d$_6$*): δ 165.73, 162.08, 161.66, 159.94, 151.85, 148.68, 142.59, 138.27, 137.69, 134.89, 134.24, 132.69, 130.50, 129.09, 128.05, 124.25, 117.68, 117.20, 107.98, 62.10, 55.18, 53.06, 46.22, 18.13. Spectral data matched those previously reported.[12]

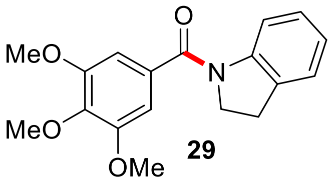

29

Indolin-1-yl(3,4,5-trimethoxyphenyl)methanone (29): Compound **29** was prepared according to Method A as described above with modification of reaction time: thioester formation (4 h) and amide formation (6 h). The crude product was purified by flash column chromatography (20% EtOAc/hexanes) afforded **29** (72.1 mg, 92% yield) as white solid; **R$_f$ =** 0.54 (50% EtOAc/50% hexanes).

^{1}H NMR (400 MHz, CDCl$_3$): δ 7.93 (brs, 1H), 7.21 (d, *J* = 7.4 Hz, 1H), 7.13 (d, *J* = 13.4 Hz, 1H), 7.01 (t, *J* = 7.4 Hz, 1H), 6.77 (s, 2H), 4.20 - 4.05 (m, 2H), 3.87 (d, *J* = 18.7 Hz, 9H), 3.12 (t, *J* = 8.2 Hz, 2H).

^{13}C NMR (101 MHz, CDCl$_3$): δ 168.69, 153.48, 142.62, 139.86, 132.55, 132.30, 127.34, 125.07, 124.05, 117.06, 104.63, 61.08, 56.39, 50.63, 28.13. Spectral data matched those previously reported.[13]

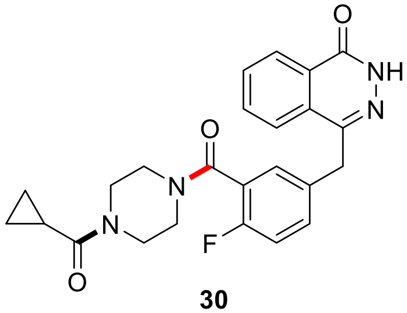

30

***N*-(4-Methyl-3-((4- (pyridin-3-yl) pyrimidin-2-yl) amino)phenyl)-4-((4-methylpiperazin-1-yl) methyl) benzamide (30):** Compound **30** was prepared according to Method B as described above with modification of 2 M EtOAc and 10 mol % DMAP used for step 1 and reaction time.

The thioester formation (6 h) and amide formation (6 h). The crude product was purified by flash column chromatography (1-5% MeOH in CH_2Cl_2) afforded **30** (93.4 mg, 86% yield) as light pink solid. **R_f =** 0.30 (5% MeOH/95 % DCM).

^{1}H NMR (400 MHz, $CDCl_3$): δ 10.25 (s, 1H), 8.52 – 8.35 (m, 1H), 7.86 – 7.61 (m, 3H), 7.43 – 7.28 (m, 2H), 7.05 (t, *J* = 8.9 Hz, 1H), 4.28 (s, 2H), 3.69 (d, *J* = 65.8 Hz, 6H), 3.32 (d, *J* = 19.7 Hz, 2H), 1.75 (s, 1H), 1.07 – 0.97 (m, 2H), 0.80 (s, 2H).

^{13}C NMR (126 MHz, $CDCl_3$): δ 172.37, 165.26, 161.05, 157.0 (d, *J* = 248.0 Hz), 145.52, 134.59 (d, *J* = 3 Hz),133.63, 131.70 (d, *J* = 8.8 Hz), 131.56, 129.55,129.17, 128.25, 127.12, 125.01, 123.68, (d, *J* = 17.4 Hz), 116.17 (d, *J* = 21.4 Hz), 47.14-46.81 (m), 45.66-45.15, 42.27, 42.02, 37.69, 11.04, 7.71.

^{19}F NMR (376 MHz, $CDCl_3$): δ -117.61. Spectral data matched those previously reported.[14]

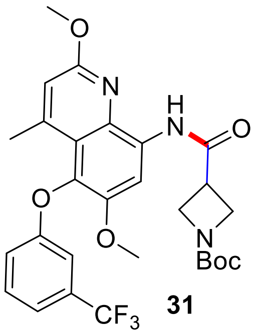

31

Dimethoxy-4-methyl-5-(3-(trifluoromethyl)phenoxy)quinolin-8-yl)carbamoyl)azetidine-1-carboxylate (31): Compound **31** was prepared according to Method B as described above with modification of reaction time. The thioester formation (4 h) and amide formation (6 h). The crude product was purified by flash column chromatography (10-15% EtOAc in hexanes) afforded **31** (133.8 mg, 95% yield) as light pink solid; **R_f** = 0.45 (30% EtOAc/70% hexanes).

^{1}H NMR (400 MHz, $CDCl_3$): δ 9.84 (s, 1H), 8.77 (s, 1H), 7.36 (t, *J* = 8.0 Hz, 1H), 7.04 (t, *J* = 2.1 Hz, 1H), 6.93 (dd, *J* = 8.2, 2.6 Hz, 1H), 6.72 (d, *J* = 1.2 Hz, 1H), 4.37 – 4.16 (m, 4H), 4.03 (s, 3H), 3.84 (s, 3H), 3.50 (tt, *J* = 8.4, 5.9 Hz, 1H), 2.61 (d, *J* = 1.1 Hz, 3H), 1.46 (s, 9H).

^{13}C NMR (126 MHz, $CDCl_3$): δ 170.22, 160.20, 158.81, 156.32, 147.91, 146.92, 132.51, 132.43, 132.17, 131.91, 131.66, 131.31, 130.13, 123.75 (q, *J* = 272.1 Hz) 120.23, 118.49, 118.46, 118.43, 118.40, 118.16, 115.95, 111.99, 111.96, 111.92, 111.89, 105.62, 79.99, 56.61, 53.11, 52.12, 35.35, 28.35, 23.07.

^{19}F NMR (376 MHz, $CDCl_3$): δ -62.60.

HRMS: *m/z* calcd for $C_{28}H_{31}F_3N_3O_6$; 562.2165 [M+1]: found 562.2177.

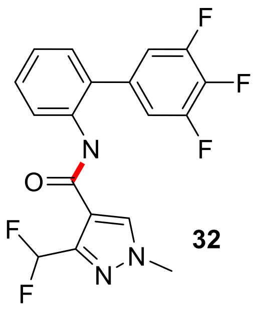

32

3-(Difluoromethyl)-1-methyl-N-(3',4',5'-trifluoro-[1,1'-biphenyl]-2-yl)-1H-pyrazole-4-carboxamide (32): Compound **32** was prepared according to Method B as described above with modification of 2 M EtOAc and 10 mol % DMAP used for step 1 and reaction time. The thioester formation (4 h) and amide formation (14 h). The crude product was purified by flash column chromatography (10-20% ethyl acetate in hexanes) afforded **32** (85.7 mg, 90% yield) as white solid; $\mathbf{R_f}$ = 0.55 (50% EtOAc/50% hexanes).

^{1}H NMR (400 MHz, $CDCl_3$-*d_6*): δ 8.20 (d, *J* = 8.2 Hz, 1H), 7.96 (s, 1H), 7.80 (s, 1H), 7.43 (ddd, *J* = 8.5, 6.0, 3.1 Hz, 1H), 7.25 – 7.19 (m, 2H), 7.09 – 6.90 (m, 2H), 6.64 (t, *J* = 54.2 Hz, 1H), 3.92 (d, *J* = 1.0 Hz, 3H).

^{13}C NMR (126 MHz, DMSO-*d_6*): δ 160.91, 156.41, 151.51, 151.48, 151.44, 151.40, 150.56, 149.55, 149.52, 149.48, 149.44, 145.47, 145.28, 145.10, 139.58, 139.46, 139.34, 138.09, 137.60, 137.48, 137.36, 136.60, 136.56, 136.53, 136.49, 136.46, 136.43, 135.47, 134.72, 133.22, 130.69, 129.37, 128.50, 127.32, 126.10, 122.65, 116.23, 116.21, 116.18, 113.77, (dd, J = 4.7 Hz, 16.6 Hz), 111.64 (t, *J* = 232.9 Hz), 39.83.

^{19}F NMR (376 MHz, $CDCl_3$): δ -109.45, -131.04 (d, $J_{F, F}$ = 21.3 Hz), -159.13 (t, $J_{F,F}$ = 21.4 Hz) ppm. Spectral data matched those previously reported.[15]

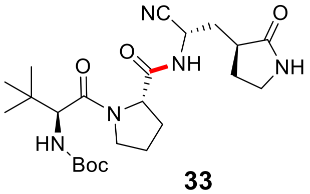

33

tert-butyl ((S)-1-((S)-2-(((S)-1-cyano-2-((S)-2-oxopyrrolidin-3-yl) ethyl) carbamoyl) pyrrolidin-1-yl)-3,3-dimethyl-1-oxobutan-2-yl) carbamate (33): Compound **33** was prepared with the following modifications to Method B: 10 mol % DMAP, 2 M EtOAc at rt for 12 h was used for step 1, and for step 2, reaction was stirred at rt for 16 h. The crude product was purified by flash column chromatography (2% MeOH in CH_2Cl_2) afforded **33** (104.3 mg, 90% yield) as white solid; $\mathbf{R_f}$ = 0.26 (5% MeOH /95% DCM).

^{1}H NMR (400 MHz, DMSO-d_6): δ 8.85 (d, *J* = 8.7 Hz, 1H), 7.63 (s, 1H), 6.59 (d, *J* = 9.0 Hz, 1H), 4.93 (ddd, *J* = 11.1, 8.6, 4.9 Hz, 1H), 4.24 – 4.07 (m, 2H), 3.70 (d, *J* = 9.8 Hz, 1H), 3.57 (dt, *J* = 9.8, 7.0 Hz, 1H), 3.17 – 2.89 (m, 2H), 2.25 – 2.01 (m, 3H), 1.96 (q, *J* = 6.4, 5.9 Hz, 1H), 1.90 – 1.59 (m, 4H), 1.35 (s, 9H), 0.91 (s, 9H).

^{13}C NMR (101 MHz, DMSO-d$_6$): δ 177.60, 171.79, 169.69, 155.59, 119.76, 78.03, 59.38, 58.39, 47.86, 37.58, 36.63, 34.72, 34.25, 29.15, 28.17, 26.94, 26.27, 24.97.

HRMS: *m/z* calcd for $C_{23}H_{37}N_5O_5Na$; 486.2692 [M+23]: found 486.2677.

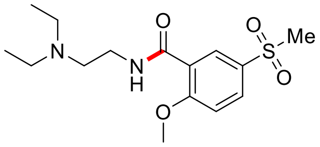

34

***N*-(2-Diethylaminoethyl)-2-methoxy-5-methylsulfonylbenzamide (34):** Compound **34** was prepared according to Method A as described above with modification of reaction time: thioester formation (5 h) and amide formation (4 h). The crude product was purified by flash column chromatography [(MeOH:NH_4OH:DCM) (3:1:96)] afforded **34** (71 mg, 86% yield) as yellow solid; **R$_f$** = 0.35 (10% MeOH/90% DCM).

^{1}H NMR (500 MHz, MeOD): δ 8.48 – 8.40 (m, 1H), 8.06 (dt, *J* = 8.9, 2.2 Hz, 1H), 7.38 (dt, *J* = 8.8, 1.7 Hz, 1H), 4.07 (d, *J* = 1.1 Hz, 3H), 3.52 (td, *J* = 6.8, 1.4 Hz, 2H), 3.11 (s, 3H), 2.71 (td, *J* = 6.8, 1.7 Hz, 2H), 2.64 (qd, *J* = 7.1, 1.7 Hz, 4H), 1.10 (td, *J* = 7.2, 1.4 Hz, 7H).

^{13}C NMR (126 MHz, MeOD): δ 166.10, 162.72, 134.24, 133.26, 131.84, 124.10, 113.92, 57.35, 52.32, 48.09, 44.59, 38.53, 11.89. Spectral data matched those previously reported.[16]

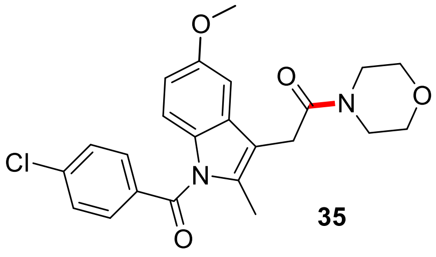

35

2-[1-(4-Chlorobenzoyl)-5-methoxy-2-methyl-1H-indol-3-yl]-1-(4-morpholinyl)ethanone (35): Compound (**34**) was prepared according to Method A as described above with modification of reaction time. The thioester formation (4 h) and amide formation (4 h.) The crude product was purified by flash column chromatography (10% EtOAc / hexanes) afforded **34** (86.5 mg, 81% yield) as white solid; **R$_f$** =0.48 (30% EtOAc/70 % hexanes).

^{1}H NMR (400 MHz, $CDCl_3$) δ 7.73 - 7.59 (m, 2H), 7.55 - 7.39 (m, 2H), 6.98 (d, *J* = 2.6 Hz, 1H), 6.81 (d, *J* = 9.0 Hz, 1H), 6.65 (dd, *J* = 9.0, 2.5 Hz, 1H), 3.82 (s, 3H), 3.69 (d, *J* = 21.3 Hz, 6H), 3.61 - 3.45 (m, 4H), 2.38 (s, 3H).

^{13}C NMR (126 MHz, $CDCl_3$) δ 168.96, 168.36, 156.14, 139.42, 135.34, 133.96, 131.30, 130.99, 130.95, 130.71, 129.25, 115.01, 113.14, 111.62, 101.64, 67.03, 66.61, 55.83, 46.47, 42.48, 30.23, 13.54. Spectral data matched those previously reported.[17]

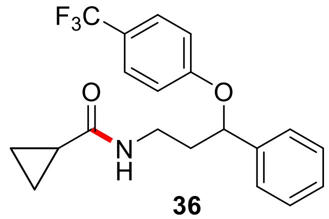

36

***N*-Methyl-*N*-(3-phenyl-3-(4-(trifluoromethyl) phenoxy) propyl) cyclopropane carboxamide (36):** Compound 35 was prepared according to Method B as described above with modification of reaction time and the addition of NMM as a base for step 2. Thioester formation (3 h) and amide formation (4 h). The crude product was purified by flash column chromatography (30% EtOAc/70% hexanes) afforded **36** (87.0 mg, 92% yield) as white solid; $\mathbf{R_f}$ = 0.27 (30% EtOAc/70% hexanes).

^{1}H NMR (500 MHz, $CDCl_3$): δ 7.45 (dd, *J* = 8.8, 4.4 Hz, 4H), 7.41 - 7.30 (m, 8H), 6.90 (dd, *J* = 8.6, 6.6 Hz, 4H), 5.23 (dt, *J* = 8.4, 4.0 Hz, 2H), 3.87 (dt, *J* = 15.4, 7.8 Hz, 1H), 3.74 - 3.47 (m, 3H), 3.16 (s, 3H), 2.98 (s, 3H), 2.35 -2.18 (m, 3H), 2.18 - 2.08 (m, 1H), 1.77 - 1.62 (m, 3H), 1.02 - 0.84 (m, 4H), 0.83 - 0.61 (m, 3H), 0.52 - 0.28 (m, 1H).

^{13}C NMR (126 MHz, $CDCl_3$): δ 173.60, 173.49, 160.46, 160.17, 140.82, 140.28, 129.08, 128.87, 128.24, 127.97, 126.96, 126.93, 126.90, 126.87, 126.84, 126.81, 126.78, 125.81, 125.64, 125.54, 125.43, 123.61, 123.38, 123.35, 123.27, 123.25, 123.09, 122.99, 122.83, 122.73, 122.47, 115.81, 115.75, 78.54, 46.29, 45.63, 37.85, 36.48, 35.86, 34.22, 11.31, 10.81, 7.90, 7.72, 7.58, 7.55.

HRMS: *m/z* calcd for $C_{21}H_{22}F_3NO_2Na$; 400.150033 [M+23]: found 400.1512.

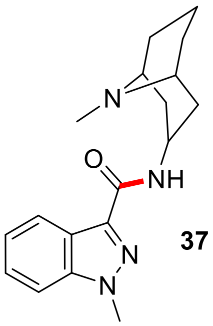

37

1-Methyl-*N*-((1*R*, 3*R*, 5*S*)-9-methyl-9-azabicyclo[3.3.1]nonan-3-yl)-1H-indazole-3-carboxamide (37): Compound **38** was prepared according to Method B as described above with modification of reaction time: thioester formation (4 h) and amide formation (6 h). The crude product was purified by flash column chromatography (0-10% MeOH in DCM) and afforded **37** (63 mg, 81% yield) as a white solid; **R_f =** 0.31 (10% MeOH/DCM).

^{1}H NMR (400 MHz, $CDCl_3$): δ 8.39 (dt, *J* = 8.1, 1.1 Hz, 1H), 7.45 – 7.36 (m, 2H), 7.30 – 7.26 (m, 1H), 6.79 (d, *J* = 8.4 Hz, 1H), 4.63 – 4.51 (m, 1H), 4.09 (s, 3H), 3.11 (d, *J* = 10.7 Hz, 2H), 2.59 – 2.50 (m, 2H), 2.53 (s, 3H), 2.06-1.98 (m, 3H), 1.45 – 1.35 (m, 2H), 1.08 (d, *J* = 10.7 Hz, 2H).

^{13}C NMR (101 MHz, $CDCl_3$): δ 161.89, 141.26, 137.59, 126.77, 123.03, 122.89, 122.42, 108.98, 77.41, 77.09, 76.77, 51.27, 40.76, 40.63, 35.90, 33.12, 24.79, 14.36. Spectral data matched those previously reported.[18]

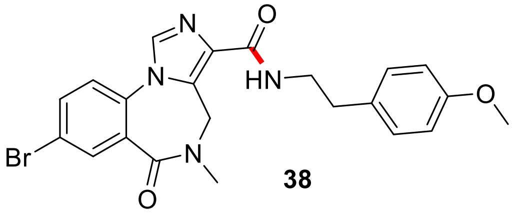

38

8-Bromo-*N*-(4-methoxyphenethyl)-5-methyl-6-oxo-5,6-dihydro-4H-benzo[f]imidazo [1,5-a][1,4] diazepine-3-carboxamide (38): Compound **36** was prepared according to Method A as described above with modification of reaction time: thioester formation (4 h) and amide formation (3 h). The crude product was purified by flash column chromatography (1-3% MeOH in DCM) afforded **38** (105.3 mg, 90% yield) as white solid; **R_f** = 0.3 (5% MeOH/95% DCM).

^{1}H NMR (400 MHz, DMSO-*d_6*): δ 8.34 (s, 1H), 8.20 (t, *J* = 6.0 Hz, 1H), 7.99 (d, *J* = 2.4 Hz, 1H), 7.94 (dd, *J* = 8.6, 2.4 Hz, 1H), 7.70 (d, *J* = 8.6 Hz, 1H), 7.20 – 7.08 (m, 2H), 6.91 – 6.75 (m, 2H), 5.16 (s, 1H), 4.47 (s, 1H), 3.71 (s, 3H), 3.46 (q, *J* = 6.9 Hz, 2H), 3.06 (s, 3H), 2.77 (t, *J* = 7.4 Hz, 2H).

^{13}C NMR (101 MHz, DMSO-*d*$_6$): δ 164.56, 162.01, 157.65, 135.22, 135.12, 134.08, 131.84, 131.28, 131.26, 130.67, 130.33, 129.56, 124.94, 120.44, 113.77, 54.95, 41.38, 40.01, 35.32, 34.39.

HRMS: *m/z* calcd for $C_{22}H_{22}BrN_4O_3$; 469.0875 [M+1]: found 469.0851.

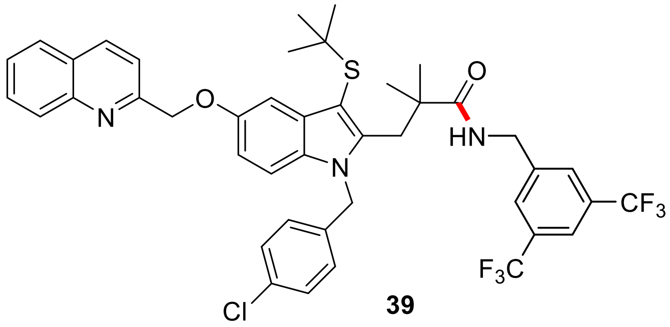

39

***N*-(3,5-*bis*(Trifluoromethyl)benzyl)-3-(3-(*t*-butylthio)-1-(4-chlorobenzyl)-5-(quinolin-2-ylmethoxy)-1H-indol-2-yl)-2,2-dimethylpropanamide (39):** Compound **39** was prepared according to Method B as described above with modification of 2 M EtOAc used for step 1 and reaction time: thioester formation (6 h) and amide formation (4 h). The crude product was purified by flash column chromatography (10% EtOAc/hexanes) afforded **39** (178.3 mg, 88% yield) as white solid; $\mathbf{R_f}$ = 0.70 (30% EtOAc/70% hexanes).

^{1}H NMR (400 MHz, DMSO-*d*$_6$): δ 8.42 – 8.31 (m, 2H), 8.08 – 8.00 (m, 1H), 7.96 (dd, *J* = 8.1, 1.5 Hz, 2H), 7.90 (d, *J* = 1.7 Hz, 2H), 7.78 (ddd, *J* = 8.4, 6.8, 1.5 Hz, 1H), 7.68 – 7.57 (m, 2H), 7.29 (d, *J* = 6.5 Hz, 2H), 7.21 (d, *J* = 8.8 Hz, 1H), 7.13 (d, *J* = 2.5 Hz, 1H), 6.87 (dd, *J* = 8.8, 2.5 Hz, 1H), 6.82 – 6.73 (m, 2H), 5.36 (d, *J* = 15.2 Hz, 4H), 4.42 (d, *J* = 5.8 Hz, 2H), 3.15 (s, 2H), 1.10 (s, 6H), 0.96 (s, 9H).

^{13}C NMR (126 MHz, $CDCl_3$): δ 177.90, 158.69, 153.88, 147.72, 147.65, 143.70, 141.06, 137.20, 137.00, 136.31, 133.20, 132.63, 132.50, 132.25, 132.08 (q, $J_{C\text{-}F}$ = 31.0 Hz), 129.83, 129.05, 129.00, 127.98, 127.95, 127.92, 127.79, 127.72, 127.14, 126.52, 124.37, 122.20, 121.64, 121.61, 121.58, 121.55, 121.52, 120.03, 119.35, 113.18, 112.62, 111.09, 105.52, 105.26, 103.93, 77.41, 77.16, 76.91, 71.89, 48.65, 48.34, 48.27, 47.37, 47.19, 44.47, 44.33, 43.05, 34.66, 31.25, 26.07.

^{19}F NMR (376 MHz, DMSO-d$_6$): δ -61.37.

HRMS: *m/z* calcd for $C_{43}H_{41}ClF_6N_3O_2S$; [M+1]: 812.2512 found 812.2501.

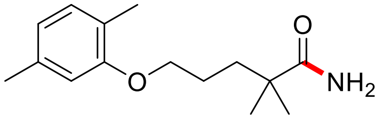

40

5-(2,5-Dimethylphenoxy)-2,2-dimethylpentanamide (40): Compound **40** was obtained using General Procedure A on a 0.25 mmol scale. The crude product was purified via General Purification Method 1-C, as described in Section 2.2.1, to provide the desired compound as a white solid (50.5 mg, 81%). $\mathbf{R_f}$ = 0.50 (50% EtOAc/50% hexanes).

^{1}H NMR (500 MHz, DMSO-*d*$_6$): δ 7.04 (s, 1H), 6.98 (d, *J* = 7.4 Hz, 1H), 6.79 (s, 1H), 6.70 (s, 1H), 6.62 (d, *J* = 7.4 Hz, 1H), 3.89 (t, *J* = 6.1 Hz, 2H), 2.25 (s, 3H), 2.09 (s, 3H), 1.63 (qd, *J* = 6.7, 3.2 Hz, 2H), 1.58 (dt, *J* = 11.9, 3.4 Hz, 2H), 1.09 (s, 6H).

^{13}C NMR (126 MHz, DMSO-*d*$_6$): δ 179.41, 157.00, 136.50, 130.50, 122.95, 120.94, 112.52, 68.25, 41.50, 37.34, 25.85, 25.19, 21.52, 16.04. Spectral data matched those previously reported.[19]

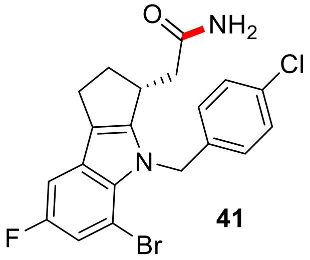

41

(*R*)-2-(5-Bromo-4-(4-chlorobenzyl)-7-fluoro-1,2,3,4-tetrahydrocyclopenta[b]indol-3-yl)acetamide (41): Compound **41** was prepared according to Method A as described above with modification of reaction time: thioester formation (4 h) and amide formation (0.5 h). The crude product was purified by 1 M NaOH wash and centrifugation to afford **41** (104.5 mg, 96% yield) as white solid; $\mathbf{R_f}$ **=** 0.49 (5% MeOH/95% DCM).

^{1}H NMR (400 MHz, DMSO-*d*$_6$): δ 7.36 (dd, *J* = 8.6, 6.6 Hz, 3H), 7.25 (dd, *J* = 9.0, 2.5 Hz, 1H), 7.14 (dd, *J* = 9.1, 2.5 Hz, 1H), 6.84 (dd, *J* = 11.0, 9.0 Hz, 3H), 5.81 (d, *J* = 17.8 Hz, 1H), 5.60 (d, *J* = 17.7 Hz, 1H), 3.49 (s, 1H), 2.90 - 2.54 (m, 4H), 2.33 (dd, *J* = 14.1, 4.3 Hz, 1H), 2.22 - 1.96 (m, 2H).

^{13}C NMR (101 MHz, DMSO-*d*$_6$): δ 172.48, 157.42, 155.06, 152.75, 138.57, 133.45, 131.56, 128.62, 127.07, 127.01, 126.91, 118.77, 118.72, 113.44, 113.15, 103.61, 103.39, 102.84, 102.72, 48.05, 40.20, 40.15, 39.99, 39.94, 39.78, 39.73, 39.57, 39.52, 39.31, 39.10, 38.89, 35.36, 34.76, 22.51.

^{19}F NMR (376 MHz, DMSO-*d*$_6$): δ -123.16.

HRMS: *m/z* calcd for $C_{20}H_{17}BrClFN_2ONa$; 457.0095 [M+23]: found 457.0109

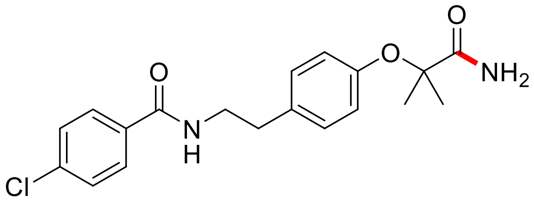

42

***N*-(4-((1-Amino-2-methyl-1-oxopropan-2-yl) oxy)phenethyl)-4-chlorobenzamide (42):** Compound **42** was obtained using General Procedure A on a 0.25 mmol scale. The crude product was purified via General Purification Method 1-C, as described in Section 2.2.1, to provide the desired compound as a white solid (72 mg, 80%). $\mathbf{R_f}$ = 0.33 (50% EtOAc/50% hexanes).

^{1}H NMR (500 MHz, DMSO-*d*$_6$): δ 8.72 (t, *J* = 5.5 Hz, 1H), 7.85 (d, *J* = 8.3 Hz, 2H), 7.53 (d, *J* = 8.2 Hz, 2H), 7.49 (s, 1H), 7.25 (s, 1H), 7.14 (d, *J* = 8.1 Hz, 2H), 6.83 (d, *J* = 8.1 Hz, 2H), 2.78 (t, *J* = 7.5 Hz, 2H), 1.64 (s, 2H), 1.39 (s, 6H).

^{13}C NMR (126 MHz, DMSO-*d*$_6$): δ 176.42, 165.54, 153.73, 136.33, 133.80, 133.43, 129.71, 129.54, 128.81, 120.19, 80.26, 41.49, 40.57, 40.48, 40.40, 40.31, 40.23, 40.14, 40.06, 39.97, 39.89, 39.81, 39.64, 39.47, 34.67, 25.38. Spectral data matched those previously reported.[18]

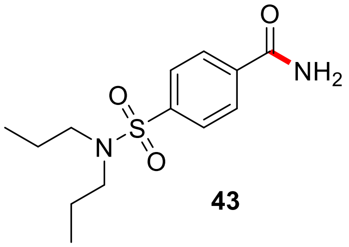

43

4-(*N*,*N*-Dipropylsulfamoyl)benzamide (43): Compound **43** was obtained using General Procedure A on a 0.25 mmol scale. The crude product was purified via General Purification Method 1-C, as described in Section 2.2.1, to provide the desired compound as a white solid (70.3 mg, 99%). $\mathbf{R_f}$ = 0.17 (50% EtOAc/50% hexanes).

^{1}H NMR (500 MHz, DMSO-*d*$_6$): δ 8.20 (s, 1H), 8.06 (d, *J* = 8.1 Hz, 2H), 7.87 (d, *J* = 7.9 Hz, 2H), 7.62 (s, 1H), 3.03 (d, *J* = 7.8 Hz, 4H), 1.47 (h, *J* = 7.4 Hz, 4H), 0.80 (t, *J* = 7.4 Hz, 6H).

^{13}C NMR (126 MHz, DMSO-*d*$_6$): δ 167.18, 142.22, 138.30, 128.90, 127.21, 50.09, 22.08, 11.42. Spectral data matched those previously reported.[19]

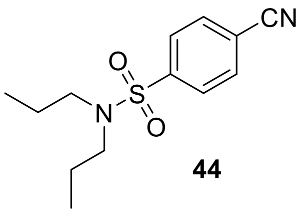

44

4-Cyano-*N*,*N*-dipropylbenzenesulfonamide (44): Compound **44** was obtained as described in section 7.1 on a 0.25 mmol scale. The crude product was purified via flash chromatography (30% EtOAc/70% hexanes) to provide the desired compound as a white solid (59.9 mg, 90%). $\mathbf{R_f}$ = 0.38 (20% EtOAc/80% hexanes).

^{1}H NMR (500 MHz, $CDCl_3$): δ 7.93 (d, *J* = 8.0 Hz, 2H), 7.81 (d, *J* = 8.0 Hz, 2H), 3.16 – 3.07 (m, 4H), 1.64 – 1.49 (m, 4H), 0.88 (t, *J* = 7.5 Hz, 6H).

^{13}C NMR (126 MHz, $CDCl_3$): δ 144.63, 132.88, 127.61, 117.43, 115.95, 49.93, 21.94, 11.12. Spectral data matched those previously reported.[20]

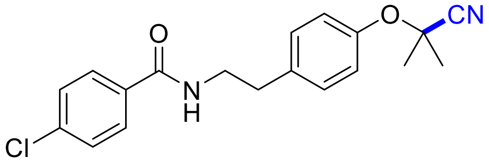

45

4-Chloro-*N*-(4-((2-cyanopropan-2-yl)oxy)phenethyl)benzamide (45): Compound **45** was prepared as described in section 4.2. $\mathbf{R_f}$ = 0.44 (20% EtOAc/80% hexanes).

^{1}H NMR (500 MHz, $CDCl_3$): δ 7.65 – 7.59 (m, 2H), 7.41 – 7.35 (m, 2H), 7.20 (d, *J* = 8.6 Hz, 2H), 7.17 – 7.10 (m, 2H), 6.10 (s, 1H), 3.69 (q, *J* = 6.7 Hz, 2H), 2.91 (t, *J* = 6.9 Hz, 2H), 1.71 (s, 6H).
^{13}C NMR (126 MHz, $CDCl_3$): δ 166.57, 137.85, 135.39, 133.06, 130.01, 129.01, 128.38, 122.33, 120.98, 77.41, 77.16, 76.91, 72.36, 41.32, 35.07, 27.66.

HRMS: *m/z* calcd for $C_{19}H_{20}ClN_2O_2$; [M+1]: 343.1213 found 343.1214, calc'd for $C_{19}H_{21}ClN_2O_2Na$ [M+23]: 365.1033, found 365.1032.

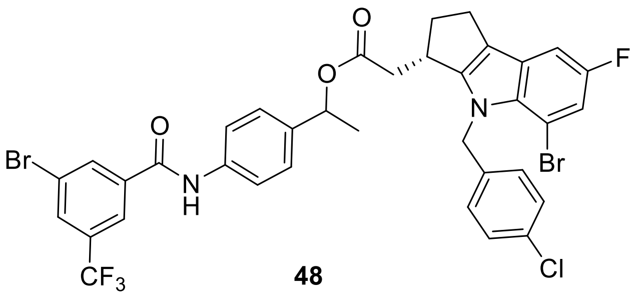

48

1-(4-(3-Bromo-5-(trifluoromethyl) benzamido) phenyl) ethyl 2-((S)-5-bromo-4-(4-chlorobenzyl)-7-fluoro-1,2,3,4-tetrahydrocyclopenta[b]indol-3-yl) acetate (48): Compound **48** was prepared as described in section 4.3.

^{1}H NMR (400 MHz, CDCl$_3$): δ 8.17 (s, 1H), 8.05 (d, *J* = 10.8 Hz, 2H), 7.92 (s, 1H), 7.55 (dd, *J* = 14.0, 8.2 Hz, 2H), 7.27 (s, 1H), 7.20 (dd, *J* = 10.9, 8.2 Hz, 2H), 7.04 (td, *J* = 8.1, 2.8 Hz, 2H), 6.78 (dd, *J* = 11.8, 8.1 Hz, 2H), 5.82 (tt, *J* = 7.1, 3.5 Hz, 1H), 5.72 (dd, *J* = 17.4, 13.8 Hz, 1H), 5.59 – 5.44 (m, 1H), 3.51 (dt, *J* = 10.3, 6.0 Hz, 1H), 2.91 – 2.63 (m, 3H), 2.51 (ddd, *J* = 15.4, 7.7, 4.2 Hz, 1H), 2.41 (ddd, *J* = 15.4, 9.2, 2.6 Hz, 1H), 2.21 (qd, *J* = 10.0, 3.8 Hz, 1H), 1.48 (dd, *J* = 12.8, 6.6 Hz, 3H), 1.26 (q, *J* = 6.8 Hz, 1H).

^{13}C NMR (126 MHz, CDCl$_3$): δ 171.36, 171.17 (d, *J* = 1.7 Hz), 162.98, 157.92, 156.02, 150.66 (d, *J* = 1.7 Hz), 138.15 (d, *J* = 8.3 Hz), 137.70 (d, *J* = 6.9 Hz), 137.46 (d, *J* = 2.4 Hz), 136.96 (d, *J* = 4.7 Hz), 134.15, 133.72, 133.01, 132.88 (q, *J* = 31.5 Hz), 131.67 – 131.40 (m), 128.91, 127.18 (d, *J* = 2.6 Hz), 127.04 (d, *J* = 8.0 Hz), 126.88, 123.37, 122.82 (q, *J* = 3.7 Hz), 122.72 (q, *J* = 273.4 Hz), 121.63, 120.59 (d, *J* = 8.2 Hz), 119.99 (d, *J* = 4.8 Hz), 114.45 (dd, *J* = 28.5, 5.1 Hz), 103.65 (d, *J* = 22.5 Hz), 103.21 (dd, *J* = 11.9, 2.1 Hz), 72.37 (d, *J* = 4.5 Hz), 60.49, 53.46, 48.58 (d, *J* = 3.1 Hz), 39.35, 35.49 (dd, *J* = 16.2, 2.6 Hz), 29.72, 22.90 (d, *J* = 2.9 Hz), 21.91 (d, *J* = 5.2 Hz), 21.09, 14.20.

^{19}F NMR (471 MHz, CDCl$_3$): δ -62.79, -123.29 (dt, *J* = 28.8, 8.7 Hz).

HRMS: *m/z* calcd for $C_{36}H_{28}Br_2ClF_4N_2O_3$; 805.0091 [M+1]: found 805.0168.

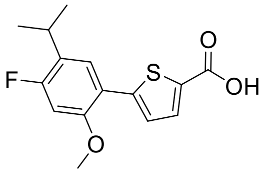

5-(4-Fluoro-5-isopropyl-2-methoxyphenyl)thiophene-2-carboxylic acid: This compound was prepared as described in section 3.1. **R$_f$ =** 0.05 (1:1 EtOAc/hexanes).

^{1}H NMR (500 MHz, DMSO-*d$_6$*): δ 7.70 (d, *J* = 8.5 Hz, 1H), 7.65 (dd, *J* = 17.5, 3.9 Hz, 2H), 7.05 (d, *J* = 12.5 Hz, 1H), 3.93 (s, 3H), 3.14 (p, *J* = 7.0 Hz, 1H), 1.25 (d, *J* = 6.9 Hz, 6H).

^{13}C NMR (126 MHz, DMSO-d_6): δ 163.35, 161.34, 159.38, 154.68 (d, J = 10.5 Hz), 144.07, 132.34, 126.91 (d, J = 15.6 Hz), 126.67 (d, J = 7.3 Hz), 125.55, 117.85, 117.83, 100.61 (d, J = 27.5 Hz), 56.35, 40.11, 40.02, 39.94, 39.85, 39.78, 39.69, 39.61, 39.52, 39.35, 39.19, 39.02, 26.53, 22.54.

^{19}F NMR (376 MHz, DMSO-d_6): δ -115.55.

HRMS: *m/z* calcd for $C_{15}H_{15}FO_3SNa$; 317.0623 [M+23]: found 317.060.

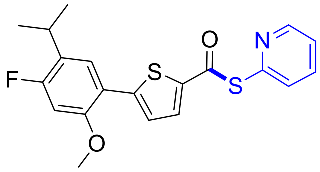

S-(Pyridin-2-yl) 5-(4-fluoro-5-isopropyl-2-methoxyphenyl)thiophene-2-carbothioate : This compound was obtained using the General Procedure, Method A, on a 0.25 mmol scale. The crude product was purified via silica gel column chromatography (eluent: 30% EtOAc/70% hexanes) to provide the desired compound as a pale-yellow solid (101 mg, 89% yield). R_f = 0.46 (1:1 EtOAc/hexanes). **R_f =** 0.55 (50% EtOAc/50% hexanes).
^{1}H NMR (400 MHz, CDCl$_3$): δ 8.65 (d, J = 4.8 Hz, 1H), 7.88 (d, J = 4.1 Hz, 1H), 7.85 – 7.74 (m, 2H), 7.52 (d, J = 8.4 Hz, 1H), 7.46 (d, J = 4.2 Hz, 1H), 7.32 (h, J = 3.8 Hz, 1H), 6.69 (d, J = 11.9 Hz, 1H), 3.92 (s, 3H), 3.19 (hept, J = 6.9 Hz, 1H), 1.28 (d, J = 7.0 Hz, 6H).

^{13}C NMR (126 MHz, CDCl$_3$): δ 180.84, 162.42, 160.43, 155.23 (d, J = 10.1 Hz), 151.50, 150.38, 147.99, 139.19, 137.13, 131.96, 130.60, 127.74 (d, J = 15.6 Hz), 127.19 (d, J = 7.4 Hz), 125.36, 123.54, 117.87, 99.99 (d, J = 27.6 Hz), 77.29, 77.04, 76.79, 56.00, 26.99, 22.73.

^{19}F NMR (376 MHz, CDCl$_3$): δ -114.06 (t, J = 10.2 Hz).

HRMS: *m/z* calcd for $C_{20}H_{18}FNO_2S_2$; 388.084125 [M+1]: found 388.0818.

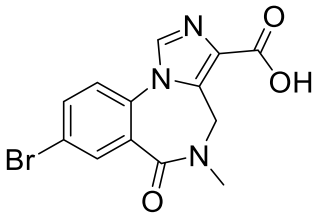

8-Bromo-5-methyl-6-oxo-5,6-dihydro-4H-benzo[f] imidazo [1,5-a] [1,4] diazepine-3-carboxylic acid:

^{1}H NMR (500 MHz, DMSO-d_6): δ 12.84 (s, 1H), 8.36 (s, 1H), 8.00 (d, J = 2.4 Hz, 1H), 7.95 (dd, J = 8.6, 2.4 Hz, 1H), 7.72 (d, J = 8.6 Hz, 1H), 5.01 (s, 1H), 4.52 (s, 1H).

^{13}C NMR (126 MHz, DMSO-d_6): δ 164.91, 164.28, 136.64, 135.74, 135.53, 134.47, 131.77, 130.81, 128.99, 125.57, 121.10, 42.29, 40.59, 40.49, 40.42, 40.33, 40.25, 40.16, 40.08, 39.99, 39.92, 39.83, 39.66, 39.49, 35.64.

HRMS: *m/z* calcd for $C_{13}H_{11}BrN_3O_3$; [M+1]: 335.9984 found 335.9946.

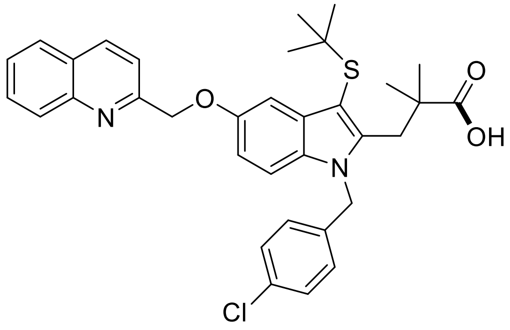

3-(3-(*t*-Butylthio)-1-(4-chlorobenzyl)-5-(quinolin-2-ylmethoxy)-1H-indol-2-yl)-2,2-dimethylpropanoic acid:

^{1}H NMR (500 MHz, DMSO-d_6): δ 8.45 (d, *J* = 8.5 Hz, 1H), 8.10 (d, *J* = 8.5 Hz, 1H), 8.02 (s, 1H), 7.83 (t, *J* = 7.7 Hz, 1H), 7.72 (d, *J* = 8.5 Hz, 1H), 7.64 (t, *J* = 7.5 Hz, 1H), 7.32 (dd, *J* = 8.5, 5.7 Hz, 3H), 7.16 (d, *J* = 2.6 Hz, 1H), 6.91 (dd, *J* = 8.8, 2.5 Hz, 1H), 6.83 (d, *J* = 8.2 Hz, 2H), 5.46 (d, *J* = 15.9 Hz, 4H), 3.18 (s, 2H), 2.08 (s, 1H), 1.09 (s, 6H), 0.99 (s, 9H).

^{13}C NMR (101 MHz, DMSO-d_6): δ 178.95, 158.64, 153.56, 146.51, 144.34, 138.48, 137.57, 132.31, 132.24, 130.79, 129.05, 128.52, 128.08, 127.75, 127.28, 120.00, 112.84, 112.28, 104.37, 103.63, 71.30, 49.08, 48.20, 47.08, 43.60, 40.63, 40.42, 40.21, 40.00, 39.79, 39.59, 39.38, 33.73, 31.20, 25.56, 1.64.

HRMS: *m/z* calcd for $C_{34}H_{35}ClN_2O_3S$; 587.21352 [M+1]: found 587.2141.

12. References

1. A. Wood, J. Kincaid, B. H. Lipshutz, *Green Chem.* **2022**, *24*, 2853-2858.
2. C. Lescot, D. U. Nielsen, I. S. Makarov, A. T. Lindhardt, K. Daasbjerg, T. Skrydstrup, *J. Am. Chem. Soc.* **2014**, *136*, 6142−6147.
3. Choi, M.; Jo, H.; Park, H.-J.; Kumar, A. S.; Lee, J.; Yun, J.; Kim, Y.; Han, S.; Jung, J.-K.; Cho, J.; Lee. K.; Kwak, J.-H. Lee, H. Bioorg. Med. Chem. Lett. 2015, 25, 2545-2549.4.
4. C. Pan, L. Wang, J. Han, *Adv. Synth. Catal.* **2021**, *364*, 268-273.
5. P. V. Ramachandran, H. J. Hamann, *Org. Lett.* **2021**, *23*, 8, 2938-2942.
6. C. Chen, L. Ling, M. Luo, X. Zeng, *Bull. Chem. Soc. Jpn.* **2021**, *94*, 762-766.
7. R. Zhu, Z. Xu, W. Ding, S. Liu, X. Shi, X. Lu, *Chin. J. Chem.* **2014,** *32*, 1039-1048.

8. L. Wang, X. Zhou, M. Xiao, N. Jiang, F. Liu, W. Zhou, X. Wanga, Z. Zheng, S. Li, *Bioorg. Med. Chem. Lett.* **2014,** *24*, 3739-3743.
9. Z. Zhou, X. Zheng, J. Liu, J. Li, P. Wen, H. Wang, *Synlett*, **2017**, *28*, 999-1003.
10. H. Long, X. Hu, B. Wang, Q. Wang, R. Wang, S. Liu, F. Xiong, Z. Jiang, X-Qi. Zhang, W-C. Ye, H. Wang, *J. Med. Chem.* **2021**, *64*, 12089-12108.
11. B. S. Takale, R. R. Thakore, F. Y. Kongab, B. H. Lipshutz. *Green Chem.* **2019,** *21*, 6258-6262.
12. J. C. Vantourout, H. N. Miras, A. Isidro-Llobet, S. Sproules, A. J. B. Watson, *J. Am. Chem. Soc.* **2017,** *139*, 4769-4779.
13. C. W. Cheung, J. Ma, X. Hu, *J. Am. Chem. Soc.* **2018**, *140*, 6789–6792.
14. H. P. Collin, W. J. Reis, D. U. Nielsen, A. T. Lindhardt, M. S. Valle, R. P. Freitas, T. Skrydstrup, *Org. Lett.* **2019,** *21*, 5775–5778.
15. P. Orecchia, D. S. Petkova, R. Goetz, *Green Chem.*, **2021**, *23*, 8169-8180.
16. X. Xu, H. Feng, L. Huang, X. Liu, *J. Org. Chem.* **2018**, *83*, 7962-7969.
17. T. Liu, X. Zhang, Z. Peng, J. Zhao, *Green Chem.*, **2021**, *23*, 9916-9921.
18. Opie, C. R.; Noda, H.; Shibasaki, M.; Kumagai, N. *Chem. Eur. J.* **2019,** *25*, 4648-4653.
19. Miao, Y.-Q.; Kang, J.-X.; Ma, Y.-N.; Chen, X. *Green Chem.* **2021**, *23*, 3595-3599.
20. Wang, Z.; Wang, X.; Ura Y.; Nishihara, Y. *Org. Lett.* **2019**, *21*, 6779-6784.

13. NMR Spectra

1

^{1}H NMR (400 MHz, $CDCl_3$):

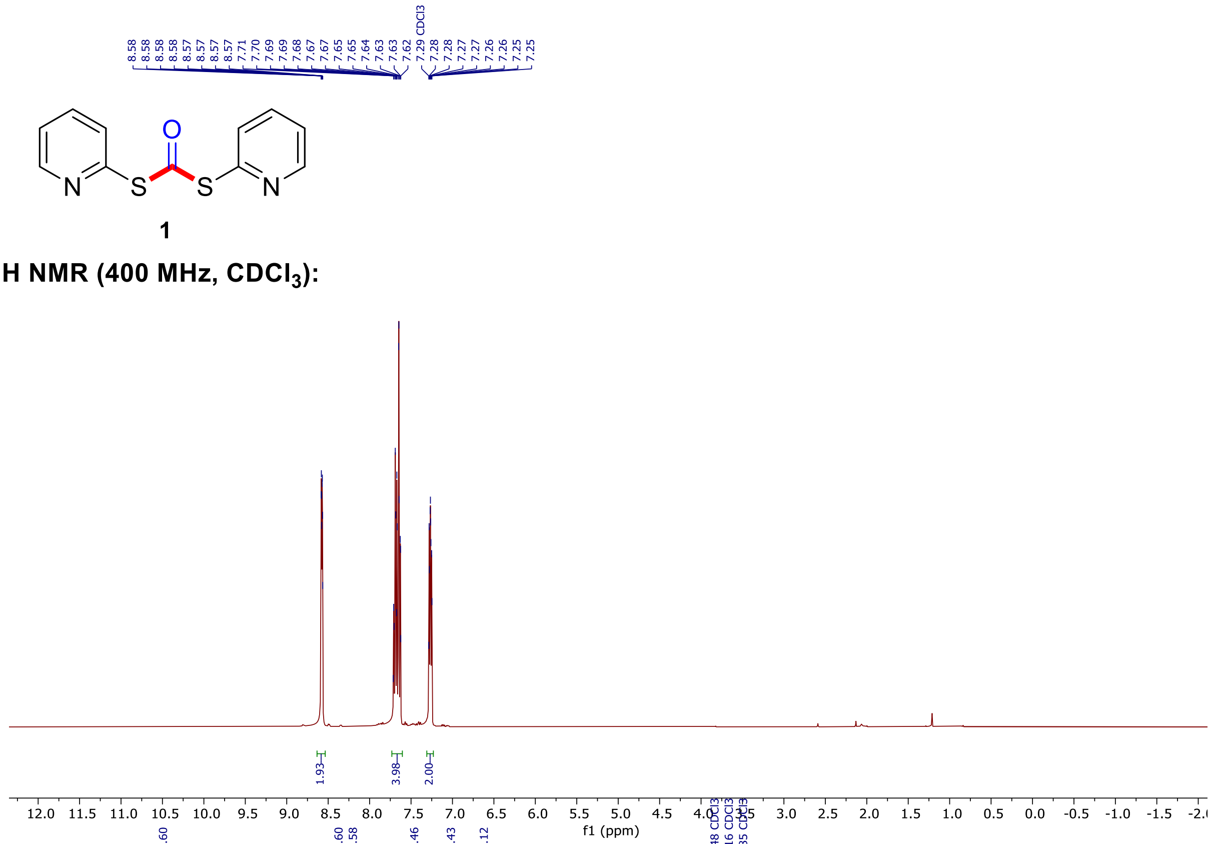

1

^{13}C NMR (101 MHz, $CDCl_3$):

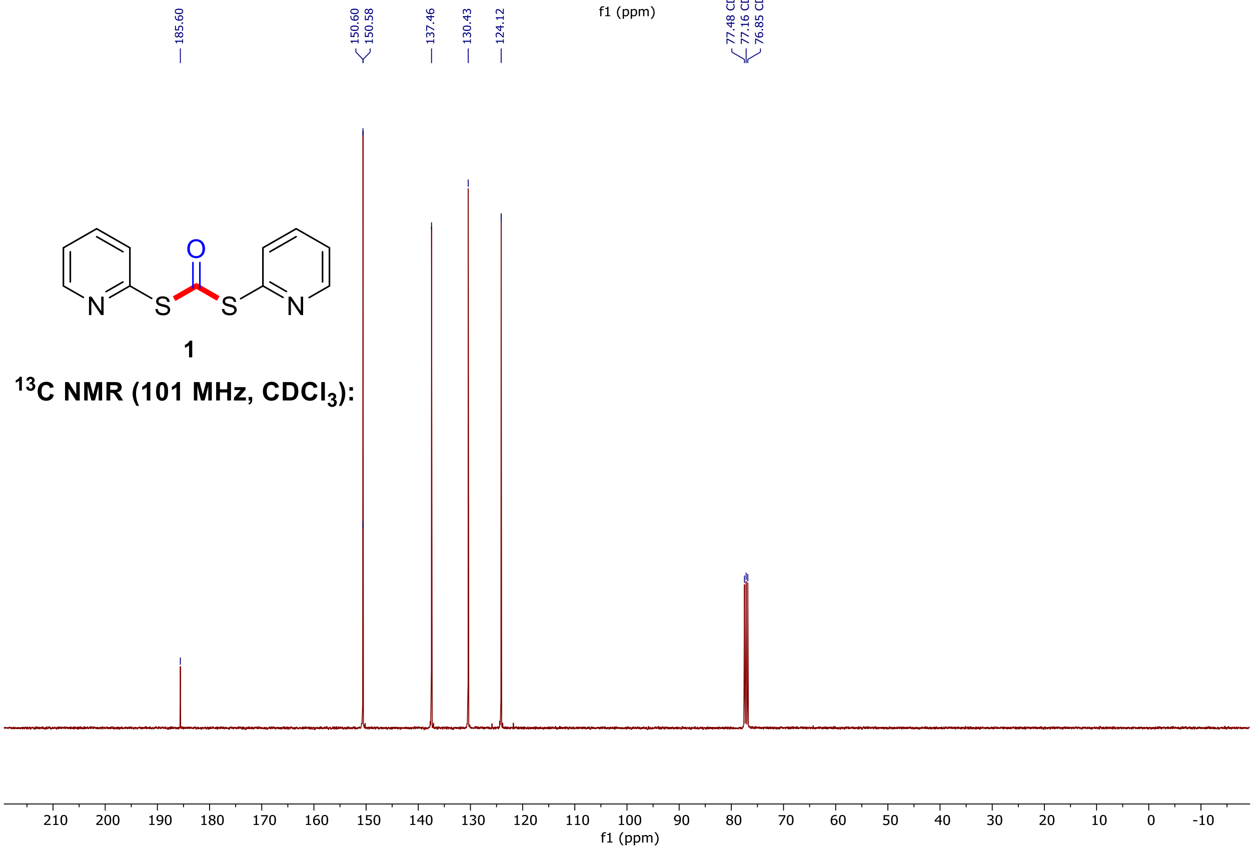

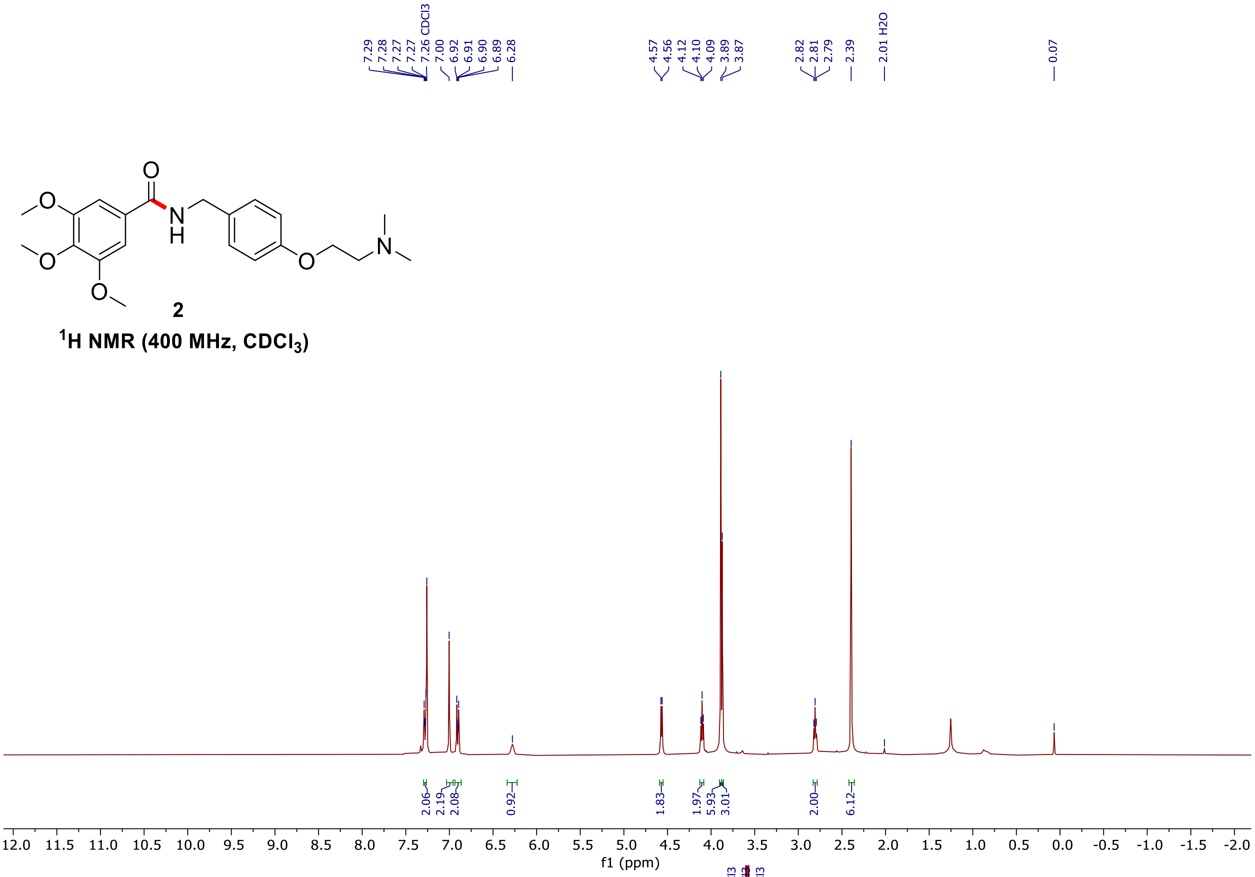

2

^{1}H NMR (400 MHz, CDCl$_3$)

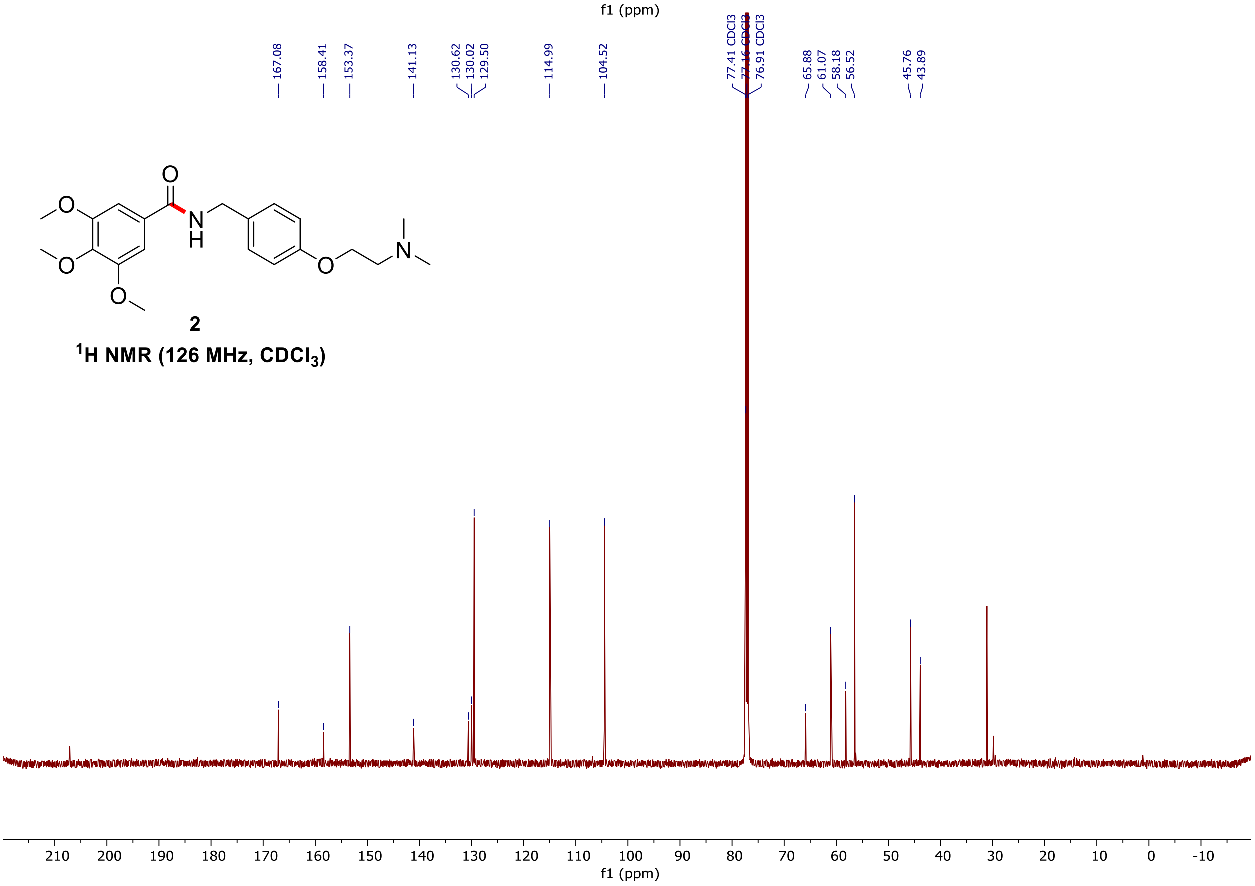

2

^{1}H NMR (126 MHz, CDCl$_3$)

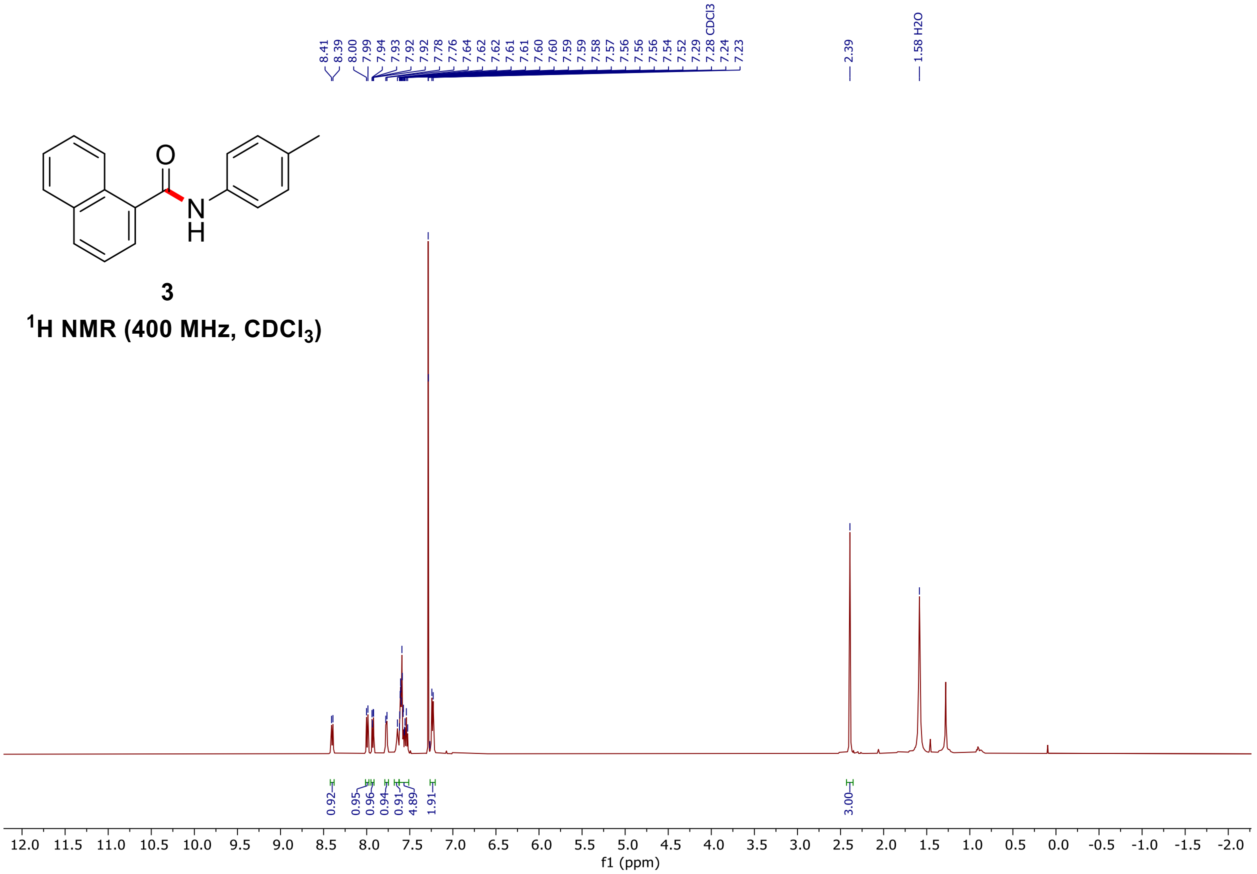
8.41
8.39
8.00
7.99
7.94
7.93
7.92
7.92
7.78
7.76
7.64
7.62
7.62
7.61
7.61
7.60
7.59
7.59
7.58
7.57
7.56
7.56
7.56
7.54
7.52
7.29
7.28 CDCl3
7.24
7.23
2.39
1.58 H2O
3
¹H NMR (400 MHz, CDCl₃)
0.92
0.95
0.96
0.94
0.91
4.89
1.91
3.00
f1 (ppm)

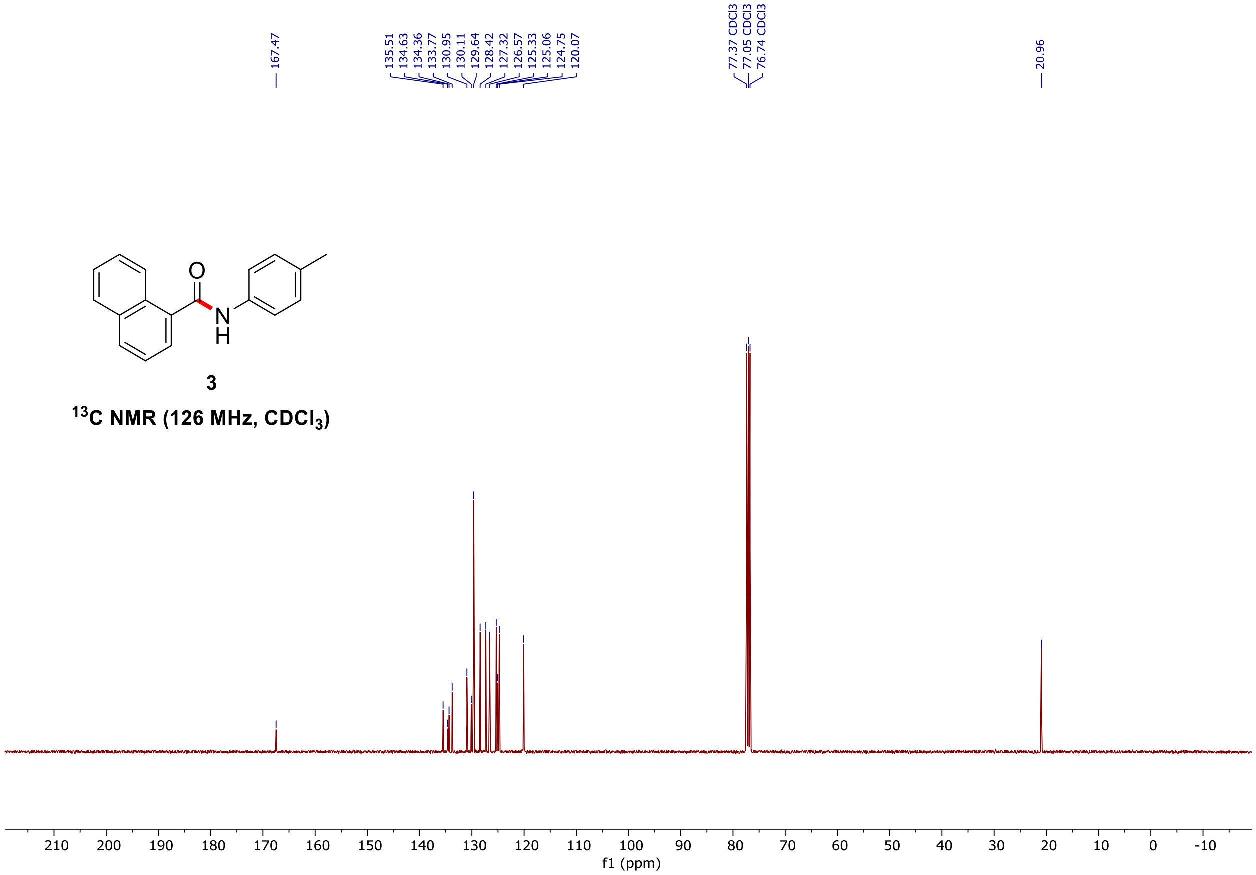
167.47
135.51
134.63
134.36
133.77
130.95
130.11
129.64
128.42
127.32
126.57
125.33
125.06
124.75
120.07
77.37 CDCl3
77.05 CDCl3
76.74 CDCl3
20.96
3
¹³C NMR (126 MHz, CDCl₃)
f1 (ppm)

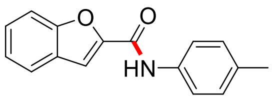

4

^{1}H NMR (400 MHz, $CDCl_3$)

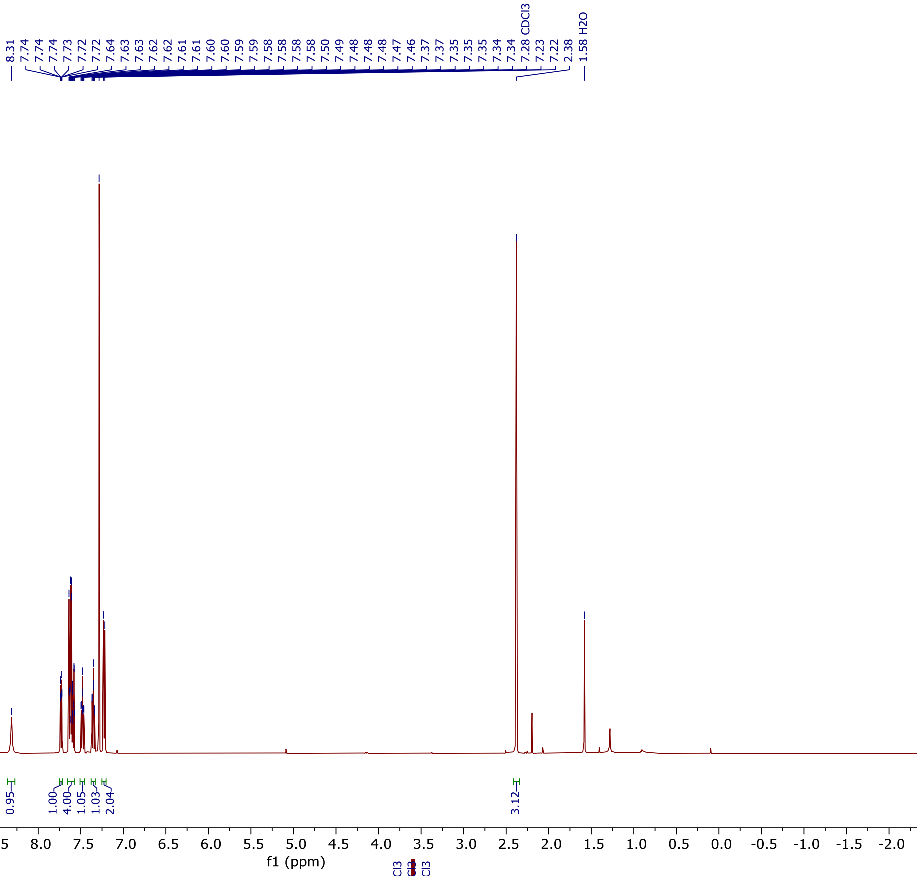

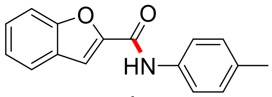

4

^{13}C NMR (101 MHz, $CDCl_3$)

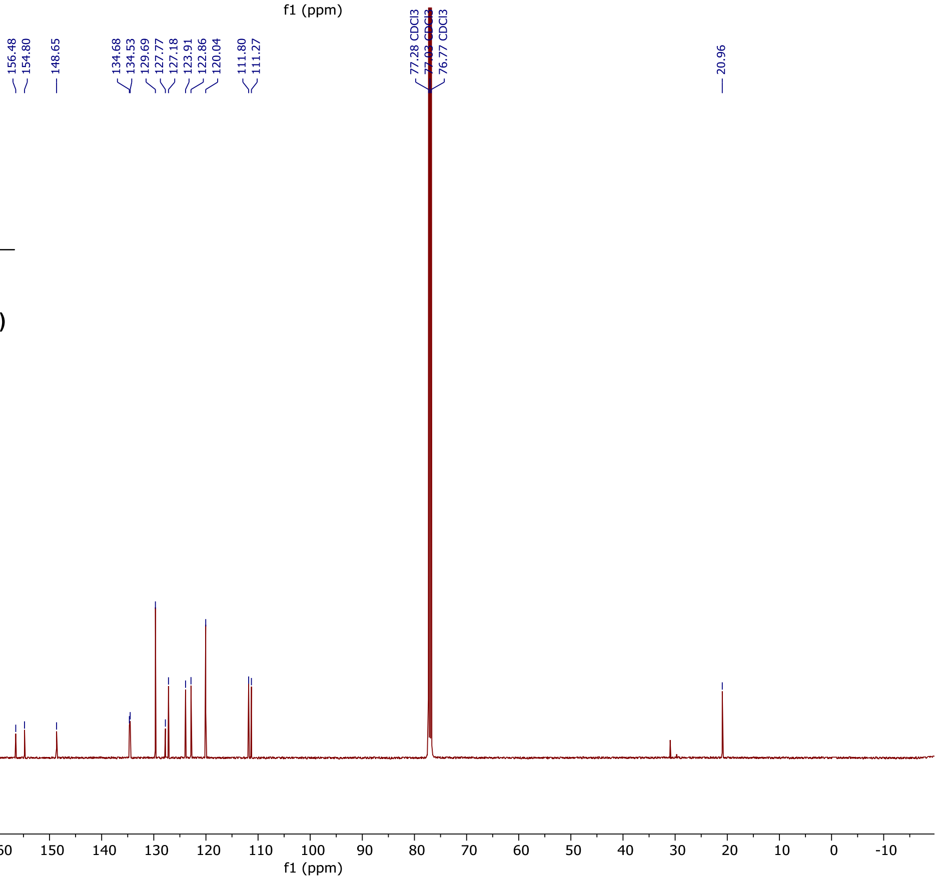

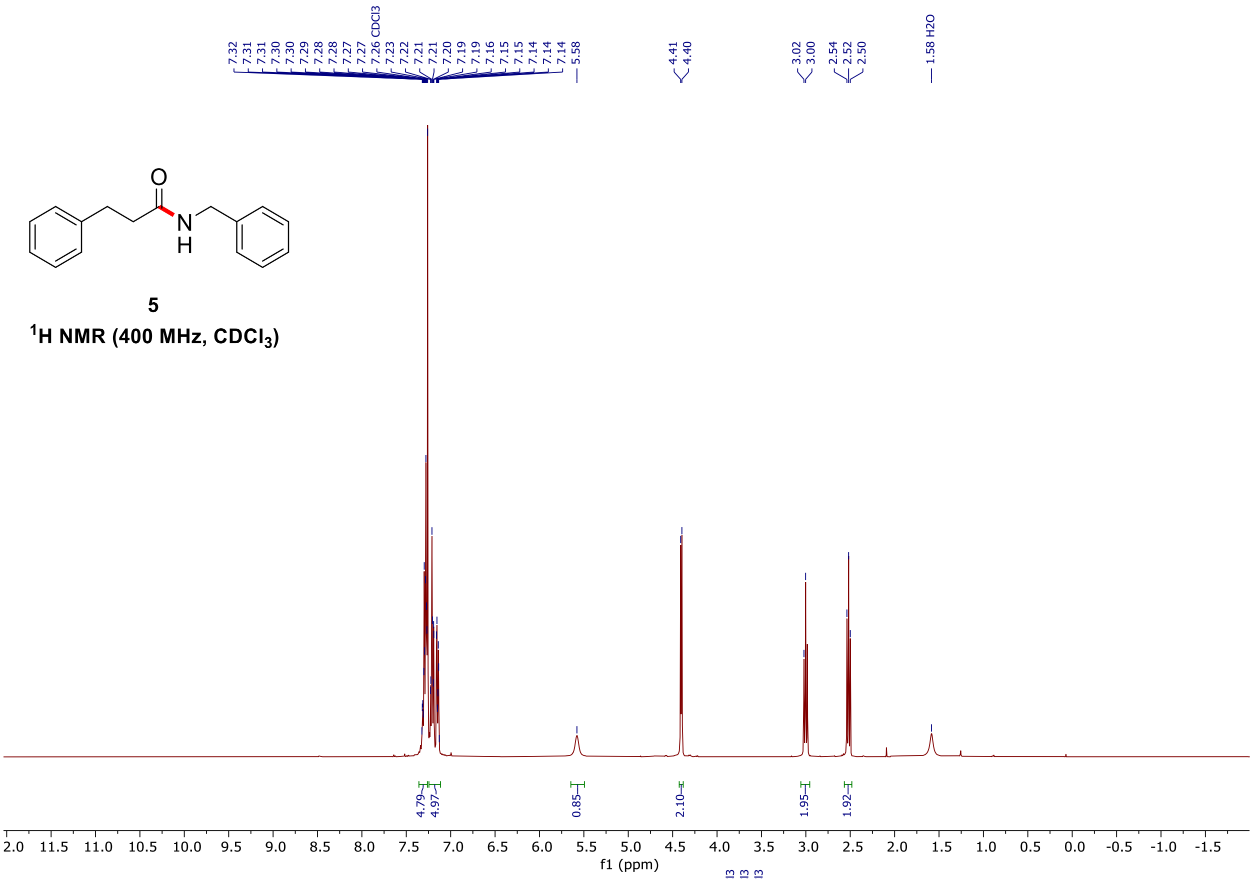

5

^{1}H NMR (400 MHz, $CDCl_3$)

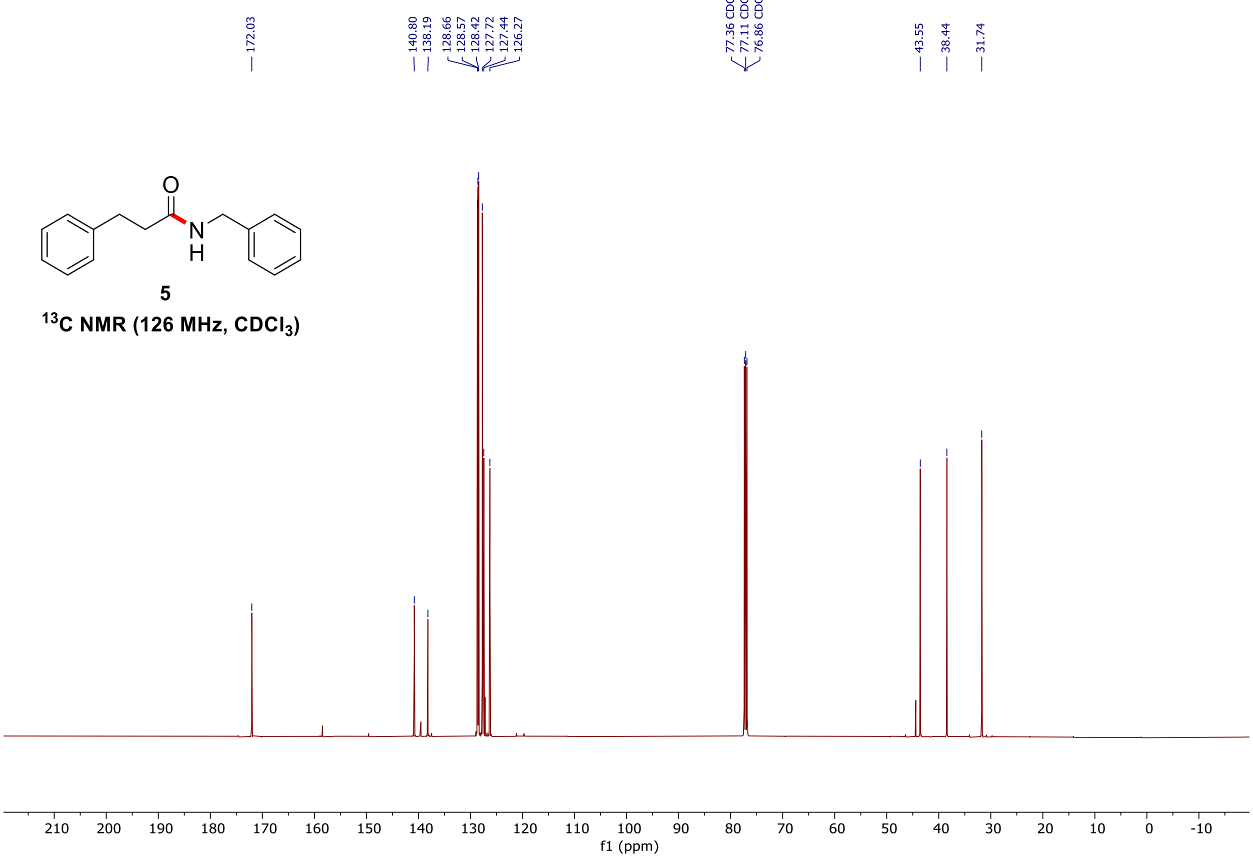

5

^{13}C NMR (126 MHz, $CDCl_3$)

6

^{1}H NMR (400 MHz, CDCl$_3$)

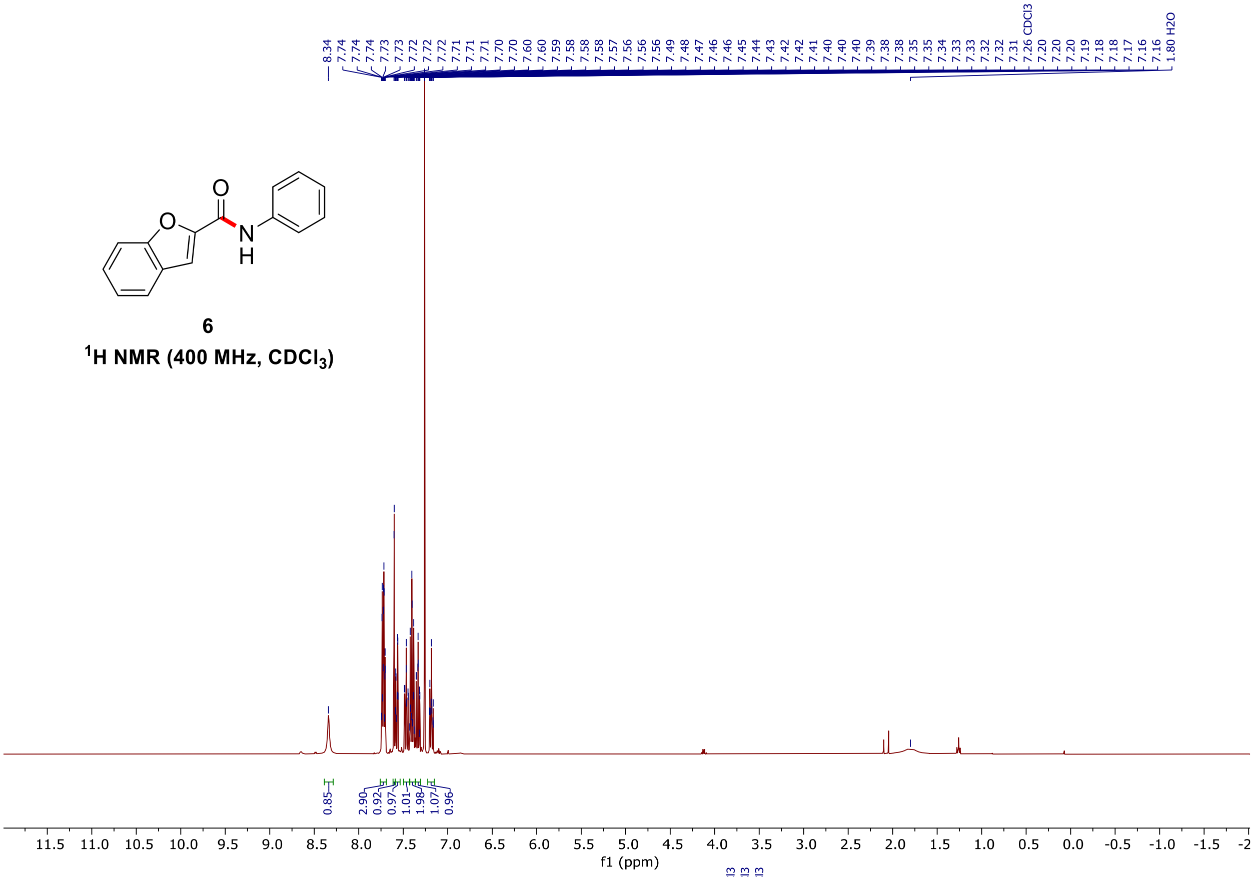

6

^{13}C NMR (126 MHz, CDCl$_3$)

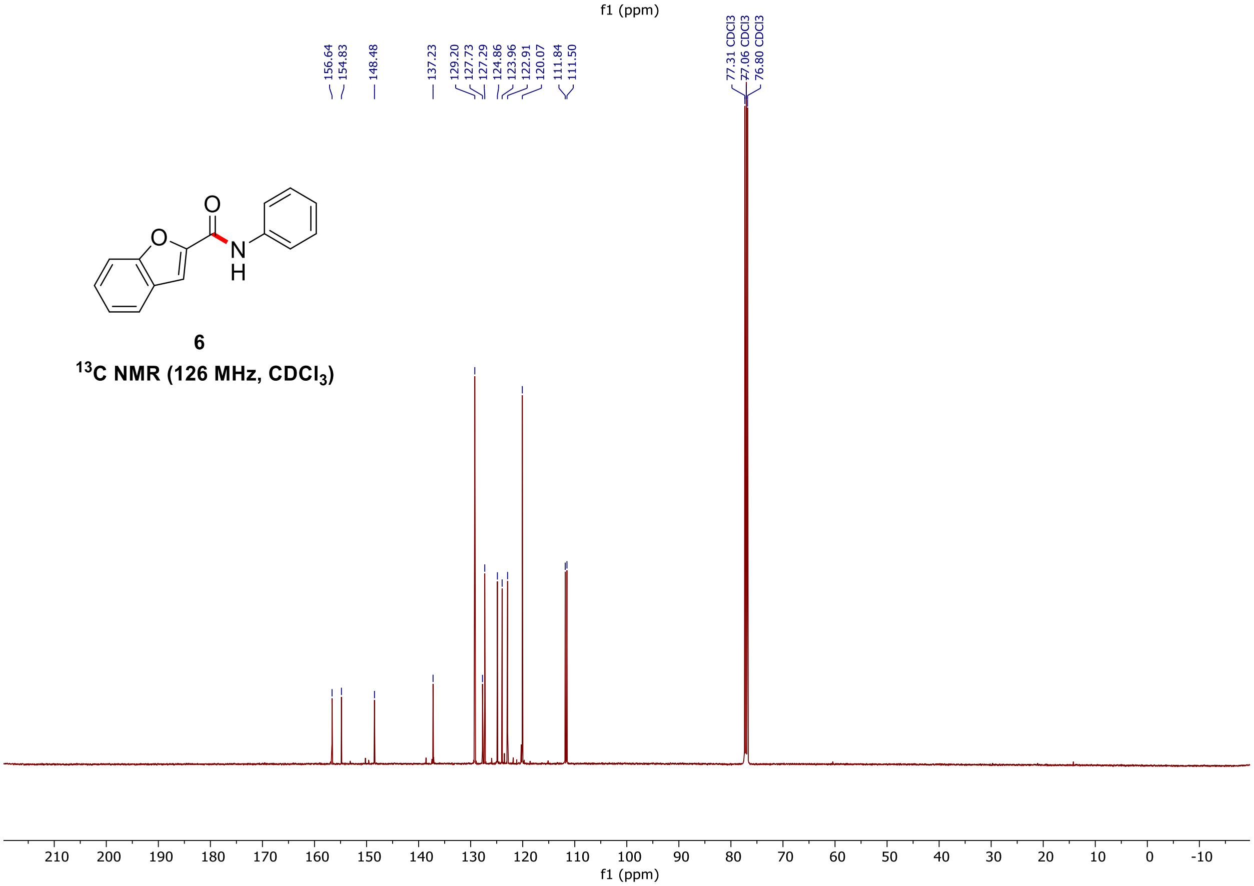

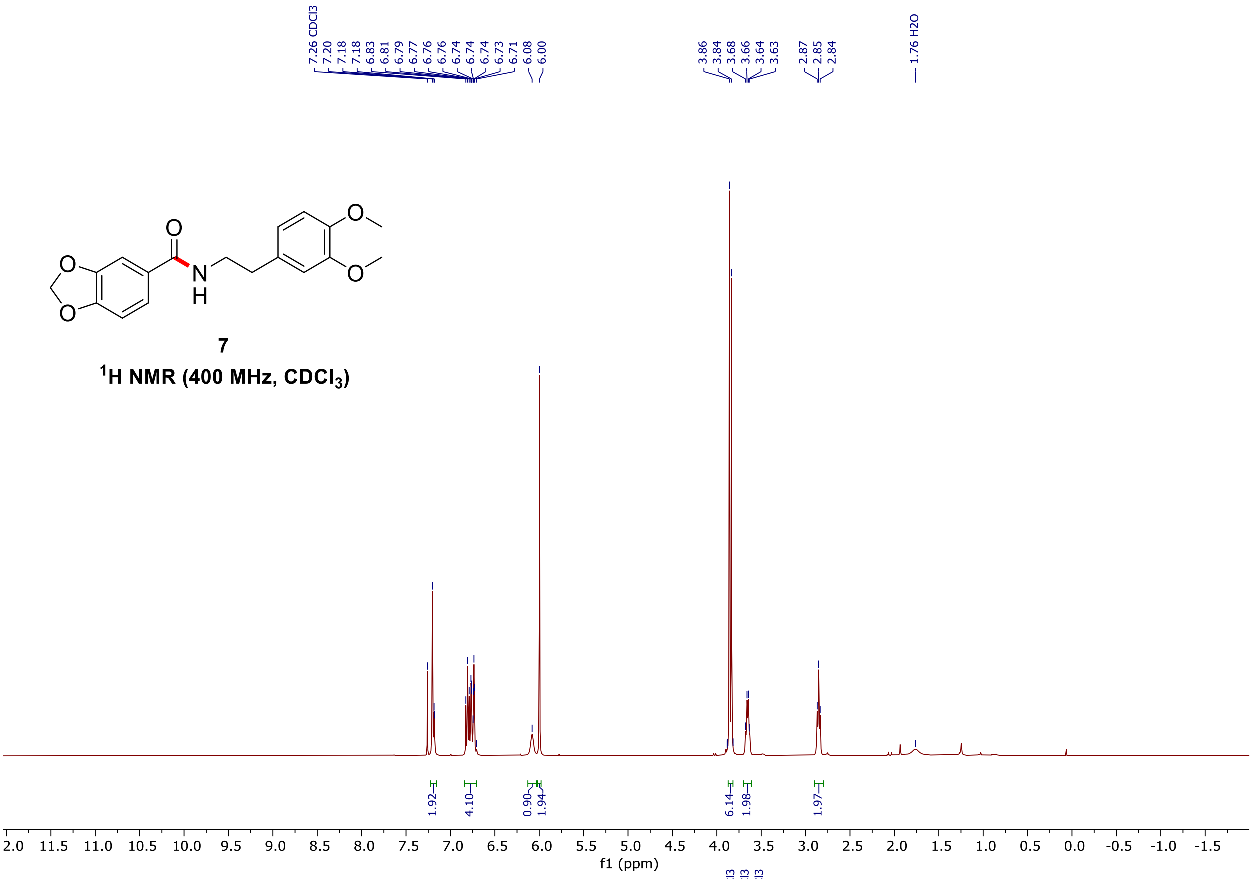
7.26 CDCl3
7.20
7.18
7.18
6.83
6.81
6.79
6.77
6.76
6.76
6.74
6.74
6.74
6.73
6.71
6.08
6.00
3.86
3.84
3.68
3.66
3.64
3.63
2.87
2.85
2.84
1.76 H2O
O
N
H
O
O
O
O
7
^{1}H NMR (400 MHz, CDCl$_3$)
1.92
4.10
0.90
1.94
6.14
1.98
1.97
f1 (ppm)

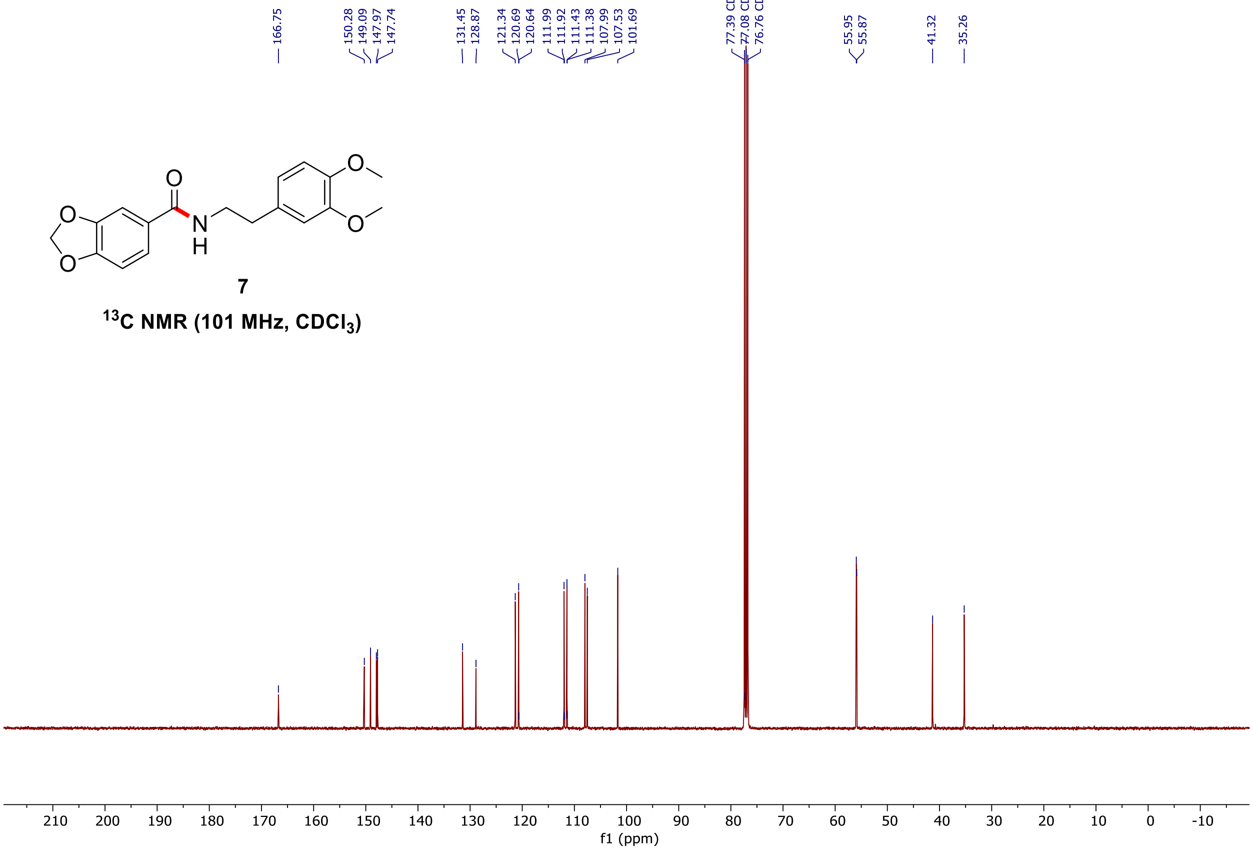
166.75
150.28
149.09
147.97
147.74
131.45
128.87
121.34
120.69
120.64
111.99
111.92
111.43
111.38
107.99
107.53
101.69
77.39 CDCl3
77.08 CDCl3
76.76 CDCl3
55.95
55.87
41.32
35.26
O
N
H
O
O
O
O
7
^{13}C NMR (101 MHz, CDCl$_3$)
f1 (ppm)

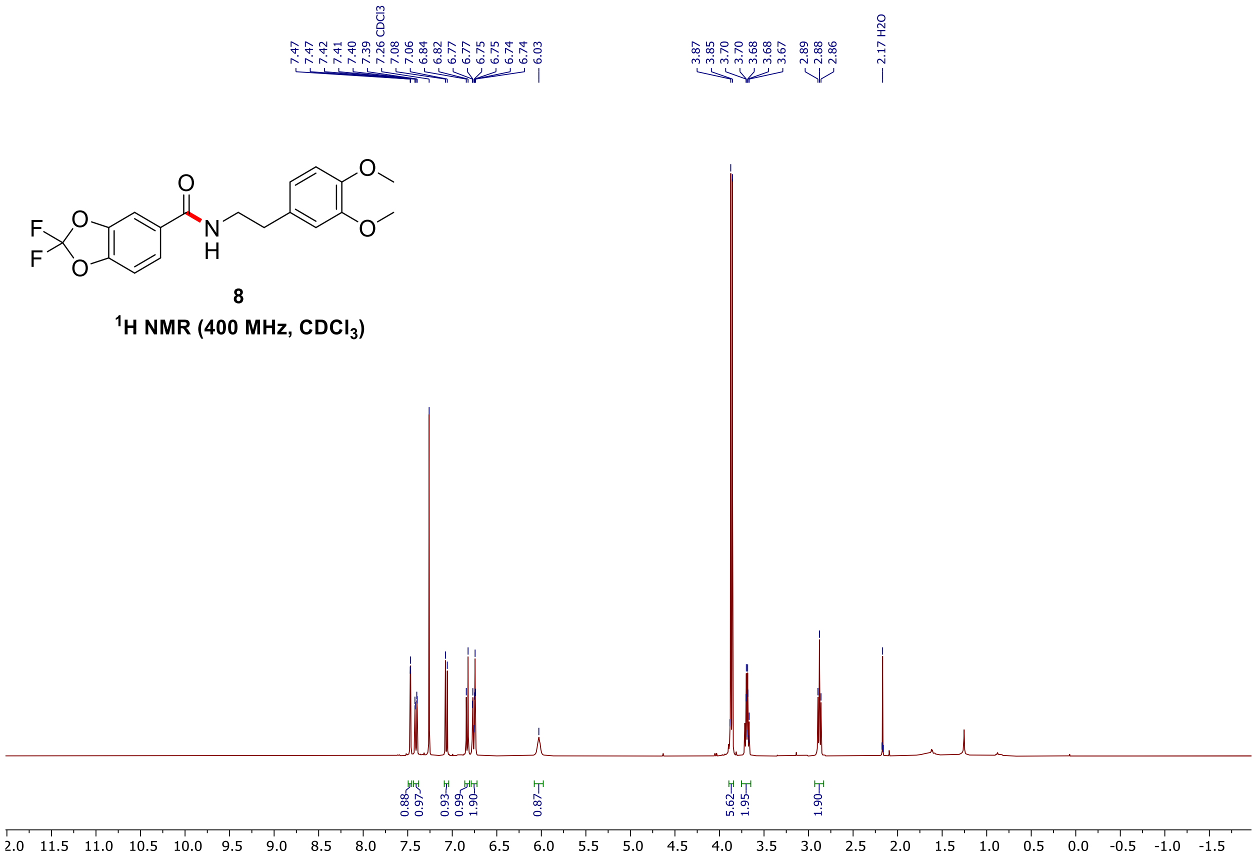

8

^{1}H NMR (400 MHz, $CDCl_3$)

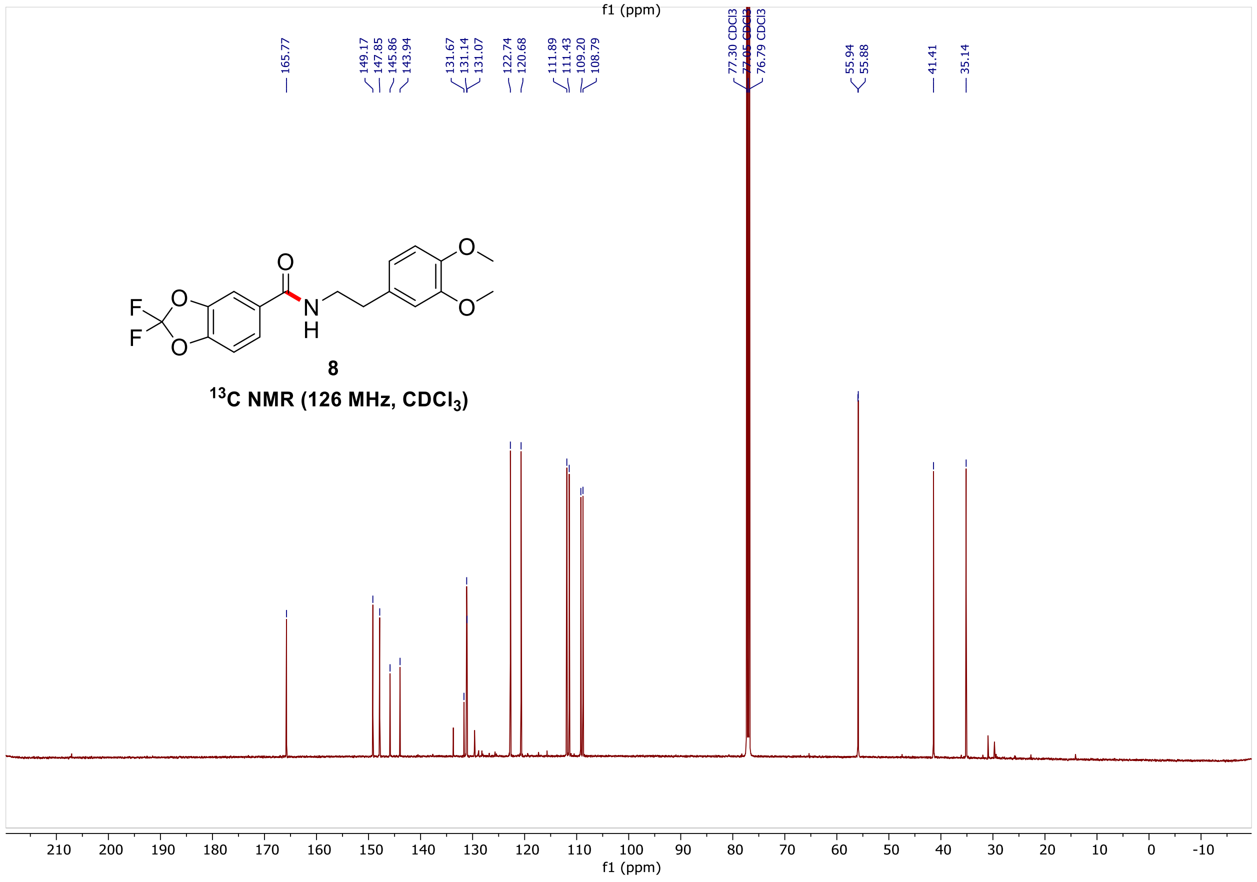

8

^{13}C NMR (126 MHz, $CDCl_3$)

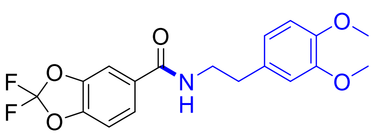

8

^{19}F NMR (500 MHz, $CDCl_3$)

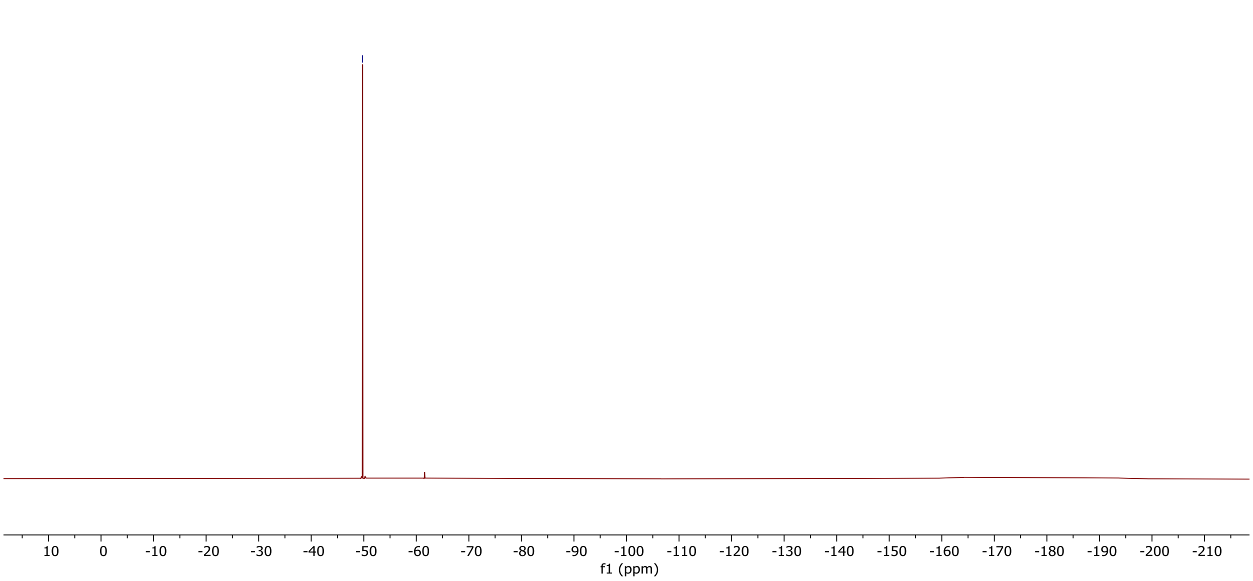

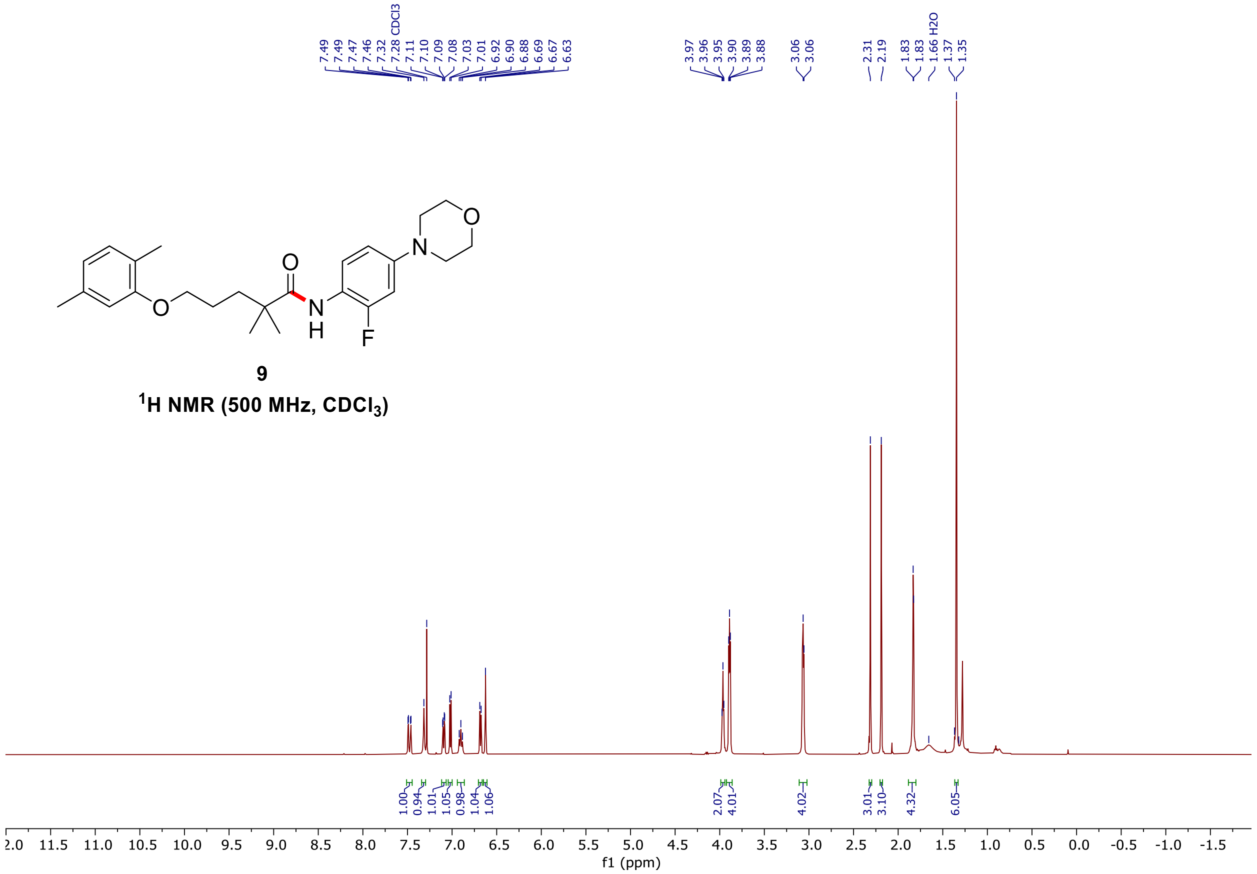

9

^{1}H NMR (500 MHz, $CDCl_3$)

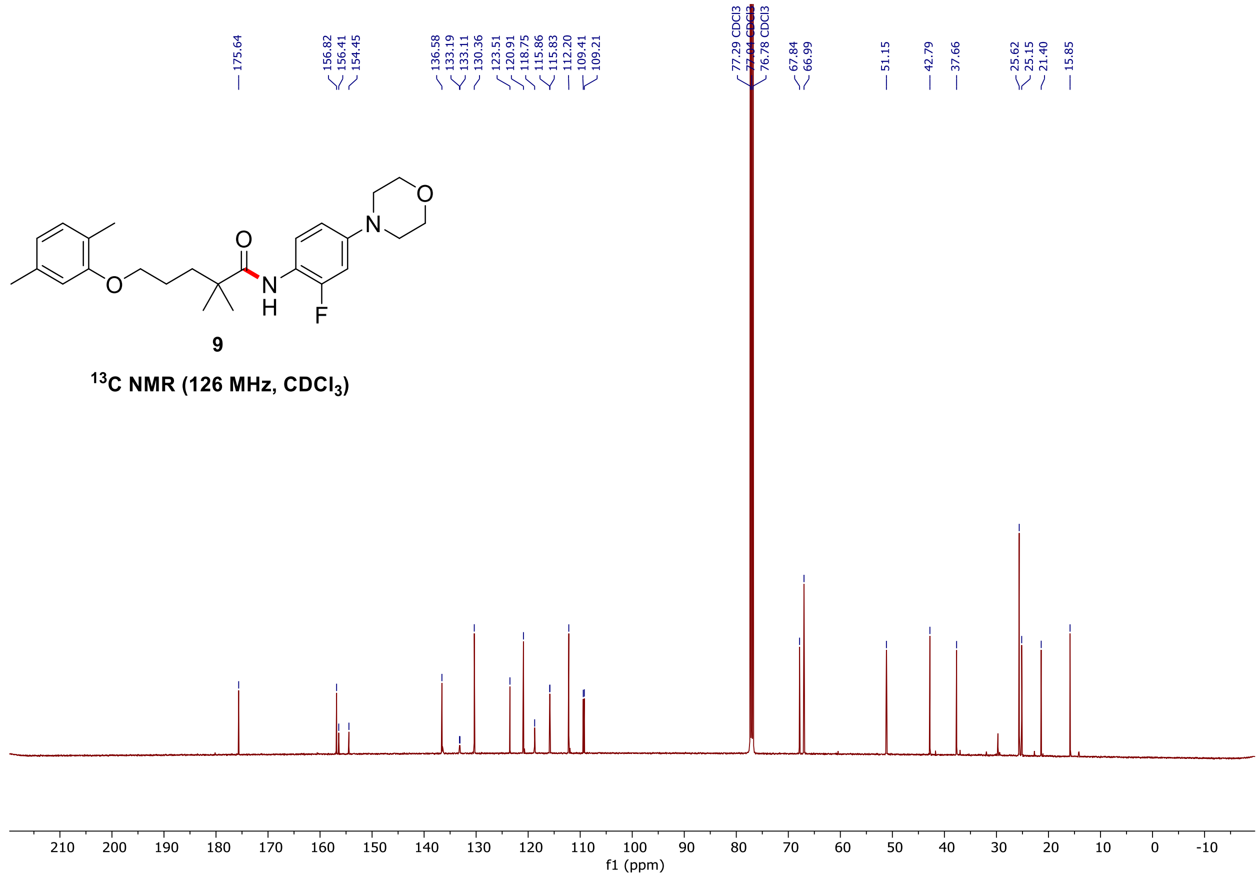

9

^{13}C NMR (126 MHz, $CDCl_3$)

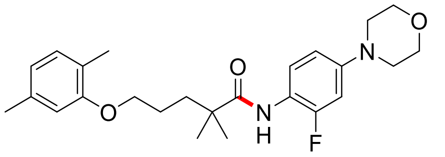

9

^{19}F NMR (500 MHz, $CDCl_3$)

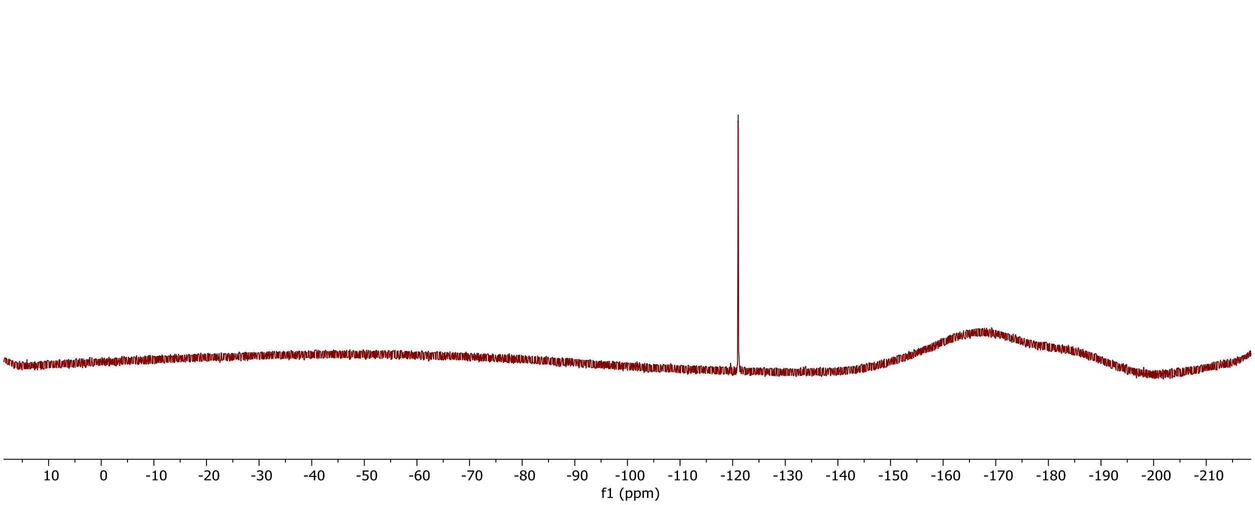

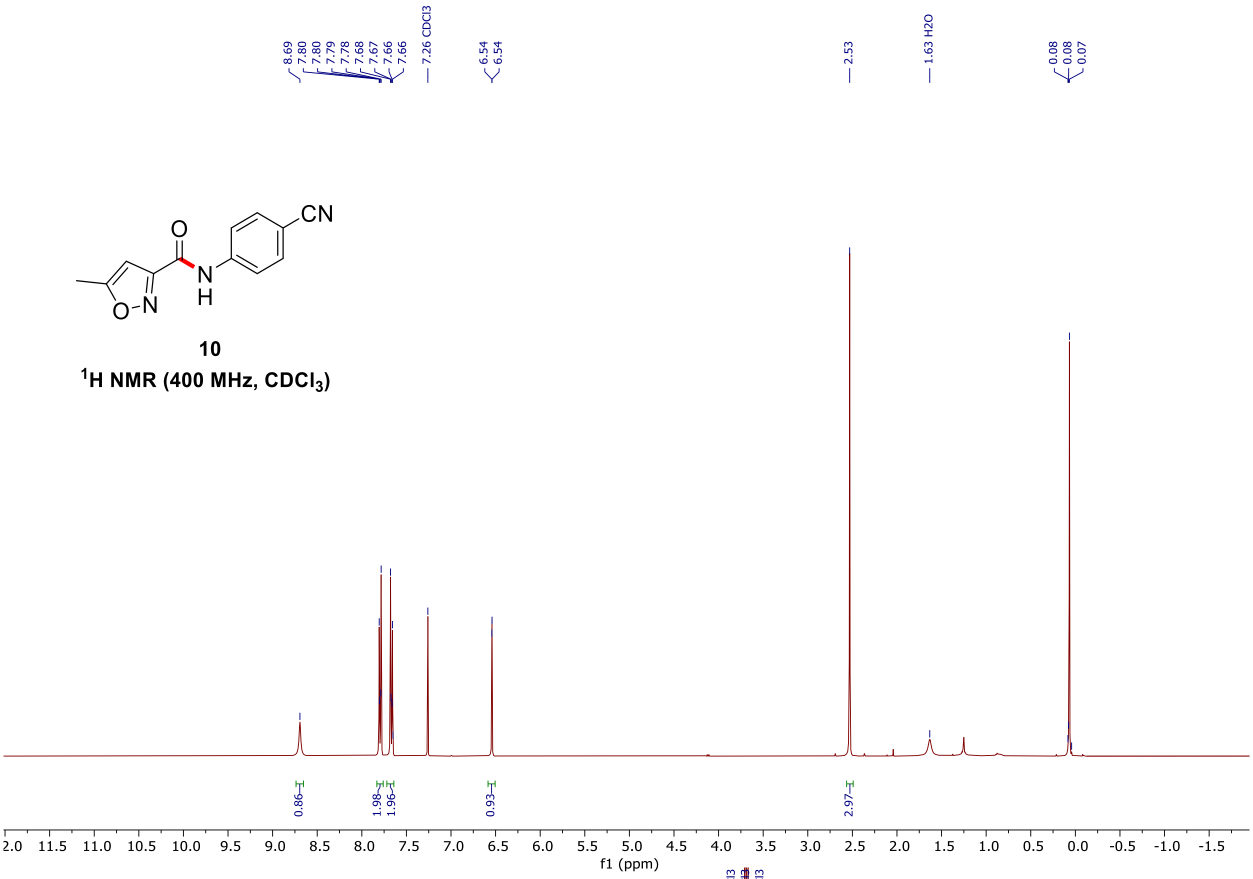
8.69
7.80
7.80
7.79
7.78
7.68
7.67
7.66
7.66
7.26 CDCl3
6.54
6.54
2.53
1.63 H2O
0.08
0.08
0.07
CN
O
N
H
O
N
10
1H NMR (400 MHz, CDCl3)
0.86
1.98
1.96
0.93
2.97
f1 (ppm)

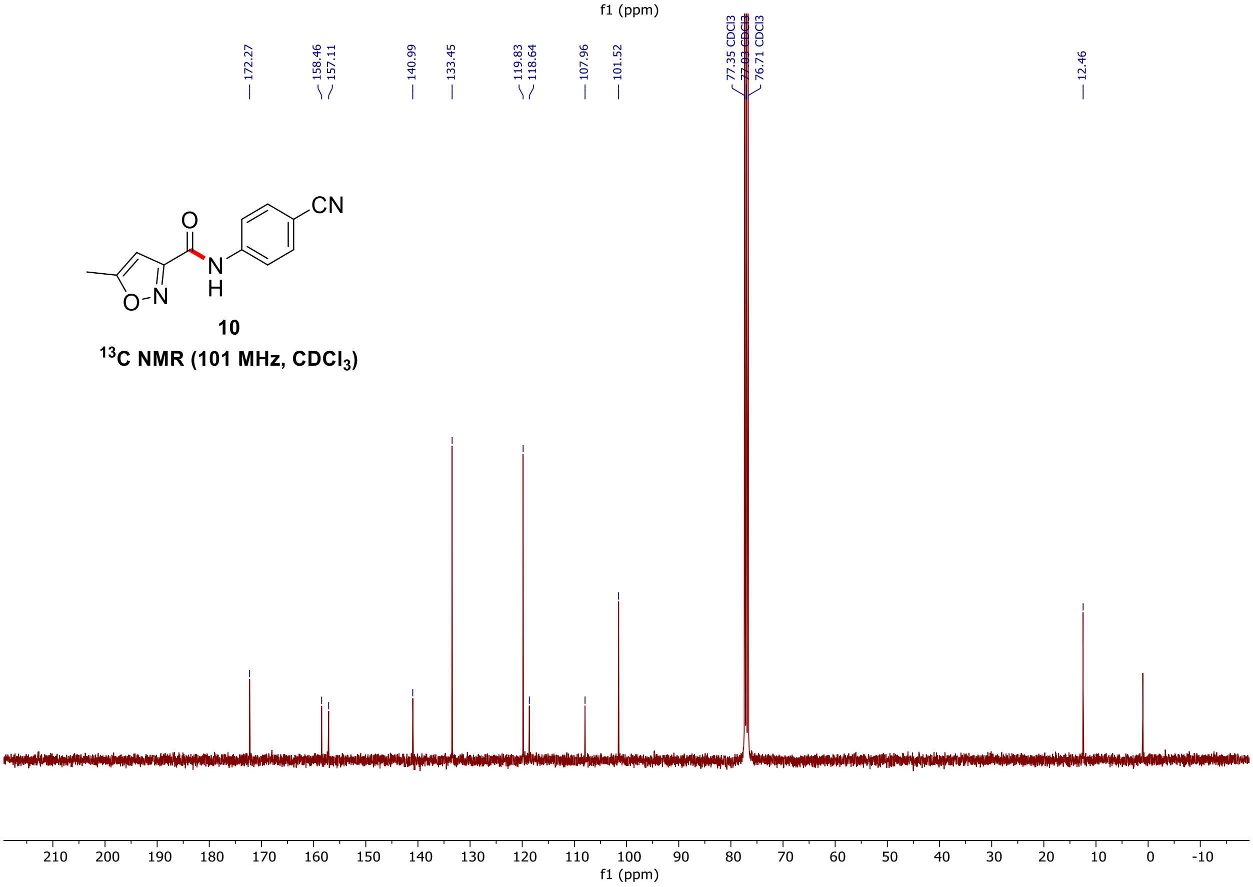
172.27
158.46
157.11
140.99
133.45
119.83
118.64
107.96
101.52
77.35 CDCl3
77.03 CDCl3
76.71 CDCl3
12.46
CN
O
N
H
O
N
10
13C NMR (101 MHz, CDCl3)
f1 (ppm)

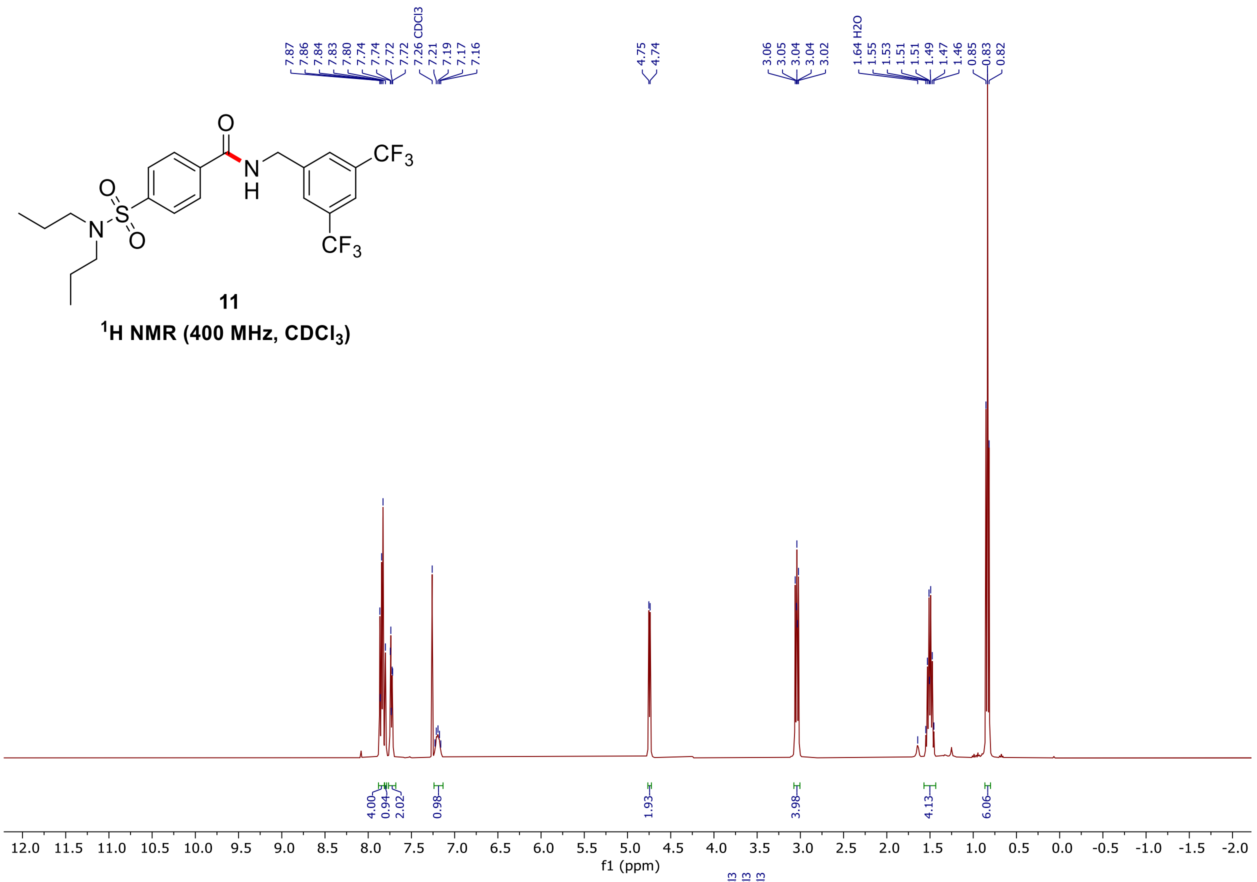
11
1H NMR (400 MHz, CDCl3)
7.87 7.86 7.84 7.83 7.80 7.74 7.74 7.72 7.72 7.26 CDCl3 7.21 7.19 7.17 7.16
4.75 4.74
3.06 3.05 3.04 3.04 3.02
1.64 H2O 1.55 1.53 1.51 1.51 1.49 1.47 1.46 0.85 0.83 0.82
4.00 0.94 2.02 0.98 1.93 3.98 4.13 6.06
f1 (ppm)

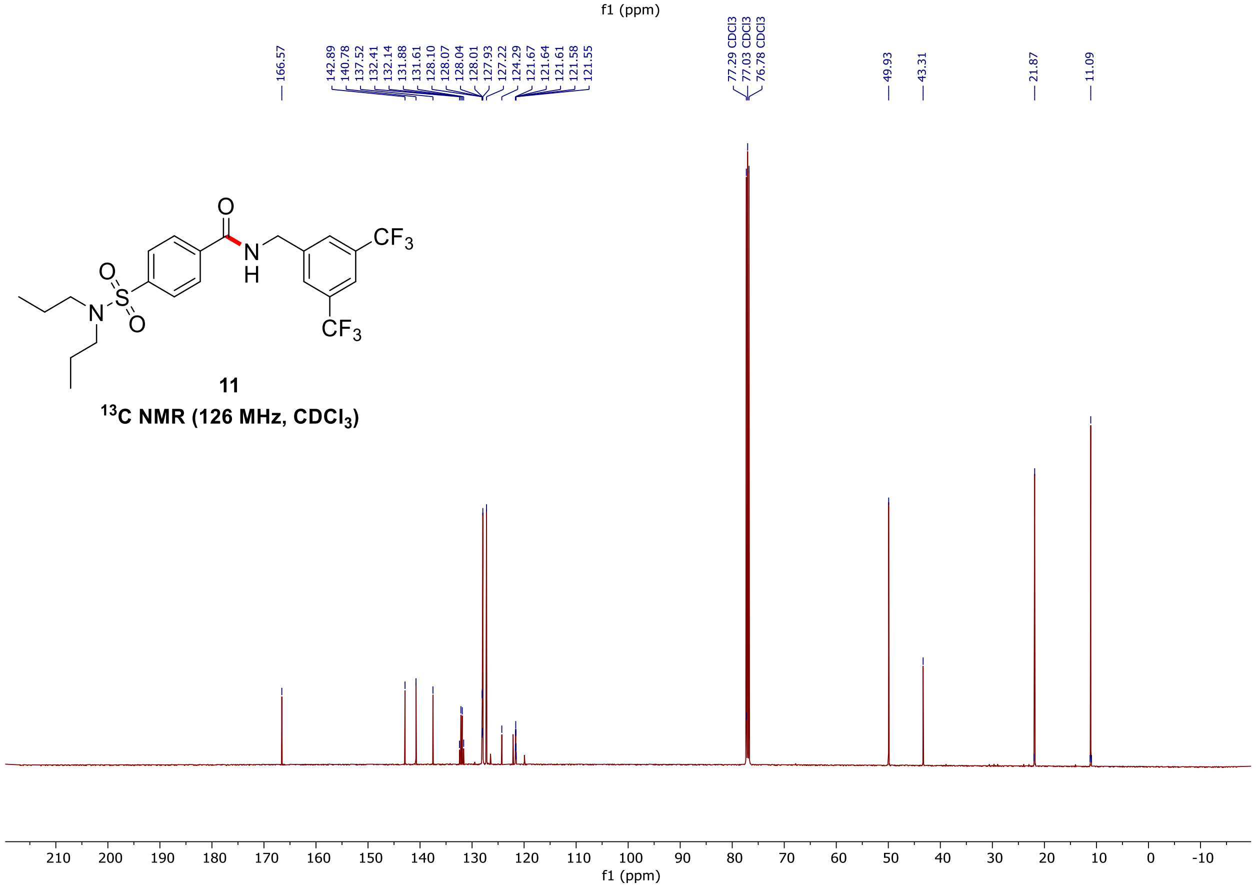
11
13C NMR (126 MHz, CDCl3)
166.57
142.89 140.78 137.52 132.41 132.14 131.88 131.61 128.10 128.07 128.04 128.01 127.93 127.22 124.29 121.67 121.64 121.61 121.58 121.55
77.29 CDCl3 77.03 CDCl3 76.78 CDCl3
49.93
43.31
21.87
11.09
f1 (ppm)

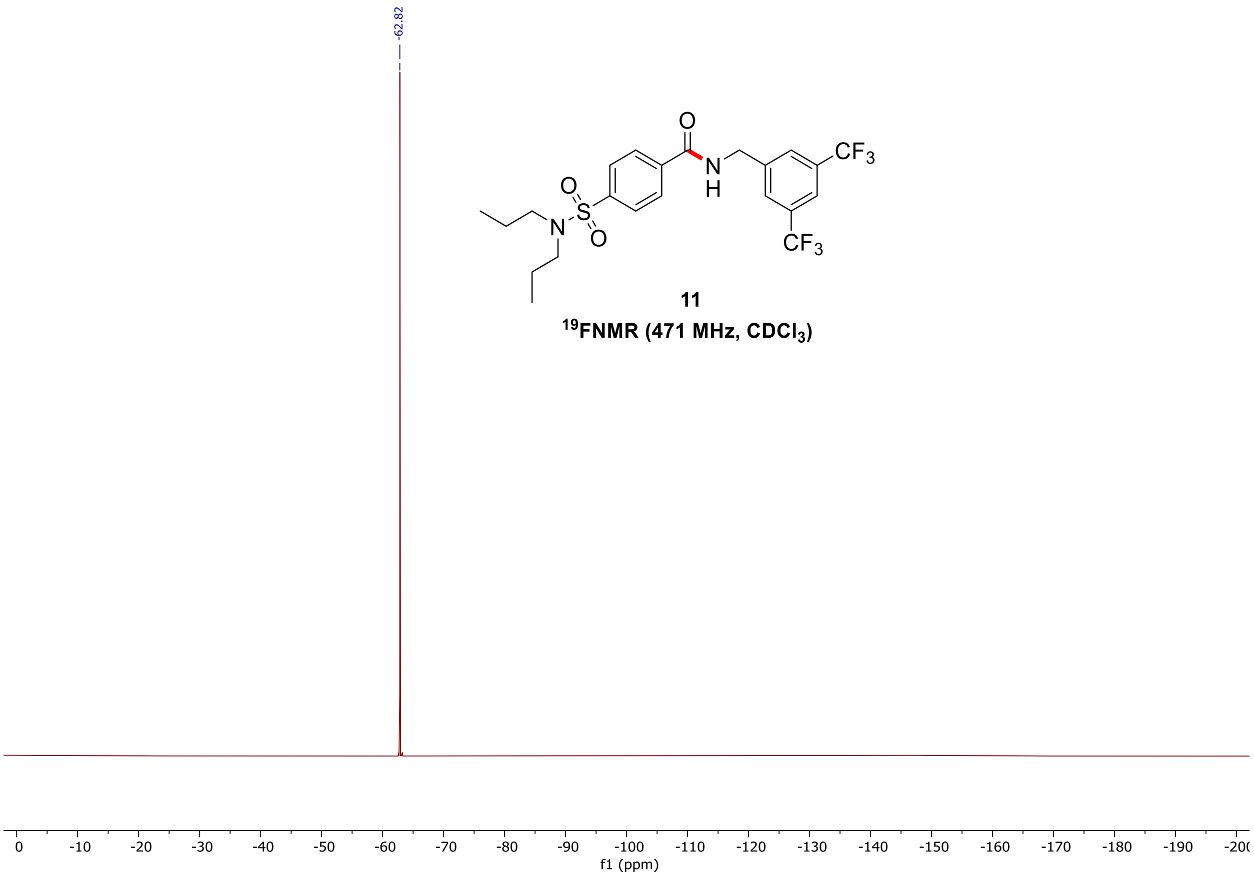
-62.82
11
19FNMR (471 MHz, CDCl3)
0 -10 -20 -30 -40 -50 -60 -70 -80 -90 -100 -110 -120 -130 -140 -150 -160 -170 -180 -190 -200
f1 (ppm)

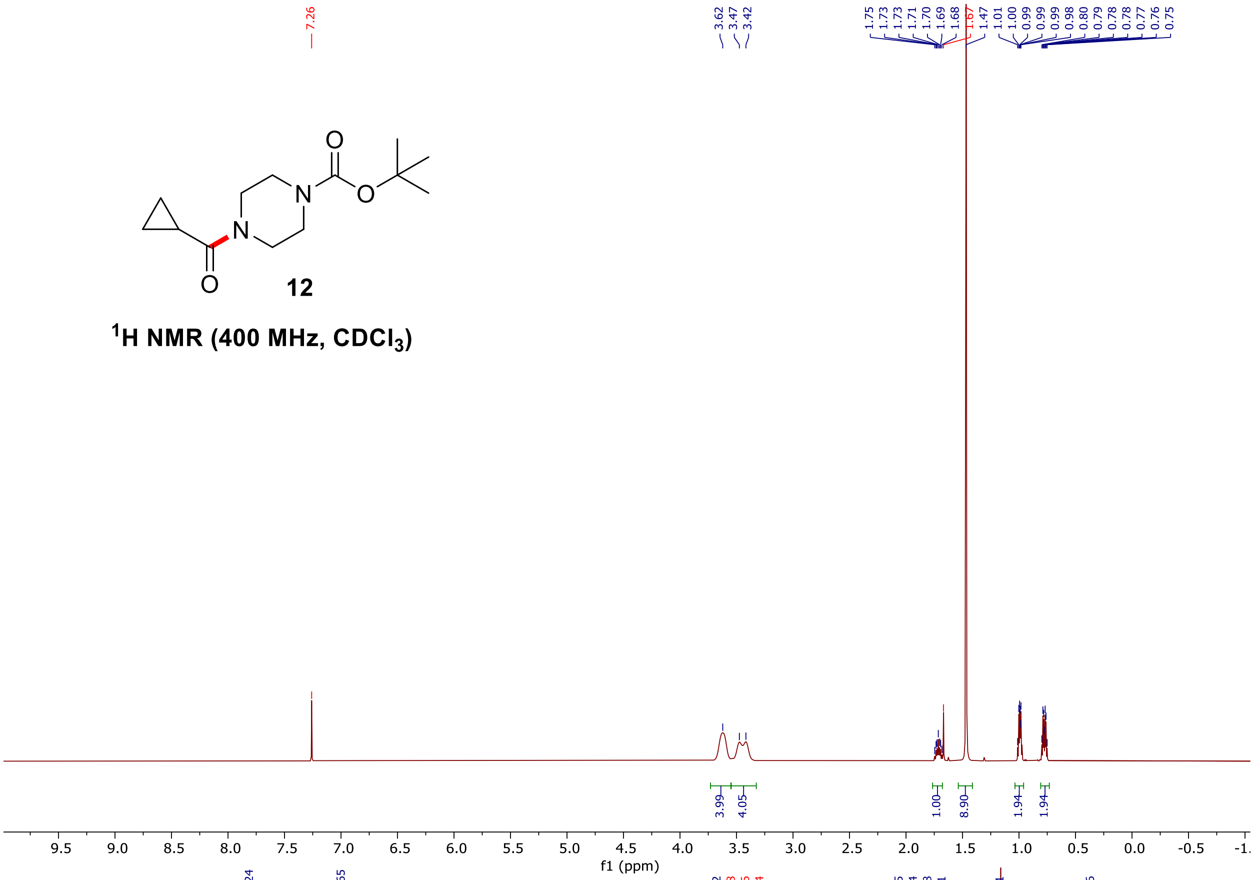
7.26
3.62
3.47
3.42
1.75
1.73
1.73
1.71
1.70
1.69
1.68
1.67
1.47
1.01
1.00
0.99
0.99
0.99
0.98
0.80
0.79
0.78
0.78
0.77
0.76
0.75
O
N
N
O
O
12
^{1}H NMR (400 MHz, CDCl$_3$)
3.99
4.05
1.00
8.90
1.94
1.94
9.5 9.0 8.5 8.0 7.5 7.0 6.5 6.0 5.5 5.0 4.5 4.0 3.5 3.0 2.5 2.0 1.5 1.0 0.5 0.0 -0.5 -1.
f1 (ppm)

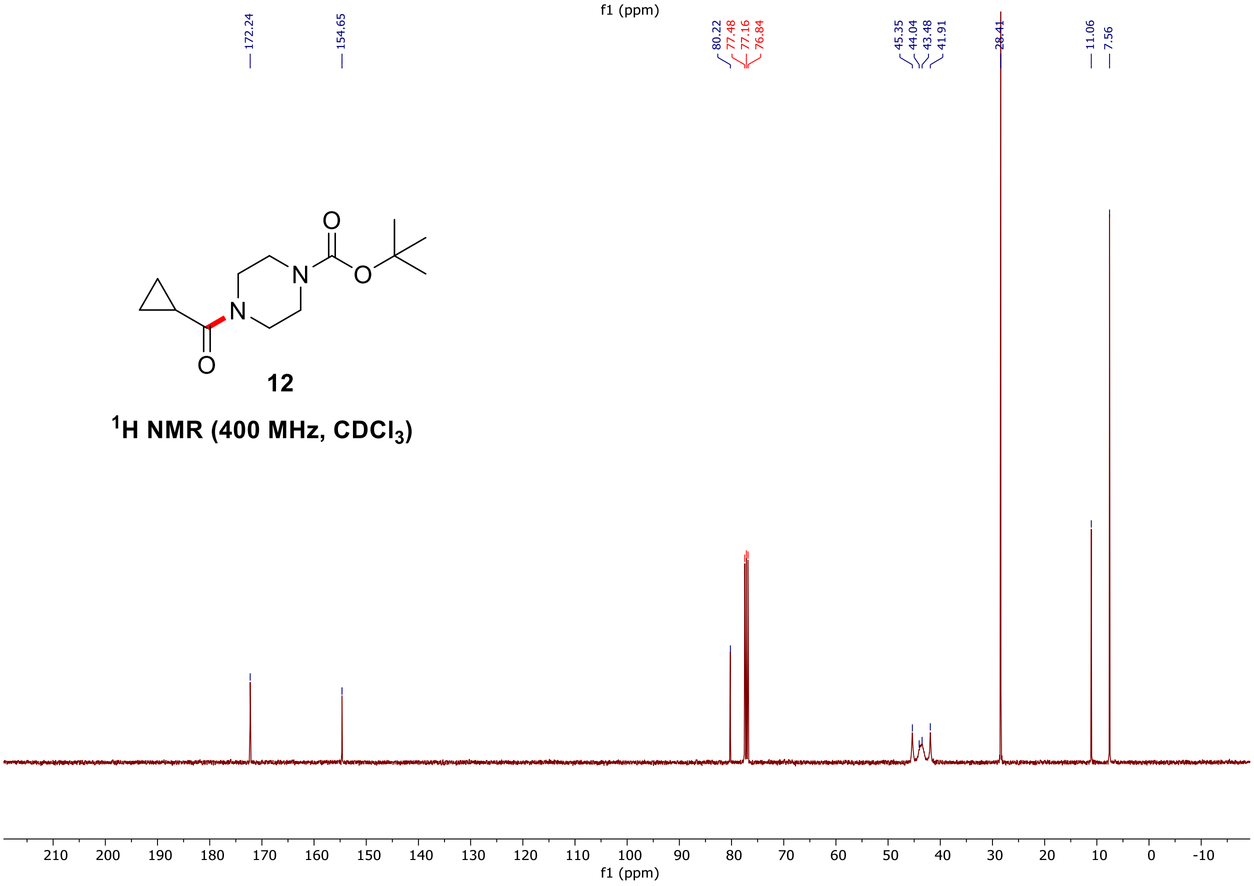
172.24
154.65
80.22
77.48
77.16
76.84
45.35
44.04
43.48
41.91
28.41
11.06
7.56
O
N
N
O
O
12
^{1}H NMR (400 MHz, CDCl$_3$)
210 200 190 180 170 160 150 140 130 120 110 100 90 80 70 60 50 40 30 20 10 0 -10
f1 (ppm)

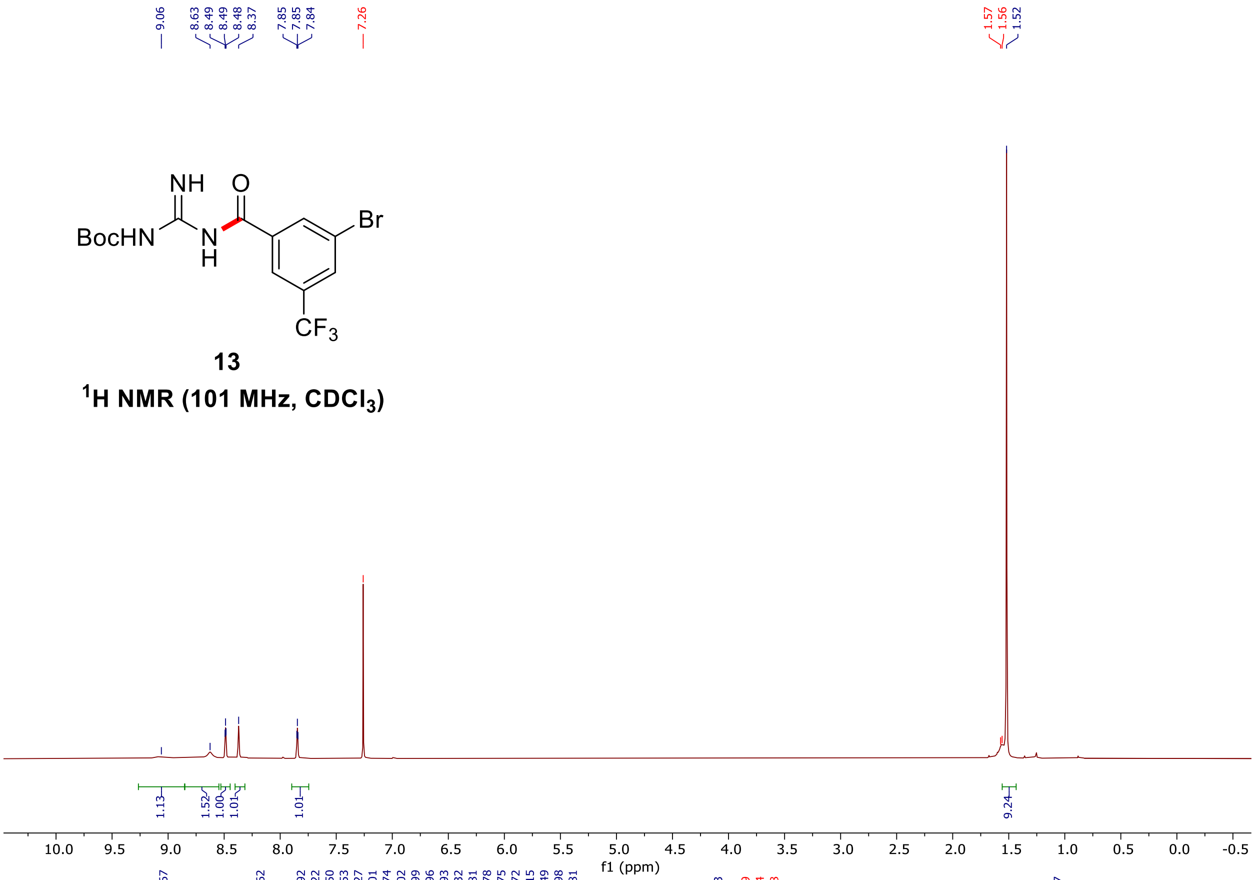

13

^{1}H NMR (101 MHz, $CDCl_3$)

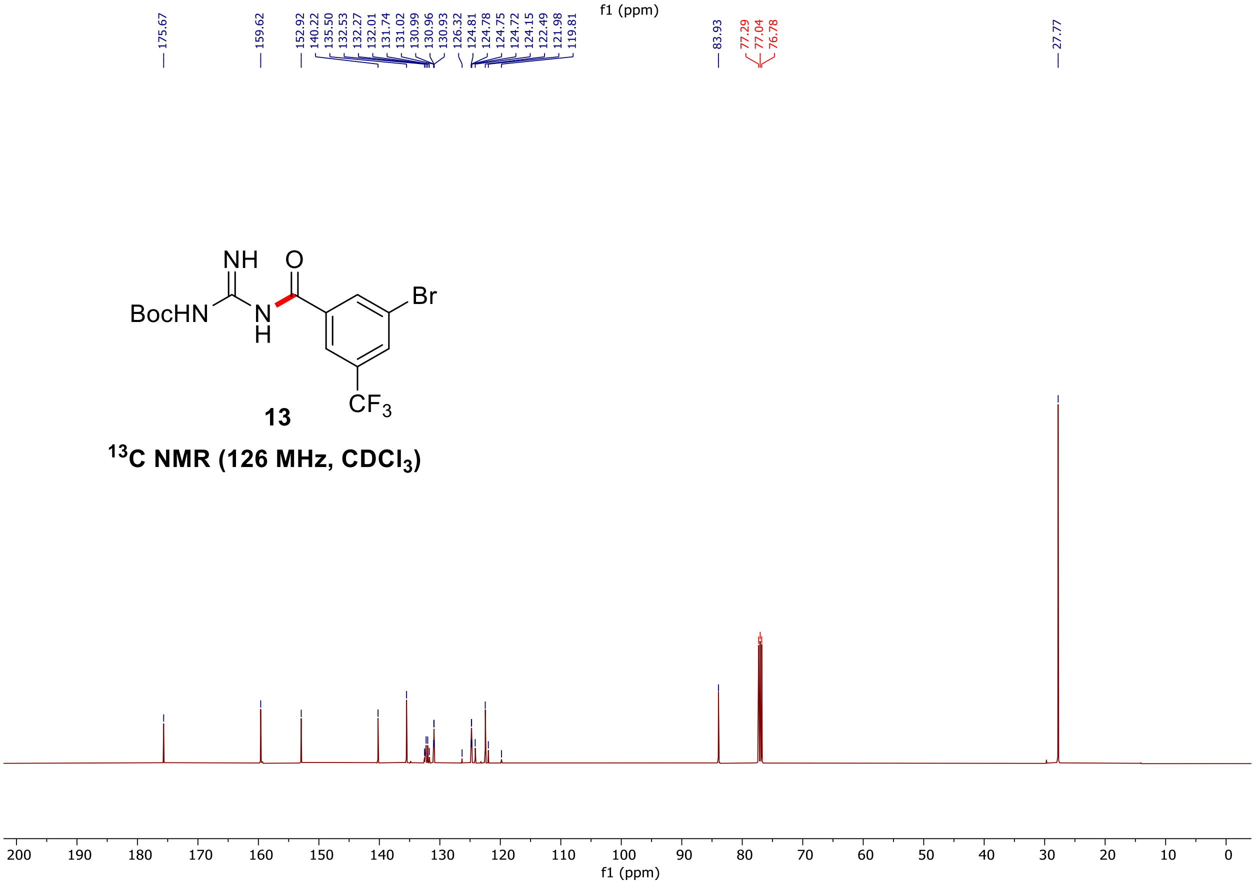

13

^{13}C NMR (126 MHz, $CDCl_3$)

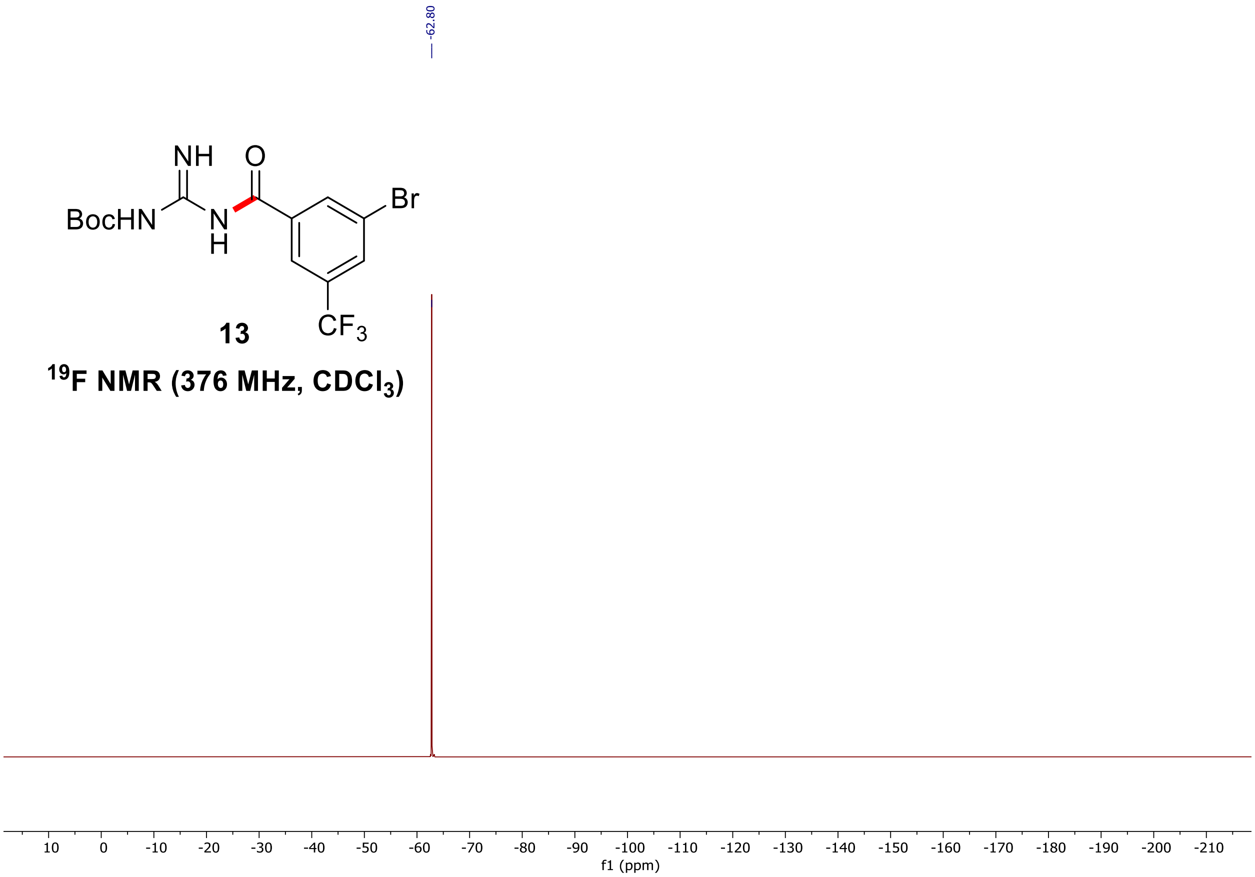

13

^{19}F NMR (376 MHz, $CDCl_3$)

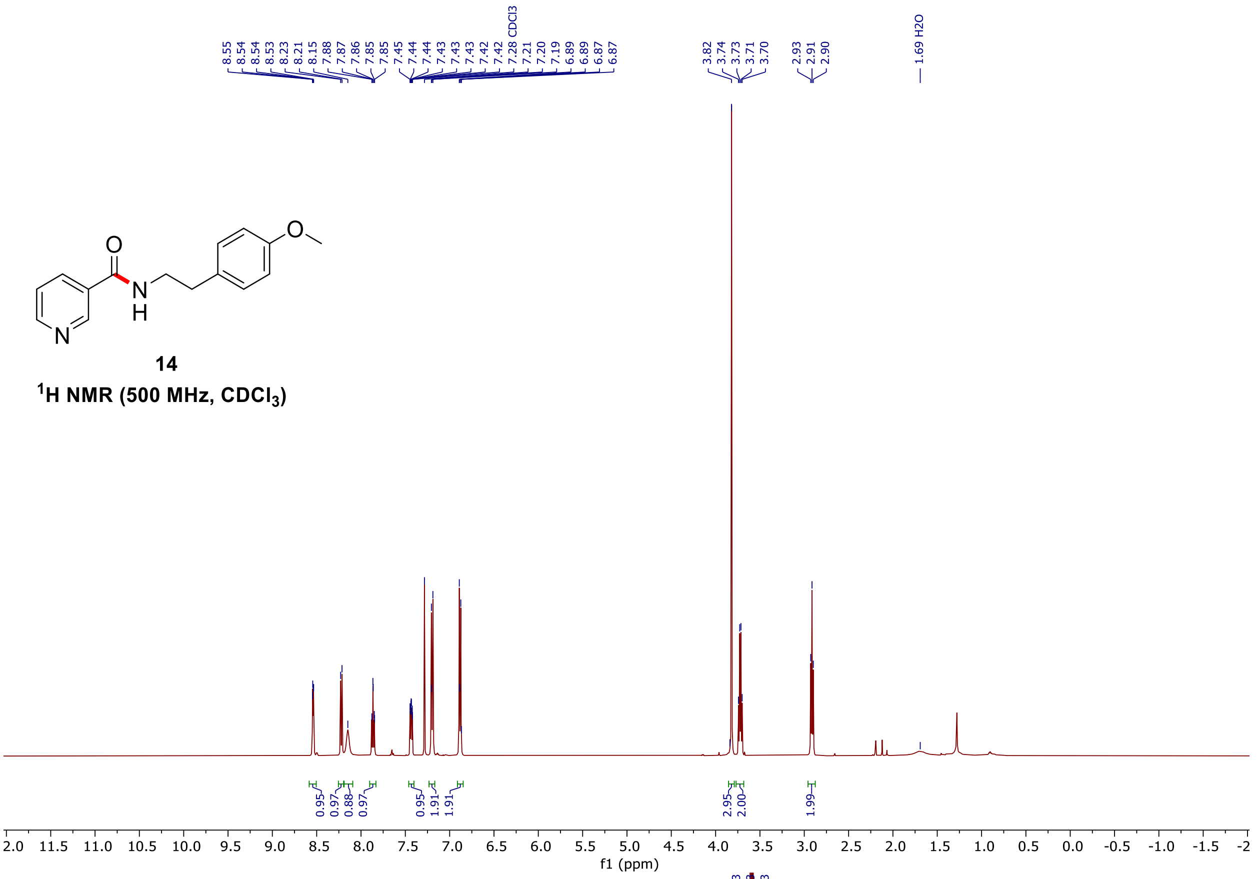
8.55 8.54 8.54 8.53 8.23 8.21 8.15 7.88 7.87 7.86 7.85 7.85 7.45 7.44 7.44 7.43 7.43 7.43 7.42 7.42 7.28 CDCl3 7.21 7.20 7.19 6.89 6.89 6.87 6.87
3.82 3.74 3.73 3.71 3.70
2.93 2.91 2.90
1.69 H2O
O
N
H
N
O
14
^{1}H NMR (500 MHz, CDCl$_3$)
0.95 0.97 0.88 0.97 0.95 1.91 1.91 2.95 2.00 1.99
f1 (ppm)

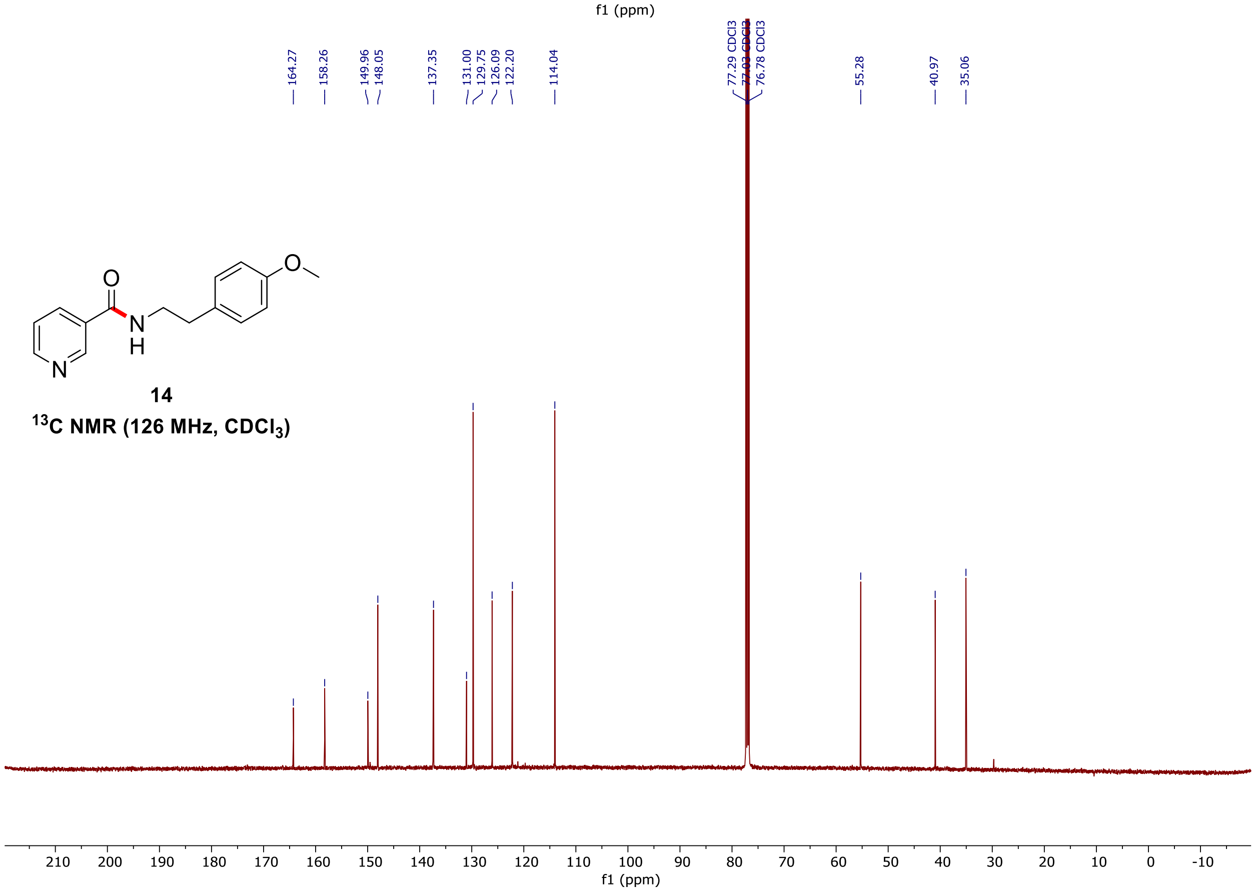
164.27 158.26 149.96 148.05 137.35 131.00 129.75 126.09 122.20 114.04
77.29 CDCl3 77.03 CDCl3 76.78 CDCl3
55.28 40.97 35.06
O
N
H
N
O
14
^{13}C NMR (126 MHz, CDCl$_3$)
f1 (ppm)

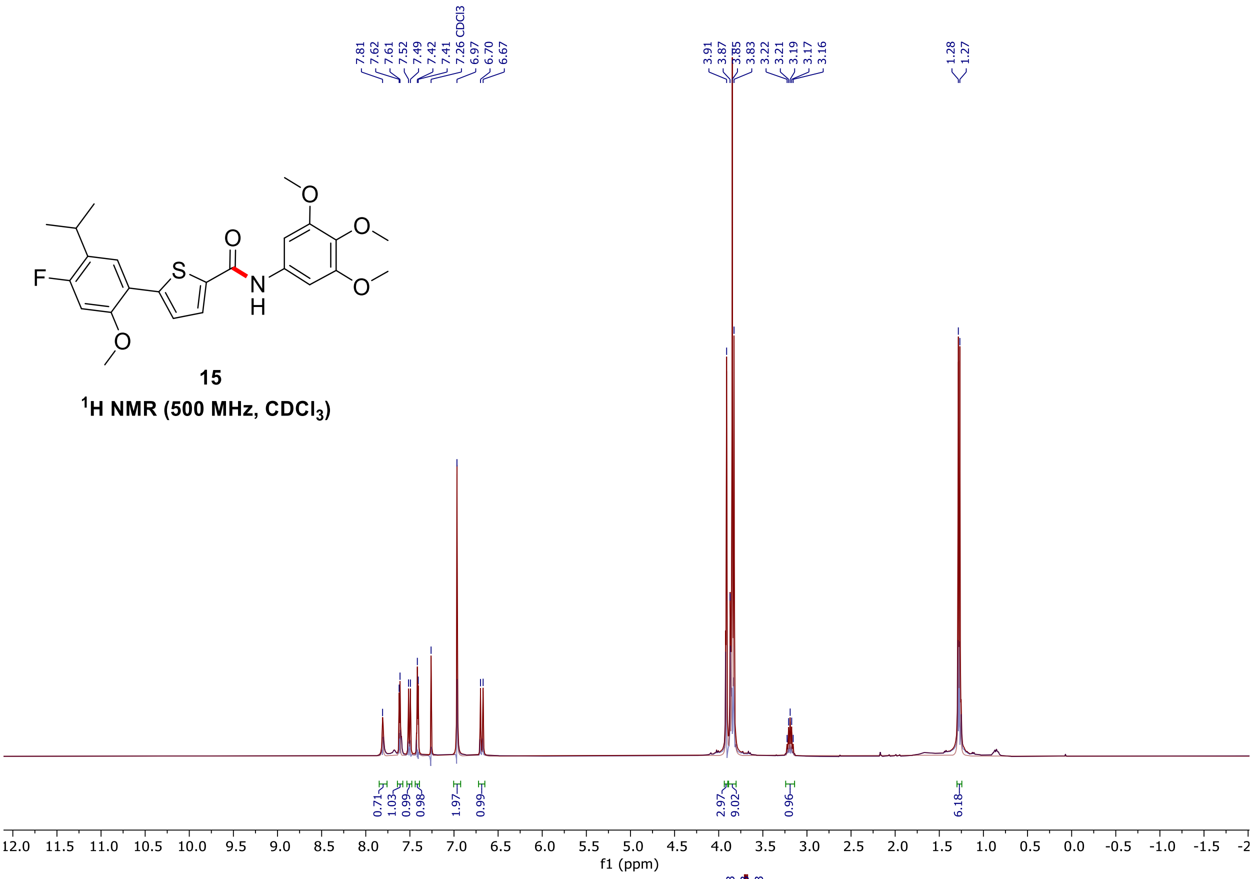

15

^{1}H NMR (500 MHz, $CDCl_3$)

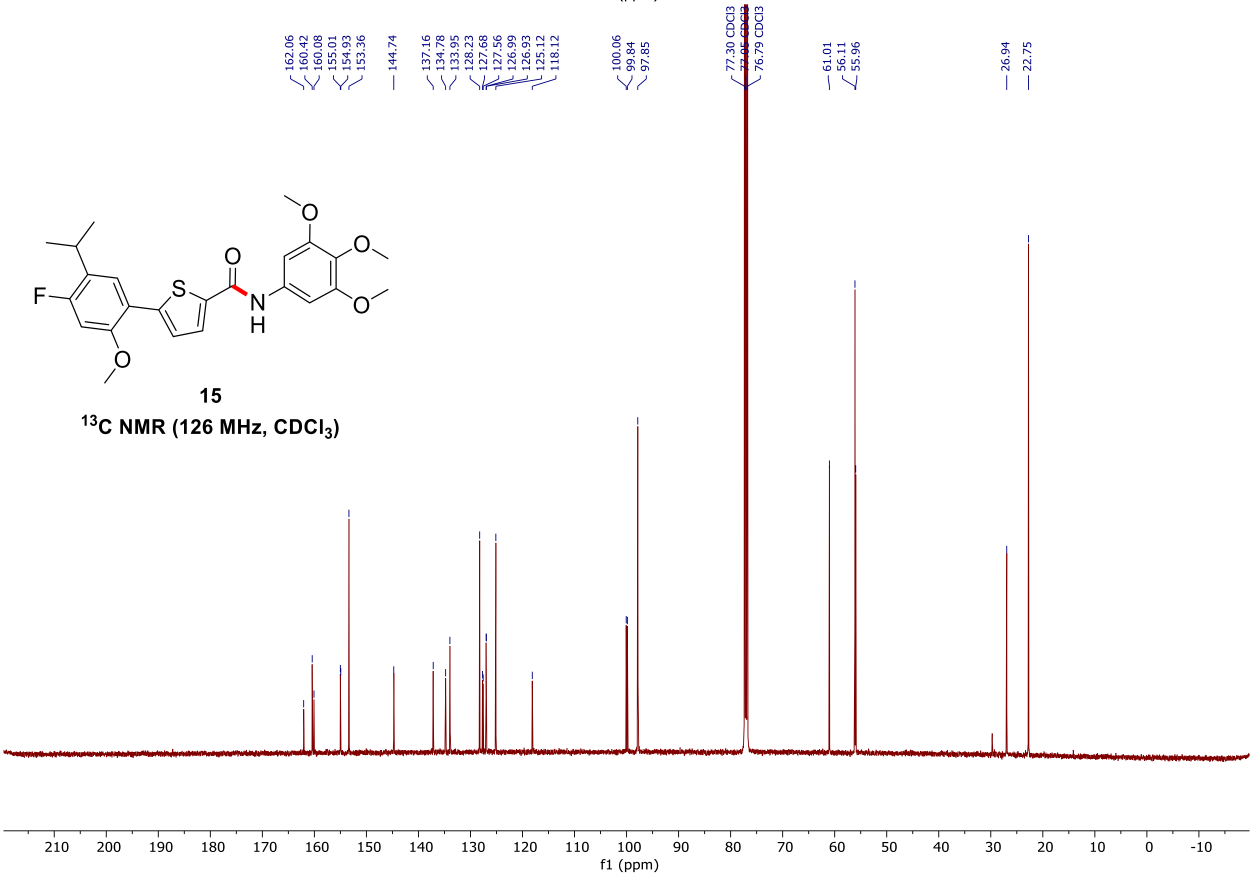

15

^{13}C NMR (126 MHz, $CDCl_3$)

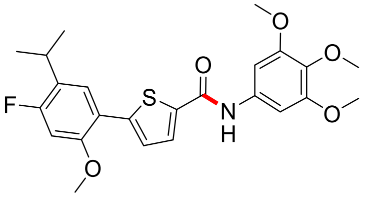

15

^{19}F NMR (376 MHz, $CDCl_3$)

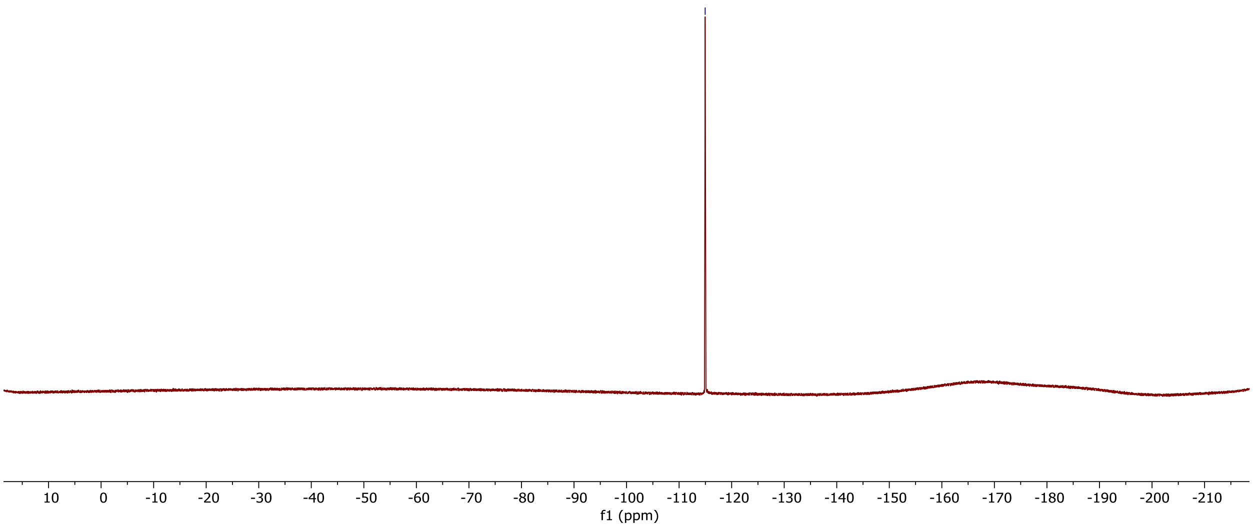

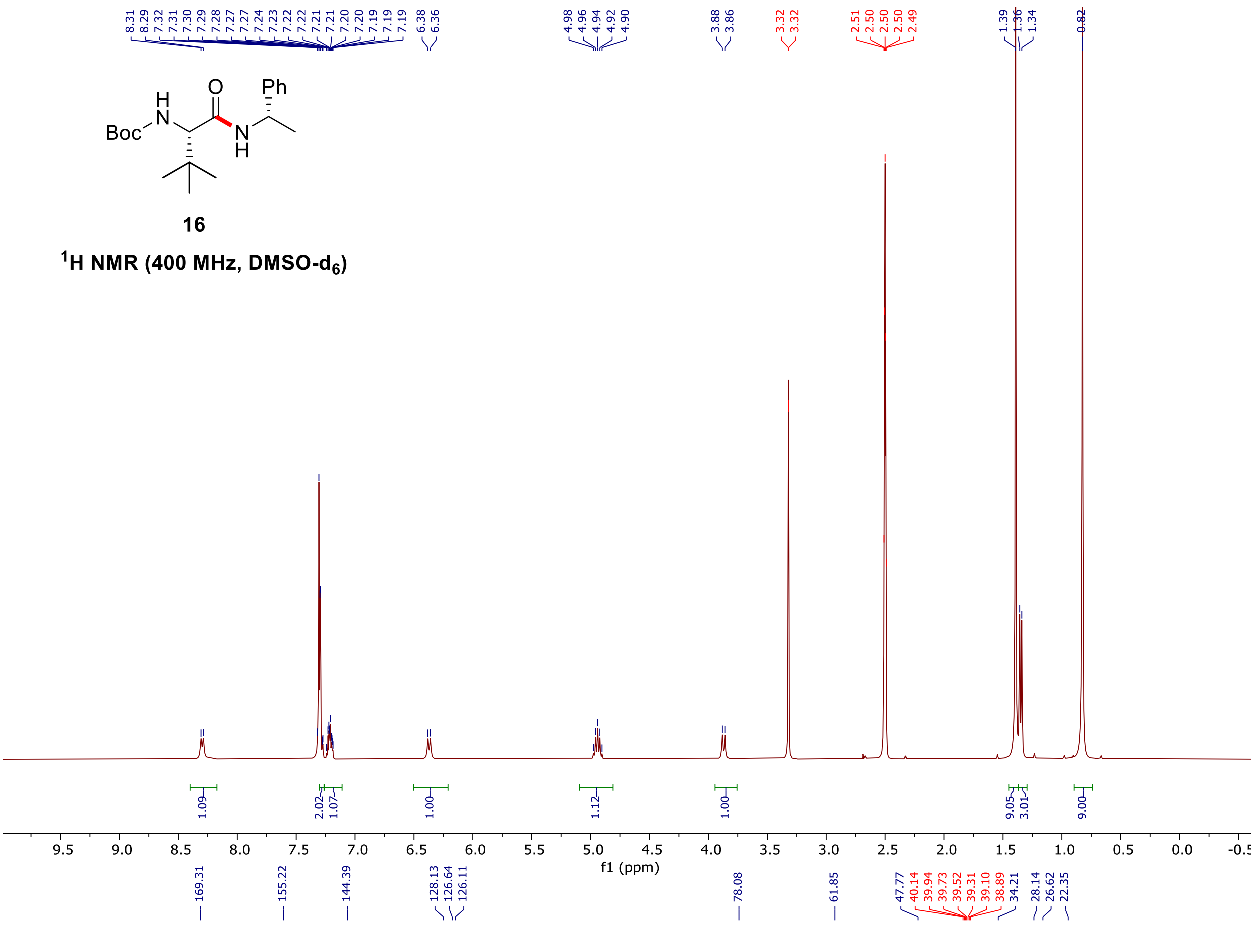

16

^{1}H NMR (400 MHz, DMSO-d$_6$)

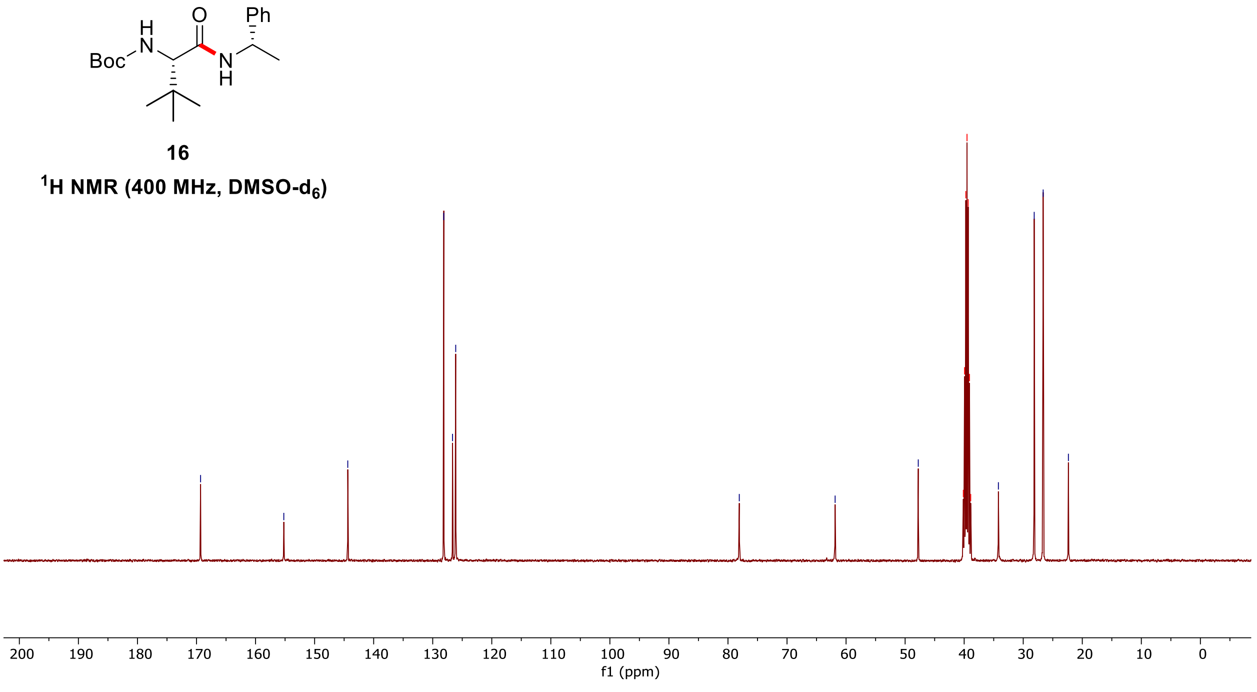

16

^{1}H NMR (400 MHz, DMSO-d$_6$)

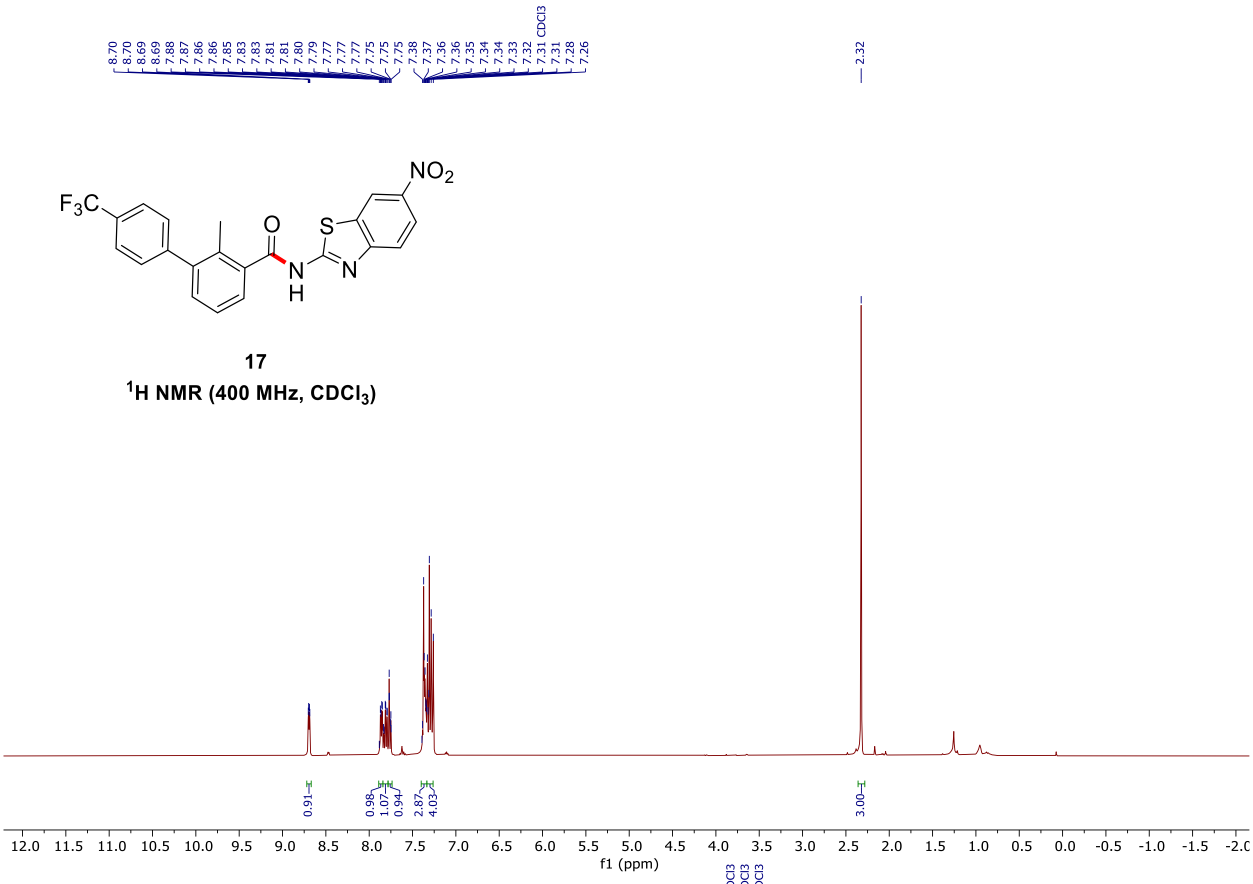

17

^{1}H NMR (400 MHz, $CDCl_3$)

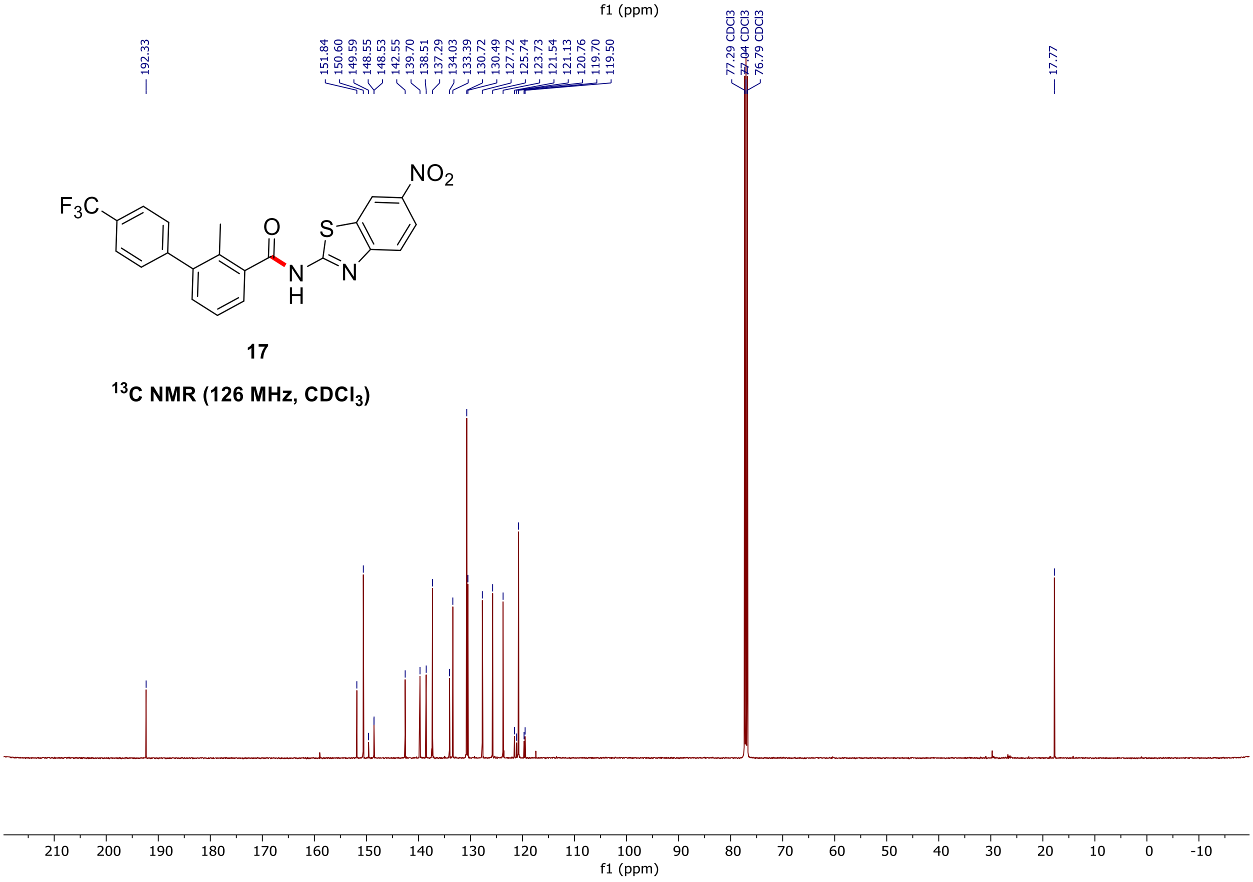

17

^{13}C NMR (126 MHz, $CDCl_3$)

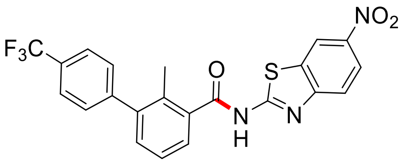

17

^{19}F NMR (376 MHz, $CDCl_3$)

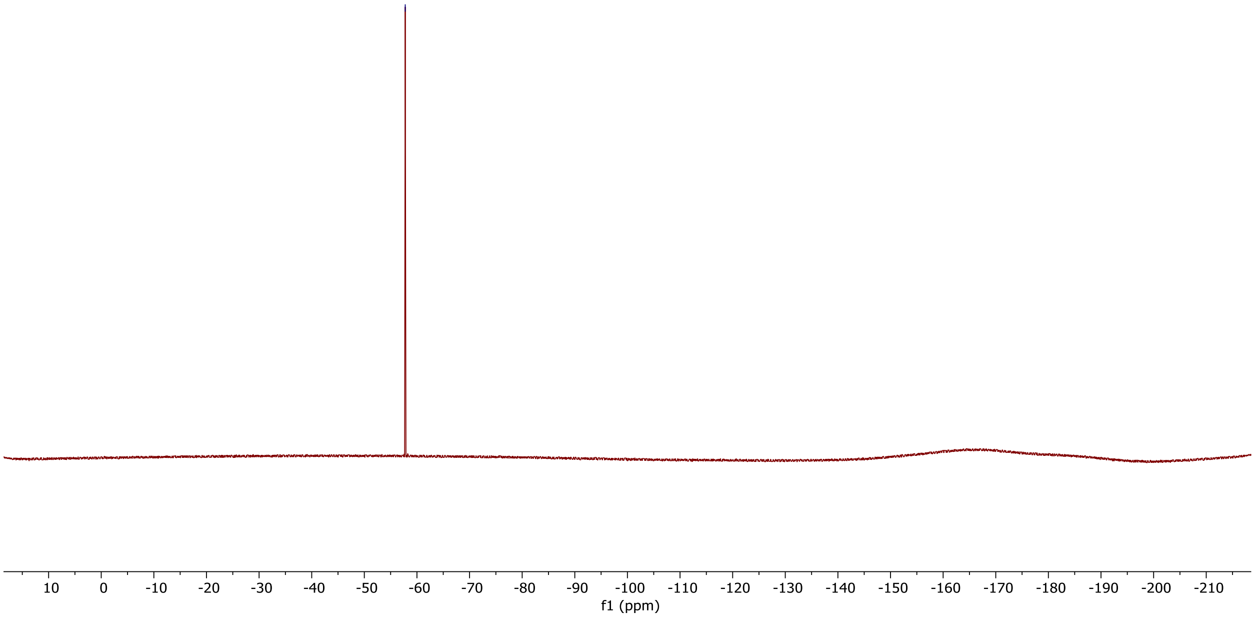

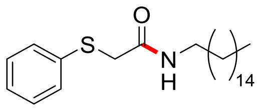

18

^{1}H NMR (500 MHz, $CDCl_3$)

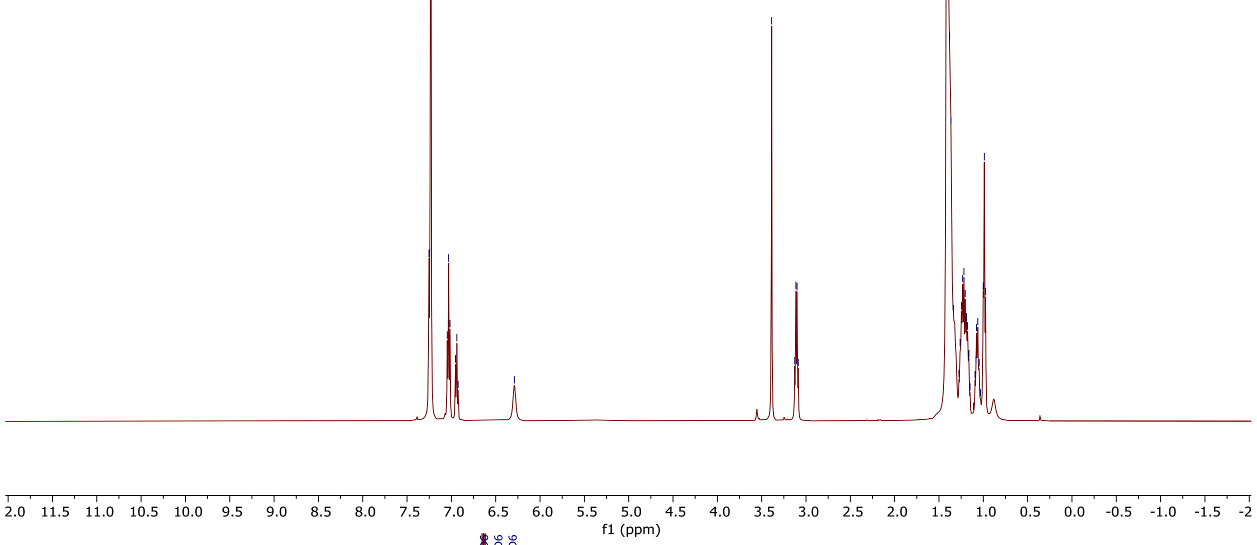

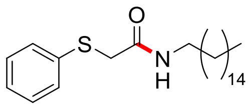

18

^{13}C NMR (126 MHz, $CDCl_3$)

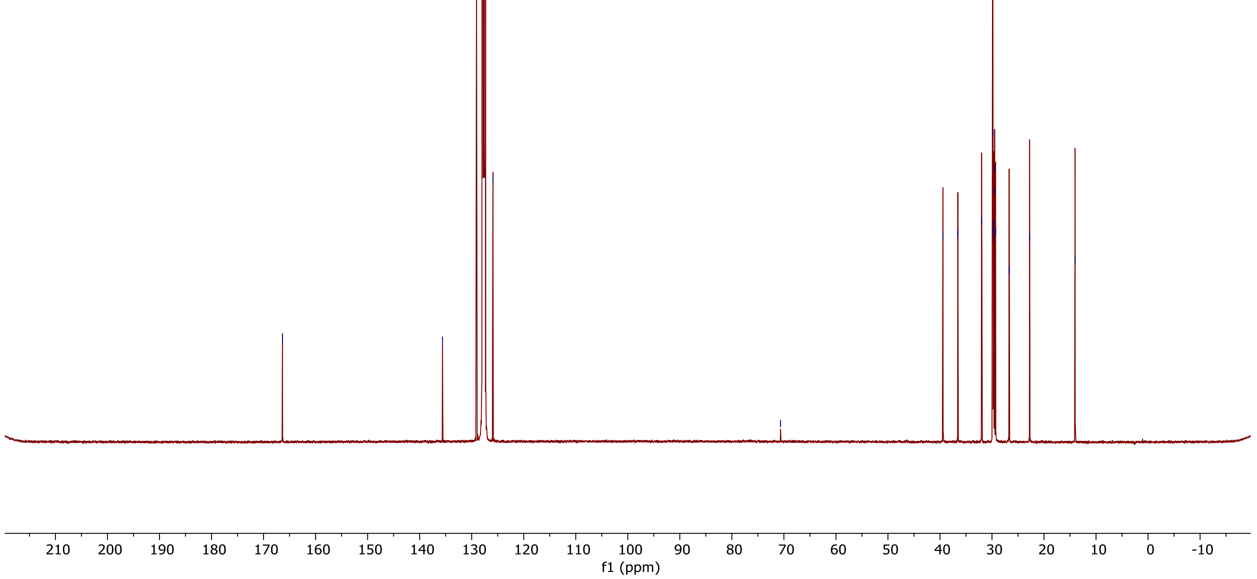

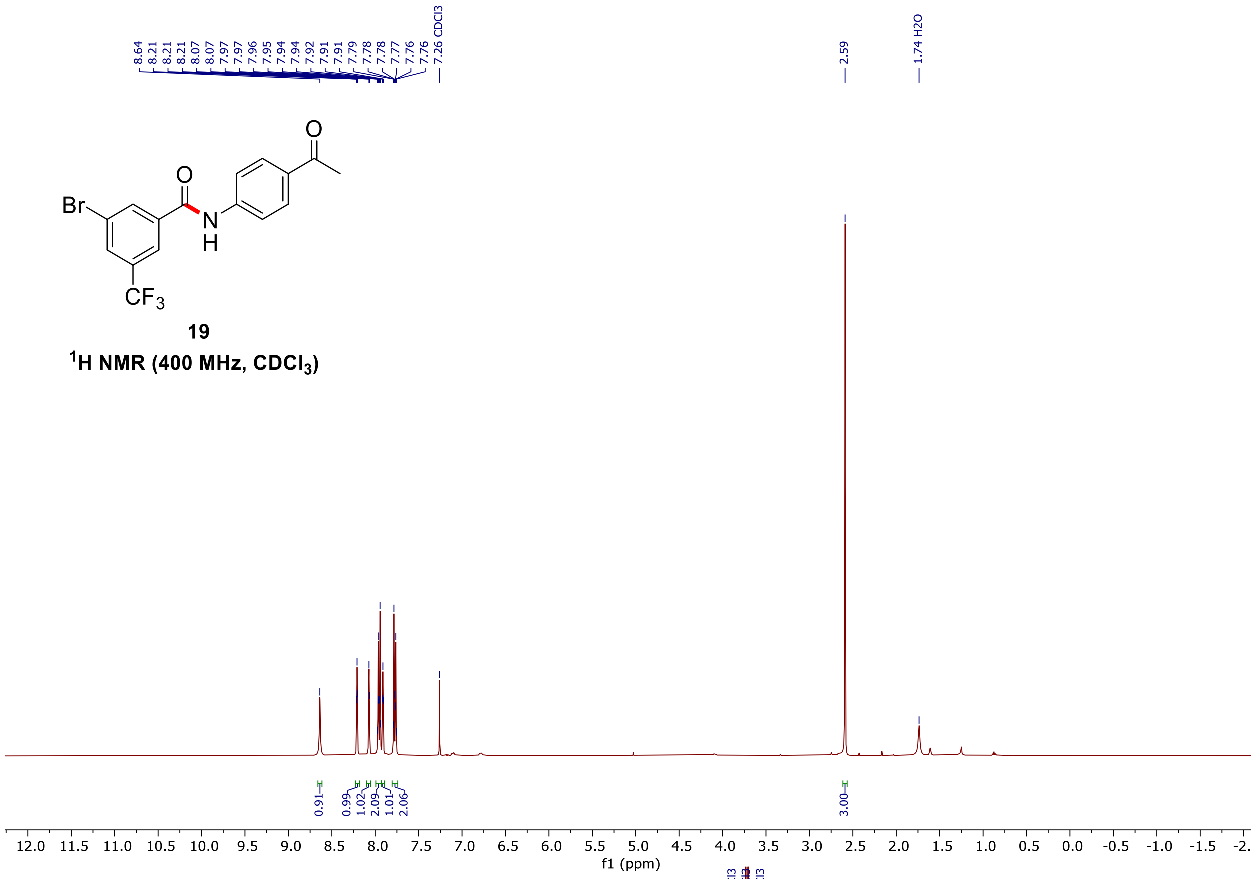

19

^{1}H NMR (400 MHz, $CDCl_3$)

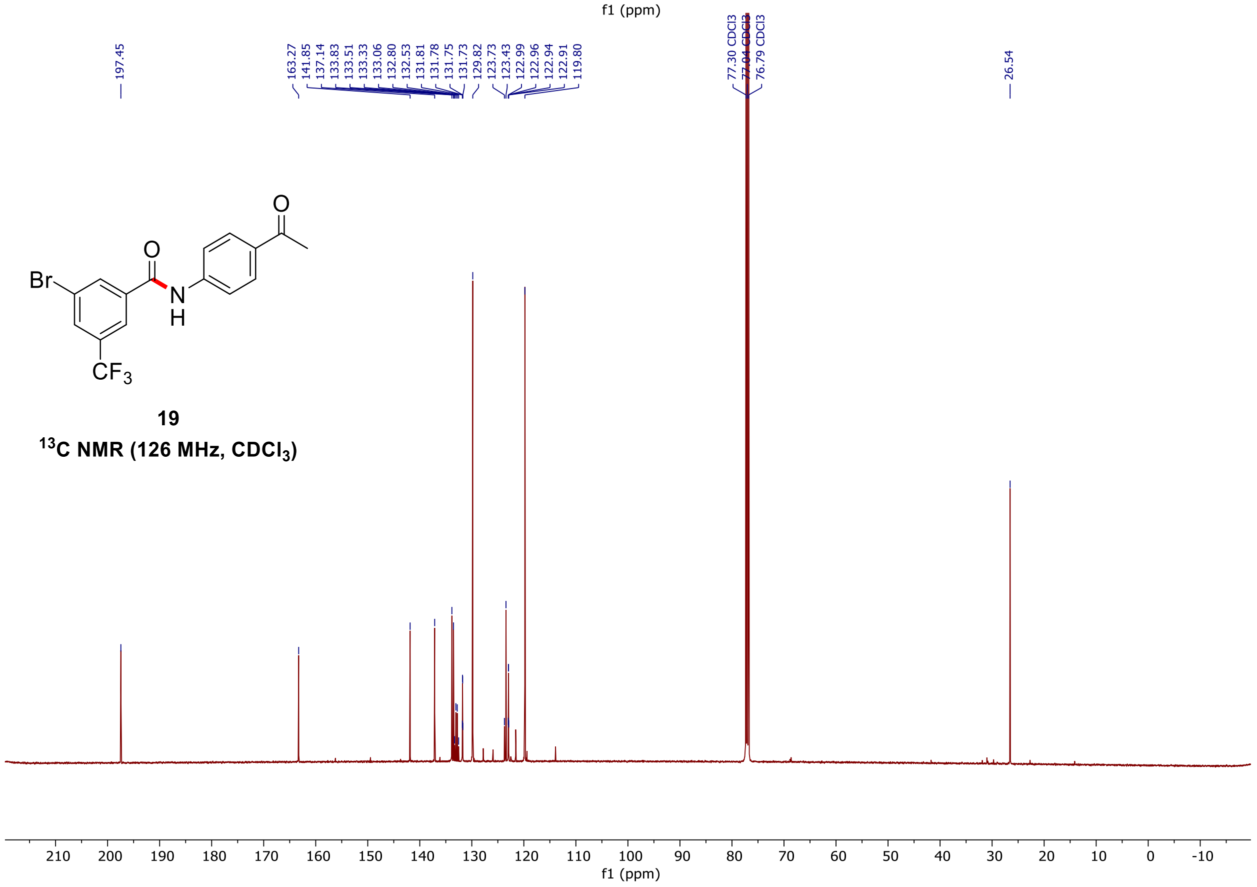

19

^{13}C NMR (126 MHz, $CDCl_3$)

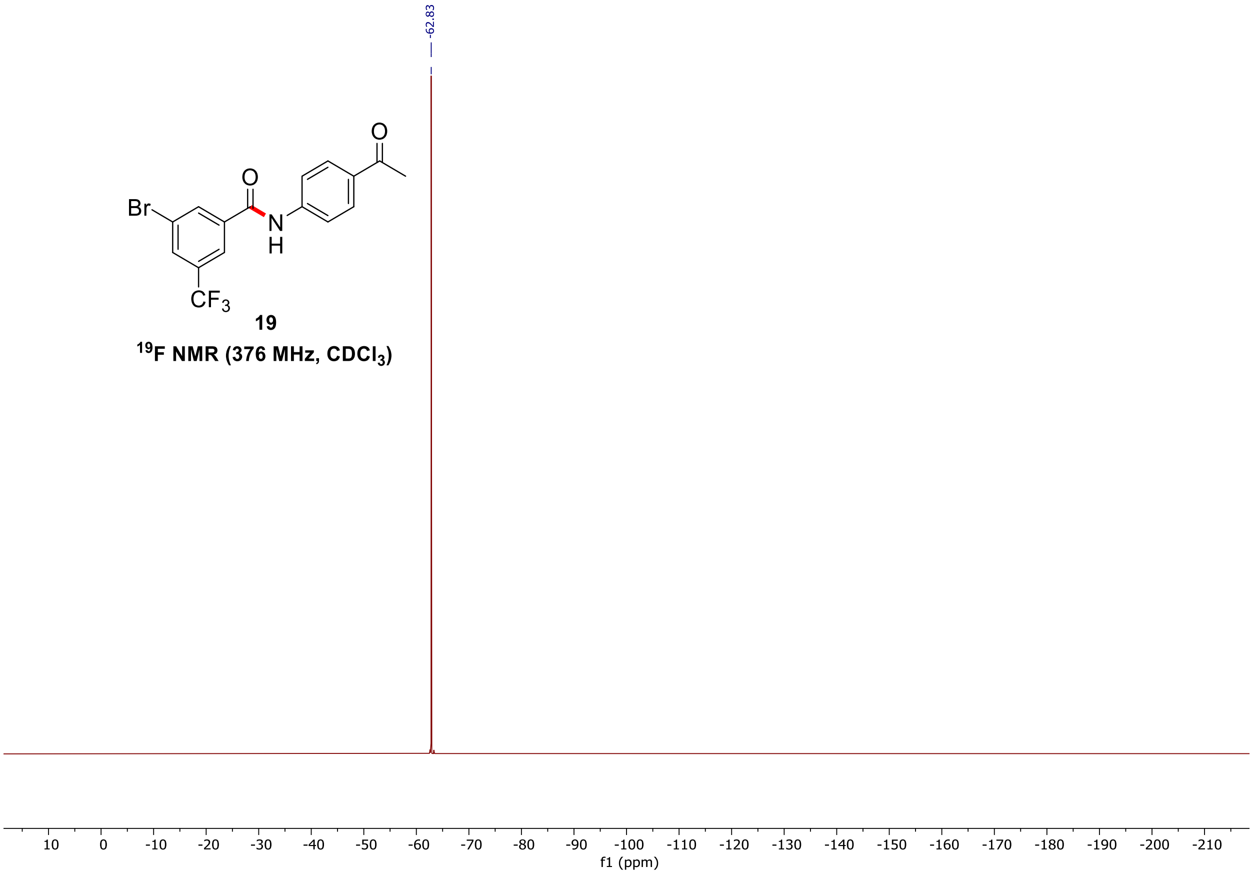

19

^{19}F NMR (376 MHz, $CDCl_3$)

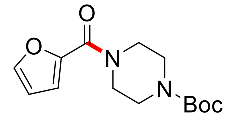

20

^{1}H NMR (400 MHz, $CDCl_3$)

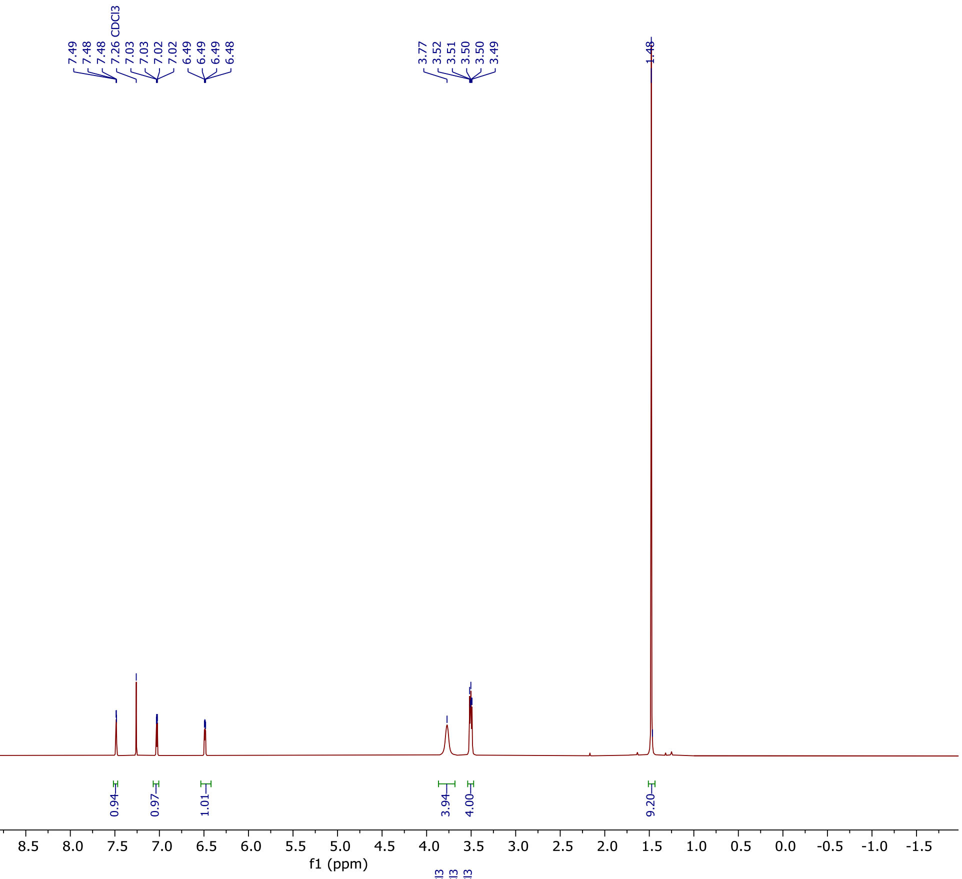

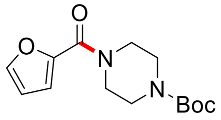

20

^{13}C NMR (126 MHz, $CDCl_3$)

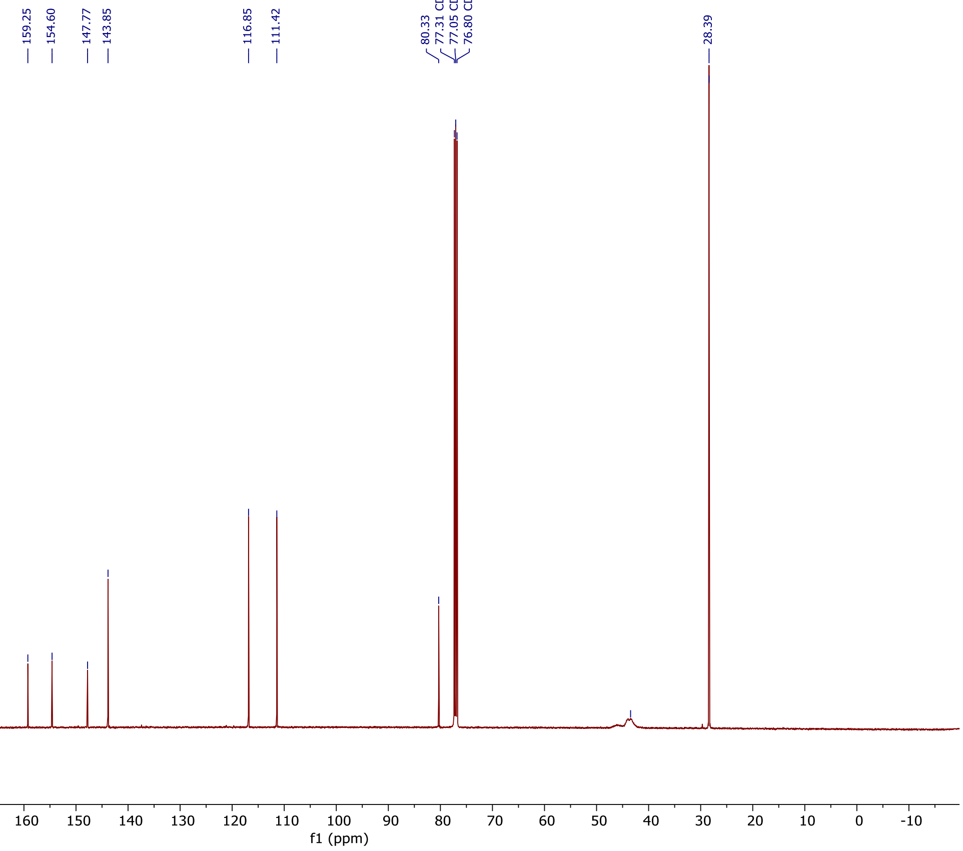

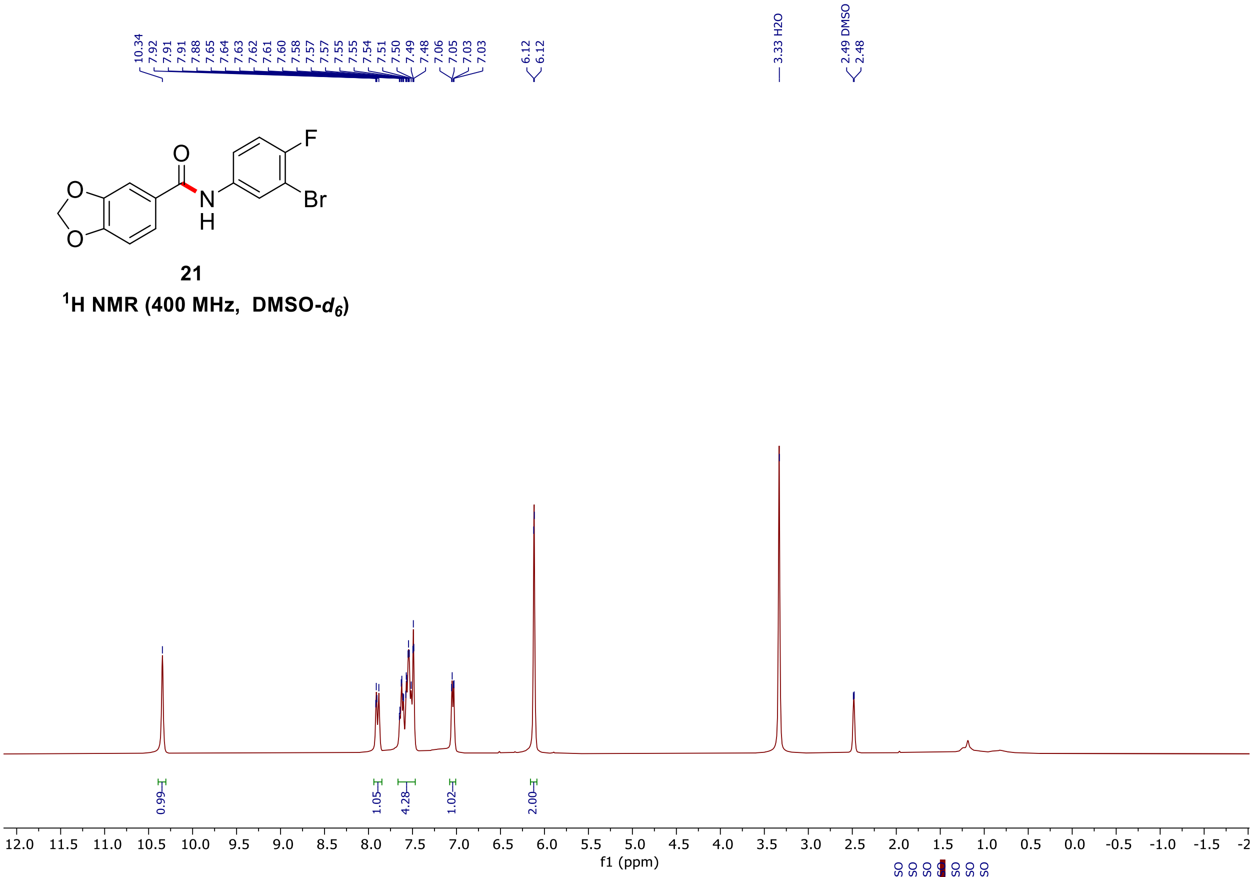
10.34
7.92
7.91
7.91
7.88
7.65
7.64
7.63
7.62
7.61
7.60
7.58
7.57
7.57
7.55
7.55
7.54
7.51
7.50
7.49
7.48
7.06
7.05
7.03
7.03
6.12
6.12
3.33 H2O
2.49 DMSO
2.48
O
F
O
N
H
Br
O
21
^{1}H NMR (400 MHz, DMSO-d_6)
0.99
1.05
4.28
1.02
2.00
12.0 11.5 11.0 10.5 10.0 9.5 9.0 8.5 8.0 7.5 7.0 6.5 6.0 5.5 5.0 4.5 4.0 3.5 3.0 2.5 2.0 1.5 1.0 0.5 0.0 -0.5 -1.0 -1.5 -2
f1 (ppm)

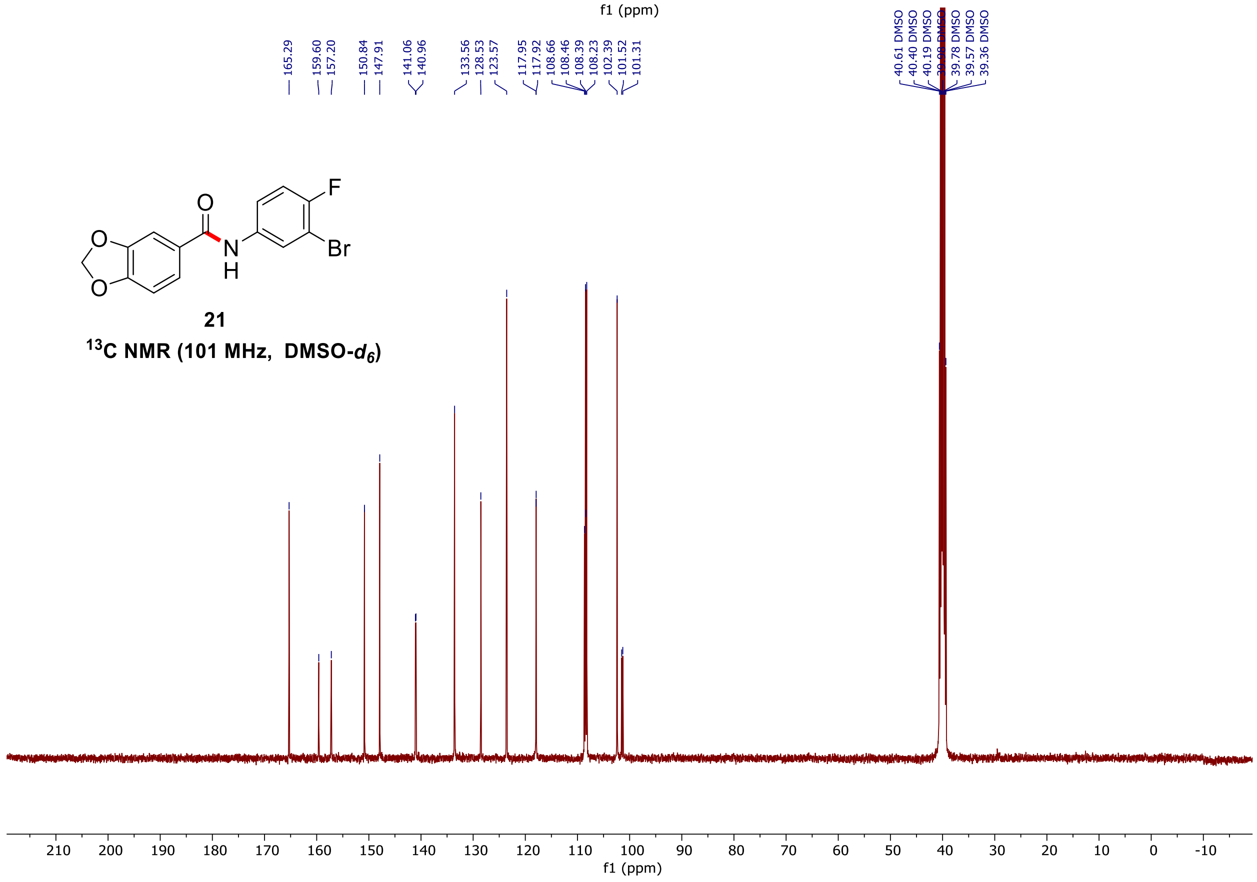
165.29
159.60
157.20
150.84
147.91
141.06
140.96
133.56
128.53
123.57
117.95
117.92
108.66
108.46
108.39
108.23
102.39
101.52
101.31
40.61 DMSO
40.40 DMSO
40.19 DMSO
39.78 DMSO
39.57 DMSO
39.36 DMSO
O
F
O
N
H
Br
O
21
^{13}C NMR (101 MHz, DMSO-d_6)
210 200 190 180 170 160 150 140 130 120 110 100 90 80 70 60 50 40 30 20 10 0 -10
f1 (ppm)

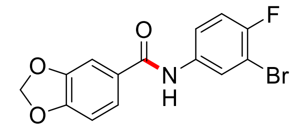

21

^{19}FNMR (376 MHz, DMSO-d_6)

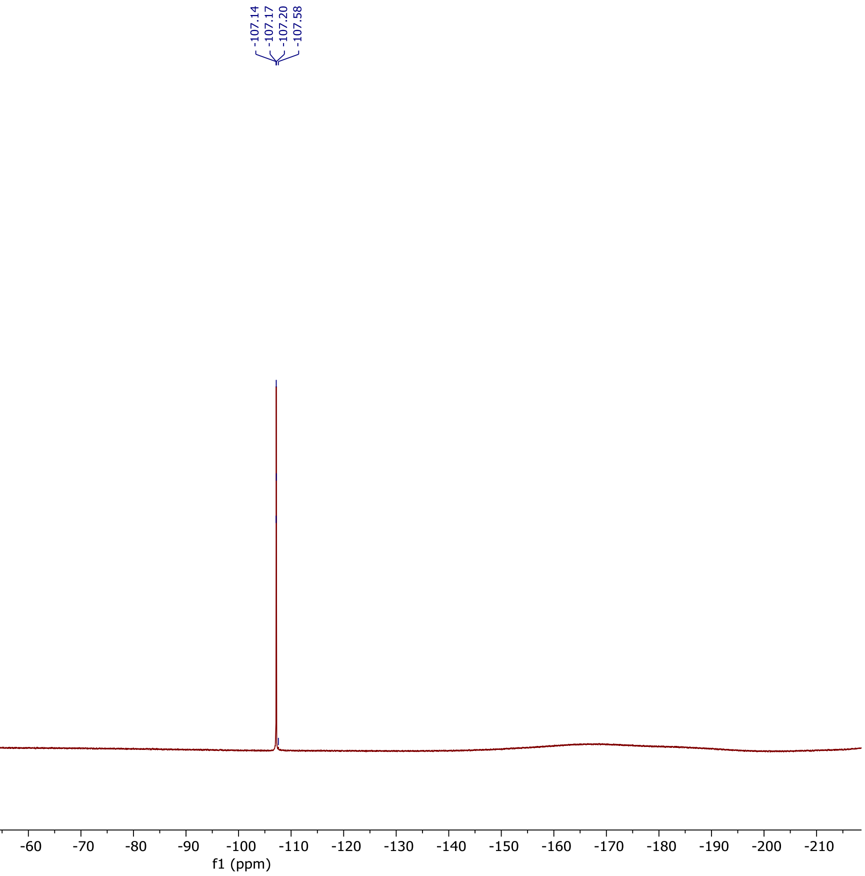

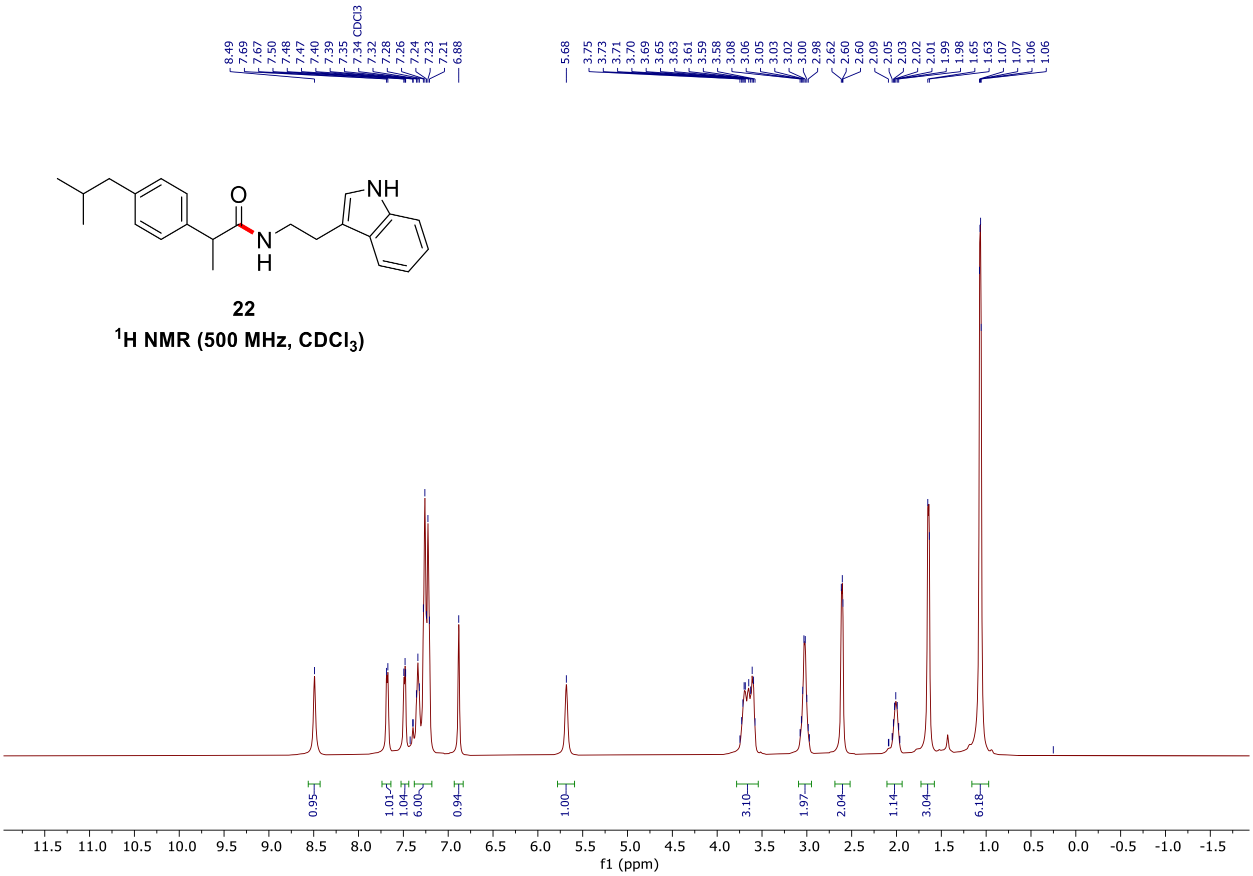

22

^{1}H NMR (500 MHz, CDCl$_3$)

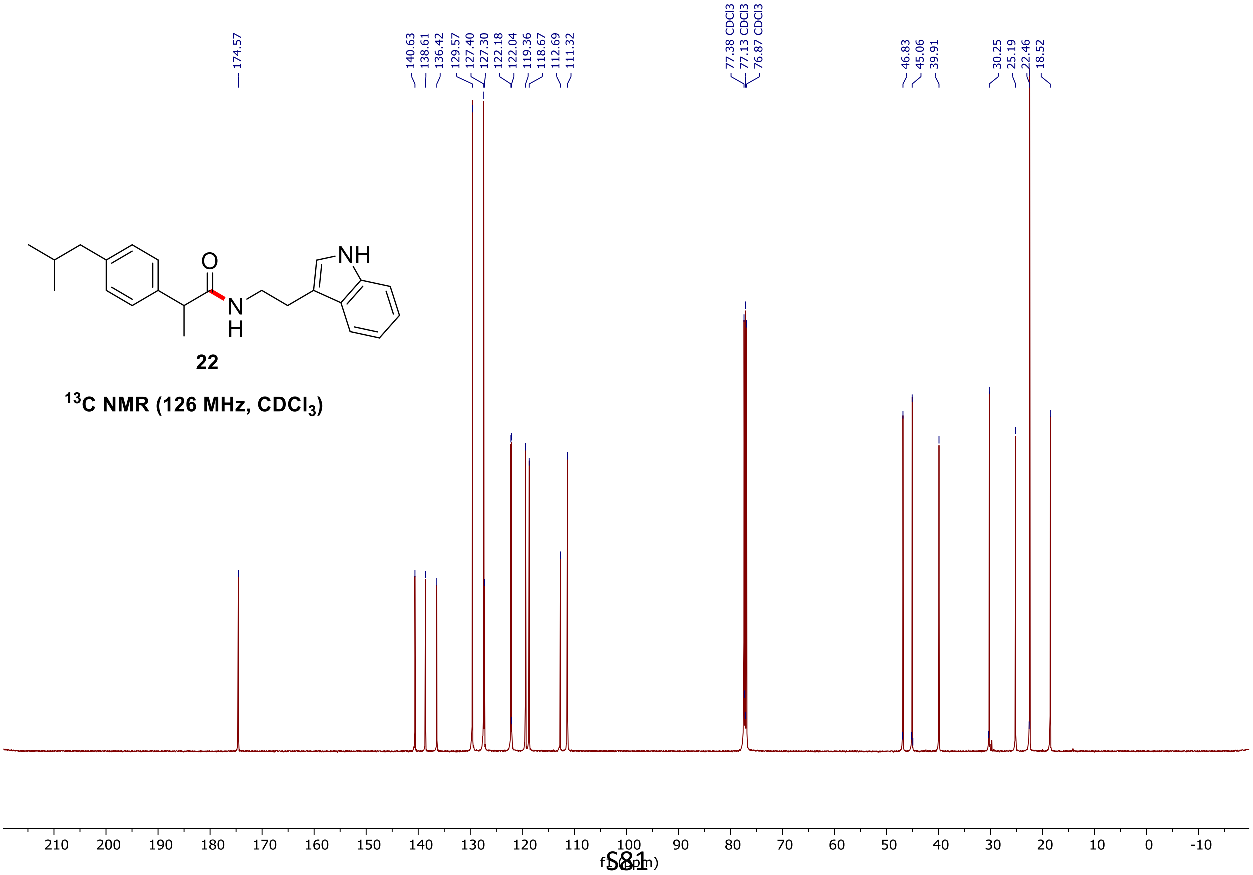

22

^{13}C NMR (126 MHz, CDCl$_3$)

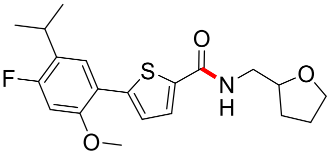

23

^{1}H NMR (500 MHz, CDCl$_3$)

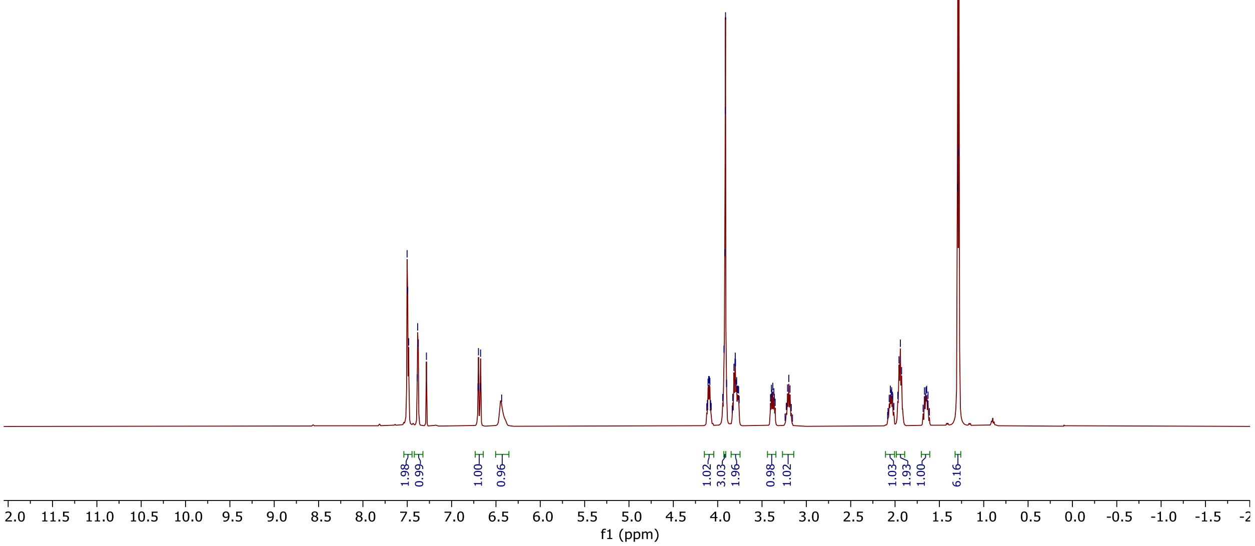

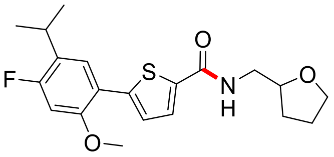

23

^{13}C NMR (126 MHz, CDCl$_3$)

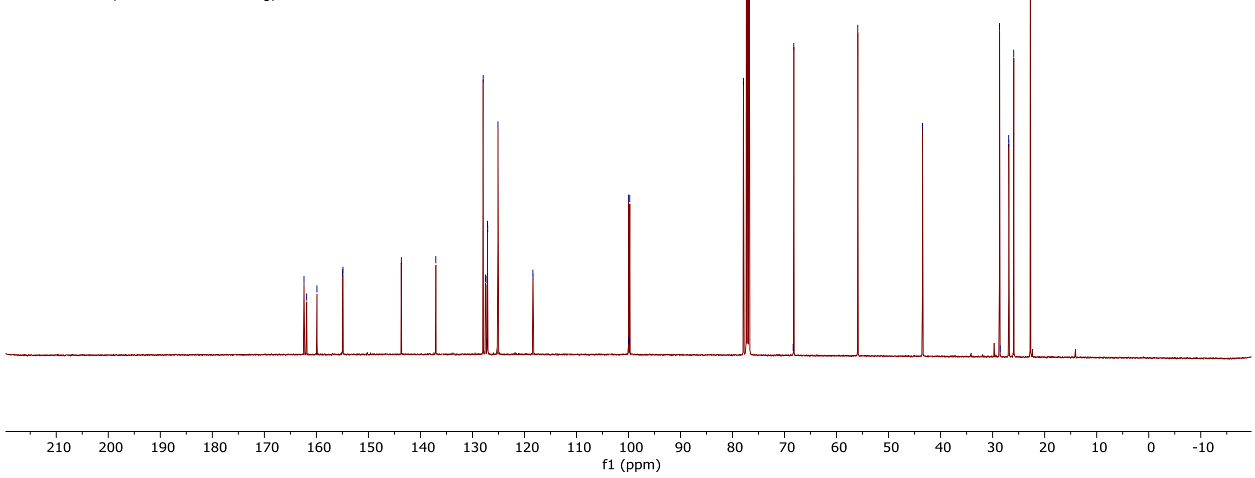

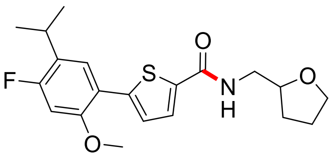

23

^{19}F NMR (500 MHz, $CDCl_3$)

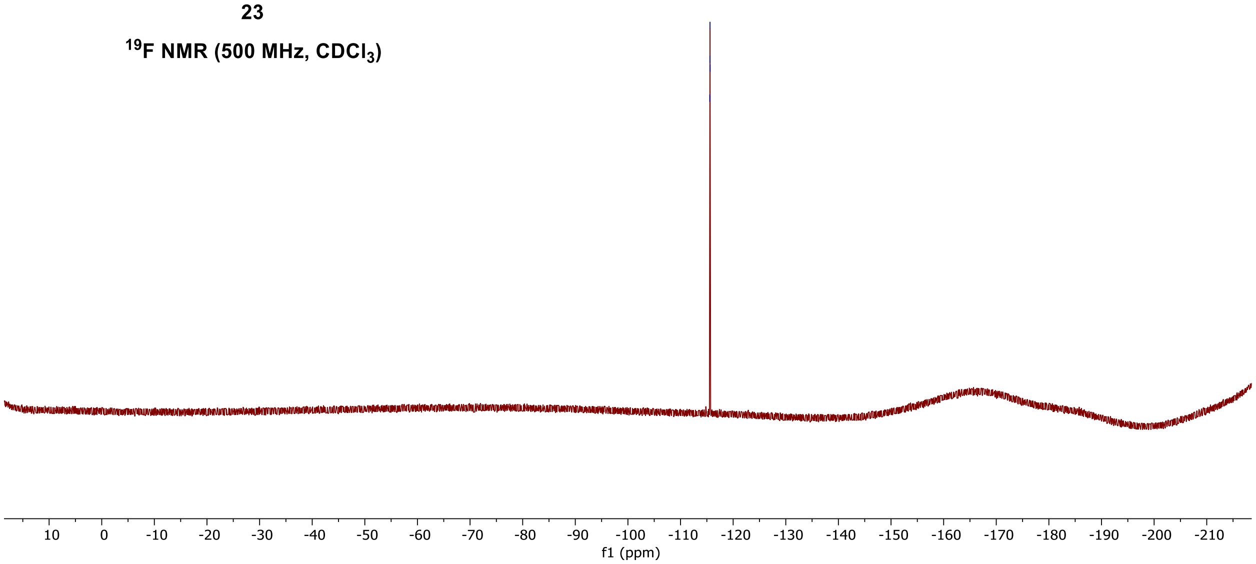

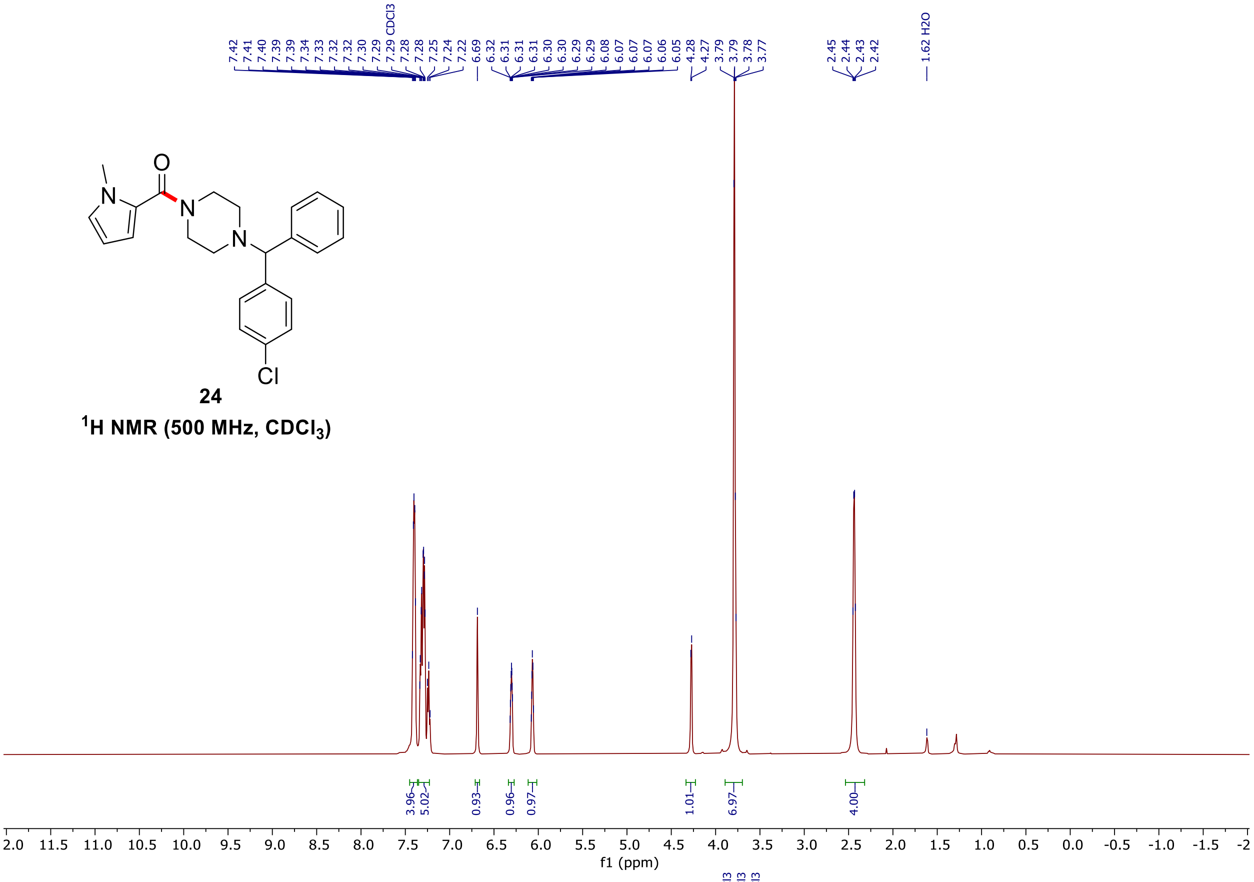
7.42
7.41
7.40
7.39
7.39
7.34
7.33
7.32
7.32
7.30
7.29
7.29 CDCl3
7.28
7.28
7.25
7.24
7.22
6.69
6.32
6.31
6.31
6.31
6.30
6.30
6.29
6.08
6.07
6.07
6.07
6.06
6.05
4.28
4.27
3.79
3.79
3.78
3.77
2.45
2.44
2.43
2.42
1.62 H2O
24
¹H NMR (500 MHz, CDCl₃)
3.96
5.02
0.93
0.96
0.97
1.01
6.97
4.00
f1 (ppm)

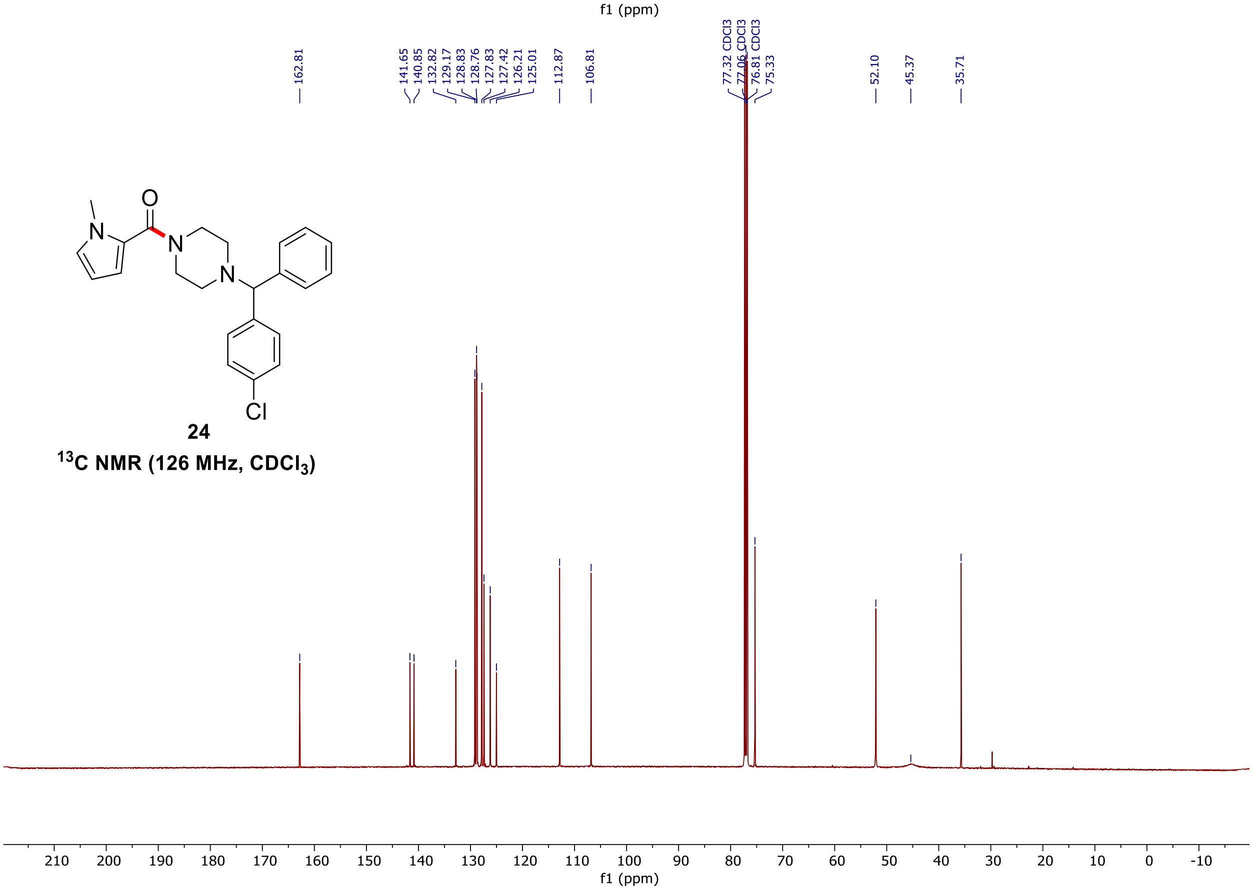
162.81
141.65
140.85
132.82
129.17
128.83
128.76
127.83
127.42
126.21
125.01
112.87
106.81
77.32 CDCl3
77.06 CDCl3
76.81 CDCl3
75.33
52.10
45.37
35.71
24
¹³C NMR (126 MHz, CDCl₃)
f1 (ppm)

25

^{1}H NMR (500 MHz, CDCl$_3$)

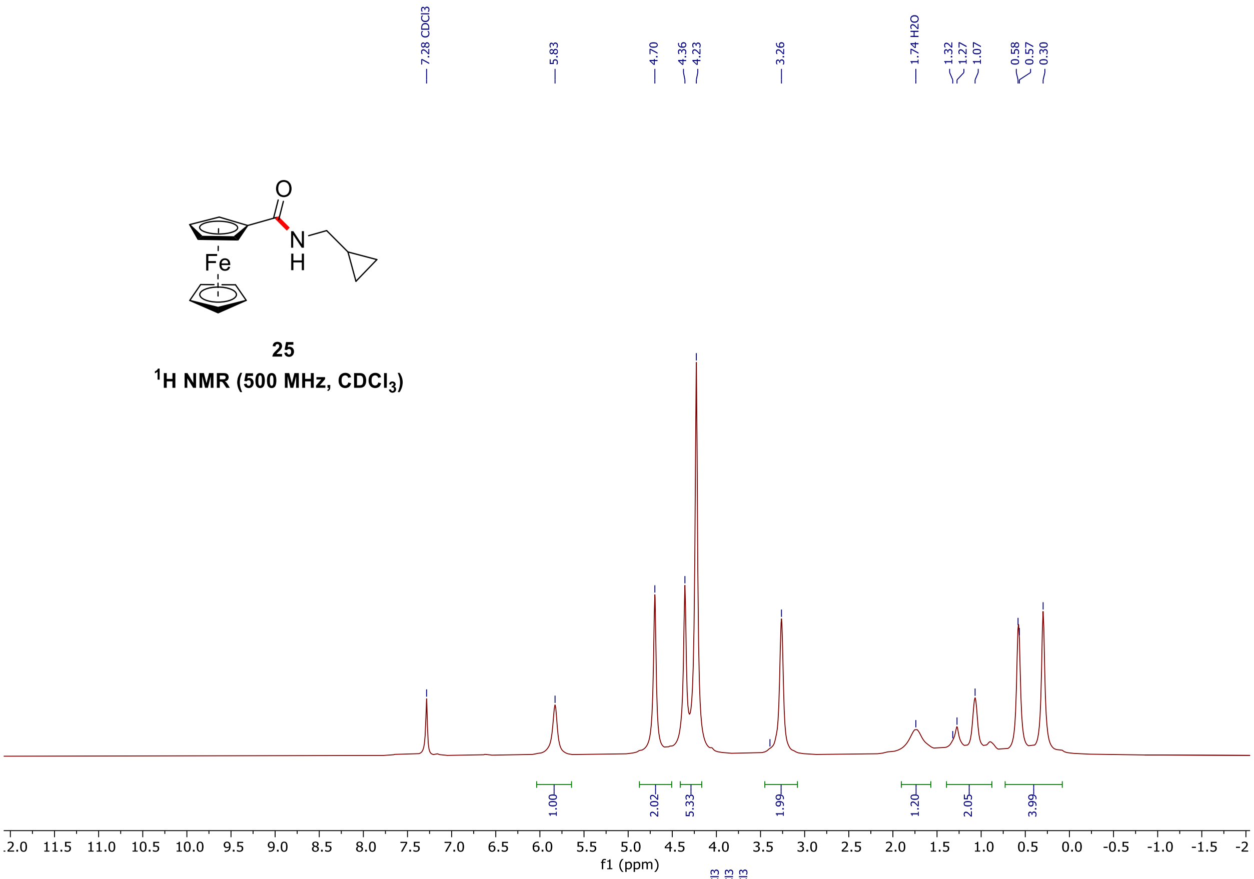

25

^{13}C NMR (126 MHz, CDCl$_3$)

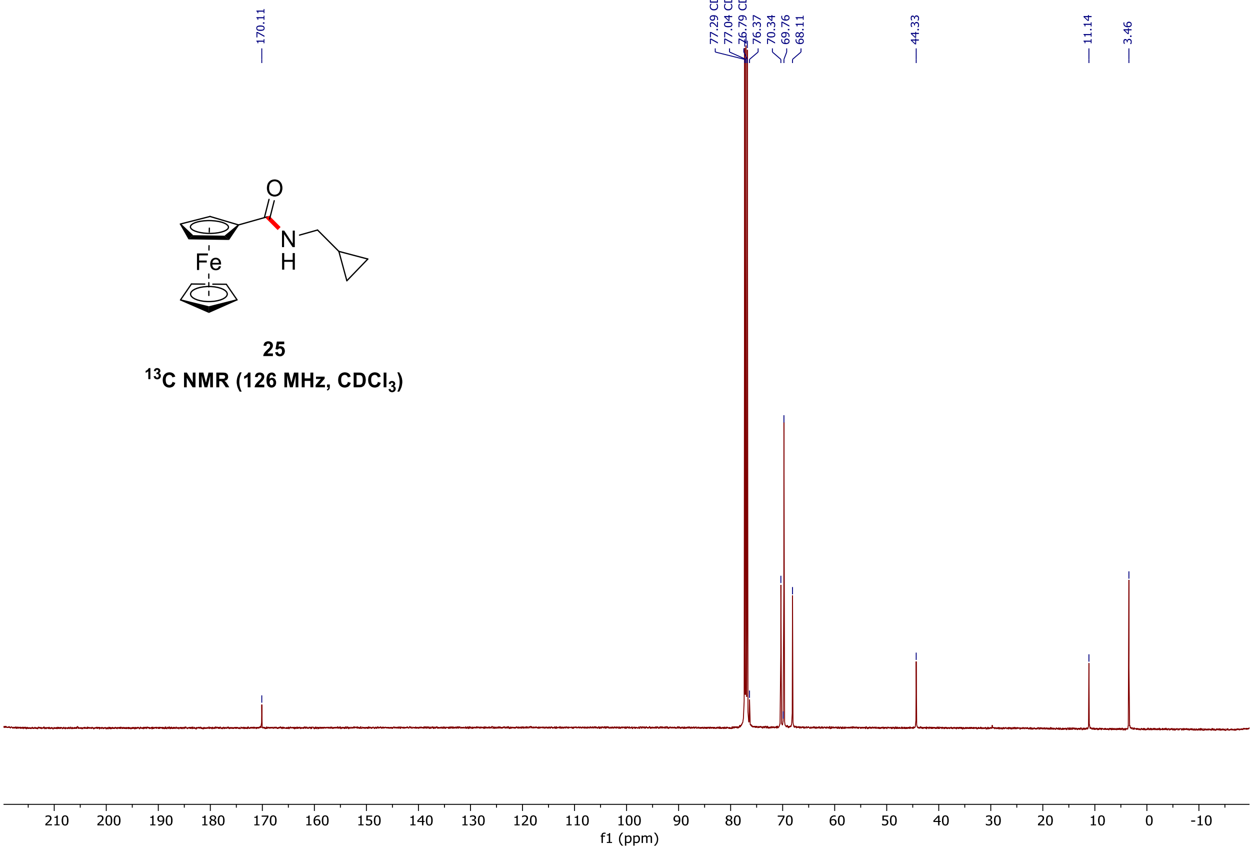

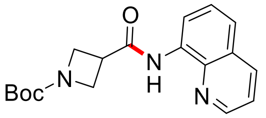

26

^{1}H NMR (500 MHz, $CDCl_3$)

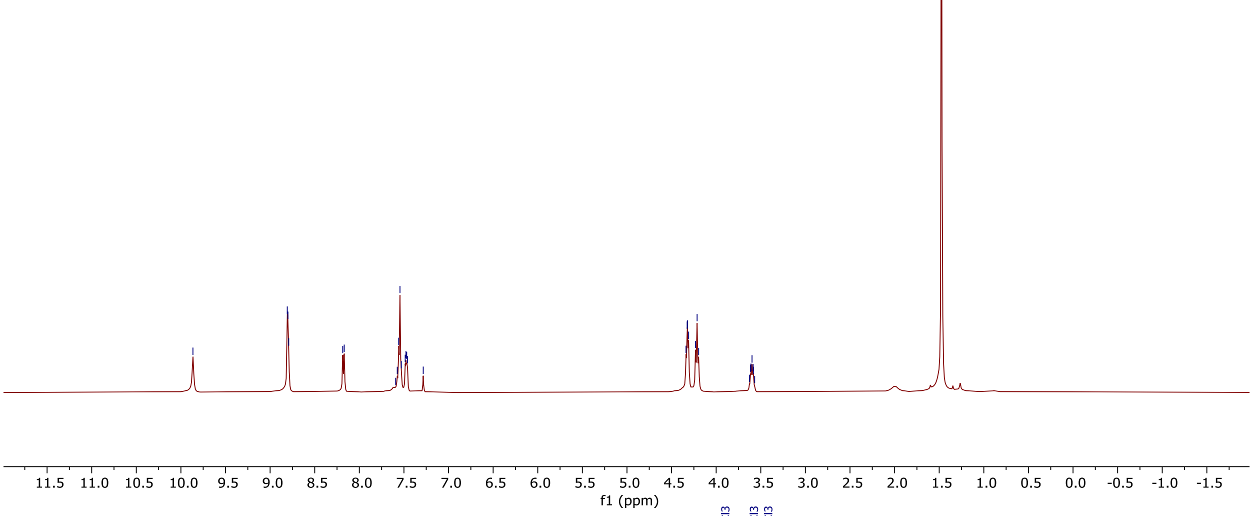

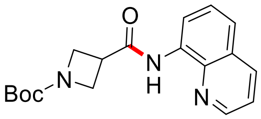

26

^{13}C NMR (126 MHz, $CDCl_3$)

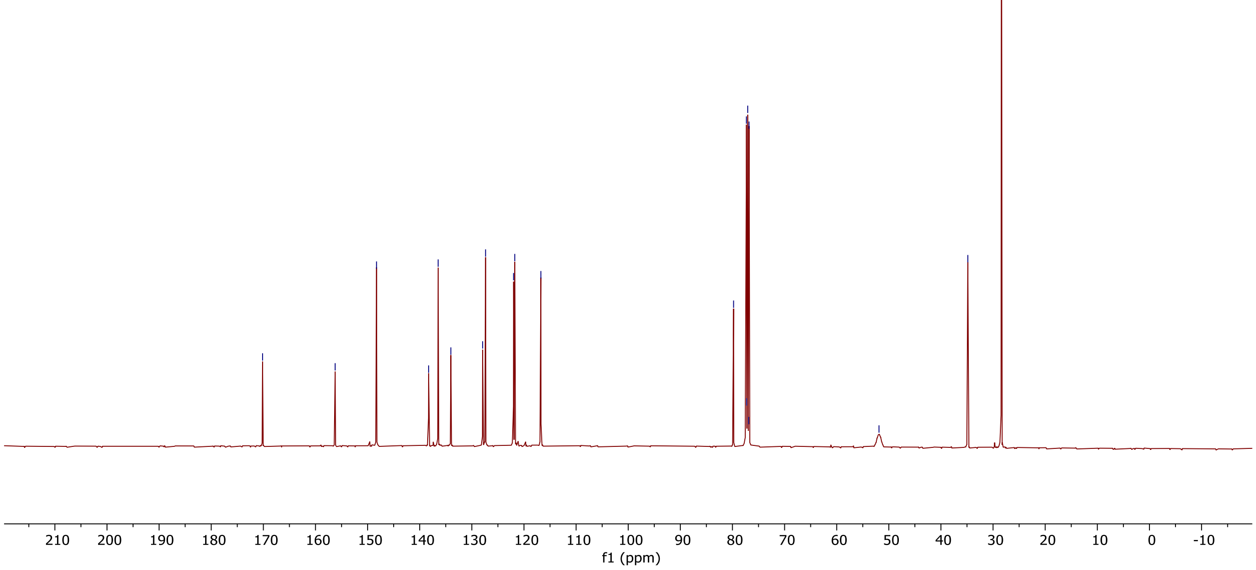

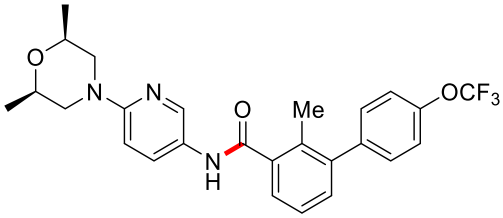

27

^{1}H NMR (400 MHz, DMSO-d$_6$)

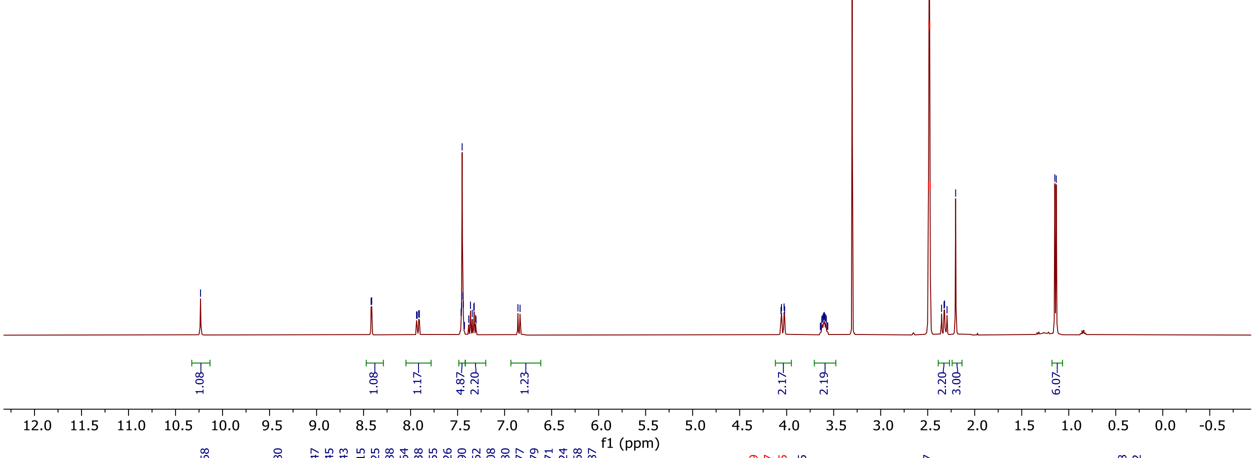

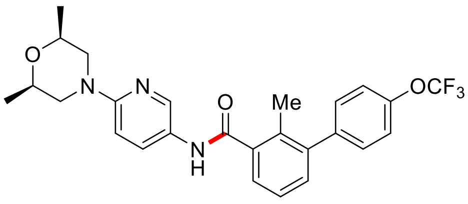

27

^{13}C NMR (101 MHz, CDCl$_3$)

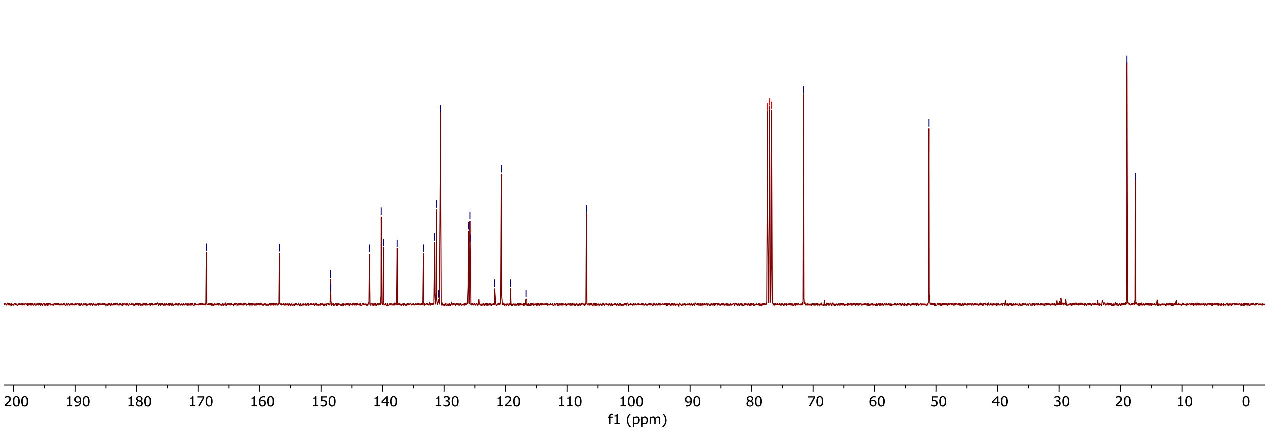

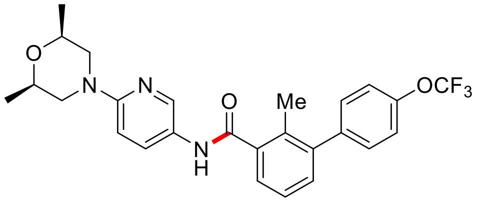

27

^{13}F NMR (376 MHz, CDCl$_3$)

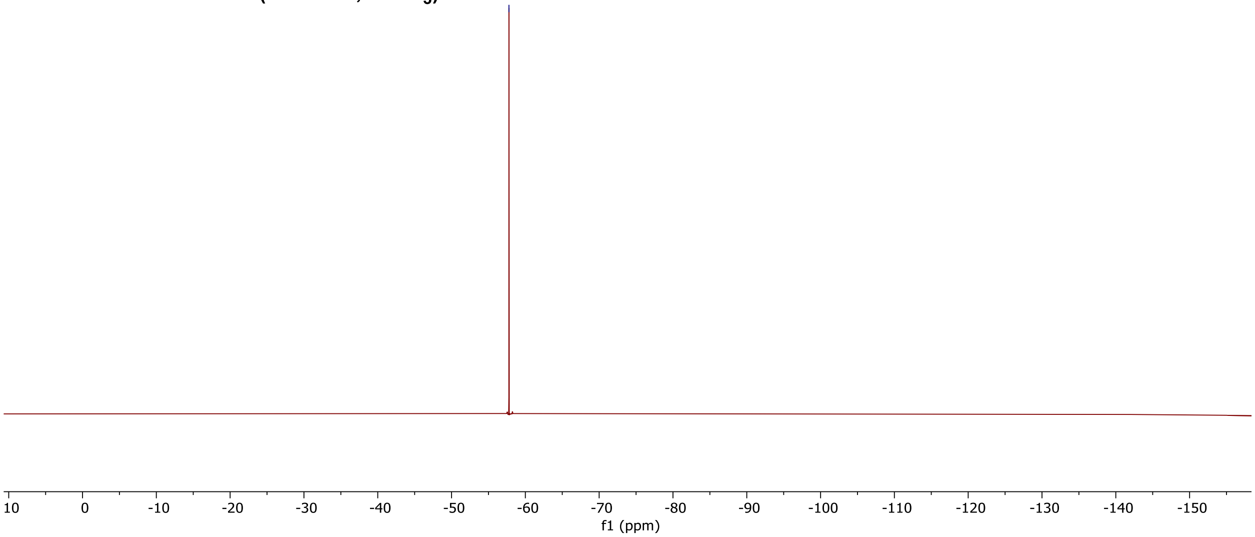

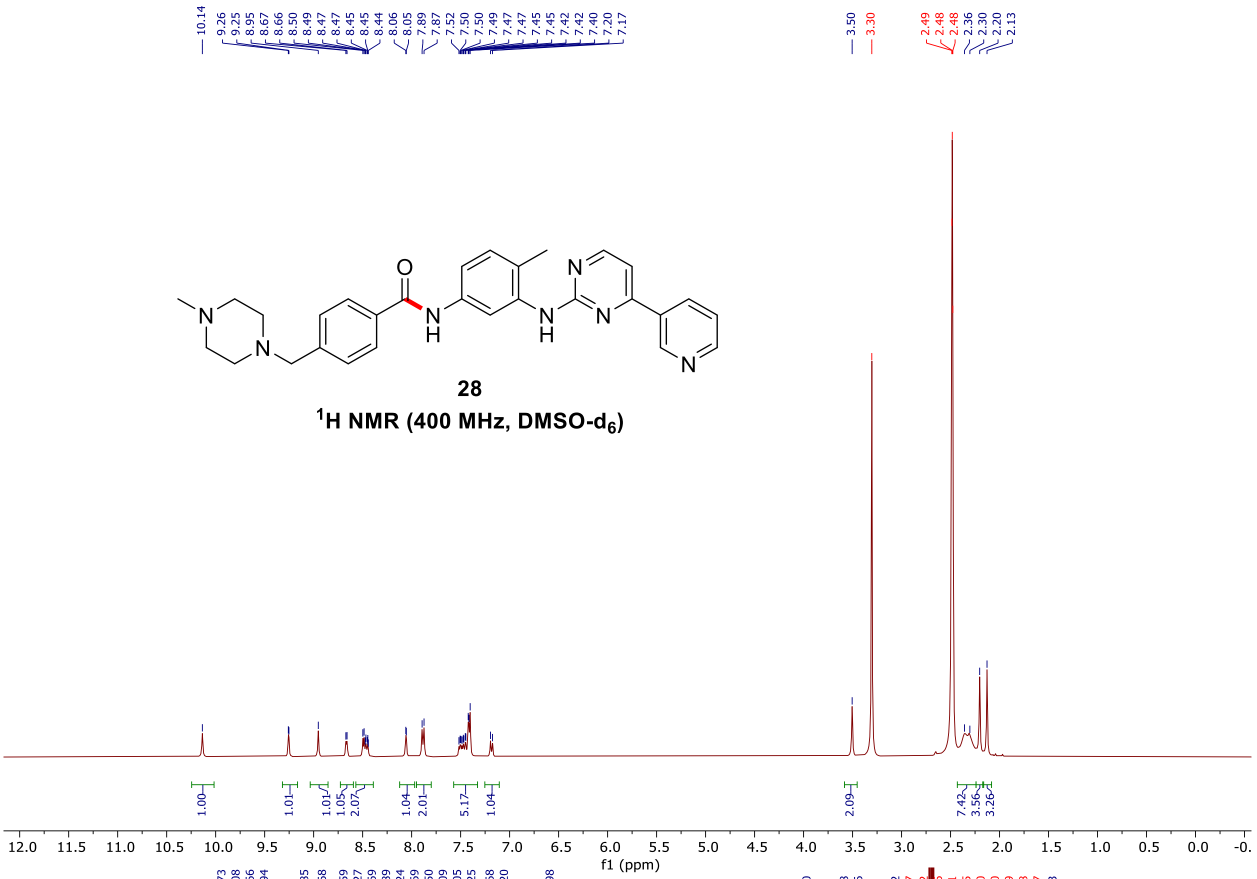
10.14 9.26 9.25 8.95 8.67 8.66 8.50 8.49 8.47 8.47 8.45 8.45 8.44 8.06 8.05 7.89 7.87 7.52 7.50 7.50 7.49 7.47 7.47 7.45 7.45 7.42 7.42 7.40 7.20 7.17
3.50 3.30
2.49 2.48 2.48 2.36 2.30 2.20 2.13
28
^{1}H NMR (400 MHz, DMSO-d$_6$)
1.00 1.01 1.01 1.05 2.07 1.04 2.01 5.17 1.04 2.09 7.42 3.56 3.26
f1 (ppm)

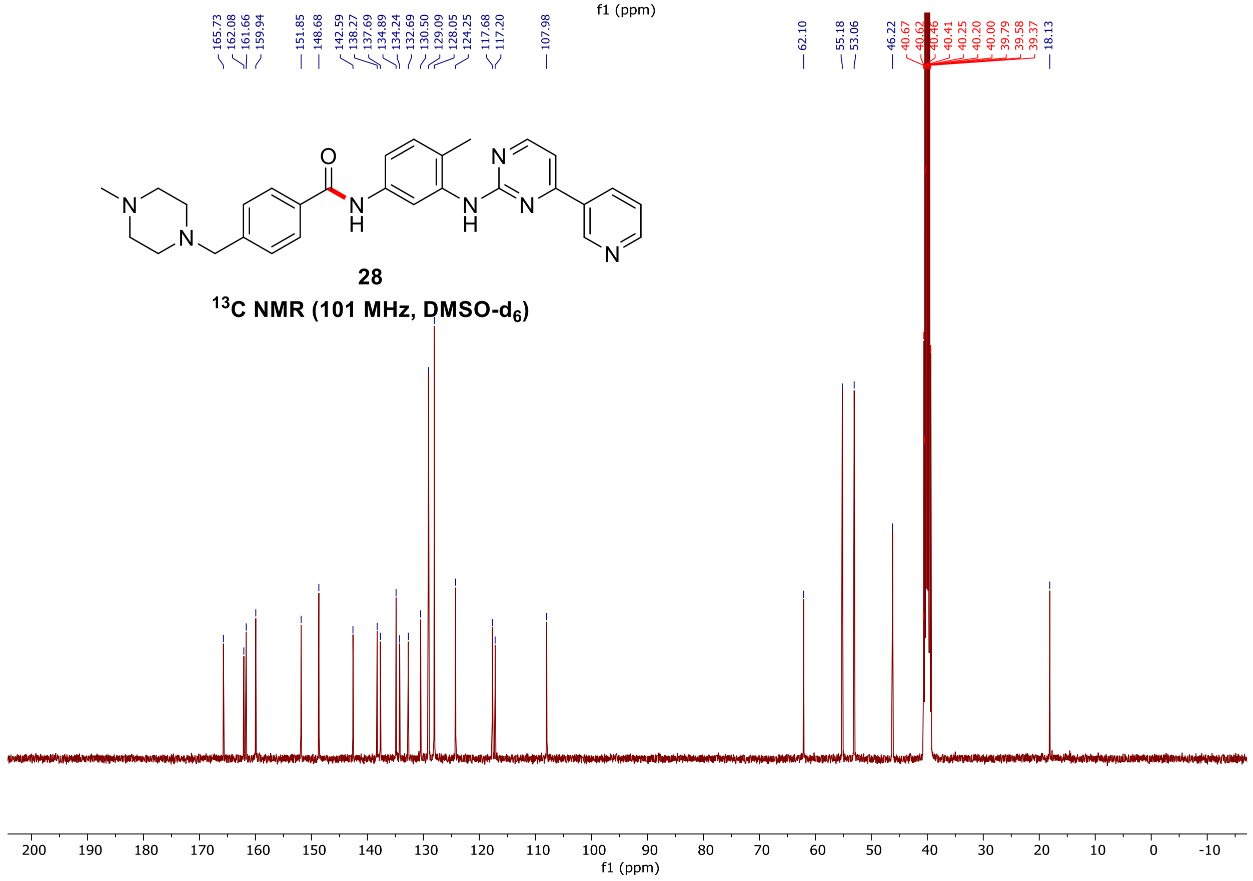
165.73 162.08 161.66 159.94 151.85 148.68 142.59 138.27 137.69 134.89 134.24 132.69 130.50 129.09 128.05 124.25 117.68 117.20 107.98
62.10 55.18 53.06 46.22 40.67 40.62 40.46 40.41 40.25 40.20 40.00 39.79 39.58 39.37 18.13
28
^{13}C NMR (101 MHz, DMSO-d$_6$)
f1 (ppm)

29

^{1}H NMR (500 MHz, CDCl$_3$)

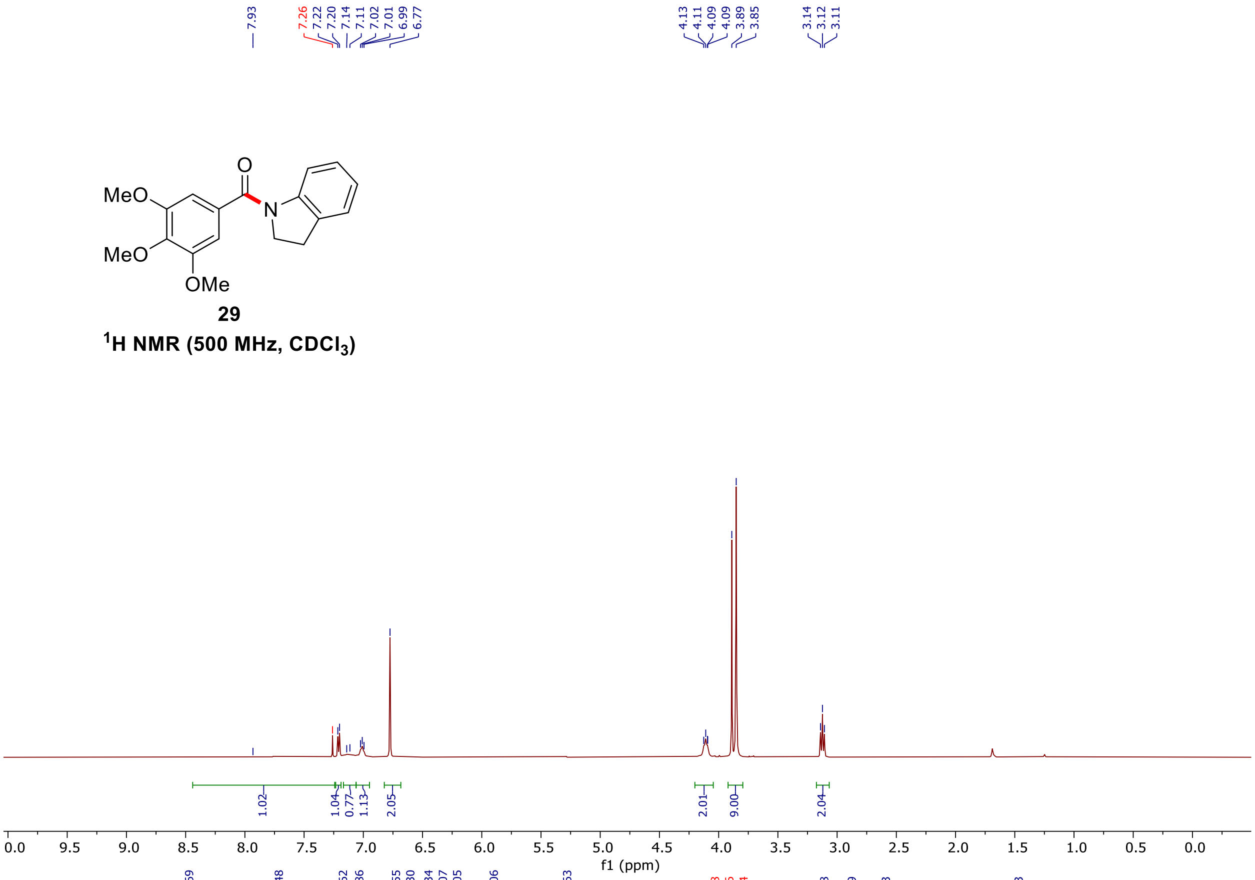

29

^{13}C NMR (101 MHz, CDCl$_3$)

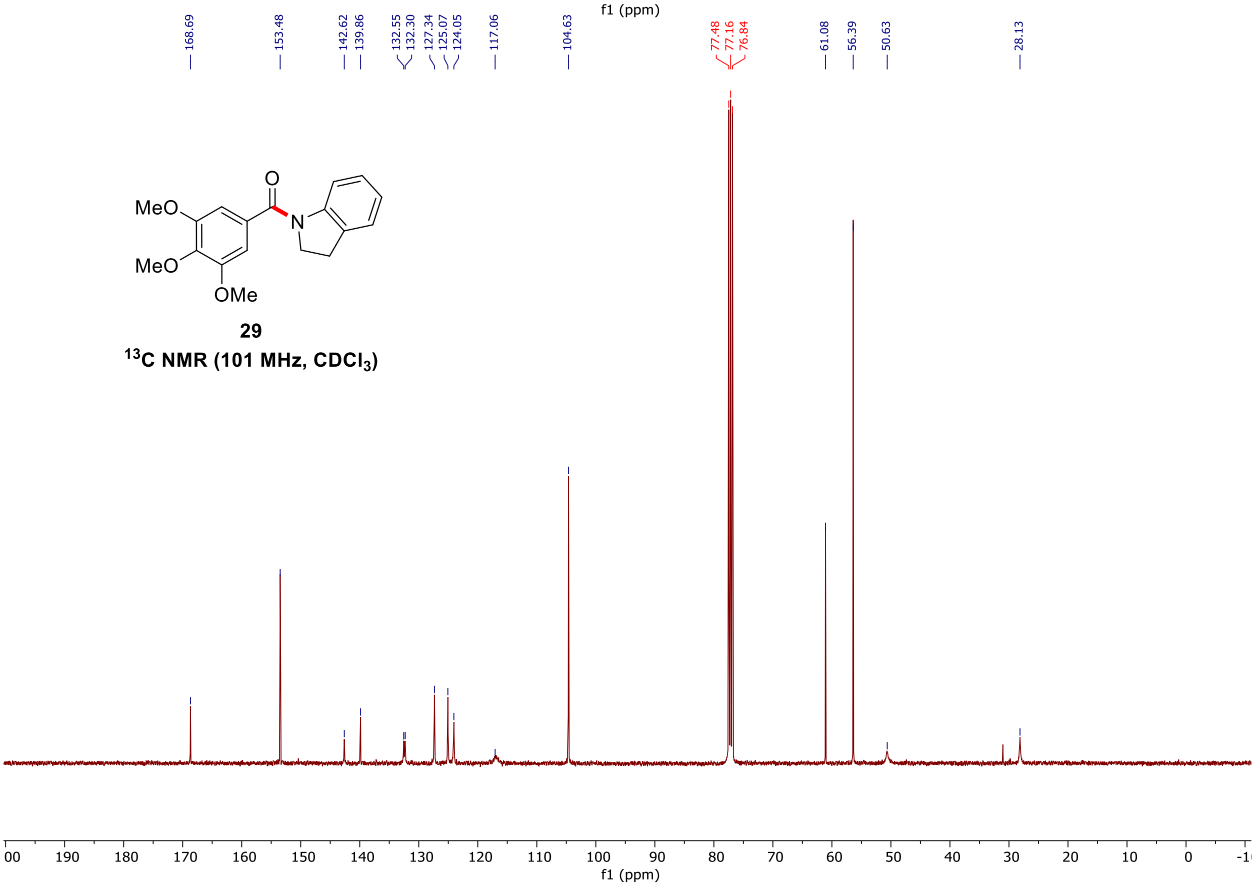

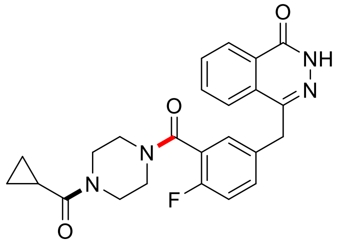

30

^{1}H NMR (400 MHz, CDCl$_3$)

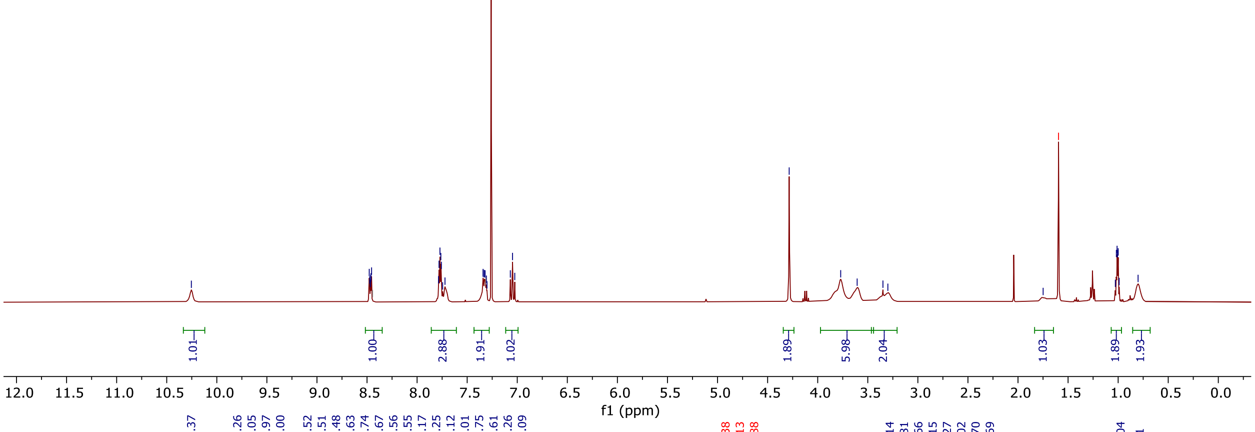

30

^{13}C NMR (126 MHz, CDCl$_3$)

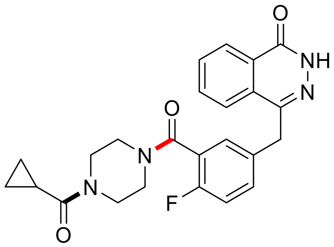

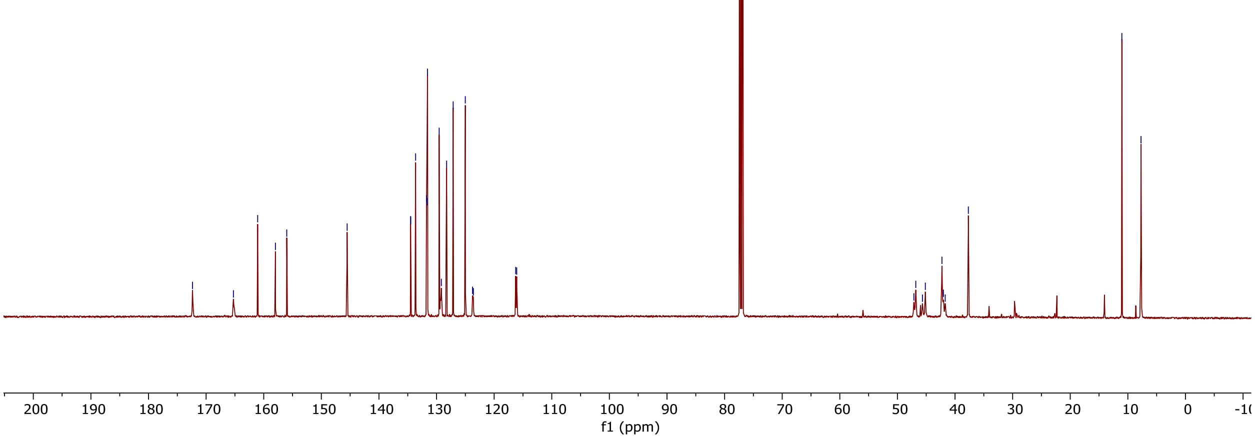

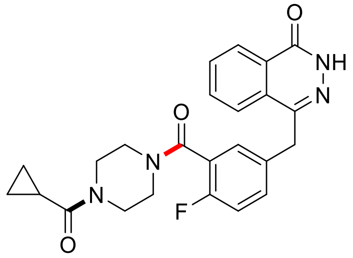

30

^{19}F NMR (376 MHz, $CDCl_3$)

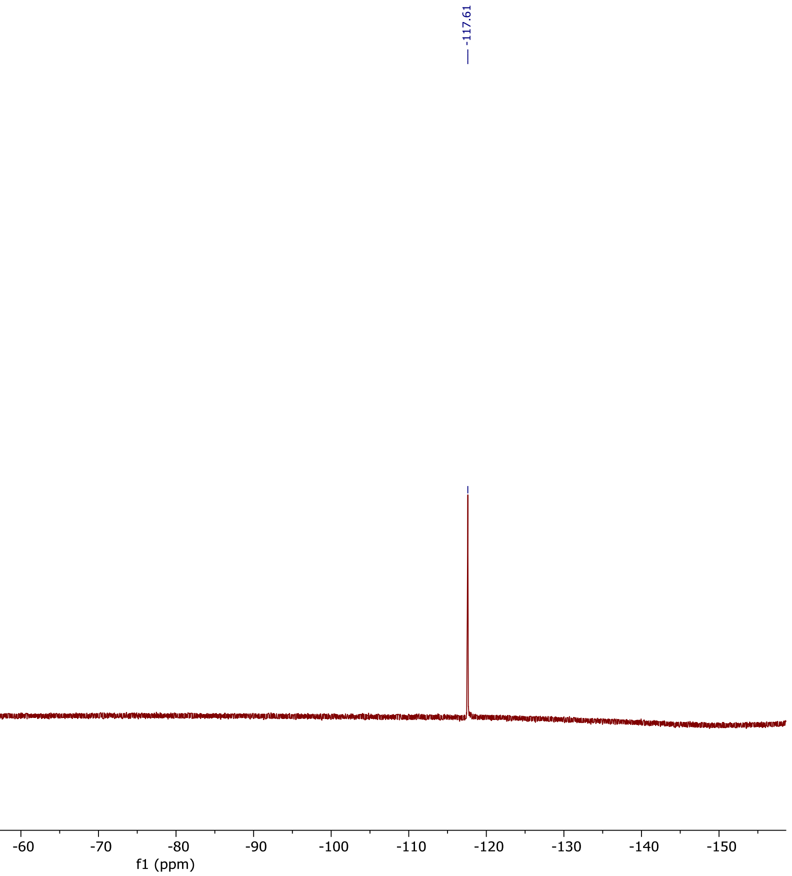

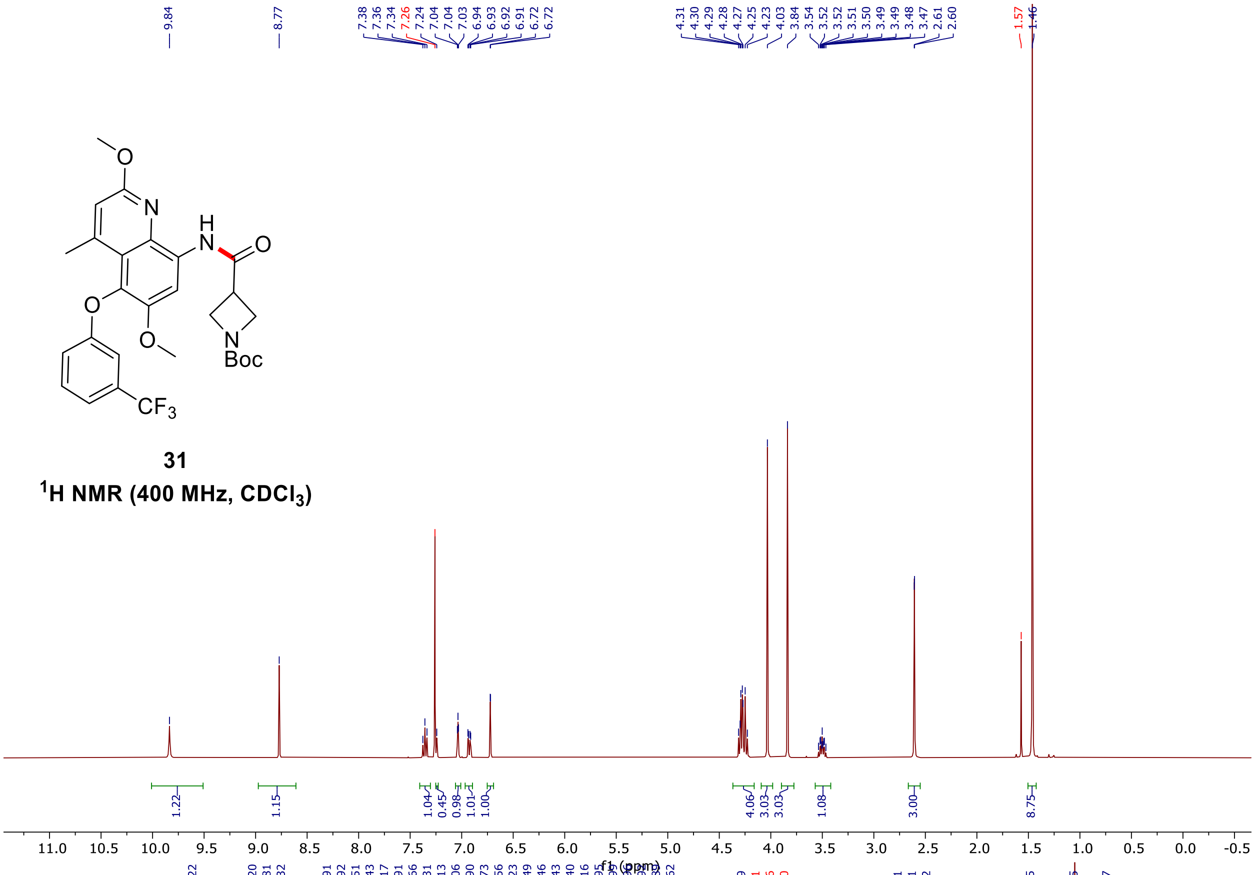

31

^{1}H NMR (400 MHz, $CDCl_3$)

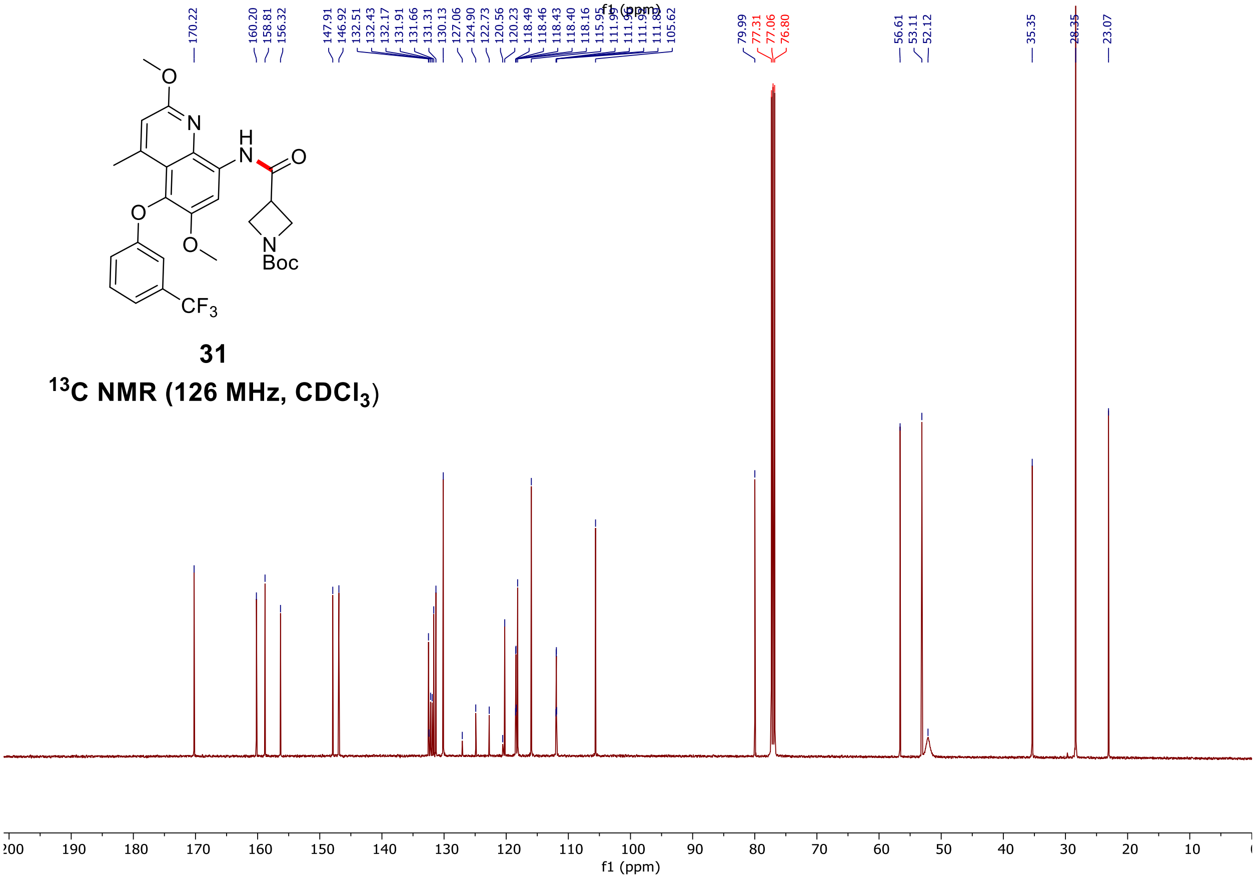

31

^{13}C NMR (126 MHz, $CDCl_3$)

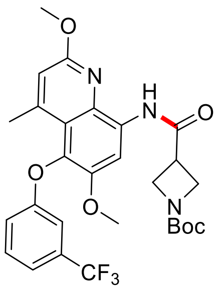

31

^{19}F NMR (376 MHz, $CDCl_3$)

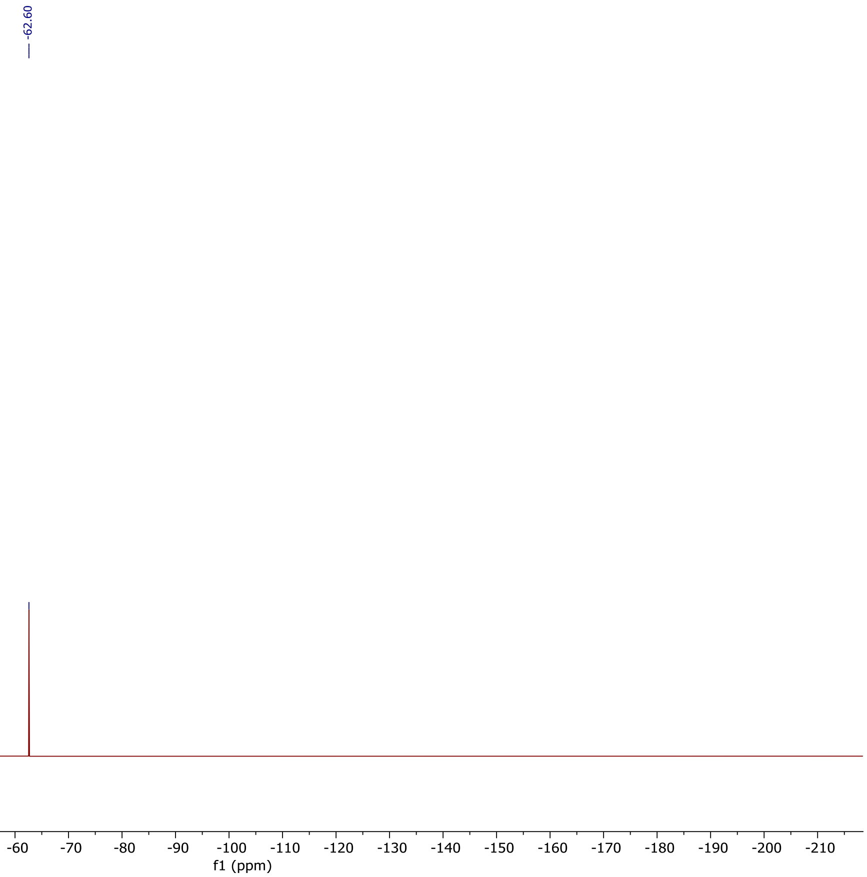

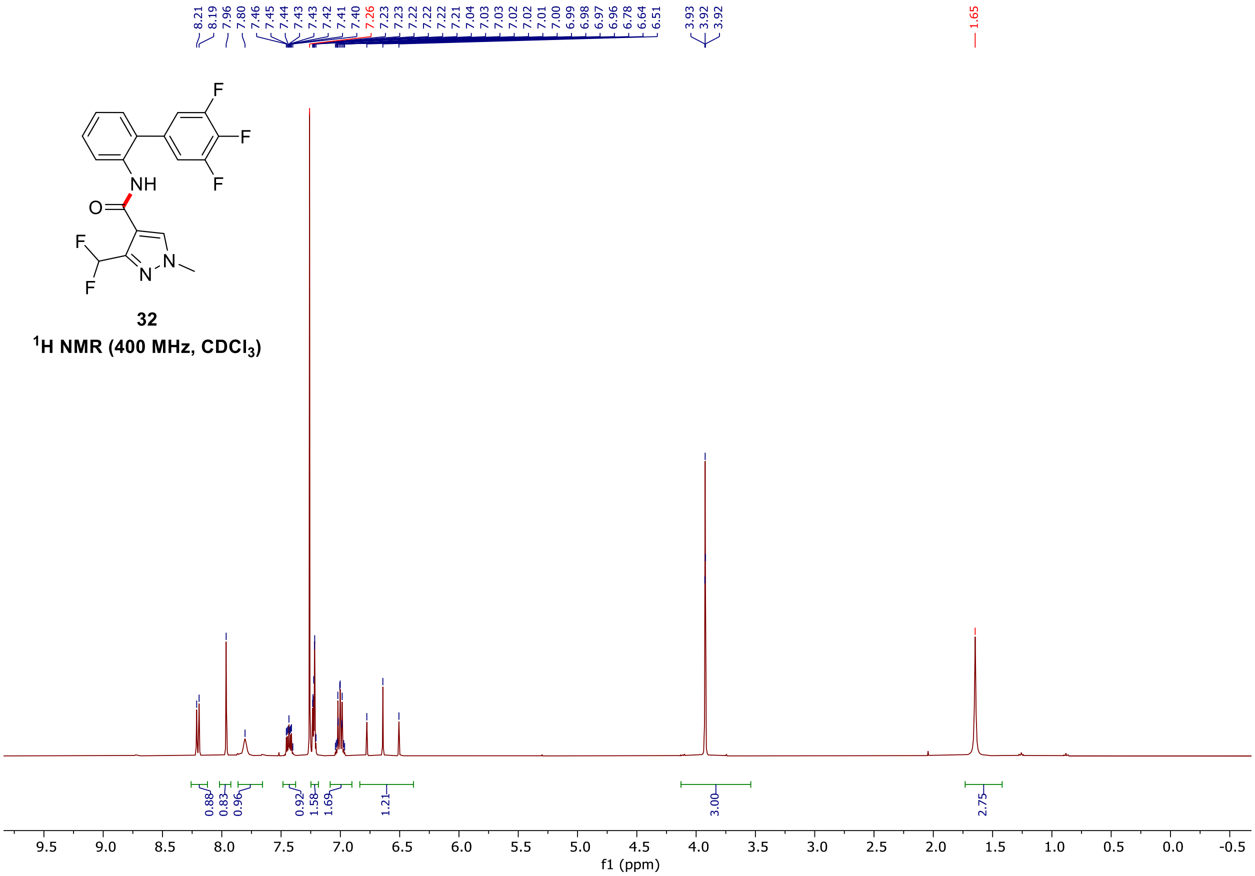
8.21 8.19 7.96 7.80 7.46 7.45 7.44 7.43 7.43 7.42 7.41 7.40 7.26 7.23 7.23 7.22 7.22 7.22 7.21 7.04 7.03 7.03 7.02 7.02 7.01 7.00 6.99 6.98 6.97 6.96 6.78 6.64 6.51
3.93 3.92 3.92
1.65
32
^{1}H NMR (400 MHz, $CDCl_3$)
0.88 0.83 0.96 0.92 1.58 1.69 1.21 3.00 2.75
f1 (ppm)

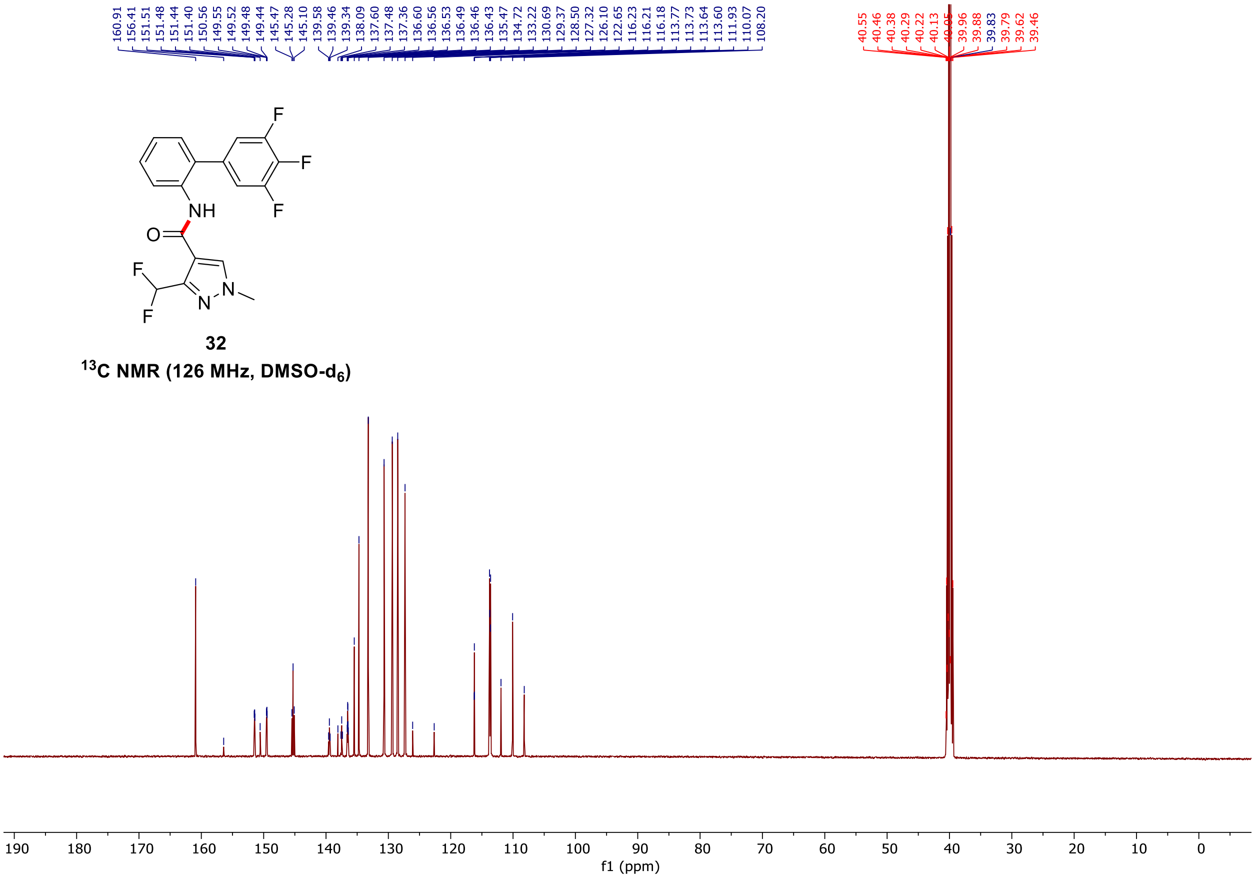
160.91 156.41 151.51 151.48 151.44 151.40 150.56 149.55 149.52 149.48 149.44 145.47 145.28 145.10 139.58 139.46 139.34 138.09 137.60 137.48 137.36 136.60 136.56 136.53 136.49 136.46 136.43 135.47 134.72 133.22 130.69 129.37 128.50 127.32 126.10 122.65 116.23 116.21 116.18 113.77 113.73 113.64 113.60 111.93 110.07 108.20
40.55 40.46 40.38 40.29 40.22 40.13 40.05 39.96 39.88 39.83 39.79 39.62 39.46
32
^{13}C NMR (126 MHz, DMSO-d_6)
f1 (ppm)

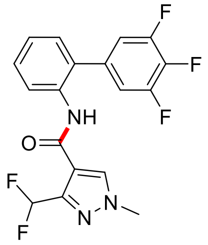

32

^{19}F NMR (376 MHz, $CDCl_3$)

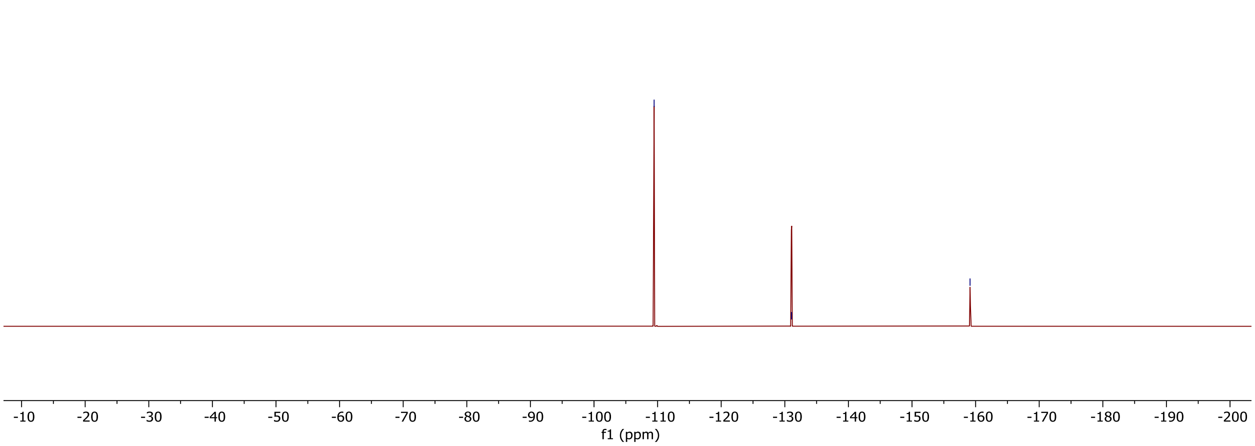

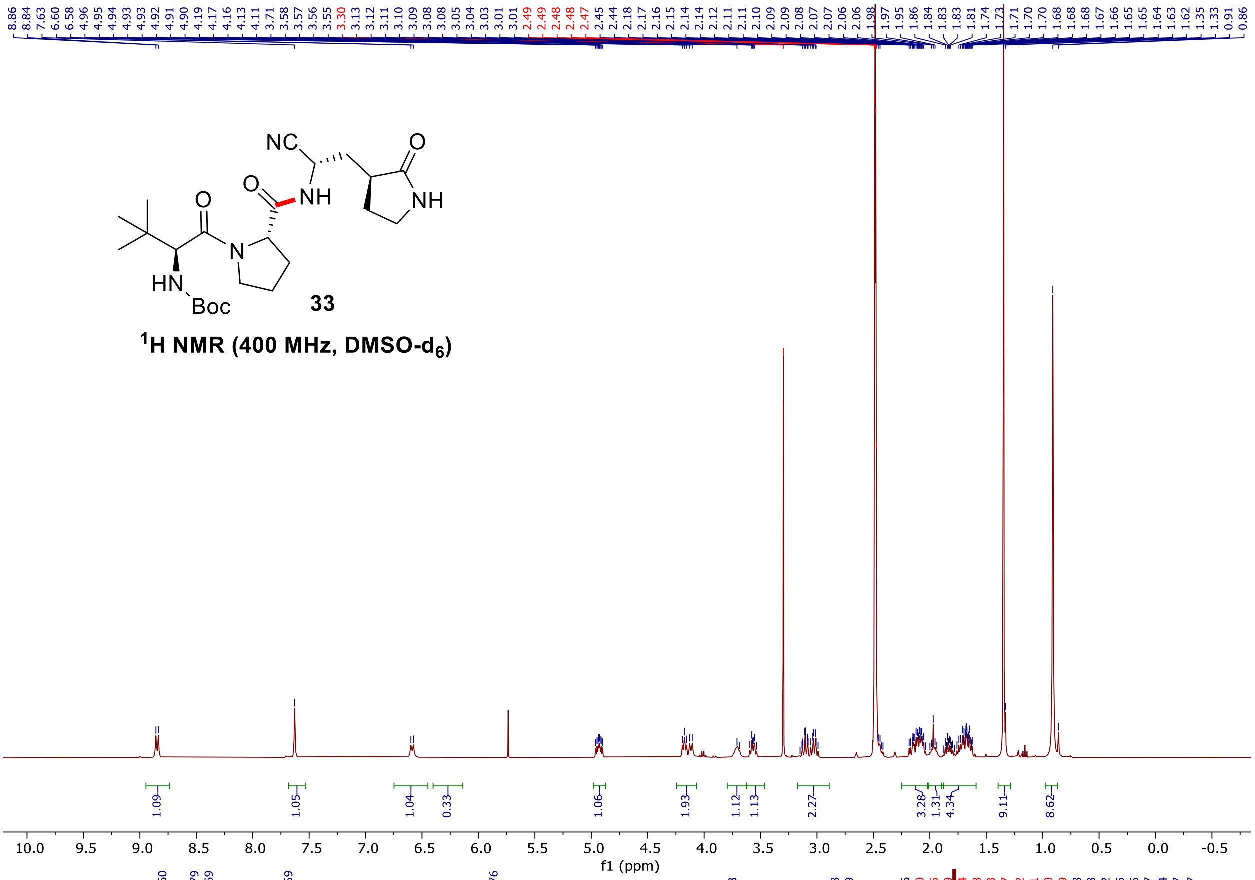

33

^{1}H NMR (400 MHz, DMSO-d_6)

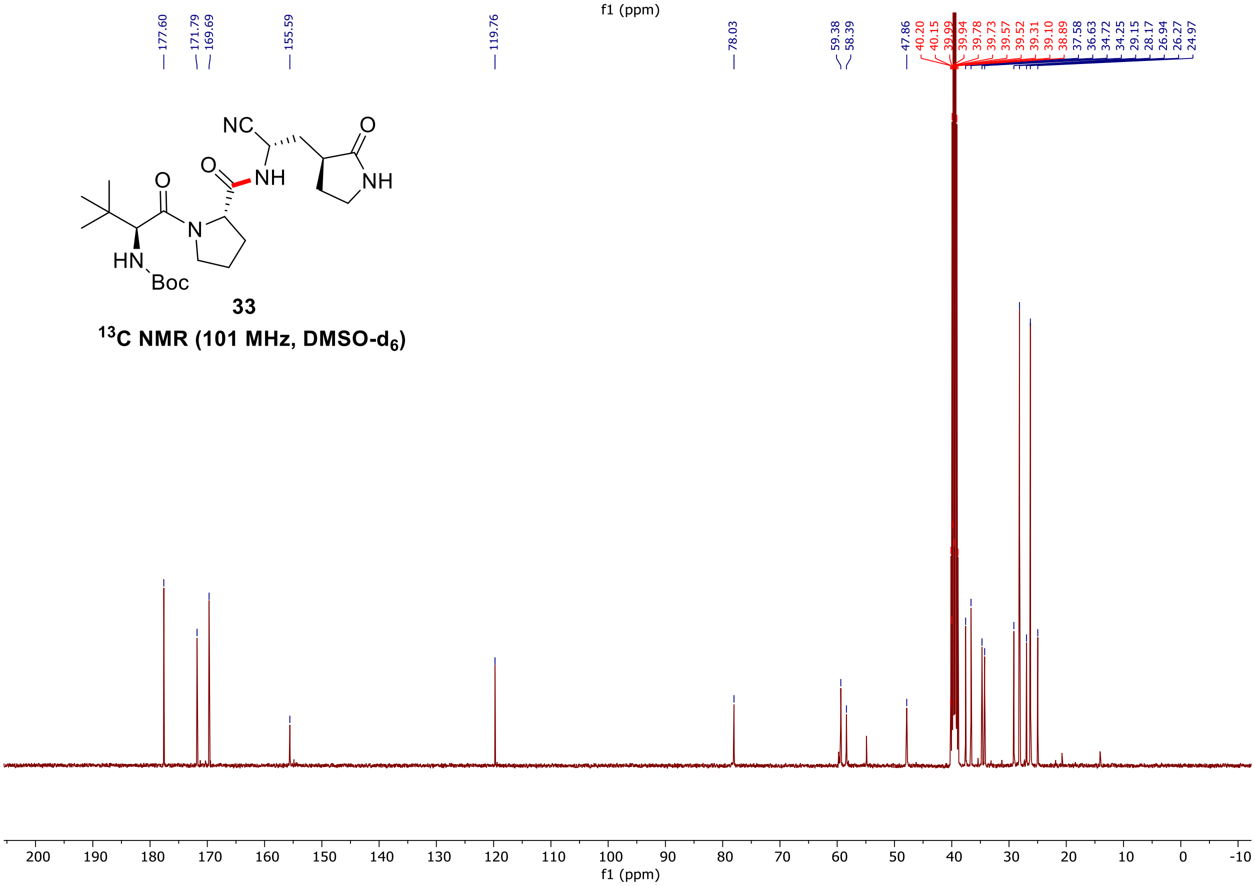

33

^{13}C NMR (101 MHz, DMSO-d_6)

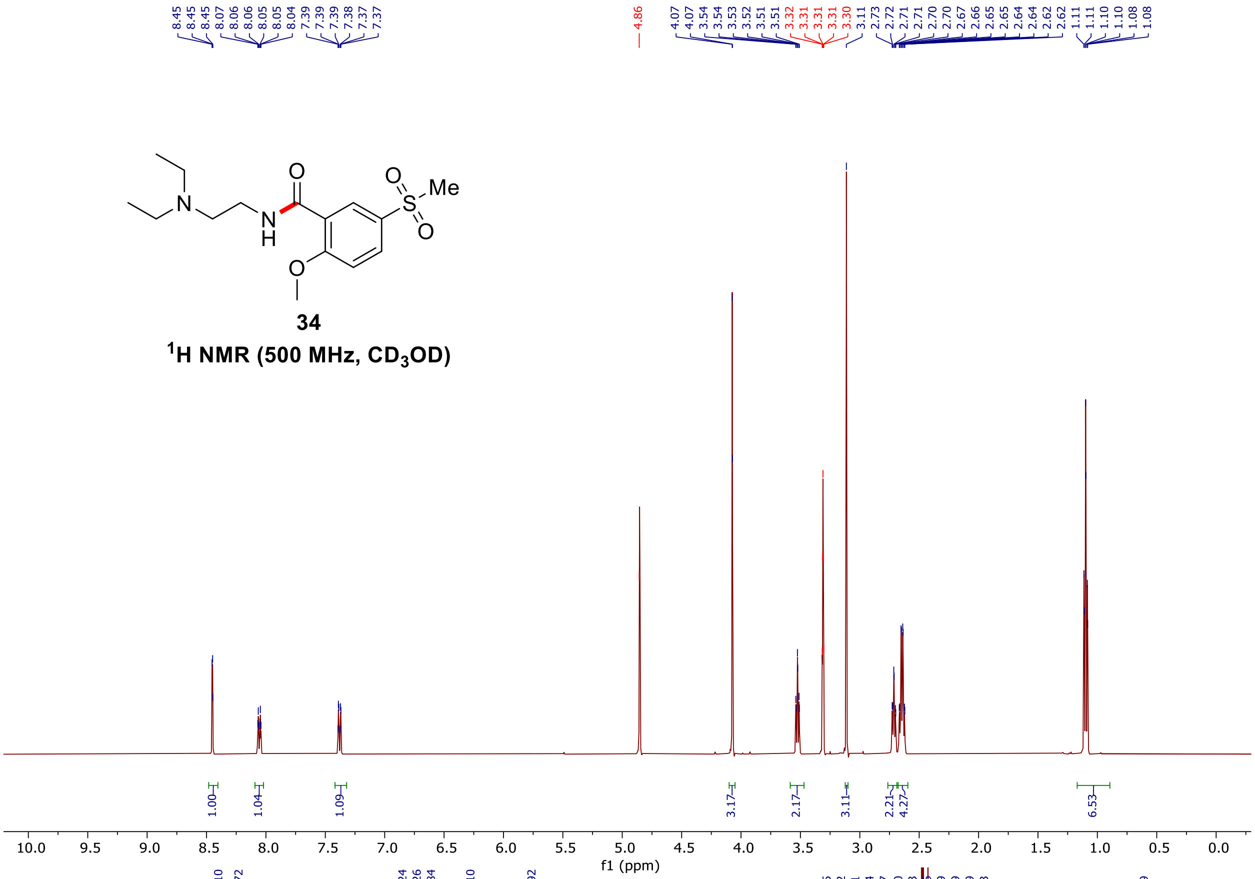

34

^{1}H NMR (500 MHz, CD_3OD)

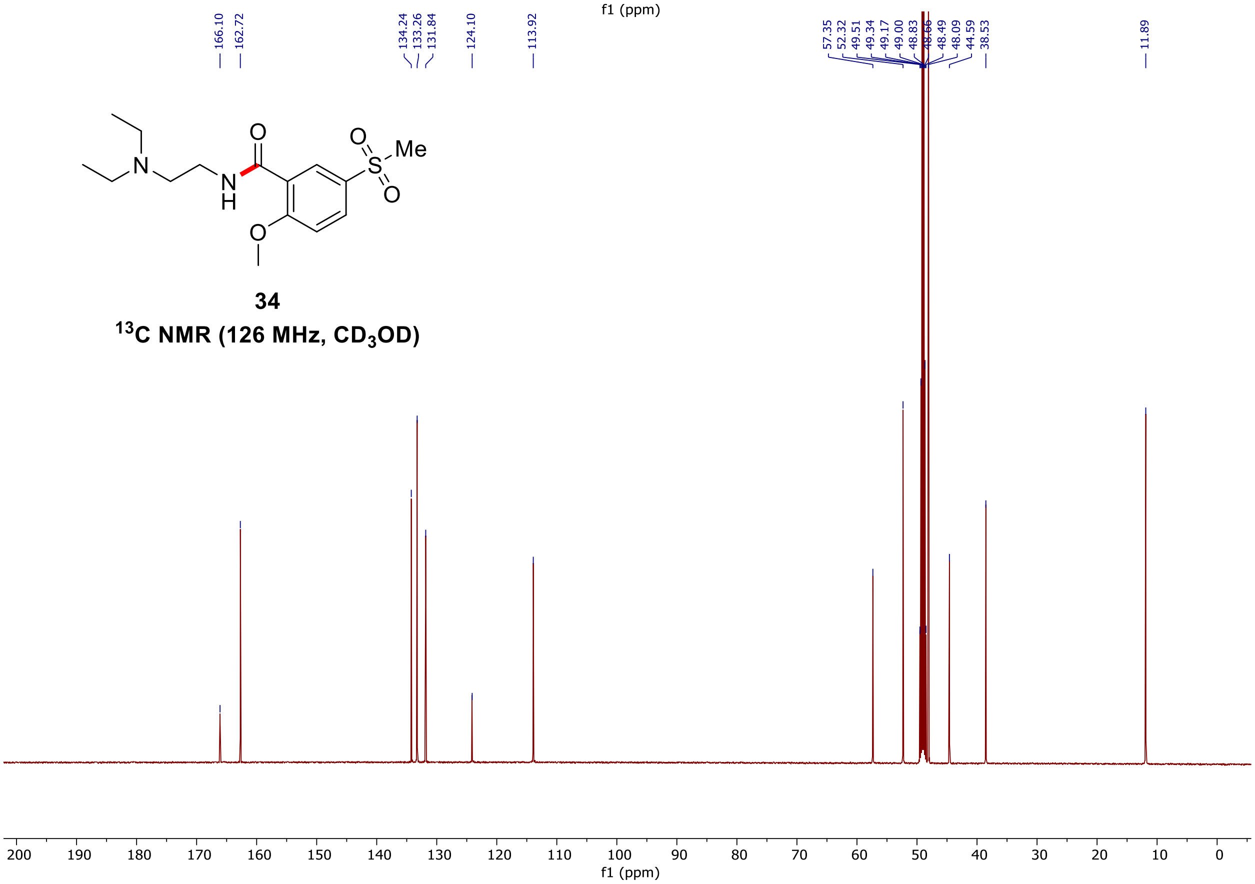

34

^{13}C NMR (126 MHz, CD_3OD)

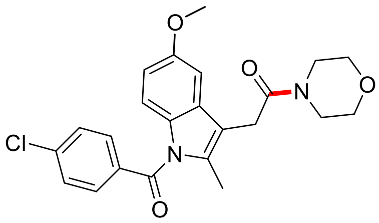

35

^{1}H NMR (400 MHz, $CDCl_3$)

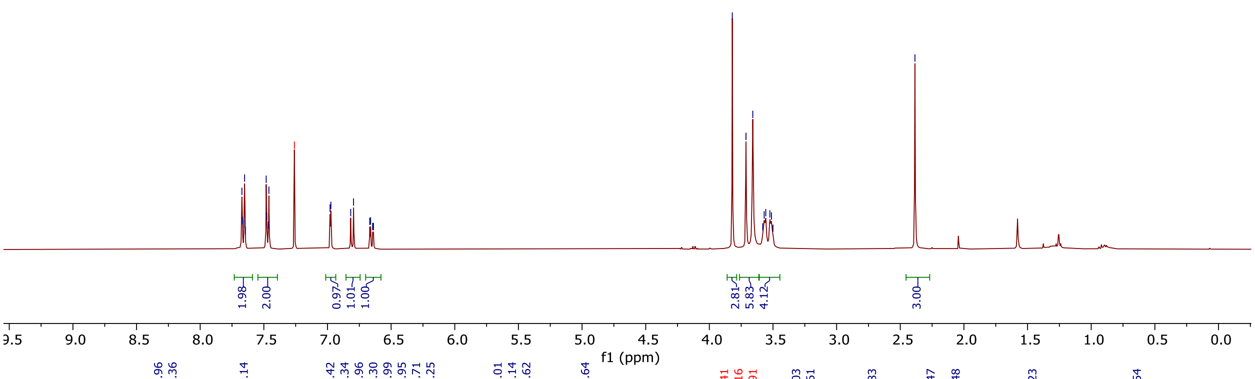

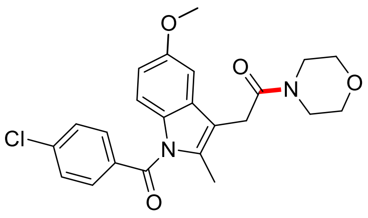

35

^{13}C NMR (126 MHz, $CDCl_3$)

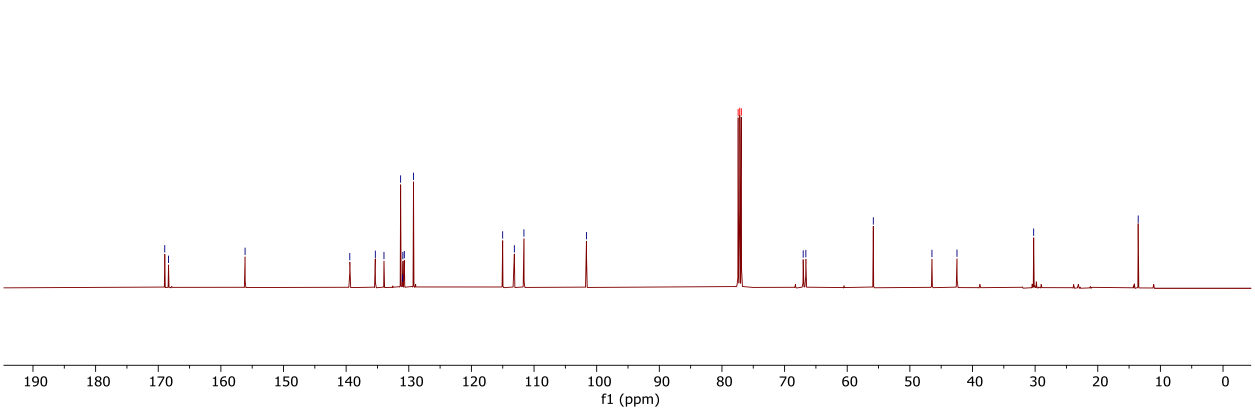

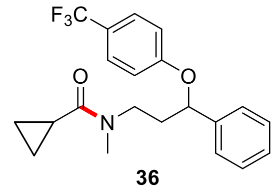

36

^{1}H NMR (500 MHz, $CDCl_3$)

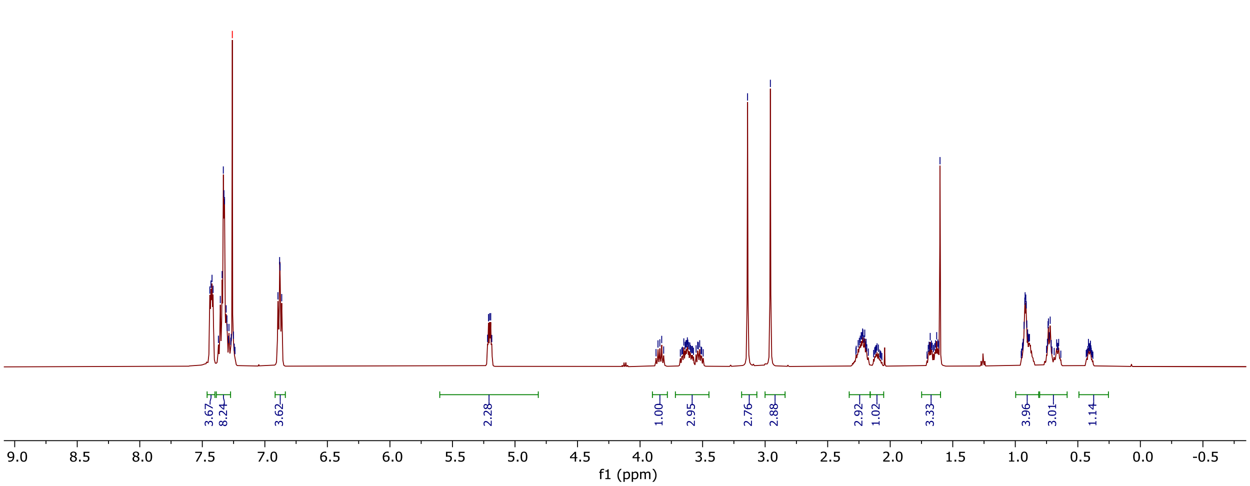

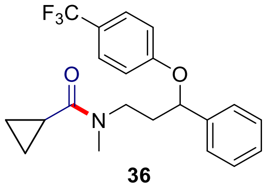

36

^{13}C NMR (126 MHz, $CDCl_3$)

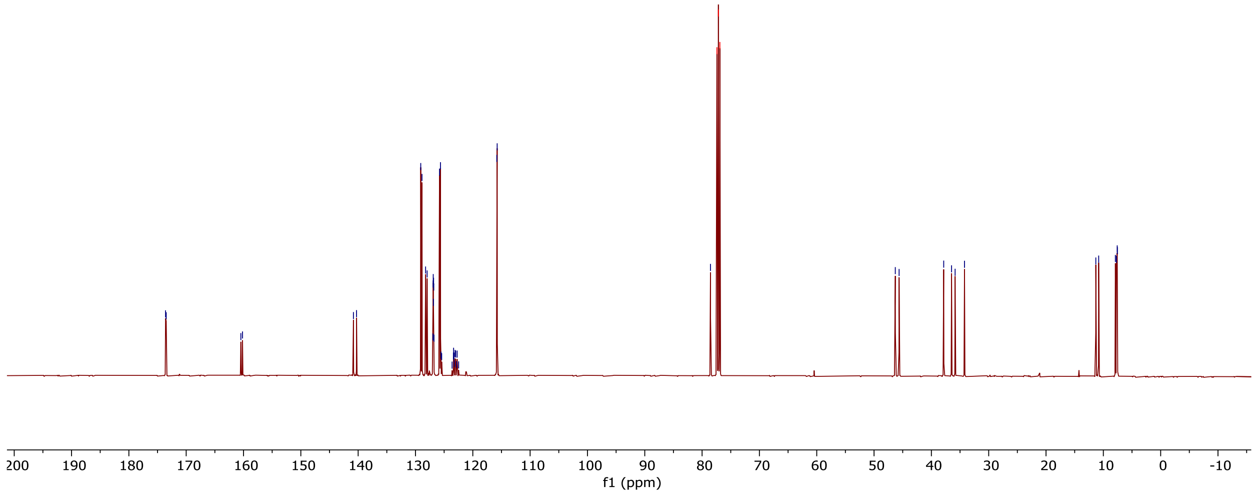

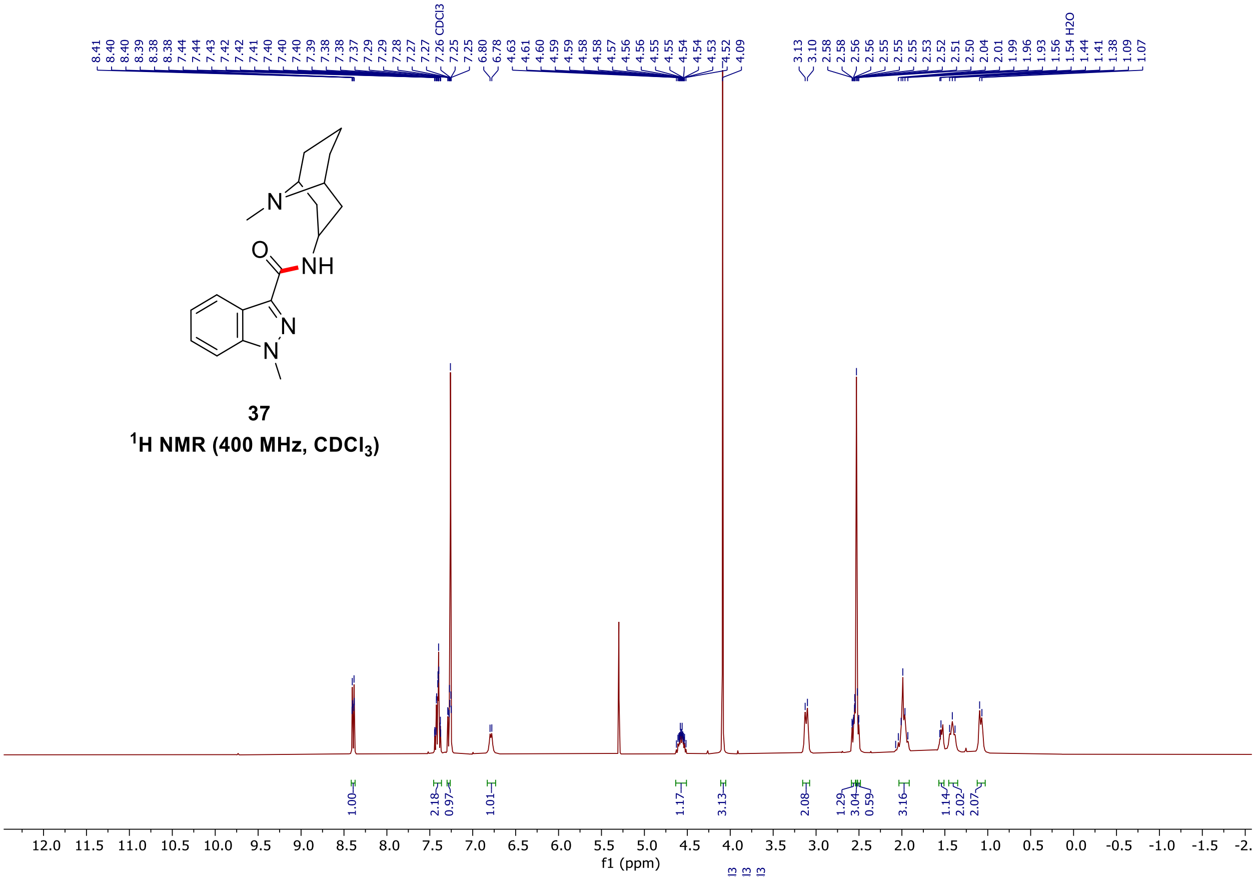

37

^{1}H NMR (400 MHz, $CDCl_3$)

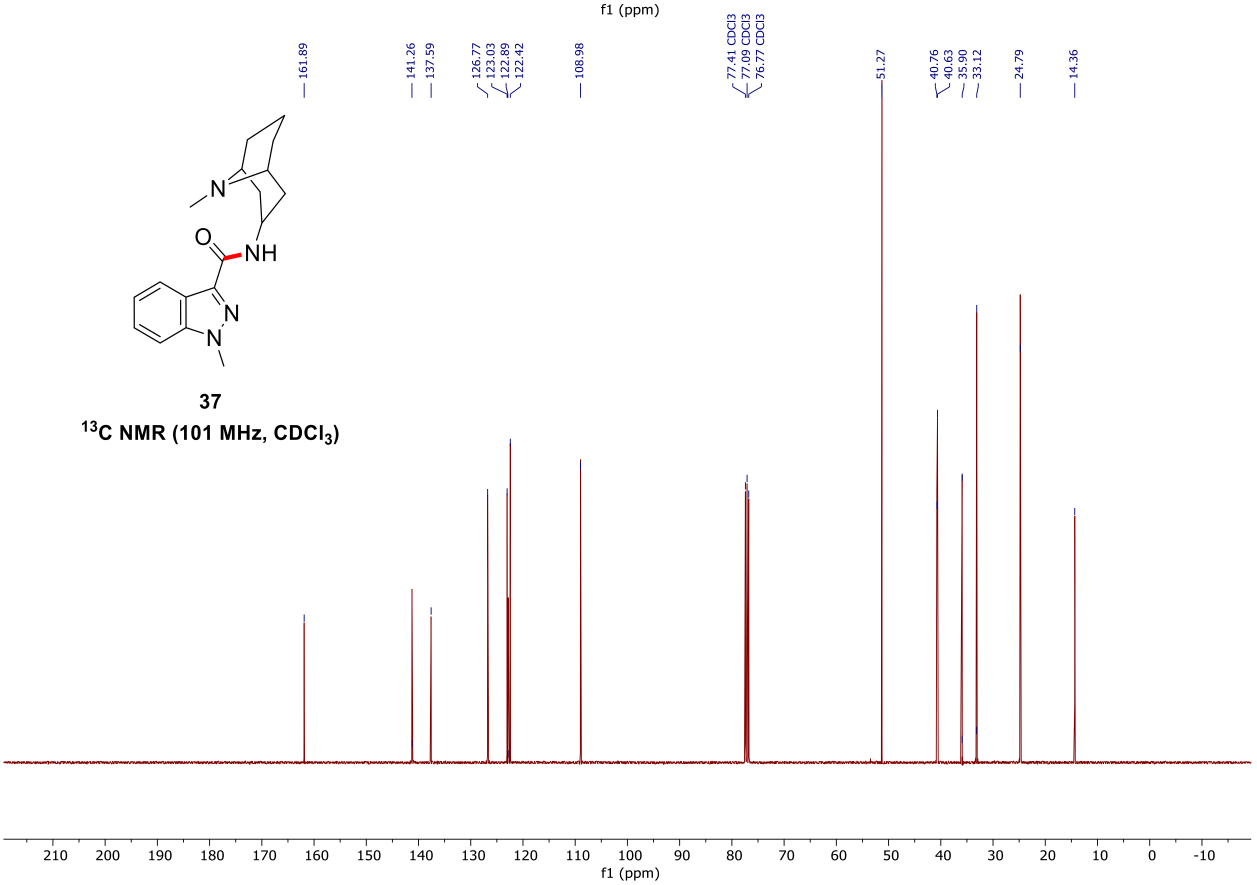

37

^{13}C NMR (101 MHz, $CDCl_3$)

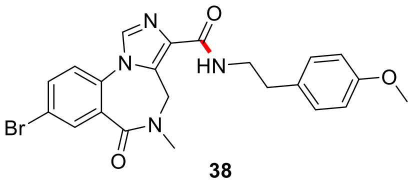

38

^{1}H NMR (400 MHz, DMSO-d_6)

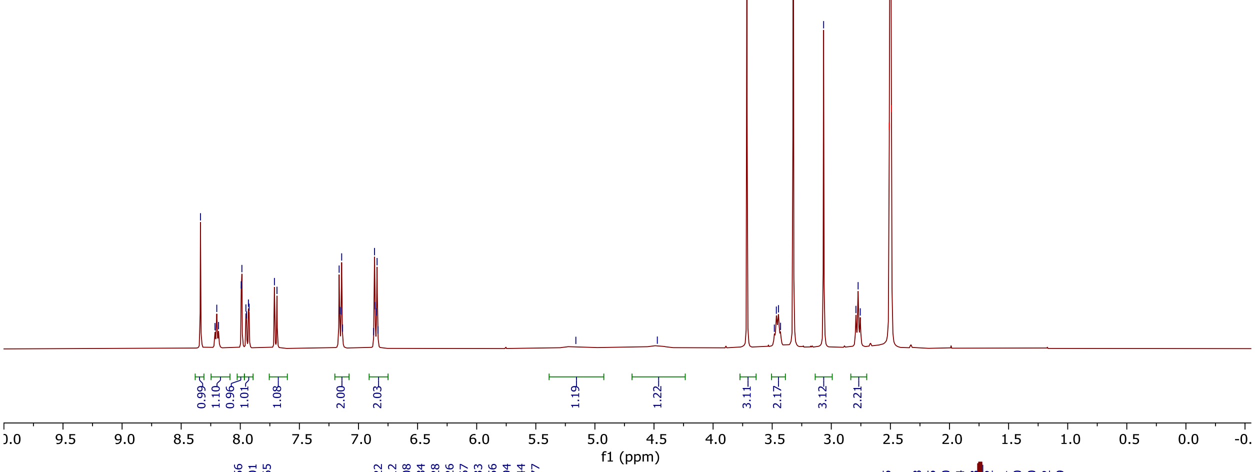

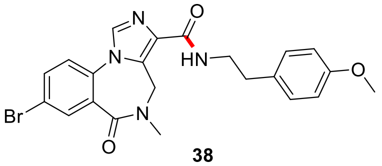

38

^{13}C NMR (101 MHz, DMSO-d_6)

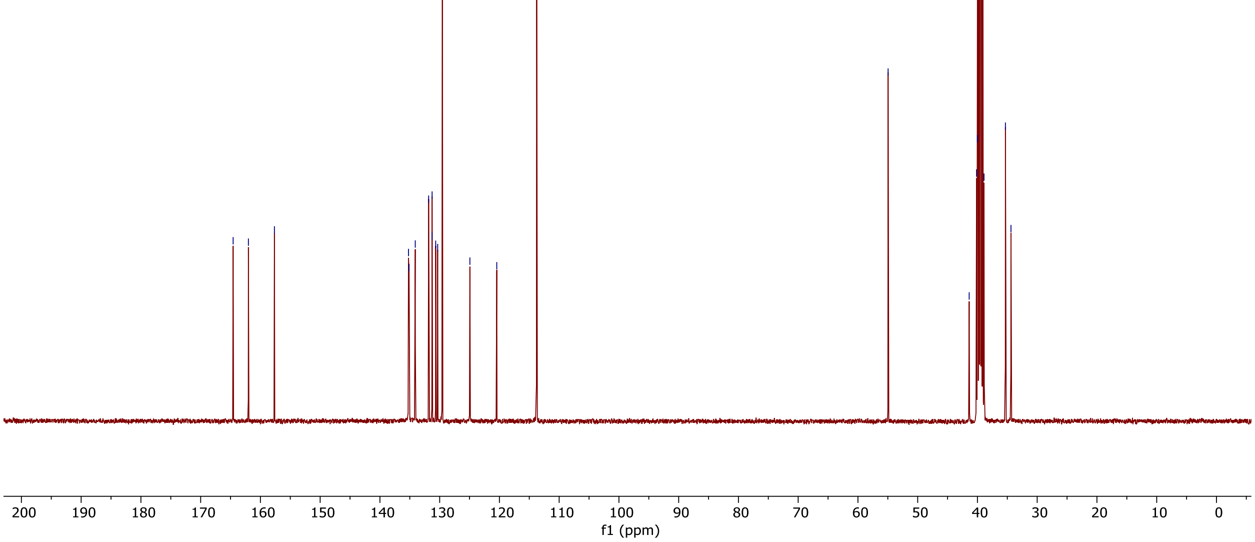

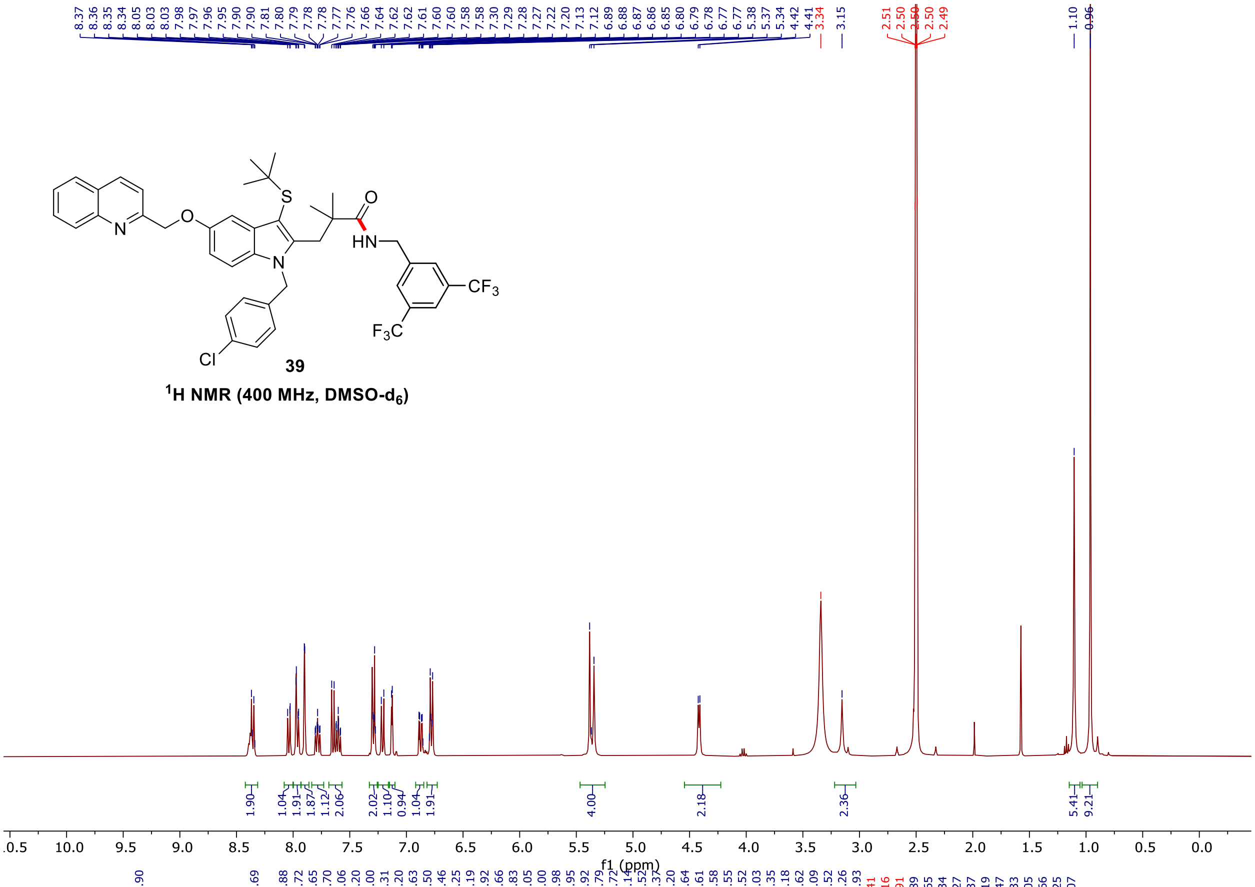
8.37 8.36 8.35 8.34 8.05 8.03 8.03 7.98 7.97 7.96 7.95 7.90 7.90 7.81 7.80 7.79 7.78 7.78 7.77 7.76 7.66 7.64 7.62 7.62 7.61 7.60 7.60 7.58 7.58 7.30 7.29 7.28 7.27 7.22 7.20 7.13 7.12 6.89 6.88 6.87 6.86 6.85 6.80 6.79 6.78 6.77 6.77 5.38 5.37 5.34 4.42 4.41 3.34 3.15 2.51 2.50 2.50 2.50 2.49 1.10 0.96
S
O
O
N
HN
N
CF_3
F_3C
Cl
39
^{1}H NMR (400 MHz, DMSO-d6)
1.90 1.04 1.91 1.87 1.12 2.06 2.02 1.10 0.94 1.04 1.91 4.00 2.18 2.36 5.41 9.21
10.5 10.0 9.5 9.0 8.5 8.0 7.5 7.0 6.5 6.0 5.5 5.0 4.5 4.0 3.5 3.0 2.5 2.0 1.5 1.0 0.5 0.0
f1 (ppm)

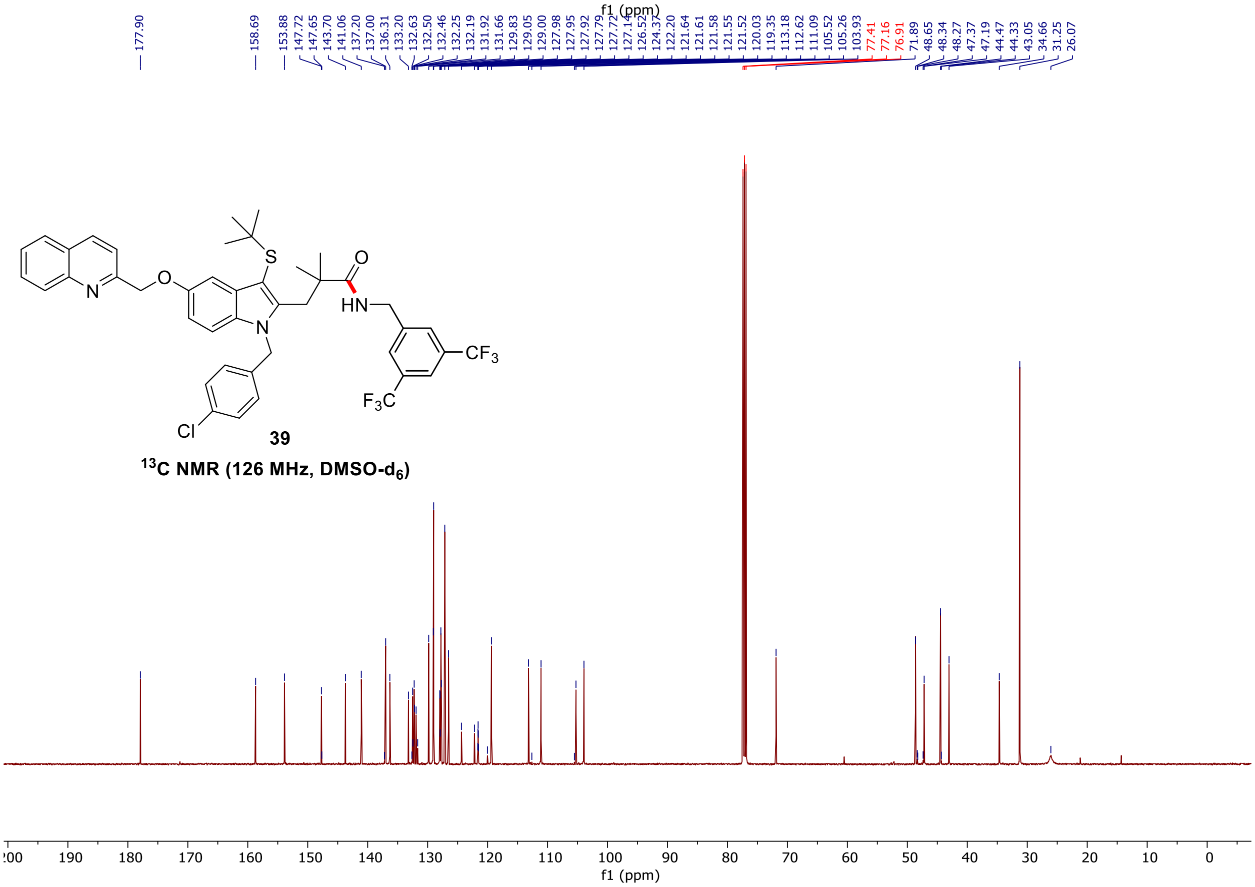
177.90 158.69 153.88 147.72 147.65 143.70 141.06 137.20 137.00 136.31 133.20 132.63 132.50 132.46 132.25 132.19 131.92 131.66 129.83 129.05 129.00 127.98 127.95 127.92 127.79 127.72 127.14 126.52 124.37 122.20 121.64 121.61 121.58 121.55 121.52 120.03 119.35 113.18 112.62 111.09 105.52 105.26 103.93 77.41 77.16 76.91 71.89 48.65 48.34 48.27 47.37 47.19 44.47 44.33 43.05 34.66 31.25 26.07
S
O
O
N
HN
N
CF_3
F_3C
Cl
39
^{13}C NMR (126 MHz, DMSO-d6)
200 190 180 170 160 150 140 130 120 110 100 90 80 70 60 50 40 30 20 10 0
f1 (ppm)

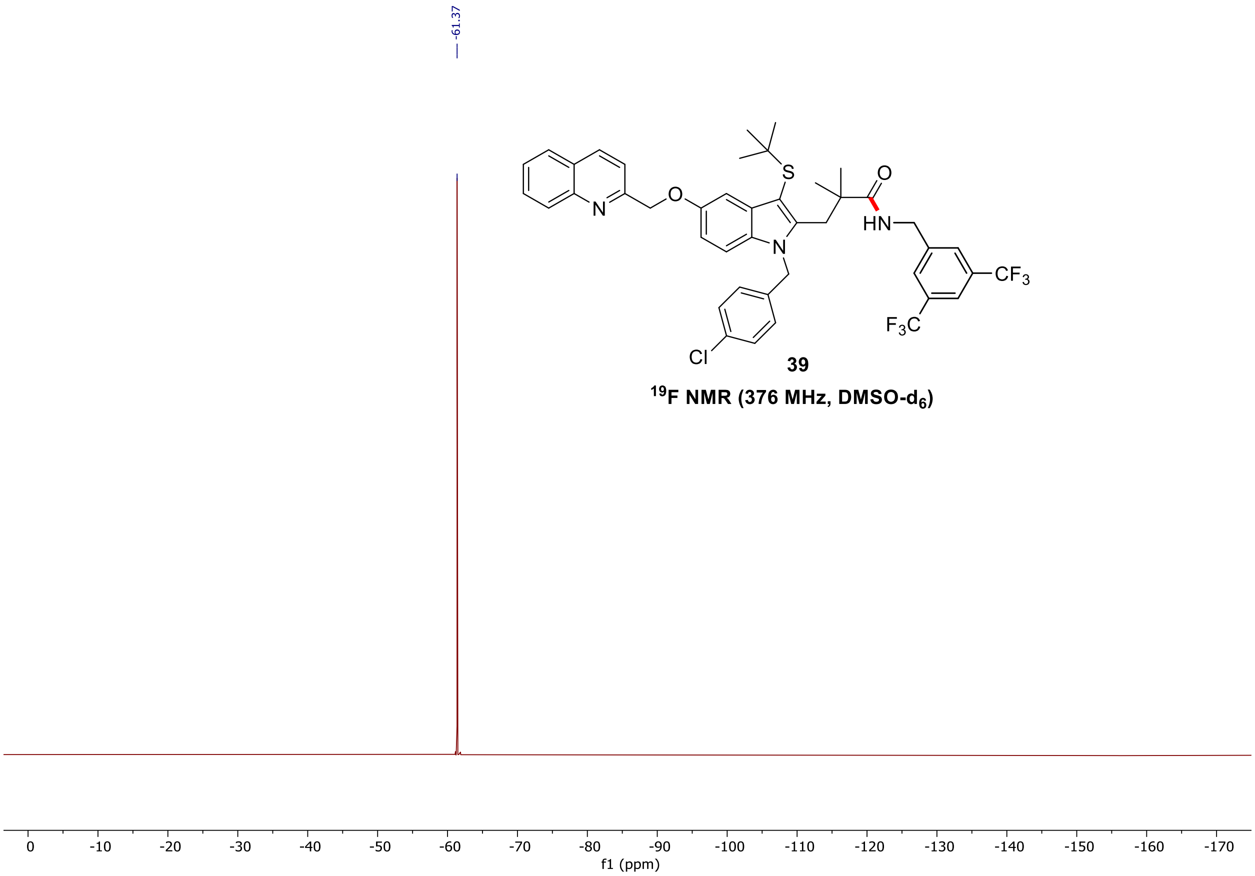

39

^{19}F NMR (376 MHz, DMSO-d$_6$)

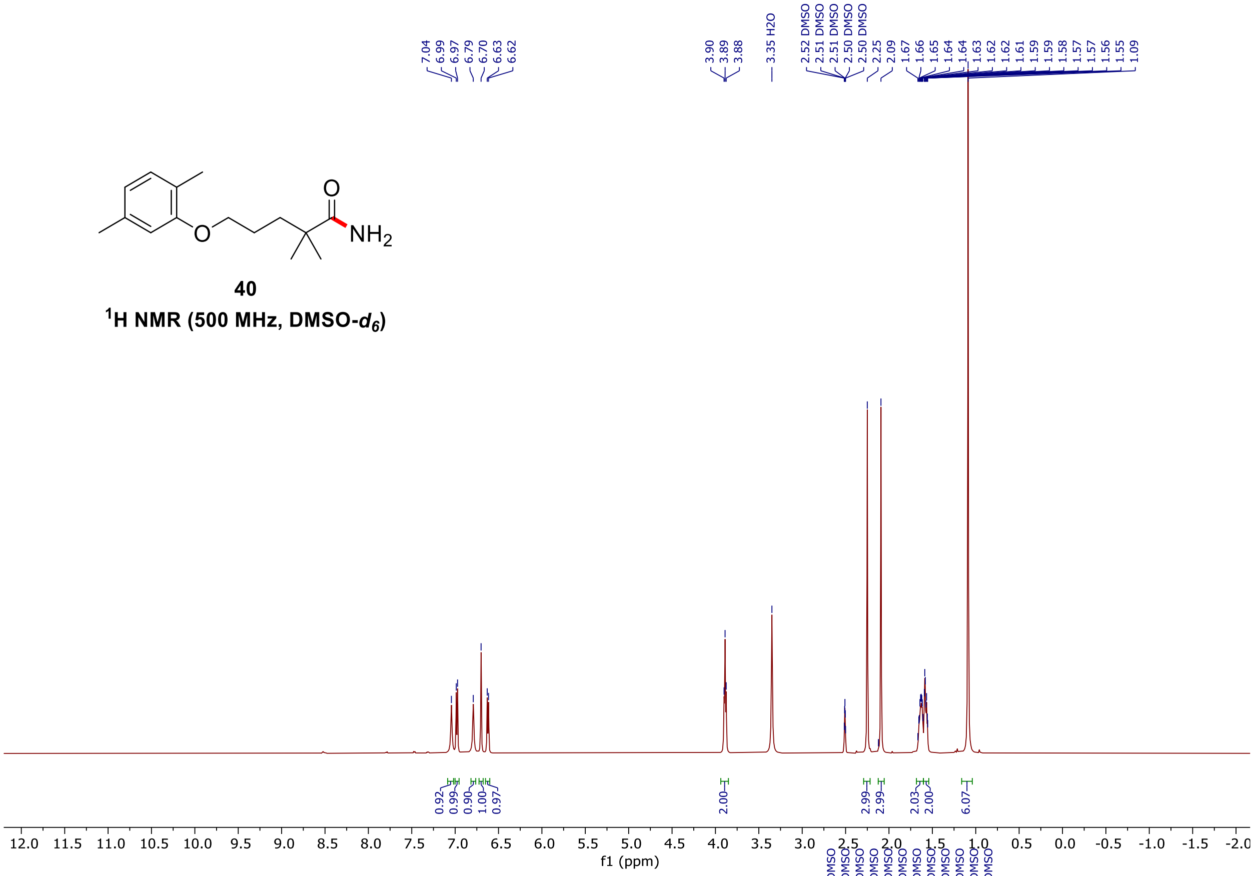

40

^{1}H NMR (500 MHz, DMSO-*d$_6$*)

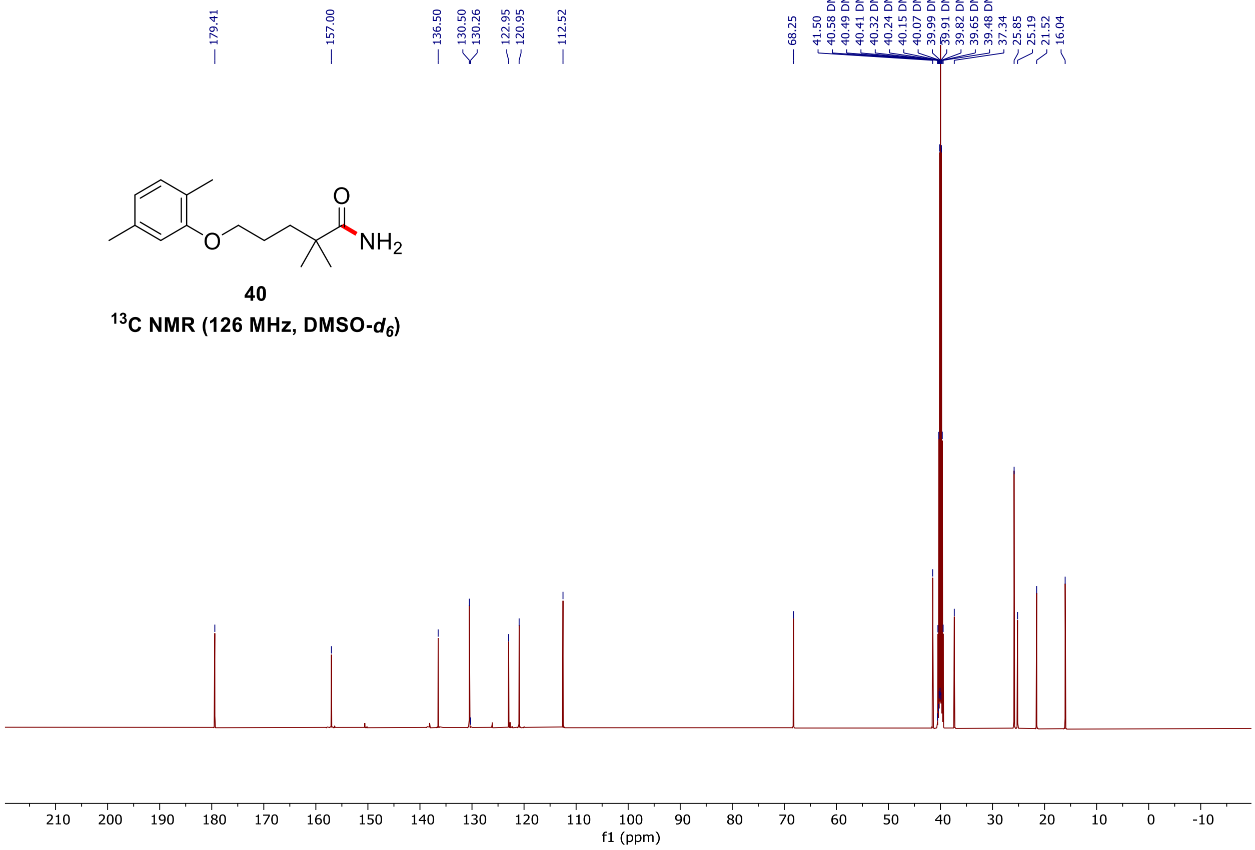

40

^{13}C NMR (126 MHz, DMSO-*d$_6$*)

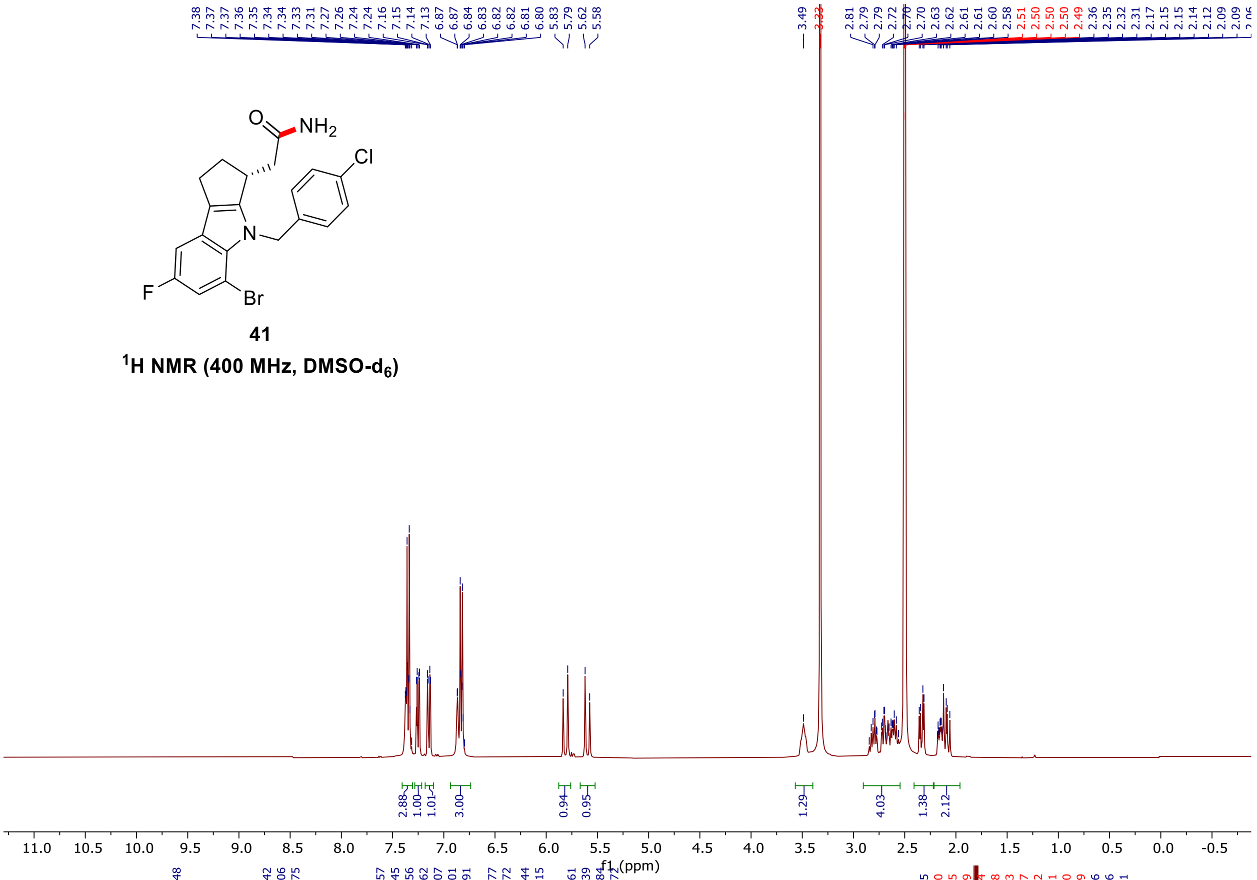

41

^{1}H NMR (400 MHz, DMSO-d$_6$)

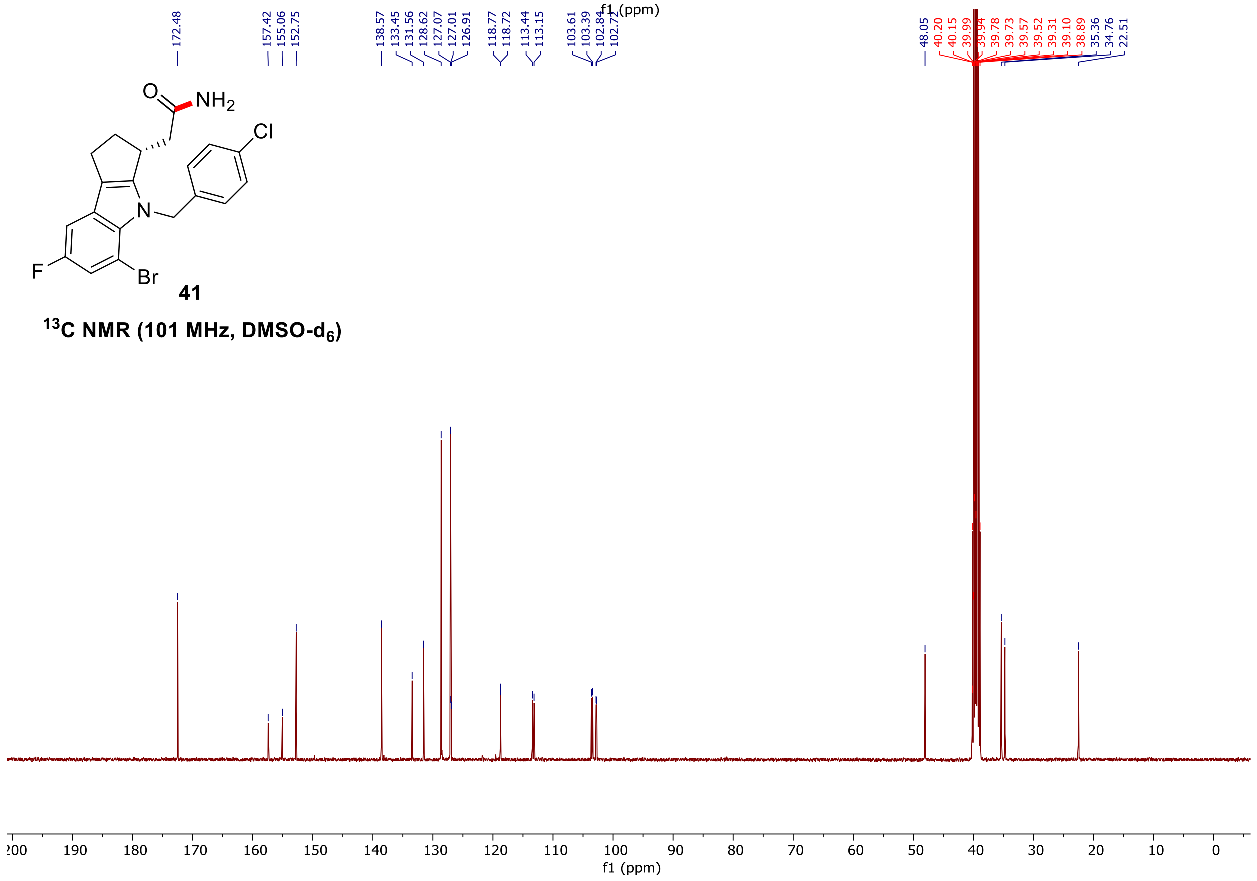

41

^{13}C NMR (101 MHz, DMSO-d$_6$)

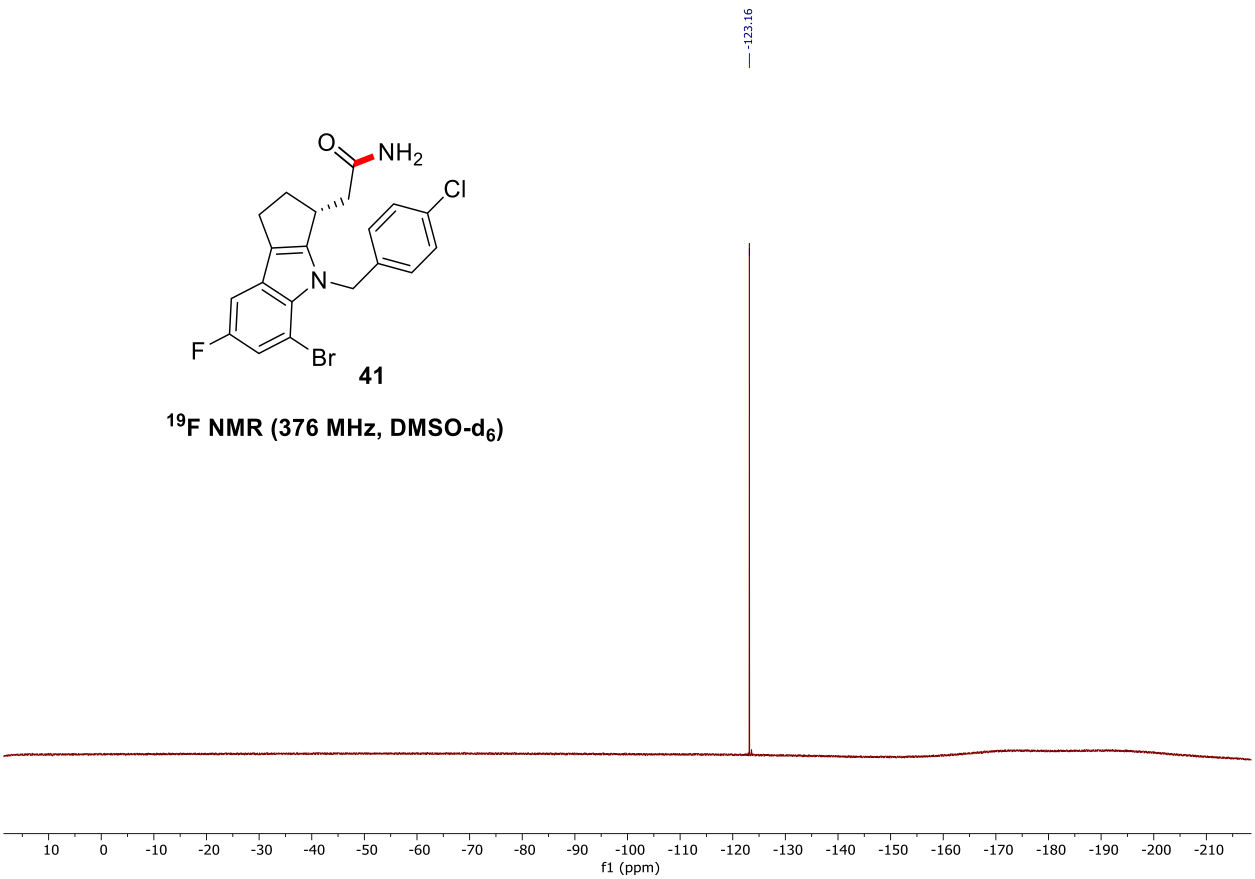

41

^{19}F NMR (376 MHz, DMSO-d_6)

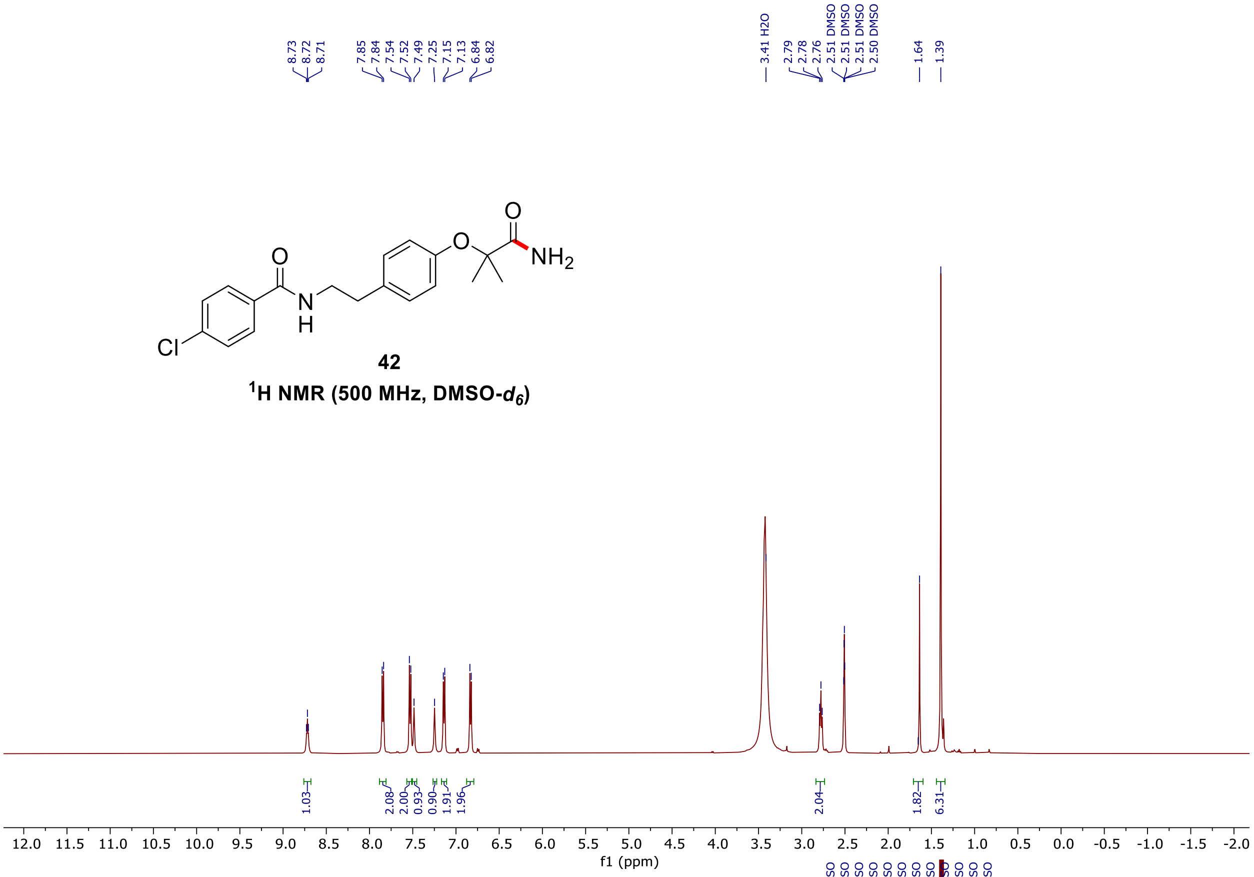
8.73
8.72
8.71
7.85
7.84
7.54
7.52
7.49
7.25
7.15
7.13
6.84
6.82
3.41 H2O
2.79
2.78
2.76
2.51 DMSO
2.51 DMSO
2.51 DMSO
2.50 DMSO
1.64
1.39
O
O
NH2
O
N
H
Cl
42
1H NMR (500 MHz, DMSO-d6)
1.03
2.08
2.00
0.93
0.90
1.91
1.96
2.04
1.82
6.31
12.0 11.5 11.0 10.5 10.0 9.5 9.0 8.5 8.0 7.5 7.0 6.5 6.0 5.5 5.0 4.5 4.0 3.5 3.0 2.5 2.0 1.5 1.0 0.5 0.0 -0.5 -1.0 -1.5 -2.0
f1 (ppm)

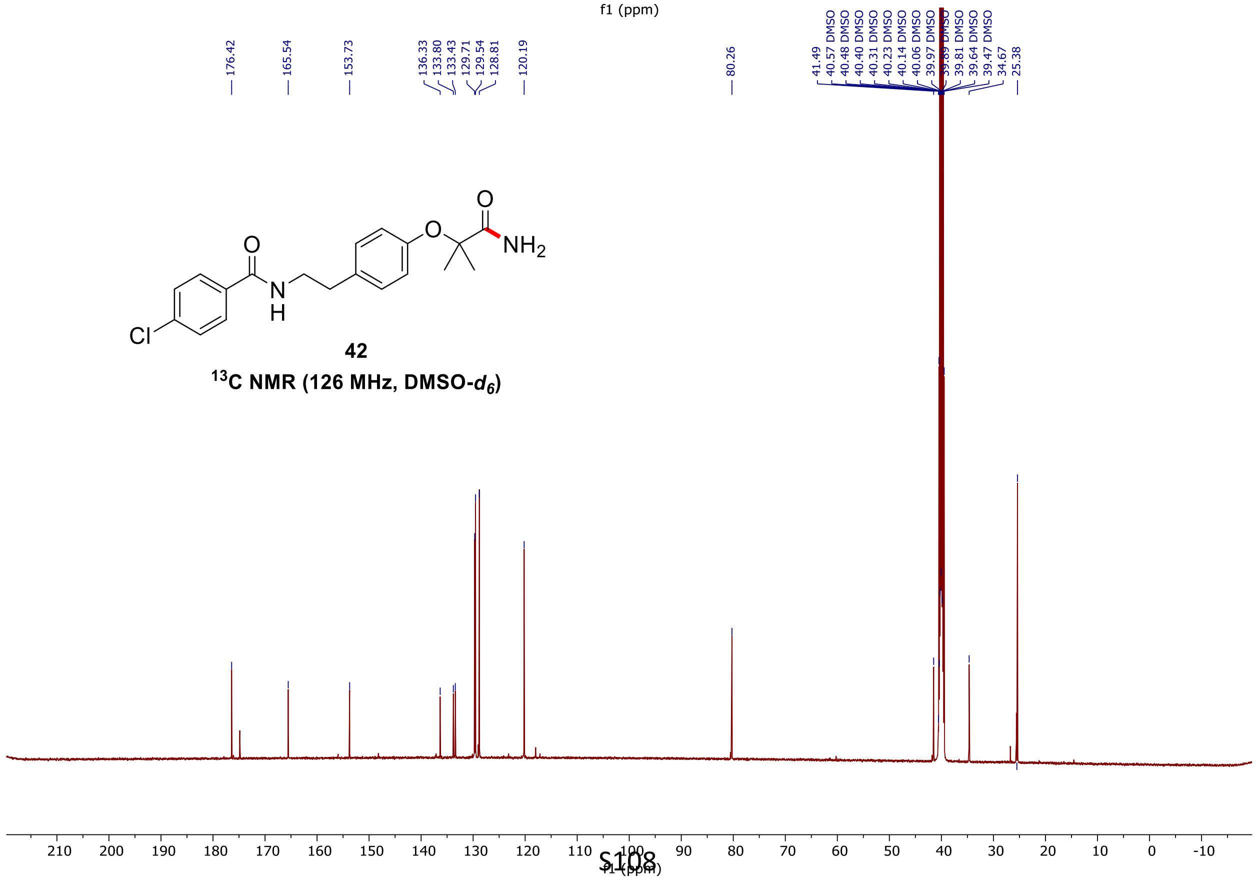
176.42
165.54
153.73
136.33
133.80
133.43
129.71
129.54
128.81
120.19
80.26
41.49
40.57 DMSO
40.48 DMSO
40.40 DMSO
40.31 DMSO
40.23 DMSO
40.14 DMSO
40.06 DMSO
39.97 DMSO
39.89 DMSO
39.81 DMSO
39.64 DMSO
39.47 DMSO
34.67
25.38
O
O
NH2
O
N
H
Cl
42
13C NMR (126 MHz, DMSO-d6)
210 200 190 180 170 160 150 140 130 120 110 100 90 80 70 60 50 40 30 20 10 0 -10
f1 (ppm)

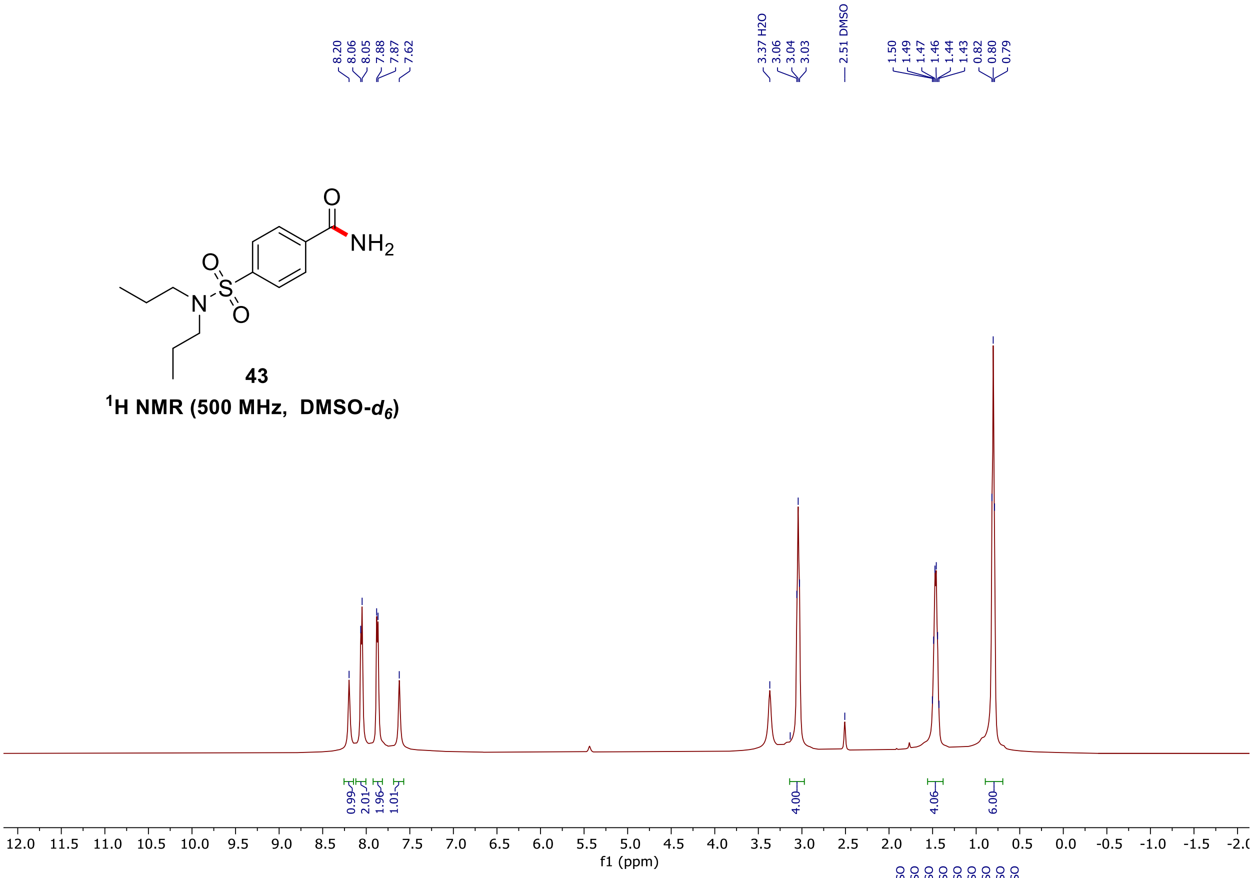

8.20
8.06
8.05
7.88
7.87
7.62
3.37 H2O
3.06
3.04
3.03
2.51 DMSO
1.50
1.49
1.47
1.46
1.44
1.43
0.82
0.80
0.79
O
NH2
O
S
N
O
43
1H NMR (500 MHz, DMSO-d6)
0.99
2.01
1.96
1.01
4.00
4.06
6.00
12.0 11.5 11.0 10.5 10.0 9.5 9.0 8.5 8.0 7.5 7.0 6.5 6.0 5.5 5.0 4.5 4.0 3.5 3.0 2.5 2.0 1.5 1.0 0.5 0.0 -0.5 -1.0 -1.5
f1 (ppm)

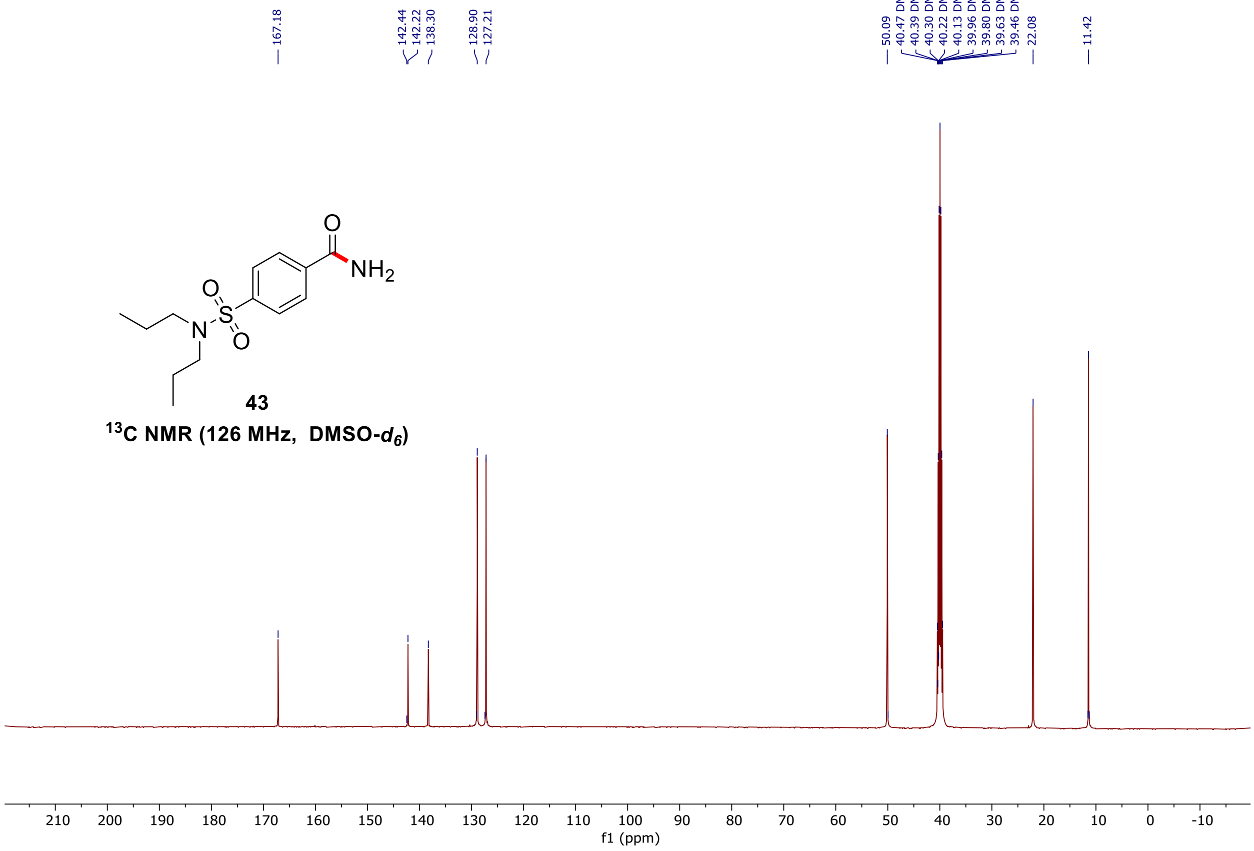

167.18
142.44
142.22
138.30
128.90
127.21
50.09
40.47 DMSO
40.39 DMSO
40.30 DMSO
40.22 DMSO
40.13 DMSO
39.96 DMSO
39.80 DMSO
39.63 DMSO
39.46 DMSO
22.08
11.42
O
NH2
O
S
N
O
43
13C NMR (126 MHz, DMSO-d6)
210 200 190 180 170 160 150 140 130 120 110 100 90 80 70 60 50 40 30 20 10 0 -10
f1 (ppm)

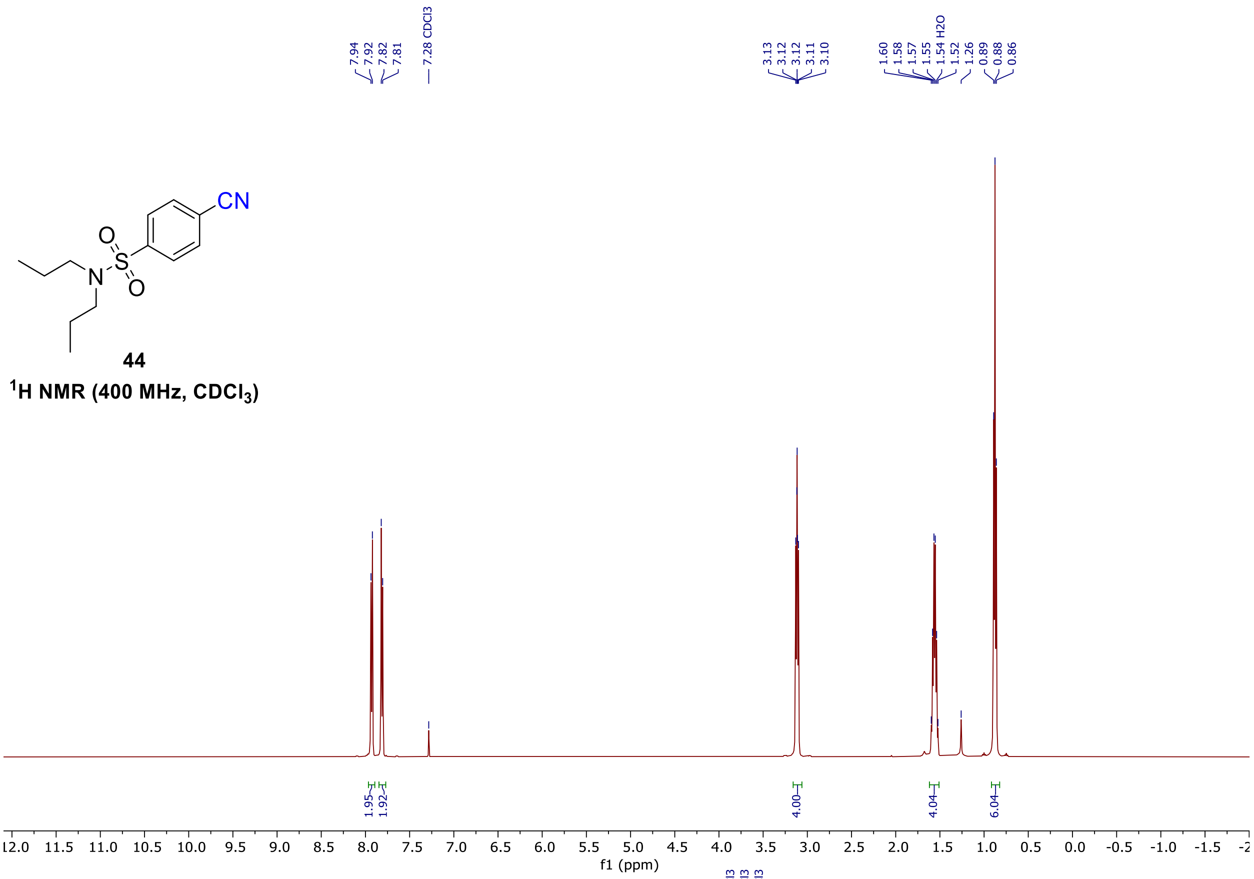

44

^{1}H NMR (400 MHz, $CDCl_3$)

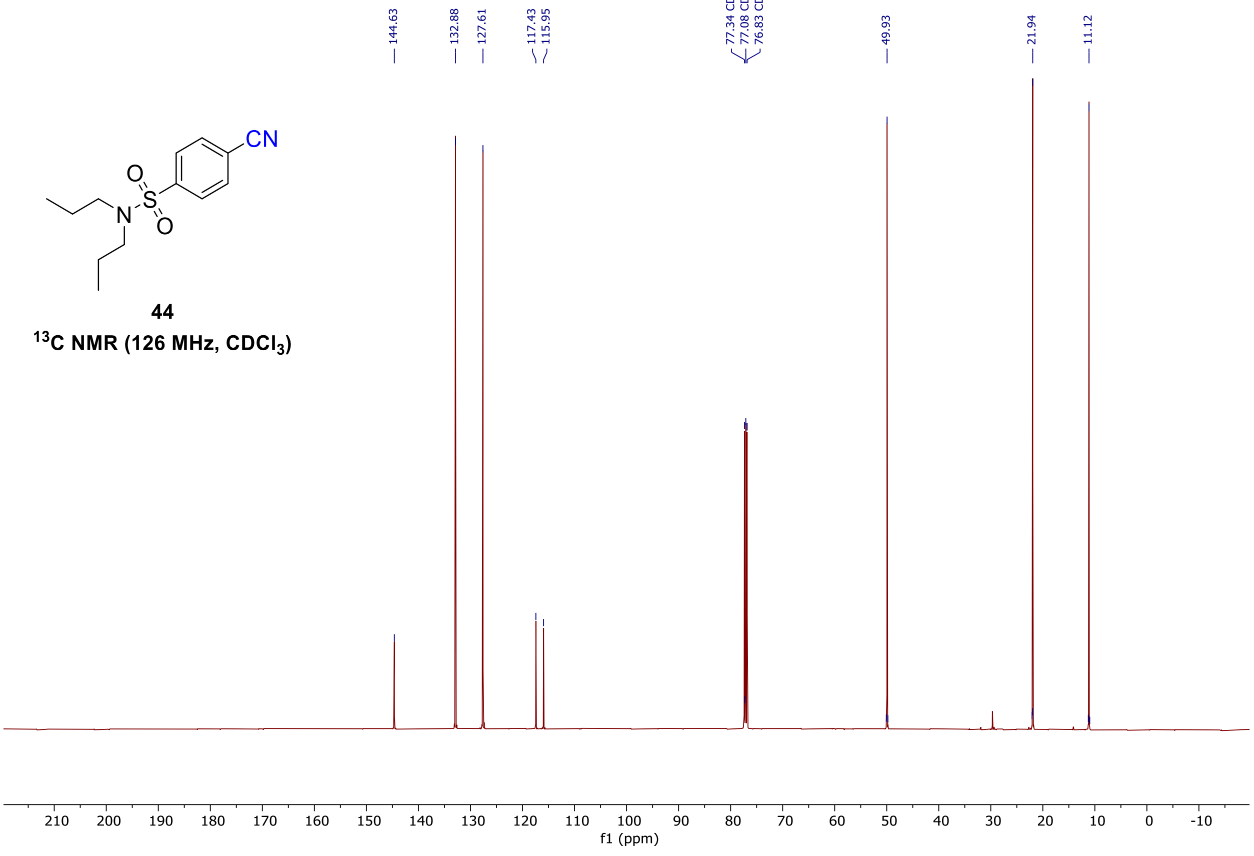

44

^{13}C NMR (126 MHz, $CDCl_3$)

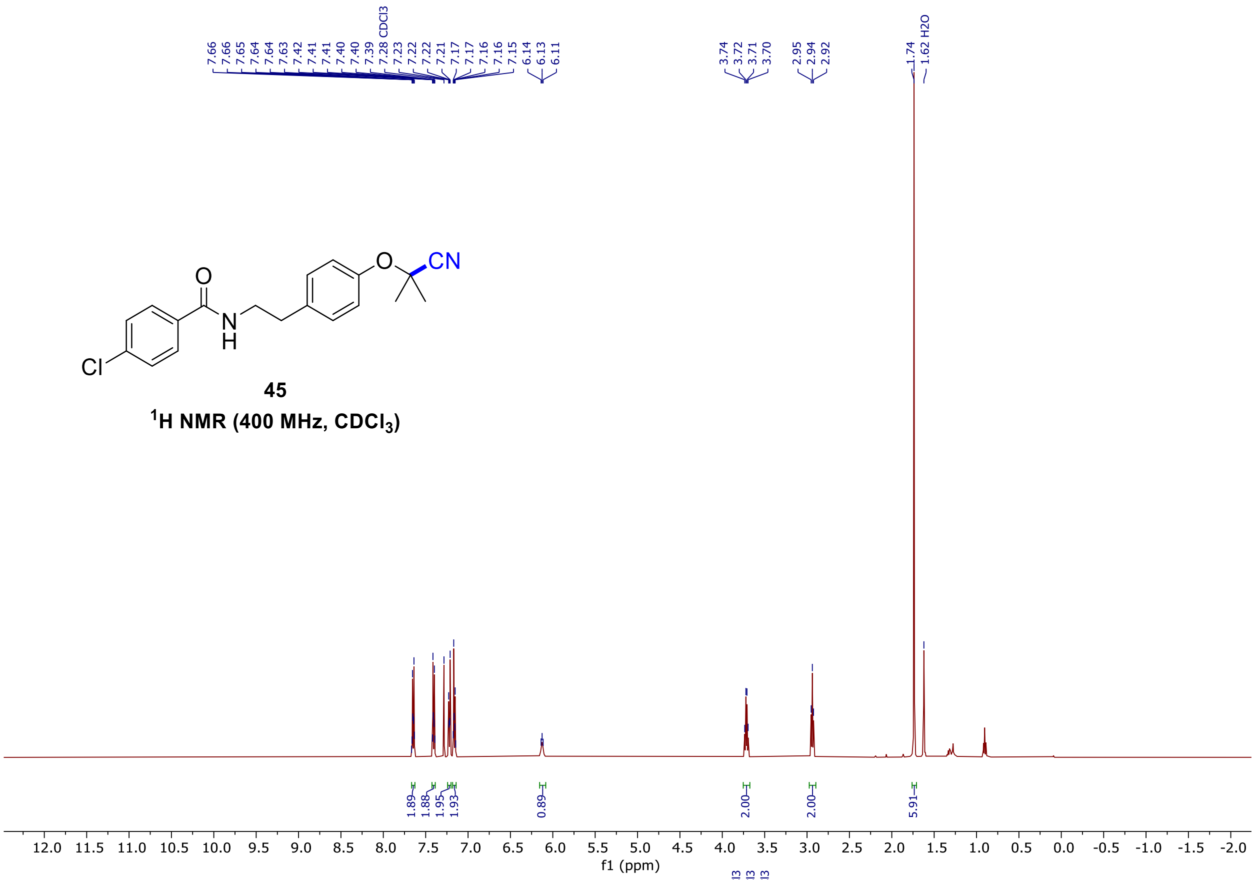

45

^{1}H NMR (400 MHz, $CDCl_3$)

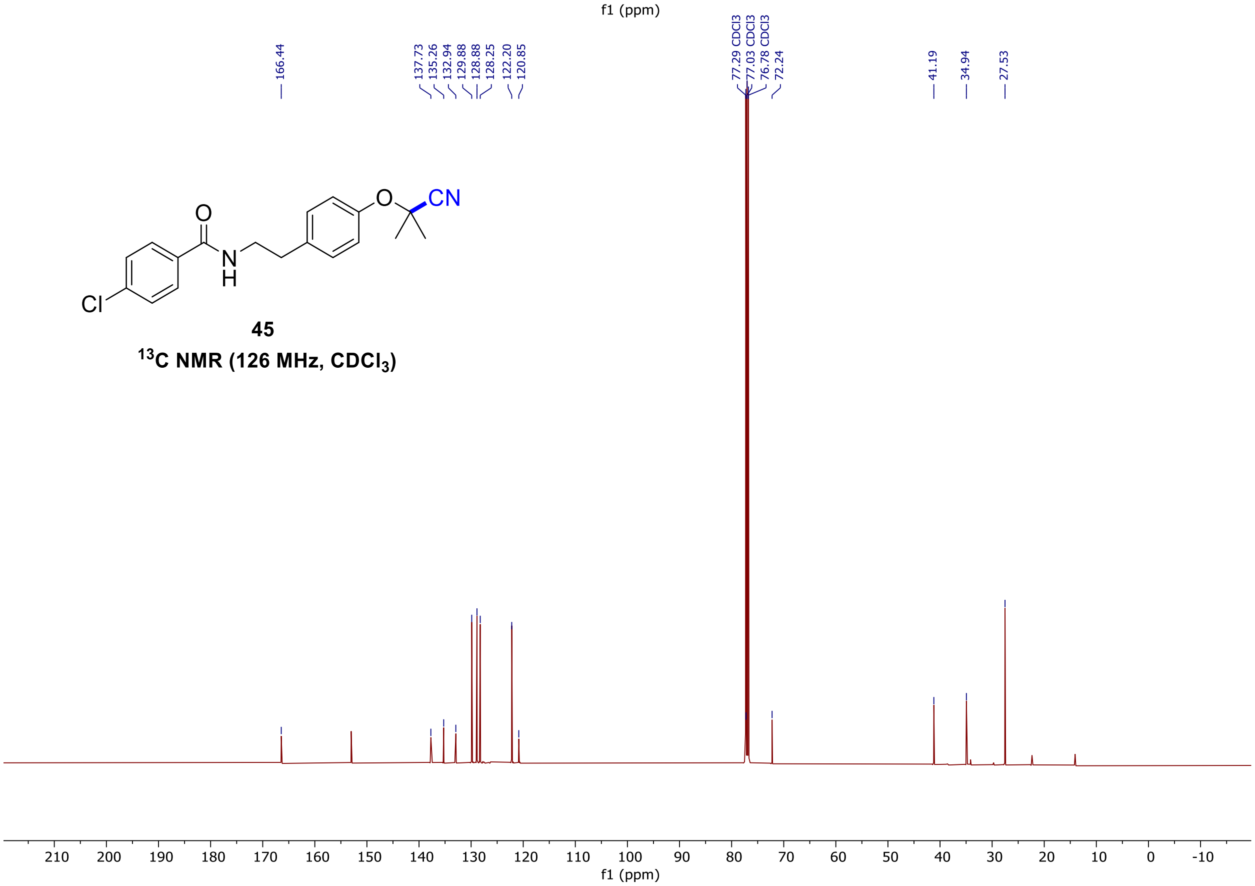

45

^{13}C NMR (126 MHz, $CDCl_3$)

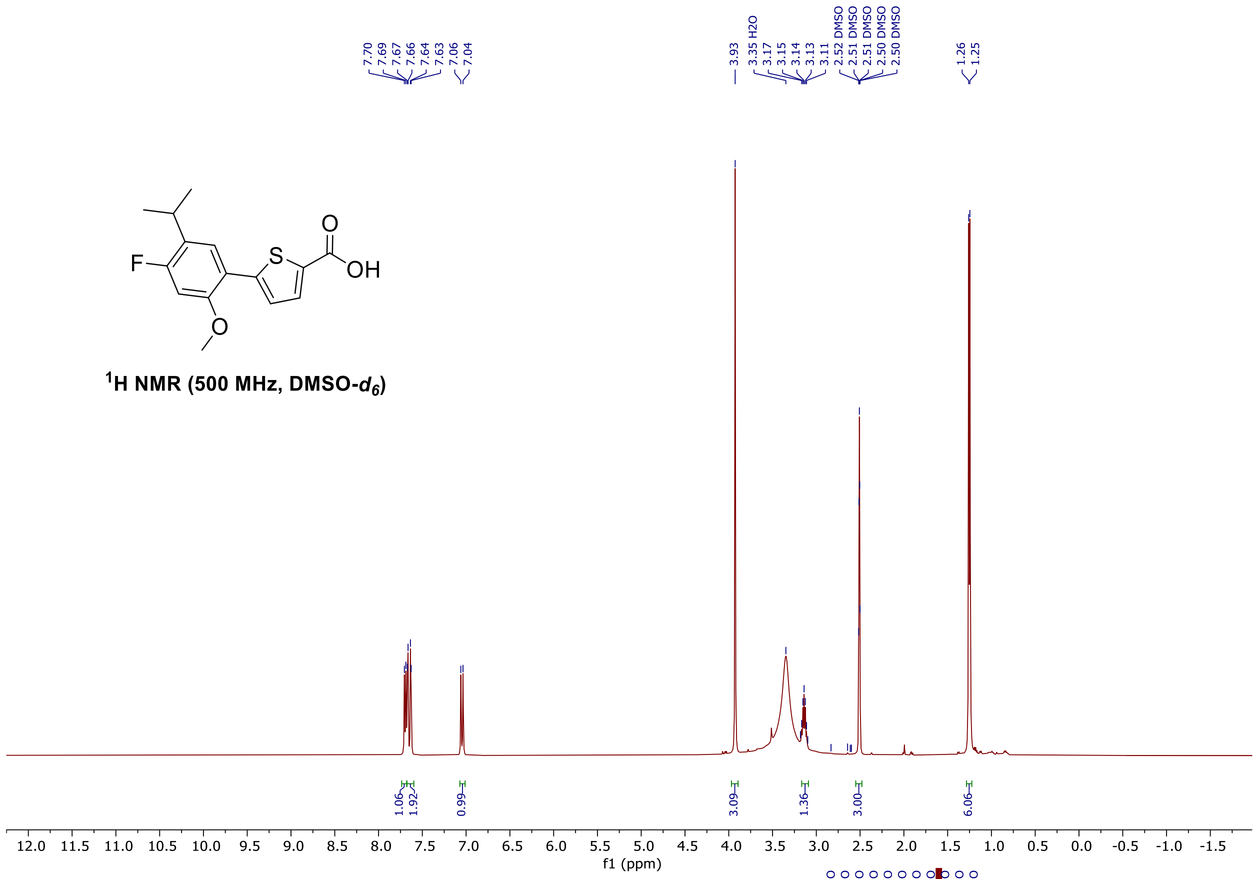
7.70 7.69 7.67 7.66 7.64 7.63 7.06 7.04
3.93 3.35 H2O 3.17 3.15 3.14 3.13 3.11 2.52 DMSO 2.51 DMSO 2.51 DMSO 2.50 DMSO 2.50 DMSO
1.26 1.25
F
S
O
OH
O
^{1}H NMR (500 MHz, DMSO-d_6)
1.06 1.92 0.99 3.09 1.36 3.00 6.06
12.0 11.5 11.0 10.5 10.0 9.5 9.0 8.5 8.0 7.5 7.0 6.5 6.0 5.5 5.0 4.5 4.0 3.5 3.0 2.5 2.0 1.5 1.0 0.5 0.0 -0.5 -1.0 -1.5
f1 (ppm)

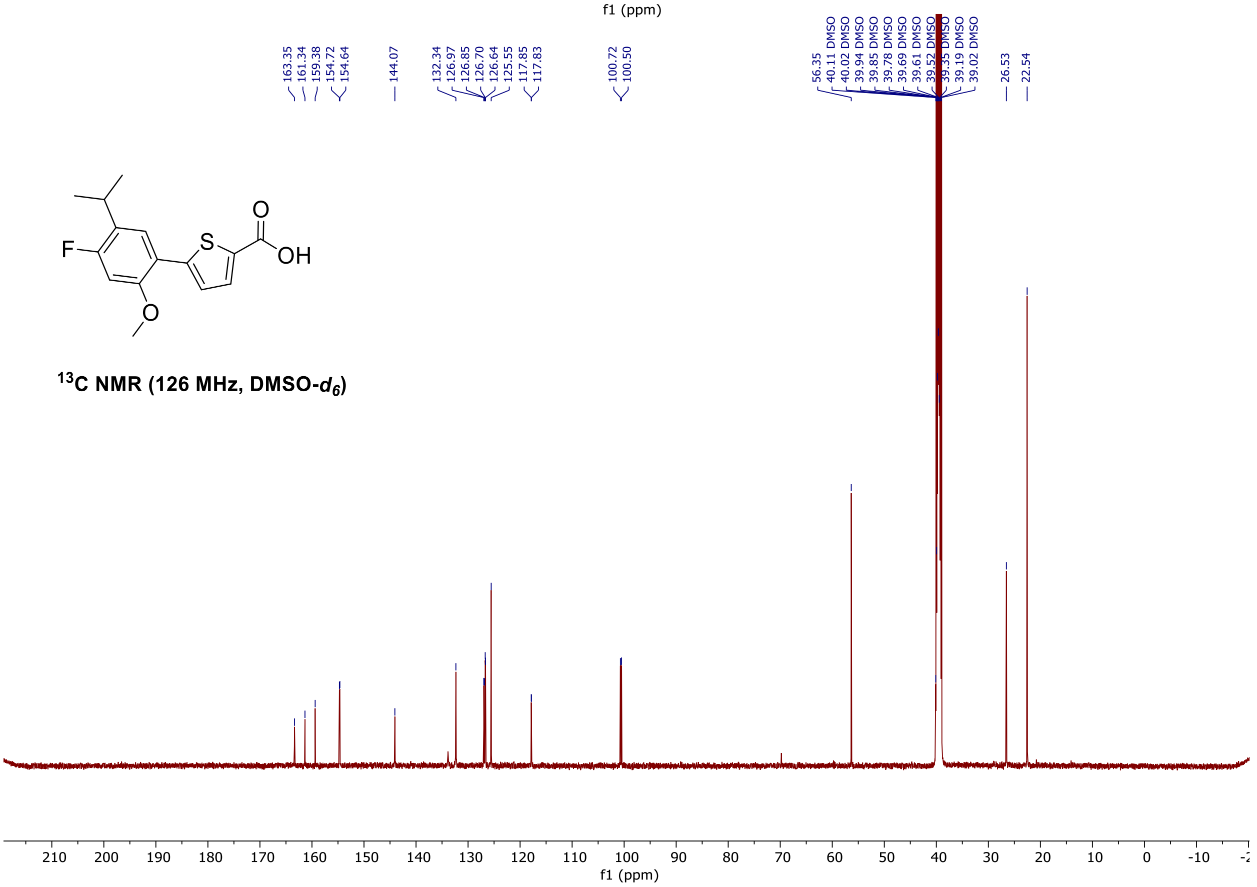
163.35 161.34 159.38 154.72 154.64 144.07 132.34 126.97 126.85 126.70 126.64 125.55 117.85 117.83 100.72 100.50
56.35 40.11 DMSO 40.02 DMSO 39.94 DMSO 39.85 DMSO 39.78 DMSO 39.69 DMSO 39.61 DMSO 39.52 DMSO 39.35 DMSO 39.19 DMSO 39.02 DMSO 26.53 22.54
F
S
O
OH
O
^{13}C NMR (126 MHz, DMSO-d_6)
210 200 190 180 170 160 150 140 130 120 110 100 90 80 70 60 50 40 30 20 10 0 -10
f1 (ppm)

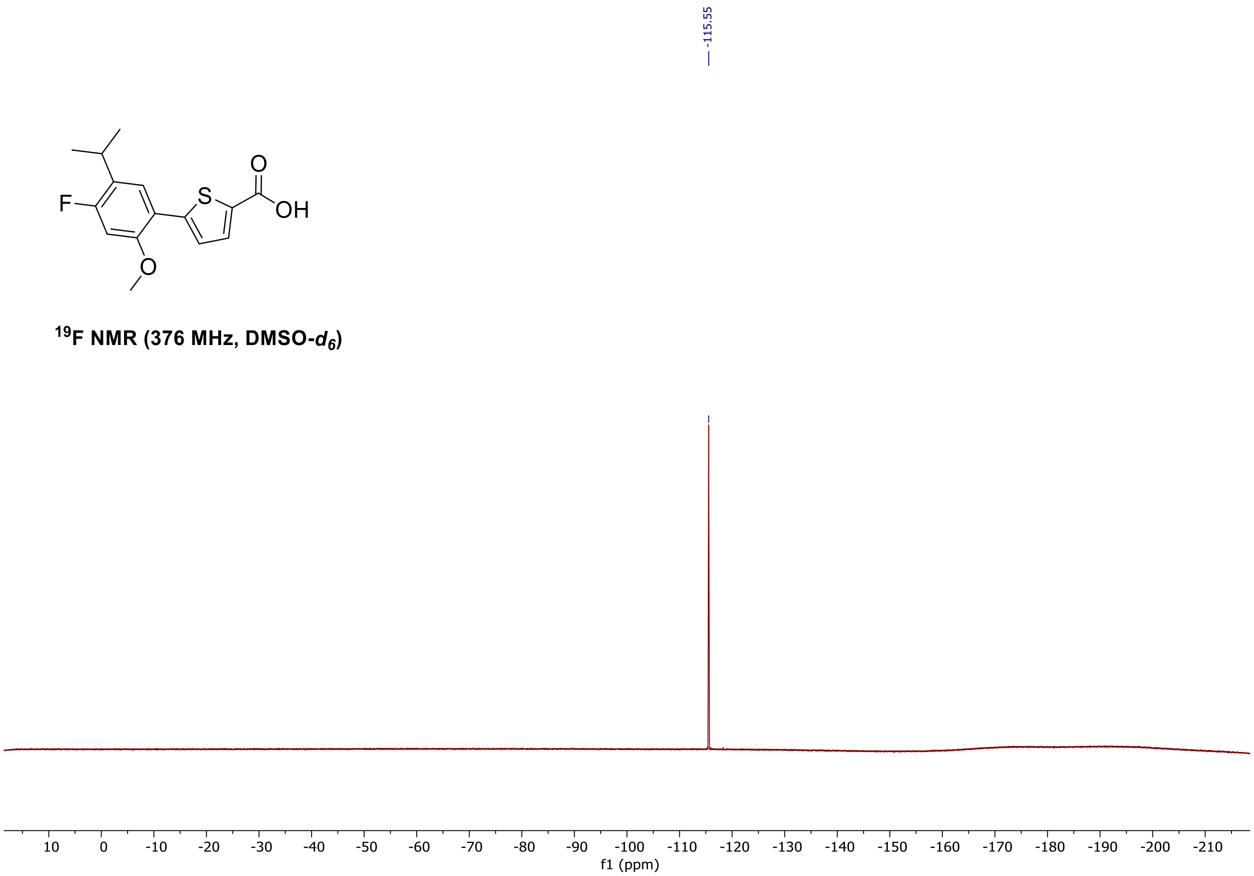

-115.55
F
S
O
OH
O
^{19}F NMR (376 MHz, DMSO-d_6)
10 0 -10 -20 -30 -40 -50 -60 -70 -80 -90 -100 -110 -120 -130 -140 -150 -160 -170 -180 -190 -200 -210
f1 (ppm)

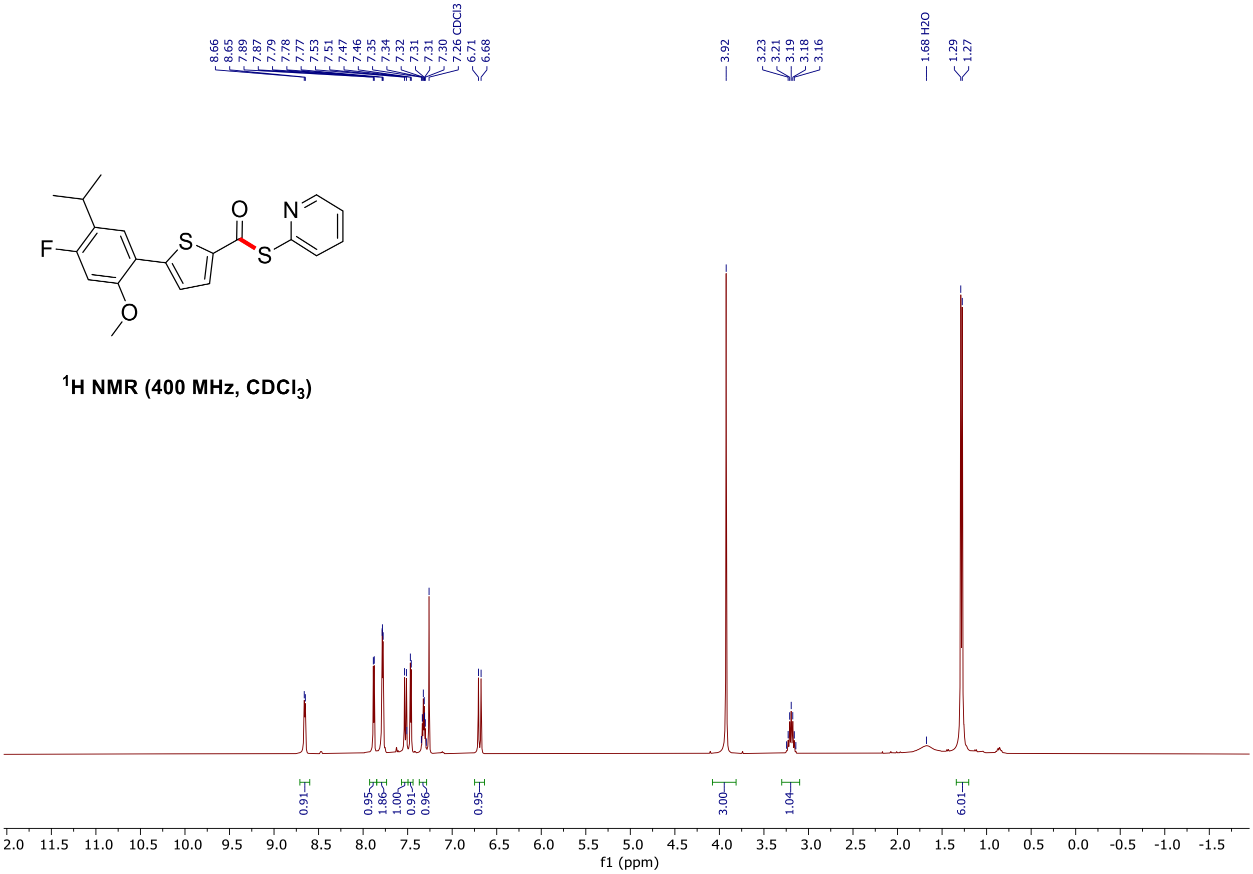

8.66 8.65 7.89 7.87 7.79 7.78 7.77 7.53 7.51 7.47 7.46 7.35 7.34 7.32 7.31 7.31 7.30 7.26 CDCl3 6.71 6.68
3.92
3.23 3.21 3.19 3.18 3.16
1.68 H2O
1.29 1.27
F
S
O
N
S
O
^{1}H NMR (400 MHz, CDCl$_3$)
0.91 0.95 1.86 1.00 0.91 0.96 0.95 3.00 1.04 6.01
2.0 11.5 11.0 10.5 10.0 9.5 9.0 8.5 8.0 7.5 7.0 6.5 6.0 5.5 5.0 4.5 4.0 3.5 3.0 2.5 2.0 1.5 1.0 0.5 0.0 -0.5 -1.0 -1.5
f1 (ppm)

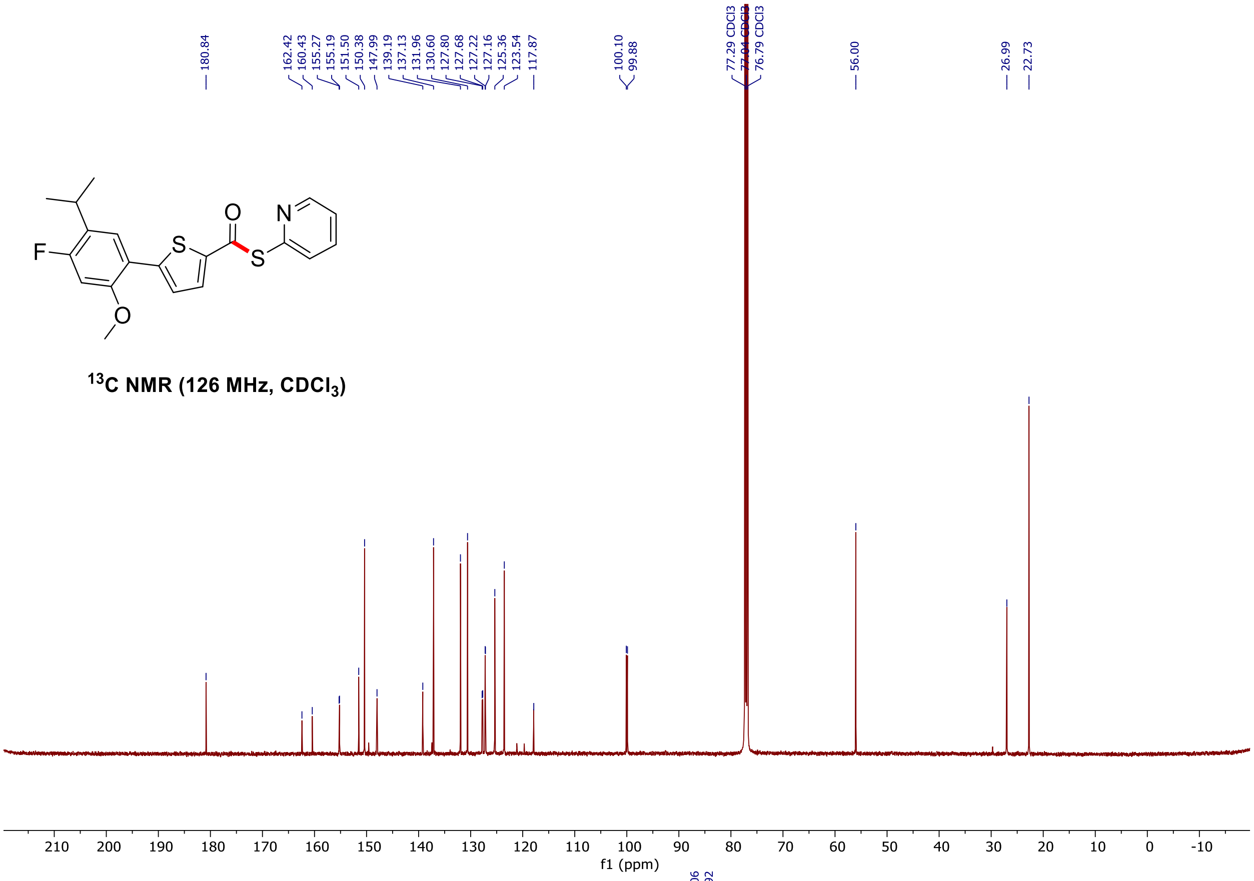
180.84
162.42
160.43
155.27
155.19
151.50
150.38
147.99
139.19
137.13
131.96
130.60
127.80
127.68
127.22
127.16
125.36
123.54
117.87
100.10
99.88
77.29 CDCl3
76.79 CDCl3
56.00
26.99
22.73
O
N
S
S
F
O
^{13}C NMR (126 MHz, CDCl$_3$)
f1 (ppm)

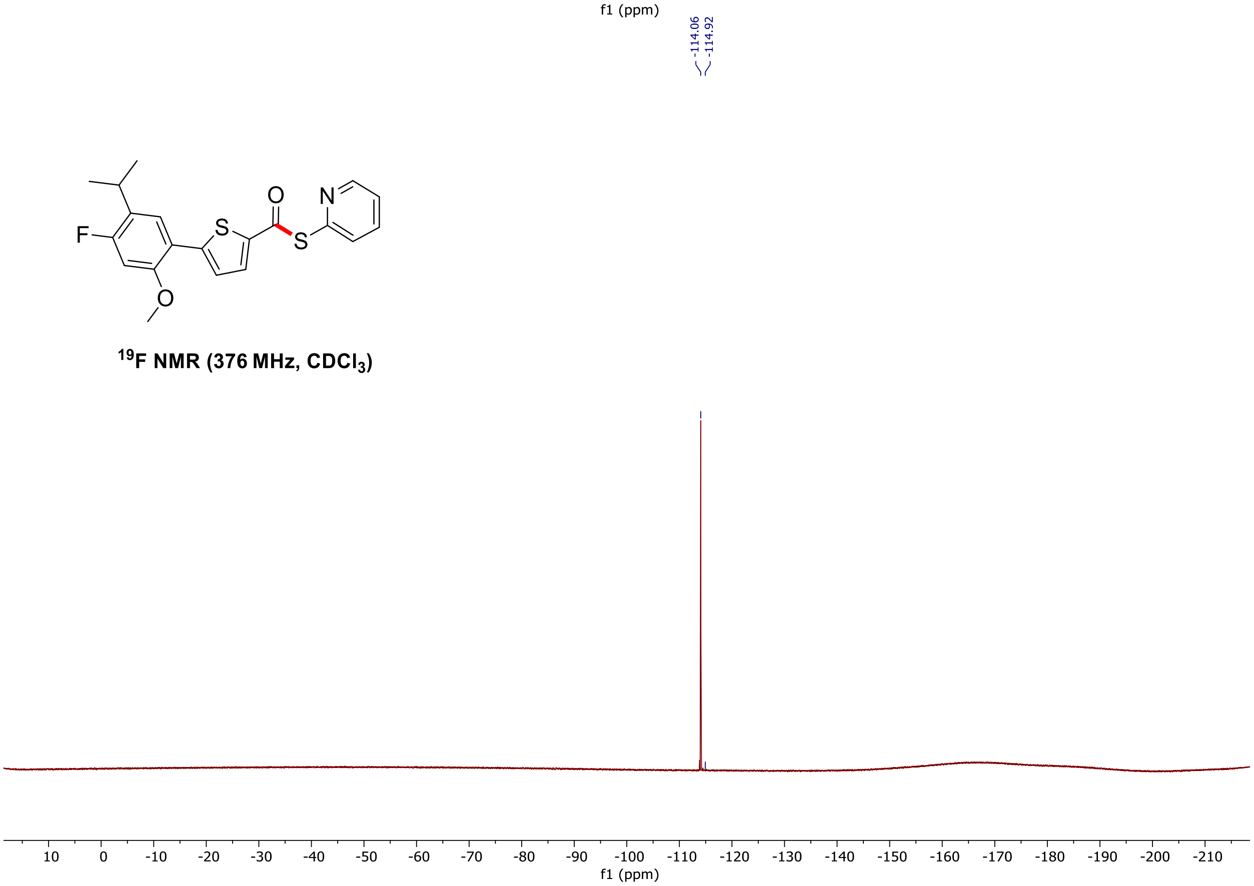
-114.06
-114.92
O
N
S
S
F
O
^{19}F NMR (376 MHz, CDCl$_3$)
f1 (ppm)

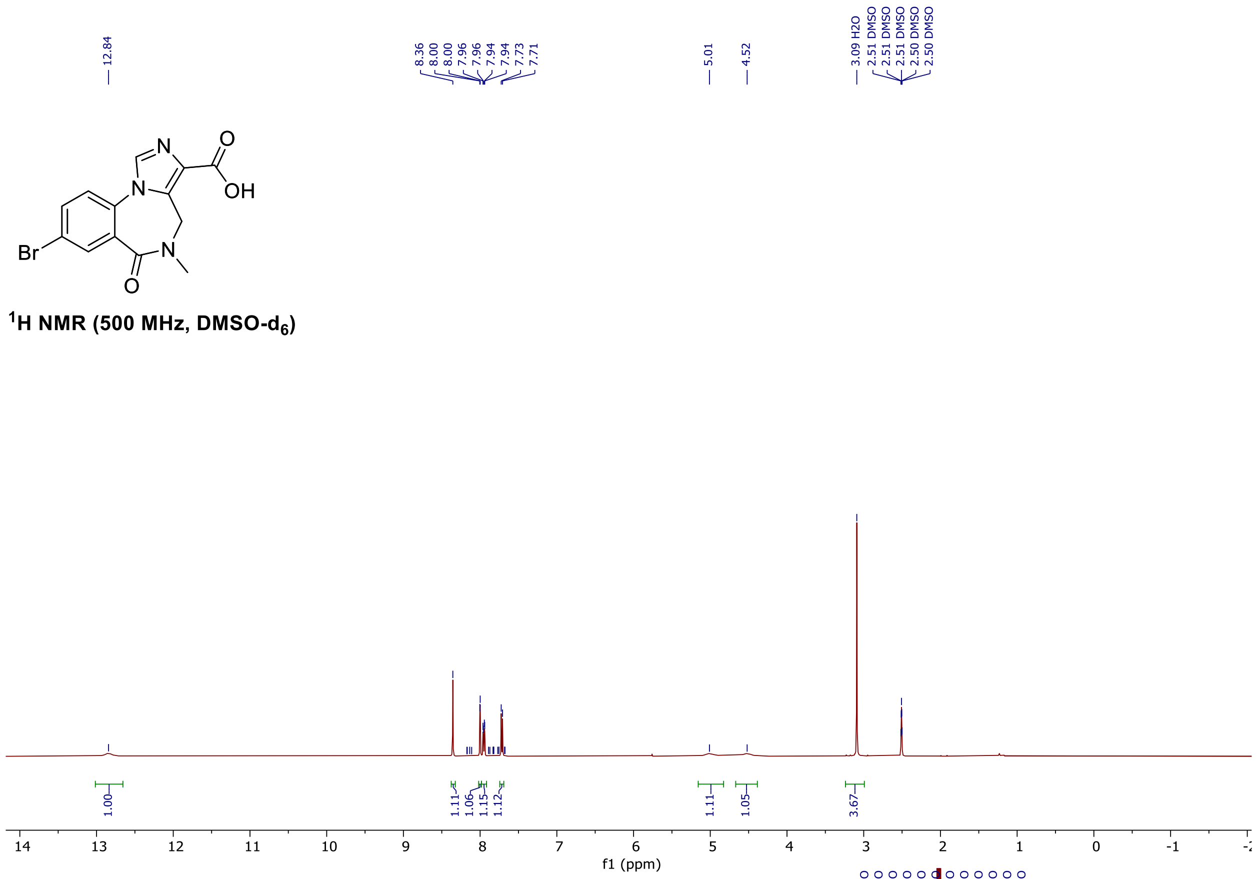

^{1}H NMR (500 MHz, DMSO-d$_6$)

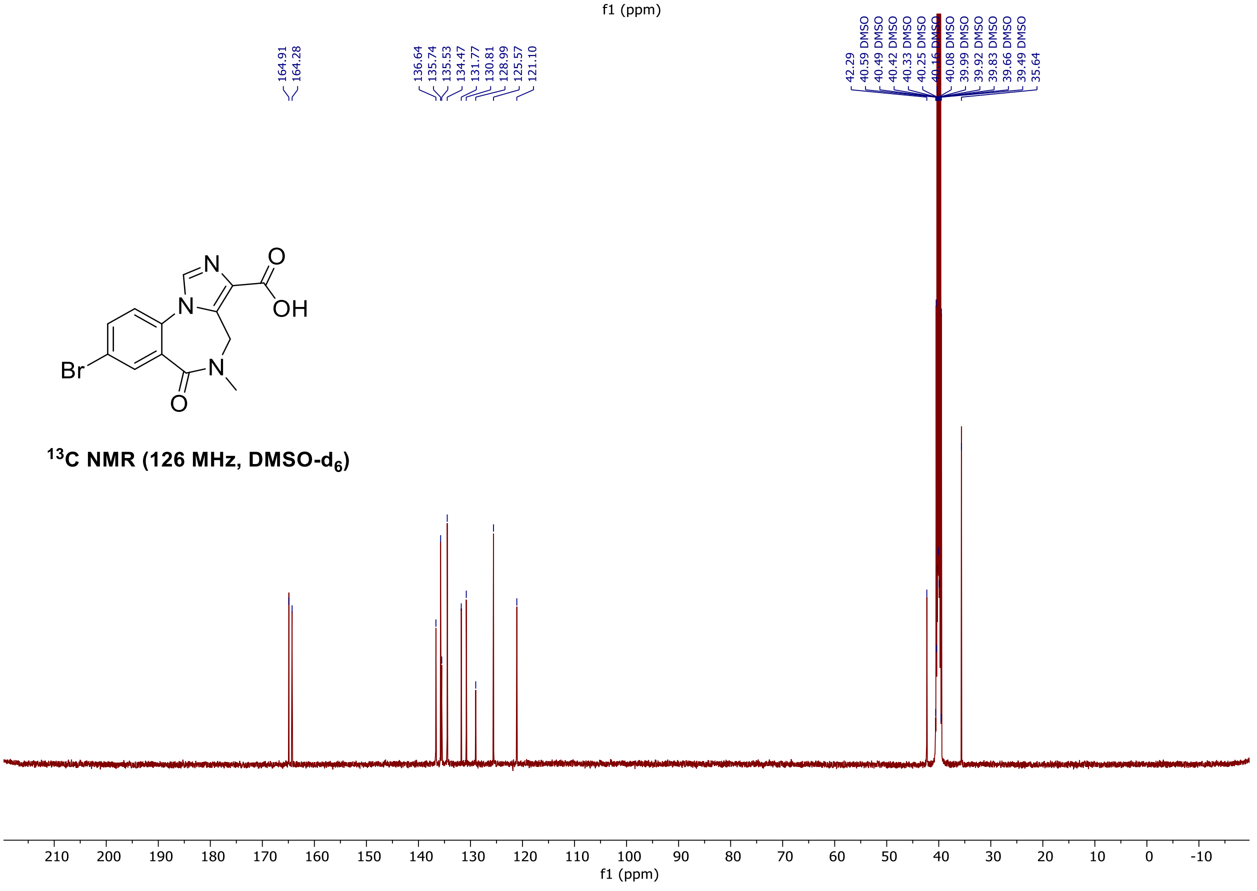

^{13}C NMR (126 MHz, DMSO-d$_6$)

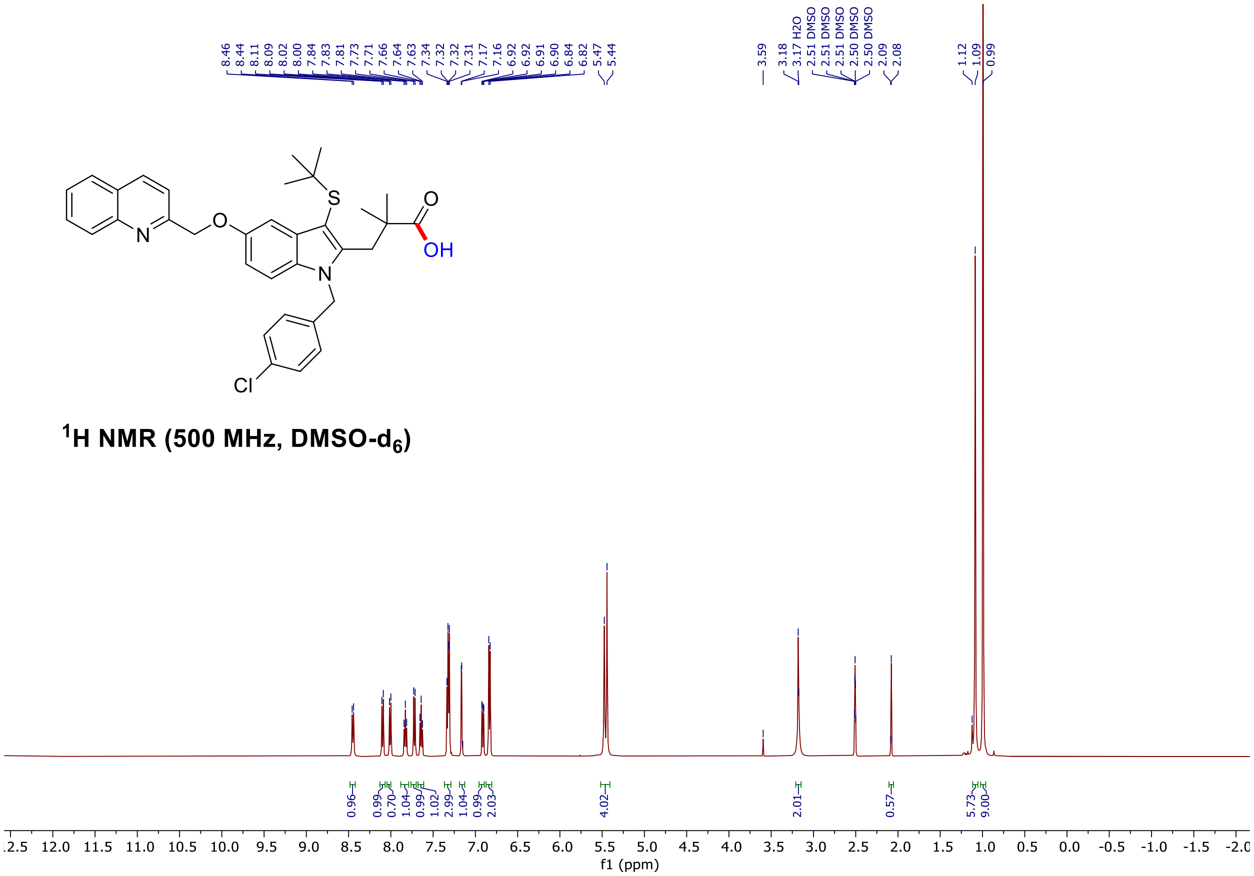
8.46 8.44 8.11 8.09 8.02 8.00 7.84 7.83 7.81 7.73 7.71 7.66 7.64 7.63 7.34 7.32 7.32 7.31 7.17 7.16 6.92 6.92 6.91 6.90 6.84 6.82 5.47 5.44
3.59 3.18 3.17 H2O 2.51 DMSO 2.51 DMSO 2.51 DMSO 2.50 DMSO 2.50 DMSO 2.09 2.08
1.12 1.09 0.99
O
OH
S
N
O
N
Cl
^{1}H NMR (500 MHz, DMSO-d$_6$)
0.96 0.99 0.70 1.04 0.99 1.02 2.99 1.04 0.99 2.03 4.02 2.01 0.57 5.73 9.00
f1 (ppm)

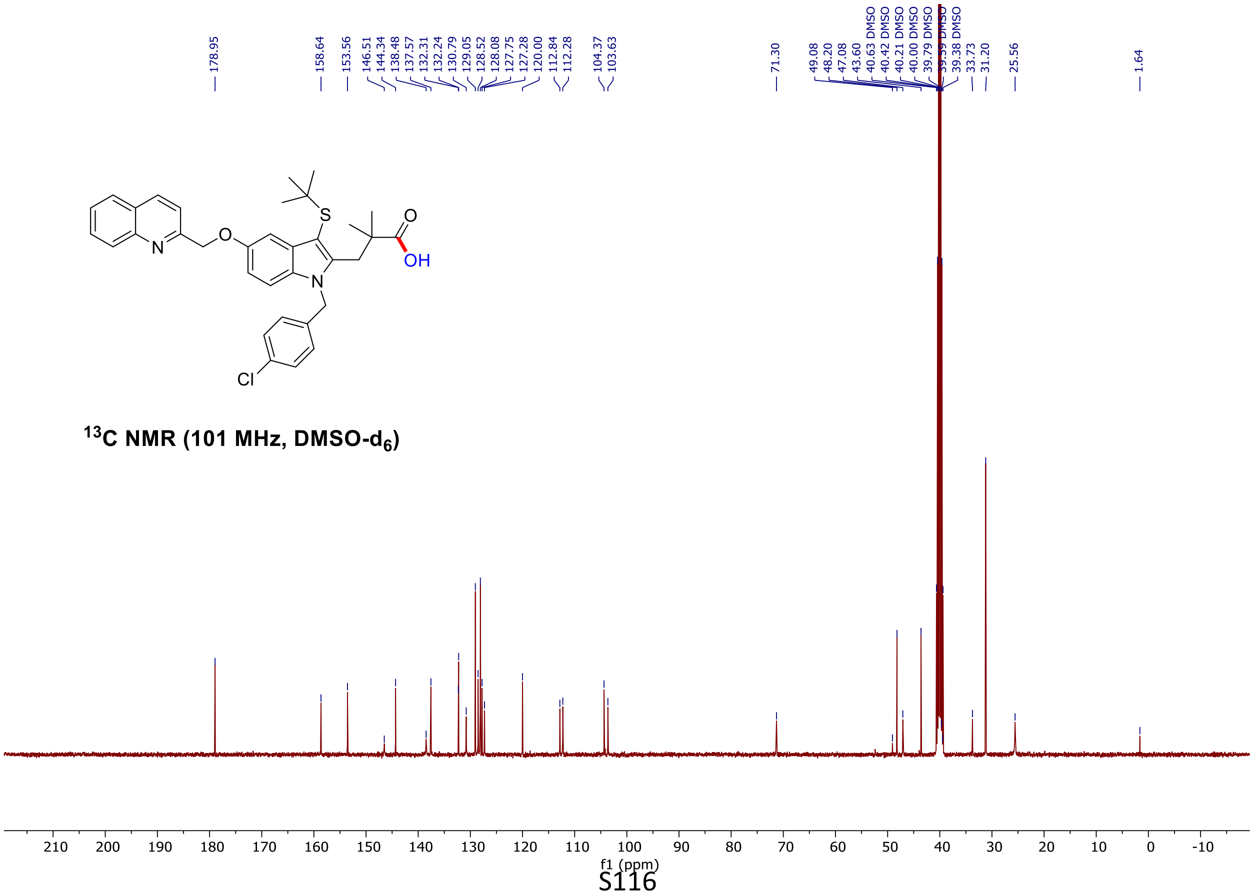
178.95
158.64 153.56 146.51 144.34 138.48 137.57 132.31 132.24 130.79 129.05 128.52 128.08 127.75 127.28 120.00 112.84 112.28 104.37 103.63
71.30
49.08 48.20 47.08 43.60 40.63 DMSO 40.42 DMSO 40.21 DMSO 40.00 DMSO 39.79 DMSO 39.59 DMSO 39.38 DMSO 33.73 31.20 25.56
1.64
O
OH
S
N
O
N
Cl
^{13}C NMR (101 MHz, DMSO-d$_6$)
f1 (ppm)
